# Understanding veterinary practitioners’ responses to adverse events using a combined grounded theory and netnographic natural language processing approach

**DOI:** 10.1371/journal.pone.0314081

**Published:** 2024-12-05

**Authors:** Julie Gibson, Catherine Oxtoby, Marnie L. Brennan, Kate White

**Affiliations:** 1 School of Veterinary Medicine and Science, University of Nottingham, Nottingham, United Kingdom; 2 Centre for Evidence-based Veterinary Medicine, University of Nottingham, Nottingham, United Kingdom; 3 Veterinary Defence Society, Knutsford, United Kingdom; Kitami Institute of Technology, JAPAN

## Abstract

Support that mitigates the detrimental impact of adverse events on human healthcare practitioners is underpinned by an understanding of their experiences. This study used a mixed methods approach to understand veterinary practitioners’ responses to adverse events. 12 focus groups and 20 interviews with veterinary practitioners were conducted and analysed using grounded theory principles. Experiencing stress, externalising facts and feelings, morally contextualising events and catalysing personal and professional improvements were identified as components of practitioners’ response. Natural language processing content analysis of posts regarding involvement in adverse events (n = 572) written by members of a veterinary member-only Facebook group was also performed, to categorise and count words within texts based on underlying meaning. Percentile scores of four summary variables along with relative frequency of function, psychological process and time orientation words used were recorded and compared with content analysis of posts where members discussed euthanasia (n = 471) and animal health certification (n = 419). Lower authenticity scores (reflecting lower honesty), differences in clout scores (reflecting dominance) and higher frequencies of moralisation, future focus, prosocial behaviour and interpersonal conflict were observed in the adverse event group compared to either comparison group. Analytical thinking scores (reflecting logical thinking) and frequencies of total, positive and negative emotion, anxiety, anger and cognitive processing words (reflecting debate) were not significantly different between the adverse events and euthanasia groups. Integration of findings confirmed and expanded inferences made in both studies regarding the emotionally detrimental impact of adverse events and the role that peer-to-peer mediated reflection and learning plays in mitigating pathologisation of responses in the aftermath of adverse events. Discordance in findings related to practitioners’ intentions and expressions of honesty suggest that work is needed to normalise open discussion about adverse events. Findings may be used to lever, and to inform, peer-to-peer support for practitioners in relation to veterinary adverse events.

## Introduction

Adverse events can have profound personal and professional consequences for human healthcare practitioners, threatening workforce wellbeing, contributing to career attrition and impeding the delivery of safe sustainable healthcare [1]. In recognition of this, human healthcare support programs [2–6], toolkits [7], websites and online training courses [8] are plentiful and ever evolving [9]. Such support is based on extensive research explicating the experiences, responses and recovery of medical practitioners who are involved in adverse events [10,11].

Since coined over two decades ago and despite contestation that the term unhelpfully reflects a self-pitying professional identity [12], *second victim* [13] is a steadfast, succinct and internationally employed descriptor of human healthcare practitioners who are detrimentally affected by adverse events. Studies indicate that during their career, almost 80% of healthcare practitioners are involved in at least one adverse event or near miss that impacts them emotionally [14,15]. Frequently cited ‘symptoms’ include flashbacks, concentration issues, sleep disturbance and loss of self-confidence. Suggestion that second victims have an increased risk of developing burnout [16,17], post-traumatic stress disorder (PTSD) [18–21], a self-harming dependency on alcohol and drugs [22] and suicidal thoughts [23] focuses concern for affected practitioners’ longer term psychological state. Numerous studies highlight the persistent emotional burden that adverse event involvement may inflict on healthcare practitioners. Over a quarter of 4369 respondents in a Netherlands based survey study experienced stress for at least a month following involvement in an adverse event, with some continuing to feel shameful, fearful, uncomfortable working within the team, unhappy and dejected for in excess of a year [24]. Such emotional reactions not only have implications for practitioners’ wellbeing but have been shown to negatively influence the way healthcare is delivered for protracted periods. In the same study over 45% of respondents indicated that they were unable to provide quality care for between a month and a year following an adverse event [24].

Although organisation led support for healthcare practitioners who are involved in adverse events is driven by ethical obligation to alleviate practitioners’ distress, it is also necessary if the cycle of repeat adverse event occurrence is to be broken. Second victims are thought to be more likely to practise defensive medicine which inadvertently jeopardises the safety of future patients [25]. Nearly 3% of those affected profess to an inability to provide quality care to patients for an indefinite time after the event [24]. Some sufferers report leaving their job as a direct result [16] with further implications for team performance and workforce sustainability. By supporting practitioners in a way that reduces fear and encourages transparency about ‘systems’ factors that may have contributed, organisational lessons can be learnt and mitigating improvements made.

Evidence that adverse events also represent a source of stress for veterinary practitioners is emerging. In a 2018 survey of Veterinary Information Network (VIN) members, over three quarters of respondents reported a degree of impact on both their professional and personal life following involvement in an adverse event, with over half declaring that these effects had lasted over one week [26]. Over a third of respondents involved in an adverse event in the previous twelve months reported difficulties with sleeping, loss of concentration and feelings of decreased overall happiness, as well as perceptions of burnout. Many experienced reduced professional confidences, a tendency to question career choice, and a reluctance to speak up about adverse events, findings that are reflected in qualitative studies [27,28]. Ethical challenges experienced as a result of adverse events may predispose to peer-peer and veterinary-client relationship strain [29]. Associated complaints not only compound distress but may concerningly contribute to defensive behaviours, increasing the risk of future adverse event occurrence [30,31]. Little is documented about veterinary practitioners’ specific coping strategies in the aftermath of adverse events but in a small-scale qualitative survey of thirty-two spay-neuter veterinary practitioner respondents, experienced support was identified as a factor influencing the development of trauma or resilience [27]. Low and Wu (2022) also provide a powerful example of the positive influence collegiality has on practitioners who are involved in adverse events [32]. Institution led support programs mirroring those available in human healthcare have been suggested as a potential solution [26,28,33] but to meet veterinary practitioners nuanced needs, an evidence-based understanding of their response to adverse events is needed.

Scant existing research into veterinary practitioners’ experiences of adverse events is limited to focus groups that are subject to the influences of group dynamics, and survey techniques that may limit the depth of understanding achieved. One-to-one interviews where researchers develop a trusting and confidential space for participants to share their experiences facilitate deeper exploration of emotions but may still be limited by social desirability bias introduced by researcher presence [34]. Naturalistic data sources, where the study population lacks real time knowledge of research activity, may be utilised to navigate these limitations and are of particular use in the study of sensitive topics. Text written online is a valuable source of such data that has previously been used to investigate infertility [35], peri natal depression [36], drug use [37] and breast-feeding support [38].

Netnography is an adaptation of ethnography which is used to study social interactions in a digital communications context [39]. According to the Office for National Statistics, ninety-six percent of households in Great Britain had internet access in 2020, with eighty nine percent of individuals reporting daily connection [40,41]. Social networking entails real and digital world communication and relationship formation. Online spaces enable connection and interaction between individuals that may never have met otherwise. Usage of social networking sites (SNS) in adults in the United Kingdom (UK) soared to seventy percent in 2020, with Facebook (Meta Platforms, Inc.) being most prolific at the time of writing [42]. Text written by users of Facebook provides a source of naturalistic data, which overcomes bias associated with researcher presence. Vast amounts of data are often readily available, countering limitations imposed by time when generating qualitative data by means of interview and focus groups. Although Facebook provides a source of research data [43], ownership of such online content is a grey area. Despite widespread awareness amongst Facebook users that their data may not be private [44], collecting, analysing and reporting qualitative data risks inadvertent identification and traceability of individuals, which potentially breeches research integrity. Anonymised automated analysis and reporting of solely quantitative data mitigates this risk.

Natural language processing (NLP) is a form of artificial intelligence (AI) where computers are programmed to understand and, or, to generate human language [45]. NLP can be used to understand the sentiment of digital texts and a range of computer aided text analysis (CATA) tools are commercially available for this purpose. Such tools conduct automated content analysis based on the linguistic premise that language reflects the psychological state of those using it. For example, high relative frequency of first-person singular pronouns (e.g. I, me, my) is associated with depression [46] and high relative frequency of negations (no, not, never) or conjunctions (because, but, besides) with duty and obligation [47]. By interpreting CATA outputs, inferences can be made about emotions, behaviours and motivations of individuals or groups in relation to topics under study.

Due to the ability to unobstructively collect and analyse large volumes of textual data in the digital sphere, netnographic NLP is a rapidly advancing research field. Although so far limited in the veterinary sector, such an approach was recently used to analyse the content of online discussion fora to understand farmer decision-making regarding antibiotic usage in the United Kingdom [48]. Netnographic NLP has neither been used to explore Facebook content generated by veterinary practitioners, nor triangulated with analyses of interview and focus group data to specifically understand their psychological response to adverse events.

There were therefore three aims in this research: (1) to contribute to the sparse literature surrounding veterinary practitioners’ experiences of adverse events by understanding their response in the aftermath (2) to harness veterinary practitioners’ use of Facebook to demonstrate netnographic NLP content analysis as an adjunct to more traditional interview and focus group approaches and, (3) to explore the role that social networking plays in veterinary practitioners’ responses to adverse events.

## Materials and methods

A mixed methods approach was utilised. Firstly, an entirely qualitative study using focus groups and interviews was conducted. Data collection and analysis were guided by constructivist grounded theory principles [49–51]. Secondly, qualitative online textual data extracted from Facebook were analysed quantitatively using NLP content analysis. Integration of qualitative and quantitative data is a pillar of mixed methods research [52,53], which lends to gaining broad and deep perspectives of topics and is often said to create more trustworthy findings [54]. This study design is underpinned by a pragmatic dialectal pluralism philosophy, which commits to engagement with multiple research paradigms to enhance understanding of topics [55,56]. A reflexive approach was taken meaning that the researchers critically examined the impact of their identity and positionality on the research process and findings [57]. Throughout the study (part one and part two), adverse events were defined as events where veterinary patients had incurred some degree of physical harm as a consequence of veterinary care rather than as a direct result of the underlying condition or disease for which they were presented [58].

### Part one methods: A grounded theory approach employing interview and focus groups

In the first part of the study a grounded theory (GT) approach to data collection and analysis was employed. GT approaches are based on simultaneous, inductive and iterative principles; data analysis is concurrent with data collection, is not confined by predetermined ideas and informs further data collection and analytic direction [51,59].

#### Study sample and recruitment

Recruitment of focus group and interview participants took place between 31^st^ October 2019 and 24^th^ May 2022. Initially, a purposive sampling [60] technique was used to invite veterinary practitioners to participate in focus groups about their experiences of adverse events. A broad range of perspectives were sought at this stage. Inclusion criteria was defined as any veterinary surgeon, registered or student veterinary nurse currently working within a veterinary clinical or management role within the United Kingdom. The researchers verbally informed personal contacts, who were in veterinary leadership positions in twelve equine, farm, small animal and mixed first opinion and referral practices located across the United Kingdom (UK), about the research. Permission was granted to place a poster, which provided information about the research and an invitation to participate in a focus group regarding veterinary practitioners’ experiences of adverse events.

Social desirability bias [34] and the effects of hierarchy observed within the focus groups, led to the decision to conduct interviews with additional participants. Sampling was in part theoretical as it was informed by the developing analysis but purposive and snowball sampling were also used [60]. Firstly, veterinary practitioners who had specifically experienced an adverse event with an accompanying client complaint were recruited in three ways (i) by verbal invitation by the primary author at an online conference presentation about the research (ii) using a snowballing technique where existing participants were invited to ask further potential participants to contact the primary researcher about participation in the study and, (iii) in collaboration with the Veterinary Defence Society (VDS; the UK’s largest provider of veterinary professional indemnity insurance) who contacted potential participants by phone between and provided them with information about the study and contact details of the primary researcher. Finally, veterinary practitioners self-identifying to be emotionally impacted by involvement in an adverse event were recruited. This was achieved by publishing a one-off recruitment post on the Veterinary Voices Facebook Group (details of the group are documented in the second part of the study within this manuscript).

#### Data collection technique

Focus groups were conducted in person within the practice where the participants worked and information about attendees is presented in Table 1. The duration of focus group discussion ranged from 28–103 minutes.

**Table 1:** Details of focus groups conducted to explore veterinary practitioners’ experiences of adverse events.

Focus Group number	Number of participants	Type of practice and personnel of participants attending the group.
Focus Group 1	6	Veterinary surgeons only. Privately owned first opinion mixed practice.
Focus Group 2	7	Veterinary surgeons and registered veterinary nurses. Privately owned first opinion mixed practice.
Focus Group 3	5	Registered and student veterinary nurses. Privately owned small animal referral hospital.
Focus Group 4	5	Veterinary surgeons (senior level) and registered veterinary nurses. Privately owned small animal referral hospital.
Focus Group 5	8	Veterinary surgeons only. Corporately owned equine referral hospital.
Focus Group 6	7	Veterinary surgeons and registered veterinary nurses. Corporately owned equine referral hospital.
Focus Group 7	5	Veterinary surgeons (senior level) and registered veterinary nurses. Corporately owned first opinion small animal practice.
Focus Group 8	5	Veterinary surgeons (< 8years qualified). Corporately owned first opinion small animal practice.
Focus Group 9	6	Registered and student veterinary nurses. Corporately owned first opinion small animal practice.
Focus Group 10	4	Registered and student veterinary nurses. Privately owned first opinion mixed practice.
Focus Group 11	4	Veterinary surgeons (senior level). University teaching hospital.
Focus Group 12	7	Veterinary surgeons (resident and interns). University teaching hospital.

Information about participants involved in interviews, which were conducted by phone or by videoconference call (MS Teams) due to national COVID-19 pandemic restrictions at the time of data collection, are shown in Table 2. The duration of interviews ranged from 36–78 minutes.

**Table 2:** Details of interviews conducted to explore veterinary practitioners’ experiences of adverse events.

Interview	Type of adverse event that interview participants were involved in.
1	Adverse event with accusation of negligence and or misconduct and received advice +/- legal and/or professional disciplinary representation from VDS within previous two years.
2	
3	
4	
5	
6	
7	
8	
9	Adverse event with practice level disciplinary procedure.
10	
11	Adverse event with practice level client complaint.
12	
13	
14	
15	
16	Adverse event with self-identified emotional repercussions.
17	
18	
19	
20	

An intensive interviewing approach was used during all focus groups and interviews [59]. Intensive interviewing facilitates in-depth exploration of topics where interviewees have substantial lived experience. An open-ended question guide was created prior to each focus group and interview to keep focus on the topic but new ideas emerging in the course of discussions were also explored. Participants were asked to reflect on their experiences of involvement in emotionally impactful adverse events. Questions were tailored to explore uncertainties and to refine themes created as analysis concurrently progressed. All were audio recorded and transcribed by JG who facilitated each focus group, conducted each interview and recorded accompanying written field notes.

#### Data analysis

Transcripts were uploaded to NVivo12 Plus (QSR International), a software program for managing qualitative data, where coding took place. A constant comparative method [61] was employed. First, lines of transcript were read and assigned a label, or initial code, which reflected its meaning as extrapolated by JG. During a second stage, codes were compared with each other and with existing and further data. Codes and data relevant to a particular meaning were grouped together to produce focused codes. In a final theoretical coding stage, connections between the focused codes were identified to establish overarching subthemes and themes.

#### Ethical considerations in this study

The researchers collaborated with practitioners in management and leadership positions and VDS claims consultants to recruit participants who may have been difficult to access by other means. In addition to facilitating access to a rich source of research participants, these gatekeepers were deemed essential in navigating initial contact with individuals in relation to a topic that some could have found sensitive. The potentially negative impacts of research participation and findings were mitigated by providing advanced written information about the studies, opportunities to raise questions and concerns, the ability to decline or withdraw participation and signposting to follow up support on request. No incentives were offered, involvement was voluntary and took place after written consent was ascertained. Participants personal data was stored in a coded format and in line with the University of Nottingham’s General Data Protection (GDPR), Research Data Management (RDM) and Data Secure Handling Policies. Ethics approval for the qualitative phase of this study was granted by the University of Nottingham, School of Veterinary Medicine and Science ethics committee, approval number 2444180724 and 3184 200528.

### Part 2 methods: A netnographic approach utilising natural language processing content analysis of Facebook posts

In part 2 of the study, a netnographic NLP approach was utilised. Linguistic Inquiry and Word Count (LIWC-22) [62], a validated and commercially available CATA tool, was employed to conduct automated content analysis of text written in a specific Facebook group by veterinary practitioners about three different topics (i) adverse events (ii) animal health certification and, (iii) euthanasia.

#### Study sample

One large UK based Facebook group, Veterinary Voices UK (VV UK), was utilised for data collection. The group was created in 2017 by two veterinary surgeons campaigning to be elected as Royal College Veterinary Surgeons’ (RCVS) council members. RCVS is the statutory regulator of the veterinary profession in the UK and is governed by a council, which currently includes 13 veterinary surgeons elected by the profession. The aim of VV UK is to provide a platform for discussion about any topics relevant to veterinary surgeons and nurses working in the UK and to facilitate engagement with veterinary political organisations. The group is ‘closed’; only those who are members of the RCVS are permitted to join. Members contribute ‘posts’ of written text. Members are free to contribute new posts or to respond to existing posts to form threads. Non-members cannot see who members are, nor access any material that is posted or commented upon within the group. At the time of data collection, VV UK had nearly 18,000 members.

#### Data collection

JG is a member of the VV UK Facebook group and conducted the data collection. Data collection was unobstructive as data were collected retrospectively.

*Study group*: *Rationale and identification of posts containing discussion about adverse events*. Using the word search function on the VV Facebook page and setting the search period January 2018-December 2021, posts containing the terms ‘RCVS’, ‘VDS’ and ‘complaint’ were identified for inclusion within the adverse event group (AE group). The recruitment search period was set to start from one year from the creation of the group (Group created 11th February 2017) to the completion of data collection (31^st^December 2021). The start date was chosen in collaboration with the lead moderator of the group who suggested membership numbers, topic variety and engagement stabilised following the first year. The search terms were deduced *a priori* during the concomitant interview and focus group study (Part 1), which explored veterinary practitioners’ experiences of adverse events. Original posts and associated responses (from here collectively termed ‘posts’), were reviewed by JG to ensure relevance using the inclusion criterion that discussions were centred around veterinary practitioners’ experiences of adverse events. Data collection continued until all relevant posts within the search period were identified.

*Comparison groups*: *Rationale and identification of posts containing discussion about Animal Health Certification and euthanasia*. Using the same word search function and search period, posts containing the terms ‘animal health certificate’ or ‘AHC’ (AHC), and ‘euthanasia’ (Euth) were identified. It’s possible that posts could contain both a study and comparison group term, as adverse events can happen at the time of euthanasia or during certification. Where this was the case, the study group took precedence; posts were included in the AE study group and not in the AHC or Euth comparison groups if they contained discussion relevant to practitioners’ experiences of AE.

The comparison group topics were chosen in close collaboration with the lead moderator of the group who provided valuable insight into group activity and topic engagement. Offering different types of discourse to that about adverse events, AHC are a post Brexit government document needed for animal transport, which must be completed by veterinary surgeons. Anecdotally, they require thinking skills to complete and may cause stress. Euthanasia is a documented cause of emotional and moral stress [63–65]. Data collection was continued for both comparison groups until the number of author contributors was similar to that of the study group.

#### Data management

Identified posts from the study group authors were entered into an Excel spreadsheet. Each contributing author appeared in a single row (nAE) and each column contained a single post made by that author. The same process was used to enter posts from the two comparison groups (nAHC and nEuth). An author could be represented in either one, two or three rows, depending on whether they had contributed to the study group plus one or two of the comparison groups respectively. The study and comparison posts were numerically coded (1 = AE, 2 = AHC, 3 = Euth) and authors names removed prior to importing the spreadsheet into LIWC-22 [66], an automated NLP software programme that classifies words into predetermined categories with semantic meaning associated with each one.

#### Data analysis

The LIWC-22 CATA programme has over 100 in-built dictionaries consisting of categories of words, word stems and verbal constructions that have been identified to reflect psychological constructs. Reliability and validity of LIWC categories across dozens of psychological domains has been demonstrated within the literature [67]. LIWC analyses text by comparing the content (target words) with dictionary words and categorises them accordingly. The relative frequency of each category is then calculated. LIWC also has four summary variables, which give a percentile score based on comparison of the text with large standardised samples derived in previously published findings [68]. These are analytical thinking [69], clout [70], authenticity [71] and emotional tone [72]. The LIWC categories analysed in this study along with explanation of the psychological construct they reflect are presented in Table 3.

**Table 3:** Categories from the software programme Linguistic Inquiry and Word Count (and an explanation of the construct they represent) that were analysed in a netnographic study which explored veterinary practitioners’ responses to adverse events.

Category group	Category	Explanation
Summary variables	Analytical thinking	Scored from 0 to 100 using percentiles. High score reflects logical style, formal thinking. Low score reflects narrative style, personal thinking.
	Clout	Scored from 0 to 100 using percentiles. High score reflects confident, dominant, higher status language Low score reflects tentative, submissive, lower status language
	Authenticity	Scored from 0 to 100 using percentiles. High score reflects honest, personal style Low score reflects guarded, distanced style
	Tone	Scored from 0 to 100 using percentiles. Reflects the difference between the use of positive and negative emotion words. The higher the score the higher the positive emotional tone The lower the score the lower the negative emotion tone Scores below 50 are suggestive of negative emotional tone.
Function	Total function	Total number of function words posted (the, to, and, that, I, we etc) High number reflects negativity compared to positivity
	I	High number reflective of negative, sad or depressive state
	We	High number reflects exclusivity
	You	High number reflect extreme opinion (less likely to go with status quo)
	Conjunctions	High number reflective or complex thinking High numbers reflect sense of duty especially regarding things ought not to do/have done
	Negations	
Psychological process	Cognitive process	Composite variable of insight/causation/discrepancy/tentative/certitude/differentiation) High score reflects: Complex thinking Debate (recognising conflicting goals or alternative views) Rational reflection Logical (rather than emotional) negotiation
	Insight	High number reflect deep understanding
	Causation	High number reflects appraisal
	Discrepancy	High number reflect need to
	Tentative	High numbers reflect uncertainty
	Certitude	High numbers reflect certainty with a degree of boasting or bravado
	Differentiation	High number reflects author distinguishing between two or more things or people
	Emotion	Composite variable made up of total positive and negative emotion words
	Positive	High numbers indicative of positive emotion of author
	Negative	High numbers indicative of negative emotion of author
	Sadness	High numbers indicative of sadness
	Anxiety	High numbers indicative of anxiety
	Anger	High numbers indicative of anger
	Social Behaviour	Composite variable of prosocial, politeness, interpersonal conflict, moralisation and communication
	Prosocial behaviour	High number reflect a want to help or care for others at an interpersonal level
	Politeness	High number reflect adhere to social norms and manners
	Interpersonal conflict	High number reflect conflict between authors or discussion about conflict
	Moralisation	High number reflects judgemental language, making a moral evaluation
	Communication	High number reflects authors’ discussion of communication
Time orientation	Past focus	Indicates author reflections on past events
	Present focus	Indicates author discussing present
	Future focus	Indicates author looking to future

The LIWC output was checked for completeness and errors by JG and MB before being uploaded to SPSS version 29.0 (IBM Corp, 2022). As the data was not normally distributed (Shapiro-Wilk and Kolmogorov-Smirnov p<0.001), a Kruskall-Wallis test was used to compare the variable distributions between the AE, AHC and Euth groups (p<0.05 was considered significant). Pairwise comparison tests were also performed and results were adjusted by applying the Bonferroni correction for multiple comparisons (after correction, p< = 0.05 was considered significant).

#### Ethical considerations in this study

Although guidance for internet mediated research exists [73], there is a lack of agreement surrounding Facebook research [74] which presents researchers with ethical challenge. The ownership and ‘public’ nature of content is widely debated [75] but nothing negates the need for sensitivity regarding traceability of research material, informed consent and potential for non-consensual identity disclosure of contributors. A quantitative content analysis of the qualitative textual data was employed to avoid issues surrounding potential identification of online contributors. Only JG had access to the raw qualitative data which was stored in line with University of Nottingham’s Research Data Management and Secure Data Handling Policies. The effect of published research on group dynamic was another key consideration. To navigate this, informed consent was attained from the leading moderator of the VV Facebook group, whom the study was then conducted in close collaboration with. Ethics approval was granted by the University of Nottingham, School of Veterinary Medicine and Science ethics committee, approval numbers 3400 210628 and 3506 211202.

### Integration of Part 1 and Part 2

A convergent parallel mixed methods design was used in this study [76]. The search terms used in the netnography were deduced as the interview and focus group analyses progressed but data were otherwise collected and analysed independently. The two sets of yielded findings were integrated using a visual joint display [77] of confirmed, discordant and expanded mixed methods findings [78]. Confirmation is when results gleaned from separate studies enhance inferences [79]. Expansion refers to findings that show both similarity and difference around shared themes, facilitating deeper understanding [80]. Discordance is when interpretations of data conflict, pointing to the need for further exploration [81]. A contiguous narrative was developed to allow for a more holistic and comprehensive understanding of veterinary practitioners’ response to stressful adverse events [76].

## Results

### Part one results: A grounded theory approach employing interview and focus groups

Themes constructed during analysis that have already been described included those focussing on veterinary practitioners’ experiences of ethical challenge [29] and experiences of client complaints [30] in relation to adverse events. The focus of the current study is the theme constructed around veterinary practitioners’ specific response to stressful adverse events. An overview of the theme is shown is Table 4 and is detailed below with subthemes, focussed codes and exemplar quotes from which they were derived.

**Table 4:** Overview of subthemes and focussed codes from which the theme ‘responding following adverse events’ was derived in a focus group and interview study that explored veterinary practitioners’ experiences of adverse events.

Theme	Subthemes	Focussed codes
Responding following adverse events	Experiencing stress	Feeling emotionally and cognitively overwhelmed
		Internalising the experience
		Making negative judgements
	Externalising the experience	Telling the story
		Sitting with the emotion
		Connecting with those who understand
	Morally contextualising the event	Uncoupling feelings from fact
		Accepting uncertainty and complexity
		Forgiving the imperfect ‘good’ practitioner
		Being honest and sharing ownership of outcomes
		Disclosing and apologising
	Catalysing personal and professional improvements	Technically improving
		Creating distance between self and the event
		Reassuring, supporting and teaching others

### Subtheme I: Experiencing stress

#### Feeling emotionally and cognitively overwhelmed

The emotional impact of adverse veterinary events was high, with a lack of emotional control in the immediate aftermath being commonly described. Participants reflected on out of character displays of sadness they had exhibited as a result.

“*I absolutely went to pieces [………] crying all the time it was the first time in ten years that they [colleagues] saw me cry and I said “I just can’t keep going in like this” it was awful I was so so so upset…”*

*(Interview 13)*

Disbelief and associations with symptoms likened to shock arose, with some alluding to a perceived helplessness and accompanying self-mercy. Many berated themselves for a lack of cohesive thought and action in the immediate emotional turmoil of the event.

“*when I got the phone call to tell me it had happened, I sat on the floor of my bedroom and just cried my eyes out [crying] …..mostly because I was just like oh my god…how the hell have I made such a terrible mistake. It was such a shock that it had happened to me”*

(Interview 19)

“*It was just horrendous for me to see the horse go through what it went through and it threw me and I was mad at myself for that too”*

*(Interview 16)*

Practitioners struggled to detail clinical particulars and timeframes in relation to adverse event occurrence, as if temporarily blinded by panic and running on autopilot in the immediate aftermath.

*“…the situation was dire. You know, going out with his nostrils and yeah, shocking, awful and…. I can’t exactly remember the trail of events from there on but the time had just gone by”*

*(Interview 16)*

Despite this, many had distinct clarity on feelings and physiological responses they recalled experiencing in that moment.

“*…that panic. my heart was beating in my ears and I was like dizzy, I guess I was probably holding my breath or something and sweating and then feeling really cold when I got in the car. I shouldn’t have driven really…”*

*(Interview 20)*

#### Internalising the experience

Practitioners spoke of metaphorically internalising stressful adverse veterinary events experiences. This was in contrast to adverse veterinary events that were not deemed stressful, which were mentally compartmentalised, viewed as external to self and did not have high emotional and cognitive impacts. Internalisation manifested as initial reluctance to speak openly about factors that may have contributed. Some had barely spoken of their experience at all.

“*….I just couldn’t properly [talk] about that one [adverse event]. I found it hard to be honest….like I, I actually really really messed up…yeah…I didn’t want it, you know, out loud like that”*

(Interview 18)

“*I think this is about the third time I’ve ever spoken of it…”*

(*Interview 16*)

The acute impact of adverse events clearly had potential to create a lasting imprint on practitioners and to even influence perceptions and formation of personal and professional identity.

“*yeah…..those ones [stressful adverse veterinary events] are different I think because you can’t just shut it down like you can with others and think it’s just something that happened and it’s not who I am. With that time when it happened [stressful adverse veterinary event] I couldn’t get it out of my head and I still think…. you know…. it’s part of me still now”*

*(Interview 20)*

#### Making negative judgements

Practitioners equated the severity of the emotional and cognitive impact and the likelihood of internalising the event with the perceived severity of the adverse event outcome.

“*they’re not all the same, are they… . totally different if something dies for example”*

*(Interview 7)*

The magnitude and duration of guilt and regret experienced were discussed in relation to judgement of clinical decision-making processes in addition to outcomes. Some participants spoke of the role a colleague had played in the event, suggesting that there may be a tendency to retrospectively expect perfection of others as well as oneself.

“*I felt so so guilty for what had happened because it was the way we’d gone about it and the decisions we’d made not just what ended up happening because [name of colleague] thought it was unsafe….maybe I don’t know maybe if I’d done something else….”*

*(Interview 16)*

In addition, practitioners’ negative judgments of self were an influencing factor, with them feeling weighted by a shame proportionate to their unresolved perception of personal failure.

“*…and also it felt a bit like, and I don’t think this was the case but it’s part of the reason I found it very difficult to talk about it for a long long time, I still do because I felt like it was just such a stupid terrible mistake […..talking about the event] it still feels like that if I really think about it which you know is pretty bad seven years down the line…I was good about letting stuff go when it was something I thought reasonable but when it was a mistake that I felt was entirely a fault of what I’d done it was different. I was flawed and I knew it”*

*(Interview 19)*

### Subtheme II: Externalising facts and feelings

#### Telling the story

Throughout the focus groups and interviews, participants consistently went through a process of describing series of events leading to adverse outcomes. Although stimulated by questioning, it was clear that such storytelling was an integral part of helping individuals to process events.

“*getting it out there out loud for real…not just in my head. I didn’t want to it was awful I mean embarrassing, mortifying, awful but I couldn’t think straight about it until I shared it”*

(*Interview 20*)

Participants spoke of sharing details of the adverse event informally to friends, family and colleagues. For some, it had helped to tell the series of events repeatedly to the same or different people as it allowed different details to be remembered and unpicked.

“*I would kind of just talk to my husband a lot about it and talk to others who were vets, it was definitely nice to talk to other people about it”*

(Interview 1)

“*it’s helpful to talk things through*. *Sometimes you’re so channelled…*.*you can’t see the wood for the trees*! *A new set of eyes is always good”*

*(Focus Group 7)*

In addition to talking, the helpfulness of writing down series of events was discussed. While some participants found solace in lone channels such as journaling and diary keeping, others liked and even preferred contributing to textual media where others could read and comment on their story.

“*I kept a diary since I was a kid….I guess writing about it kept me sane”*

(Interview 18)

“*I put a post on there [Veterinary Voices Facebook Group]…*.*to help me reason it…*.*”*

*(Interview 20)*

Some participants described being considered and logical in their written descriptions of events and perceived chronology of facts as important, whilst others described being more abstract and emotionally focussed in their written description. Physical distancing and real or perceived anonymity coupled with lack of real-time dialogue and ability to consider, plan and control when, what and how details were relayed were alluded comforts of those participating in written channels. Social media and specifically veterinary members only Facebook groups were mentioned as well as more formal blogs.

“*people who you work with are too close sometimes…and busy…they know about it already and you don’t want to keep on…I need to vent… I find if y’ not involved yourself and….well don’t know me its easier for me to do…”*

(Interview 10)

“*I think it [social media] can be good to just share what happened to a group of vets without having to think much about anybody knowing you like at the practice…”*

*(Interview 20)*

The benefit of longer-term informal verbal rhetoric was acknowledged. Adverse event involvement was perceived as illustrious in some cases, with participants admitting to sensationalising and even humourising events and associated emotional reactions.

“*….getting to the point where I think ‘have I told this person this story before’… embellishing all the time (laughing) ….dinner table stuff…..it becomes part of your career tapestry”*

*(Interview 17)*

#### Sitting with the emotion

Practitioners emphasised the need to be ‘heard’ emotionally when processing involvement in adverse events. Attempts by the involved practitioner(s), or others, to down-play the magnitude of the adverse event and associated were common. Although well intentioned, such tendency was perceived as unhelpful for many. Recognition and acceptance of differing emotional responses was more valuable.

“*people always say it could have happened to anybody and anyone would feel the same….with hindsight they are right but it doesn’t feel like it at the time and I’m not sure how helpful it is. I told […..person at work….] about it and […..] said come on it’s ok there’s no need to feel like that but the best thing was when […different person at work…] said nothing…..they just made me a cuppa and we just sat there. I talked……”*

*(Interview 20)*

A few participants received professional counselling following involvement in an adverse event and described the benefit of specific emotional support from an individual with no veterinary clinical experience. They felt that the protected time and headspace that counselling allowed was important.

“*I had some counselling to help because I just was very anxious about going back to work and anything that came up about it….I just broke down in tears. I just felt guilty. I had the counselling sessions which really helped for me to deal and manage the situation and to be able to continue it was important the emotional space. I couldn’t do that when I was at work and thinking about how to deal with work and cases…”*

*(Interview 2)*

The sentiment was echoed by those who had received emotional support from friends and loved ones. The ear of individuals not connected to the event, workplace or profession was helpful for some, as it was described to allow embracement of the personal impacts of the adverse event rather than a focus on facts and solutions to clinical issues.

“*I was living with my mum at the time so I was at home with mum and she was amazing but also I had support from [a member of an external union—discussing specifics of the case] …I think it would have been different without her and I think I would have felt very different….she was there for me not for anything else and I was so upset that I needed to feel that…”*

*(Interview 16)*

#### Connecting with those who understand

The importance of feeling understood was a dominant theme. Practitioners made many references to the reassurance provided by working with experienced colleagues and concerns about regarding social isolation if not available.

“*I say…oh…I’ve got this. This happened. I’m lucky, it’s two senior type vets so we can easily bounce off each other….”*

Facilitator: “And if you didn’t have that?

“*It would be very isolating”*

*(Focus Group 7)*

Feeling connected and understood was not always discussed in terms of verbal or written dialogue. Subtle social and interprofessional interactions were also highlighted. Frequent examples of camaraderie and collegiality in the aftermath of adverse events were given.

*“sometimes it’s just knowing that they get it. that smile in the corridor……that message ‘how’s that case? how are things going?”*

(Focus Group 8)

“*she put a note up on the Friday about going to this thing at the pub this thing for everyone and we knew why she was doing it—to get us together after that awful day*. *I talked about it a bit with a nurse who I’m good friends with on the way then we didn’t when we were there*, *we just got on with the night and it was a great thing…*.*”*

*(Focus Group 7)*

The power of sharing details of adverse involvement with others with similar experiences was frequently discussed. Having judged oneself to have the ‘worst’ adverse event experience and the subsequent relief of acknowledging this shared feeling with others was commonly recounted.

“*we got into a joke argument about who had messed up the most! I was crying but then laughing and then crying (laughs) I guess neither were worse and seeing it helped me feel better a bit and then in the long run”*

*(Interview 20)*

Both formal mentorship arrangements and informal working relationships were viewed as beneficial. Beyond that, practitioners found comfort in the availability of access to both local and remote technical advice and support from those with experience of clinical context and adverse event management.

“*there’s usually always somebody. So, if your vet’s [mentor] on holiday, your direct line, but you then can sort of go to one of the others or even phone or message or even like the Referral Centre to say….you know… we’ve got a big Referral Centre just down the road that is our sister company and you can always phone someone there. I think for some of us who’ve worked for the company for quite a while, there’s quite a few familiar faces over there. They’re very approachable, it’s comforting…..”*

(Focus Group 8)

“*…*.*that chitter chatter between vets is a good thing*..*”*

“*yeah*, *rubbing shoulders […*..*] it stops that clamming up when you feel under attack”*

*(Focus Group 7)*

Interview participants recruited via a VDS gatekeeper, frequently referenced the benefit of speaking with claims advisors and/or consultants who had extensive experience of adverse events management. Others experienced invaluable support from colleagues who they alluded as being in a position to empathise.

“*But then again, always very helpful to be able to talk it through with VDS, from their viewpoint….and certainly [name of consultant] having been in the role for a while and knowing how it [aftermath of adverse event] goes”*

(Interview 3)

“*Perhaps I needed that ‘its ok these things happen [from VDS] and ‘we’ll try and guide you through it and it was the informal day to day contact at the clinic too”*.

(Interview 4)

“*…my experienced senior colleague who had left six months previously was honestly the only person that was actually capable of really giving me some really good support because she had done the same sort of work as me and knew how devastating this was and I think if it hadn’t been for her I really think I really really don’t know how I would have coped with it”*

*(Interview 19)*

### Subtheme III: Morally contextualising the event

#### Uncoupling feeling from facts

A pragmatic approach to self-managing the emotional aspects of adverse event involvement was taken by many. Purposefully distinguishing facts surrounding the event and associated personal feelings was experienced as helpful.

“*As soon as I realised what was actually happening and kind of knowing my emotional side of things, I kind of well decided that for my next meeting [to discuss the adverse event], I’m going to completely emotionally detach myself from what I’m actually feeling and I just decided, I’m just going to have this meeting, like I’m an advocate or lawyer for for for this person describing everything with complete neutrality and objectivity. And that helped massively with the emotional side of things”*

*(Interview 10)*

#### Accepting uncertainty and complexity

The complexity of veterinary medical care provision was retrospectively acknowledged, with practitioners finding comfort in considering the overall context of poor clinical outcomes. For some, adverse events highlighted broader welfare perspectives and led to reflection and reaffirmation of professional values.

“*the calf died but the cow was suffering………if I hadn’t tried to calve it who knows? it could have been worse…..[shrugs]? it feels reassuring to me to think like that……”*

(Focus Group 2)

“*I think it’s difficult because I think a lot of the time you might look back on cases that have gone wrong and think we could have done that differently but it doesn’t mean you necessarily made a mistake but with hindsight you could have done a few things differently and it might have given a better outcome but it doesn’t mean it was given something wrong or I don’t know if you mean stuff like that or like proper mistakes when things have gone wrong*. *Things aren’t black and white and seeing it as a something that couldn’t necessarily be predicted by me or by anything helps me to feel better about it as long as I didn’t lie or do something unprofessional or anything…*.*”*

*(Focus Group 1)*

#### Forgiving the imperfect ‘good’ practitioner

Many practitioners battled with the tension created by perfectionist traits within an uncertain clinical environment. Acceptance that adverse events may occur despite best efforts, and relieving oneself of sole ultimate responsibility for preventing adverse events, was a recognised mechanism of coping in the aftermath. Although most actively struggled with doing so for fear of being viewed as lacking conscientiousness, a strong sense that self-forgiveness was necessary for both personal emotional healing was evident. Forgiving other practitioners was much easier.

‘*in spite of me trying and trying…my choices and what I know…I won’t always get it right, I know that now….well I knew it then but I kinda didn’t tell myself it properly but now I believe it’*

(Interview 13)

“*you’ve got to give yourself a break but it’s hard remembering it but then telling yourself you are ok and move on at the same time I mean if it’s someone else you can be easier and it’s like you would always say to them what you should say to yourself about that*.*…you can’t go back*, *you can learn and that’s what makes you good*. *no one is perfect and the best vets are the people who have loads of mistake stories in their locker*!*”*

*(Focus Group 7)*

#### Being honest and sharing ownership of the outcome

Most practitioners spoke of a desire to be open about personal factors, actions and decisions that had or that they perceived to have contributed to the adverse event. They also discussed a concurrent benefit of colleagues being open about their own errors and mistakes.

*Participant 1:* “*yeah I think so, we had one person made some bad mistakes and it was never really looked into and I think they stopped and didn’t realise how serious it was because it was never really addressed and it should have been really”*

*Participant 2*: “*…as a new grad you want people to pull you up and I always like ask and double check and I’d rather someone wasn’t like ‘ah that’ll be fine’ I’d rather ‘well actually you could do this a bit better*, *that a bit better’ cos I think I like to change the way I do things cos if you’re not aware of a reason to change then why would you*. *if it’s gone wrong it helps to just deal with it rather than mulling it”*

*(Focus Group 1)*

Participants reflected on the personal angst of being prevented from ‘owning’ a mistake and the role this may play in impeding individual learning and improvement efforts.

*Participant 1:* “*I’ve had mistakes in the past and then it’s just been like brushed under the carpet and no-one’s saying anything….. it’s like just been hushed away and I felt worse….”*

*Participant 2*: “*yeah and then those people don’t get to manage it and think about what they should do next time*. *Over protecting people doesn’t always help in the long run”*

*(Focus Group 2)*

Acceptance and desire to take collective responsibility for animal welfare at a professional level was also highlighted.

*“On the flip side of this I do feel the need to be professionally accountable…*.….*.a puppy that was here [referral] with dressing issues and it was unsure whether it was relatable to us…but the puppy ended up with a leg amputated as a result….the responsibility should still be with us in part at least anyway and it helps when we do that as a team…..*”

*(Focus Group 3)*

#### Disclosing and apologising

The will of practitioners to be open and honest about adverse events was universal throughout the study. Although many feared reputational damages at a personal, professional and organisational level, they deemed disclosure to clients’ necessary to their emotional recovery.

*“it was the thing that I’d been dishonest with them [client] and so the therapy helped me with that and the guilt and kind of the trying to put myself in the client’s shoes and to understand where they were coming from…..just being able to be honest about it helped”*

*(Interview 1)*

Well-meaning attempts by peers and leadership to protect individuals involved in adverse events from the stress that may be caused by communication with affected clients were appreciated but ironically highlighted as a potential contributor to personal distress.

“*well, it was, it all felt a bit bizarre and a bit surreal to be honest. I never really found it really hard to speak to owners and I guess I didn’t get the opportunity and when I do it’s I guess better to do it myself and just be open its much better”*

*(Interview 13)*

## Subtheme IV: Catalysing personal and professional improvements

### Technically improving

For many, experiencing stressful adverse veterinary events stimulated professional development. Formal recognition of knowledge and skills acquisition through educational achievements appeared to underpin and validate individuals’ redress of professional worth and confidence.

“*The thing that helped me most was doing my certificate. And I think I felt….I felt incompetent and unconfident until I did my certificate. And you know, no matter how dedicated I was to try and do my best. There was just so much I didn’t know. I really wasn’t competent? [………..] Yeah, the certificate helped me enormously to know I was competent or at least when I was doing something new, I knew that I was doing things like I’ve learned to do them and I wasn’t just having to sort of have a go, not really sure what I was doing. And then my confidence grew”*

*(Interview 16)*

Affecting systems change to prevent future adverse event occurrence was a positive contribution made by some.

“…*nothing was actually necessarily done wrong but we can put things in place to improve things in the future”*

*(Focus Group 3)*

#### Creating distance between self and the event

A mechanism used to self-protect and move on following stressful adverse veterinary events was that of physical and emotionally distancing from individuals and locations associated with the event. Actual or planned relocation to a practice in a difference geographic location, and even a different country, was discussed. Practitioners felt this allowed then to wipe clean the slate and start over without the burden of real or perceived judgement. Even in cases where geographic changes were not that great, removing oneself from a practice where the event had taken place was helpful for some.

“*Completely removing myself from being associated with where I was worried people were going to hear and judge one mistake and actually being somewhere far enough away. To have a fresh start. Yeah, helped me. Yeah so that that physical different distance, Umm, probably helped with the emotional distancing from it. Yeah yeah, that’s just knowing that no one’s gonna hear anything about anything”*

(Interview 16)

“*I actually felt like I’d more or less given in–the resigning from my previous job was something that I was reluctant to do*. *I hate giving up on things I hate feeling like I’d failed but leaving that job was a very big factor improving…”*

*(Interview 4)*

#### Reassuring, supporting and teaching others

Over time, practitioners felt empowered to use their negative experiences as a platform to help others suffering due to similar experiences. Beyond helping colleagues, this provided a welcomed way for practitioners to continue to process the complexity of thoughts and feelings surrounding their involvement in adverse events.

“*it’s much more easy to support someone when you’ve been there before….and also when you’re not involved in it emotionally but understand what they’re going through….I mean it’s different for everyone I know but I think it’s still stressful even if you’re someone who copes well. If they’re finding it rough and you have a case you know you found rough you feel in the pit of your stomach…..[talking about a case they were involved in] …but to be honest I like being able to pass that on…that reassurance that you’ve been there…I think it helps me to normalise how I felt as well……”*

*(Interview 7)*

Mentorship and the benefits of positive workplace relations were deemed particularly essential in navigating technical improvements within others, not just in the aftermath but in the prevention of adverse events.

“*if you were working alongside them you might have a relationship where you could say ‘actually that’s, you know, there’s a better way to do this–you should be taking it off for these…’ you could hopefully approach it in a friendship kind of way or a colleague perspective and say ‘there’ a better way to do this…..”*

*(Focus Group 9)*

### Part two results: A netnographic approach utilising natural language processing content analysis of Facebook posts

Facebook posts containing AE discussions were identified from 570 practitioner members, AHC discussions from 416 members and Euth discussions from 469 members. Results of summary variable scores and comparative relative frequencies of analysed word categories for the three groups, along with Kruskal-Wallis and pairwise comparison tests are presented in Table 5A–5D.

**Table 5:** a. Mean and median scores for the four LIWC composite summary variables in the study (AE) and two comparison (AHC and Euthanasia) groups and results from Kruskall Wallis and pairwise comparisons tests. b. Mean and median frequencies for the total number of function words, I, we, you and conjunctions and negations in the study (AE) and two comparison (AHC and Euthanasia) groups and results from Kruskall Wallis and pairwise comparisons tests. c. Mean and median frequencies for psychological process words in the study (AE) and two comparison (AHC and Euthanasia) groups and results from Kruskall Wallis and pairwise comparisons tests. d. Mean and median frequencies for time orientation words in the study (AE) and two comparison (AHC and Euthanasia) groups and results from Kruskall Wallis and pairwise comparisons tests.

	Adverse Events (AE = 573)		Animal Health Certificate (AHC = 419)		Euthanasia (Euth = 472)		P value	Pairwise comparison (*^)
	Mean	Median (IQR)	Mean	Median (IQR)	Mean	Median (IQR)		
Analytic	59.97	40.91 (7.99–48.90)	48.22	62.1 (16.22–78.33)	36.99	38.33 (8.89–62.75)	<0.001	AE-AHC AHC-Euth
Clout	62.71	55.88 (40.06–95.94)	37.19	62.2 (5.21–67.40)	47.11	68.74 (14.49–83.23)	<0.001	EP
Authenticity	32.17	56.15 (3.45–59.59)	52.85	70.94 (16.64–87.58)	49.53	63.32 (18.26–81.58)	<0.00	AE-AHC AE-Euth
Tone	38.09	73.72 (3.56–77.28)	36.18	33.34 (20.23–53.56)	38.34	56.74 (11.2–67.94)	0.07	-
	Adverse Events (AE = 573)								Animal Health Certificate (AHC = 419)							Euthanasia (Euth = 472)							P value	Pairwise comparison (*^)
	Mean	Median (IQR)	Mean	Median (IQR)	Mean	Median (IQR)																		
Total Function	60.60	8.66 (56.975–65.63)	57.45	<0.001	58.70	9.20 (54.44–63.64)	<0.001	EP
i	2.69	4.28 (0.00–4.28)	3.71	<0.001	3.64	5.45 (00.00–5.45)	<0.001	AE-AHC AE-Euth
we	0.93	0.73 (0–0.73)	0.91	<0.001	1.35	1.82 (00.00–1.82)	<0.001	AE-Euth AHC-Euth
you	4.60	7.41 (00.00–7.41)	1.68	<0.001	2.23	2.86 (0.00–2.86)	<0.001	EP
conjunctions	6.86	4.59 (4.63–9.21)	7.22	0.548	6.86	4.39 (4.76–9.15)	0.548	-
negations	2.19	3.23 (0.00–3.23)	2.52	0.122	2.32	3.33 (0.00–3.33)	0.122	-
	Adverse Events (AE = 573)		Animal Health Certificate (AHC = 419)		Euthanasia (Euth = 472)		P value	Pairwise comparison ( * ^ )
	Mean	Median (IQR)	Mean	Median (IQR)	Mean	Median (IQR)		
Cognitive processes	13.92	7.93 (9.72–17.65)	13.05	9.06 (8.33–17.39)	14.58	7.65 (10.53–18.18)	<0.001	AE-AHC AHC-Euth
Insight	2.47	3.70 (0.3.7)	2.74	4.35 (0.00–4.35)	3.19	4.35 (0–4.35)	0.005	AE-Euth AHC-Euth
Cause	1.29	2.08 (0–2.08)	1.03	1.27 (0.00–1.27)	1.55	1.55 (0.2.04)	<0.001	AE-AHC AHC-Euth
Discrepancy	2.79	4.16 (0.00–4.16)	2.60	4.17 (0–4.17)	2.68	3.57 (0.00–3.57)	0.095	-
Tentative	2.94	4.62 (0–4.62)	2.88	5.00 (0.00–5.00)	2.99	4.44 (0.00–4.44)	0.342	-
Certitude	1.51	2.33 (0.00–2.33)	0.63	0.00 (0.00–0.00)	0.99	1.21 (0.00–1.21)	<0.001	EP
Differentiation	4.21	4.61 (1.31–5.92)	4.78	7.05 (0.00–7.05)	4.44	4.78 (1.56–6.34)	0.427	-
Emotion	2.99	4.17 (0.00–4.17)	0.73	0.00 (0.00–0.00)	2.65	3.75 (0.00–3.75)	<0.001	AE-AHC AHC-Euth
Positive emotion	1.25	1.61 (0.00–1.605)	0.46	0.00 (0.00–0.00)	0.89	1.23 (0.00–1.23)	<0.001	
Negative emotion	1.56	2.15 (0.00–2.15)	0.24	0.00 (0.00–0.24)	1.52	2.08 (0.00–2.08)	<0.001	
Anxiety	0.36	0.00 (0.00–0.00)	0.07	0.07 (0.00–0.00)	0.20	0.02 (0.00–0.00)	<0.001	
Anger	0.25	0.25 (0.00–0.00)	0.02	0.02 (0.00–0.00)	0.15	0.15 (0.00–0.00)	<0.001	
Sadness	0.21	0.21 (0.00–0.00	0.01	0.01 (0.00–0.00)	0.55	0.55 (0.00–0.00)	<0.001	EP
Social behaviour	6.31	5.10 (3.23–8.23)	3.16	5.18 (0.00–5.18)	5.20	4.24 (2.38–6.62)	<0.001	EP
Prosocial behaviour	1.81	2.62 (0.00–2.62)	0.63	0.00 (0.00–0.00)	1.54	2.27 (0.00–2.27)	<0.001	AE-AHC AHC-Euth
Politeness	0.29	0.29 (0.00–0.00)	0.31	0.31 (0.00–0.00)	0.27	0.27 (0.00–0.00)	0.819	-
Interpersonal conflict	0.83	1.34 (0.00–1.34)	0.06	0.00 (0.00–0.00)	0.32	0.00 (0.00–0.00)	<0.001	EP
Moralisation	0.84	1.16 (0.00–1.16)	0.17	0.00 (0.00–0.00)	0.67	0.00 (0.00–0.00)	<0.001	EP
Communication	2.22	3.33 (0.00–3.33)	2.02	3.45 (0.00–3.45)	1.74	2.63 (0.00–2.63)	0.010	-
	Adverse Events (AE = 573)									Animal Health Certificate (AHC = 419)						Euthanasia (Euth = 472)						P value	Pairwise comparison ( * ^ )
	Mean	Median(IQR)					Mean	Median(IQR)	Mean	Median(IQR)													
past focus	5.09	6.32 (1.38–7.69)					4.47	6.69 (0.00–6.69)	4.16	6.45 (0.00–6.45)	0.001	AE-AHC AE-Euth
present focus	5.41	5.38 (2.31–7.69)					5.28	7.69 (0.7.69)	6.25	5.54 (2.86–8.40)	0.005	AHC-Euth
future focus	1.98	3.02 (0.00–3.02)					1.29	1.39 (0.00–1.39)	1.18	1.64 (0.00–1.64)	<0.001	AE-AHC AE-Euth

*Significance level is < = 0.05.

^values are adjusted by the Bonferroni correction for multiple tests. E-P = Each pairing of groups was significantly different, AE-AHC = adverse event and animal health certification groups were significantly different, AE-Euth = adverse event and euthanasia groups were significantly different, AHC-Euth = animal health certification and euthanasia groups were significantly different.

There was a lack of statistical difference between the composite emotional tone percentile score of AE, AHC and Euth groups, with the score across the groups <50 indicating a negative emotional state amongst practitioners in relation to all three topics (Table 5A). The frequencies of overall emotion and specifically positive, negative, anxiety and anger words were not significantly different between the AE and Euth groups suggesting that the emotional challenge experienced by practitioners in relation to adverse events is akin to that experienced in relation to euthanasia (Table 5C). However, significant differences in total function (Table 5B) and sadness words (Table 5C) in the AE group compared to the AHC and Euth groups suggests that adverse events may lead to unique emotional experiences.

There was a lack of significant difference in analytic thinking scores between AE and Euth groups and a significantly different analytical thinking score in the AHC group compared to both AE and Euth groups (Table 5A). This suggests that veterinary practitioners use similar thinking styles in relation to adverse events and euthanasia, which is different from that used in relation to animal health certification. A lack of significant difference in the frequency of cognitive processing words in the AE and Euth groups, but a significant difference in frequency of cognitive processing words in the AHC compared to both AE and Euth groups was also observed (Table 5C). This suggests that practitioners recognise conflicting goals and reflect and use degrees of complex thinking similarly in relation to responding to adverse events and euthanasia, which is different to when they respond in relation to animal health certification.

The mean and median authenticity score in the AE group was significantly lower than in the AHC and Euth groups suggesting that practitioners are guarded and less likely to be honest in relation to adverse events (Table 5A). The frequency of moralisation words in the AE group compared to the Euth and AHC groups was significantly higher suggesting that practitioners may be more likely to evaluate adverse event occurrence in terms of right and wrong doing (Table 5C). No significant difference in frequency of conjunctions and negations was observed across the three groups, suggesting that veterinary practitioners’ sense of duty regarding what ought to happen, or have happened, in adverse events is consistent with that of AHC and euthanasia (Table 5B).

There was a significant difference in frequencies of past and future focus in the AE group compared to both AHC and Euth groups (Table 5D). The AE group had a higher mean and median number of future focus words suggesting that practitioners are likely to consider future implications of adverse events more than they would euthanasia or animal health certification. Interpretations regarding practitioners’ use of hindsight in relation to adverse events presents challenge. The mean number of past focused words were higher in the AE group compared to the AHC and Euth group, yet the median frequency of past focused words is slightly lower in the adverse event group compared to both Euth and AHC groups. Interpretation of mean values is favoured in linguistic analysis [82,83], but median values are more sensitive to every data point within the analysis and may better reflect the true central tendency of the non-parametric data collected from the VV UK community under study.

Significantly different clout scores across the groups, coupled with significantly increased frequency of ‘you’ in the AE group compared to both AHC and Euth groups suggests that practitioners may be more dominant, confident and less likely to go with the status quo with their opinions about adverse events compared to AHC or euthanasia (Table 5A and 5B). A significantly higher mean and median frequency of certitude words in the AE compared to both AHC and Euth groups additionally suggests bravado amongst practitioners when discussing adverse events (Table 5C). A significantly higher frequency of interpersonal conflict words in the AE group compared to either the AHC or Euth groups suggests that practitioners experience more conflict with others in relation to adverse events (Table 5C).

A significantly higher frequency of prosocial words within the AE and Euth groups compared to the AHC group may indicate a desire amongst practitioners to help or care for others at an interpersonal level in relation to adverse events in a similar way they would to euthanasia (Table 5C). A significantly lower frequency of ‘we’ in the AE compared to the Euth group suggests a lack of exclusivity and sense of group cohesion when discussing adverse events (Table 5B).

### Integration of Part 1 and Part 2 results

A visual joint display of findings from part one and part two, including assessment of confirmation, expansion and discordance is presented in Table 6.

**Table 6:** Joint display illustrating the assessment of confirmatory, expansive and discordant findings of Part 1 and Part 2 analyses to understand veterinary practitioners’ response following adverse events.

	Results from Part 1: Themes derived from grounded theory informed analysis of interview and focus group data	Results from Part 2: Natural language processing content analysis of Facebook posts where veterinary practitioners discuss adverse events (AE), compared to posts where veterinary practitioners discuss Animal Health Certification (AHC) and Euthanasia (Euth)
**Confirmation** Findings in study 1 and 2 align. Integration enhances inferences made in each study.	Experiencing stressful adverse veterinary events.	Lack of statistical difference in frequency of total, positive and negative emotion, anger and anxiety between the AE and Euth groups. Significant difference in the frequency of sadness words across the groups.
	Morally contextualising the event.	Significantly higher use of moralisation and past orientated words in the AE group compared to both AHC and Euth groups. Significantly higher frequency of causation words in the AE group compared to the AHC group but a lack of significant difference in the frequency of causation words between the AE and Euth group.
	Catalysing personal and professional improvements.	Significantly higher use of prosocial words in the AE group compared to the AHC group but no significant difference was observed between the AE and Euth groups. Significantly higher use of future focussed words in the AE compared to the AHC and Euth groups.
**Expansion** Findings in study 1 and 2 show similarities and differences around a shared theme. Integration facilitates further understanding.	Experiencing stressful adverse veterinary events.	Low emotional tone in AE, AHC and Euth groups (score <50 across all groups).
	Externalising facts and feelings.	Significantly lower use of ‘we’ in the AE compared to the Euth group.
	Catalysing personal and professional improvements.	Significantly higher analytical thinking score in the AE compared to the AHC but a significance difference between the AE and Euth group was not observed.
**Discordance** Findings in study 1 and 2 conflict. Integration highlights need for further research.	Morally contextualising the event.	Significantly lower authenticity score in AE group compared to both AHC or Euth groups. Significantly higher frequency of certitude words in the AE compared to both AHC and Euth groups. Lack of statistical difference in frequency of conjunctions and negations across the groups.
	Externalising facts and feelings	Significantly higher frequency of interpersonal conflict words observed in the AE compared to the Euth and AHC group.

#### Confirmatory findings: Veterinary practitioners’ responses to adverse events involve emotional reactions, moral reflection and supporting self and others

Focus group and interview findings give insight into the often-overwhelming, shameful emotions veterinary practitioners experience in relation to adverse events; NLP content analysis reveals significant differences in frequencies of words indicative of angry, anxious and negative emotions in the AE group compared to the AHC but a lack of significant difference between the AE and Euth groups. In addition, the frequency of sadness words was significantly different across the groups, suggesting that involvement in adverse events leads to a partly unique emotional response. Focus group and interview findings provide evidence that practitioners morally reflect as a result of their experience of adverse events; NLP content analysis reveals significantly higher frequencies of moralisation in the AE group compared to both AHC and Euth groups and a lack of significant difference in causation words between the AE and Euth groups. Focus group and interview findings suggest that practitioners want to support others as a result of adverse events; NLP content analysis reveals significantly higher frequencies of words indicative of consideration of the future in the AE group compared to both AHC and Euth groups. Significantly higher frequencies of words indicative of a desire to help others was observed in the AE group than the AHC but no significant difference between the AE and Euth group was observed. Integration of findings enhances inferences made following part one and part two regarding the emotional, morally reflective and supporting nature of practitioners’ responses to adverse events.

#### Expansive findings: By identifying as part of a community of shared negative experience, veterinary practitioners collectively contextualise what happened in relation to adverse events to redress emotional detriment and to learn as a result of the experience

Focus group and interview analysis suggests that veterinary practitioners are stressed but have a desire to connect with those who understand their experience of adverse events; NLP content analysis reveals low emotional tone coupled with significantly lower mean and median frequencies of ‘we’ in the AE compared to the Euth group, reflecting lack of exclusivity. Integration therefore suggests that practitioners develop a sense of belonging through shared negative experience. Focus group and interview analysis suggests that practitioners want to affect improvements as a result of their experience of adverse events; NLP content analysis reveals a lack of significant difference in frequency of words indicative of logical thinking, acceptance of differing views, debate and positive emotion in the AE group compared to the Euth group. Expansive findings suggest that collaborative moral reflection plays a role in countering practitioners’ negative emotional responses to adverse events.

#### Discordant findings: Veterinary practitioners’ intentions and expressions of honesty and accountability in relation to adverse events conflict

Focus group and interview analysis revealed practitioners’ desire to be honest, to share accountability for and to apologise regarding adverse events; NLP content analysis reveals a significantly lower authenticity score in the AE compared to the Euth and AHC group, indicative of a guarded, distanced and potentially dishonest psychological state. No statistical difference in frequency of conjunctions and negations in the AE group compared to the AHC and Euth group suggesting a lack of difference in sense of duty practitioners experience across the topics. Significantly higher frequency of words indicative of interpersonal conflict was observed in the AE compared to the Euth and AHC group. Discordant findings highlight the need for further research regarding veterinary practitioners’ intentions and expressions of collaborative learning, honesty and accountability.

## Discussion

This study used a novel mixed methods approach to understand veterinary practitioners’ response following adverse events. A grounded theory informed analysis of transcribed data attained during focus groups and interviews with veterinary practitioners about adverse events was performed. One theme constructed focussed on veterinary practitioners’ response to adverse events and was derived from four subthemes; experiencing stress, externalising facts and feelings, morally contextualising the event and catalysing personal and professional improvements. Integrating these findings with a comparative NLP content analysis of text written by veterinary practitioners in Facebook posts about adverse events and two comparison subjects confirmed findings regarding the emotional, morally reflective and catalysing impact of adverse events. Integration also expanded understanding of the complex interplay of personal, cognitive and social factors that facilitate practitioners to collectively redress emotional detriment suffered and to learn and improve as a result of the experience.

A key contribution this study makes is a nuanced understanding of the emotional impact adverse events can have on veterinary practitioners in the UK, which reflects findings of previous studies. Nearly two thirds of 606 Veterinary Information Network (VIN) member respondents surveyed by Kogan et al. (2018) reported an overall reduction in happiness as a direct result of an adverse event and over a third had problems sleeping and, or felt persistently guilty [26]. Resonating with the finding here that practitioners internalise their experiences of adverse events, previous qualitative studies have described how initial compartmentalisation of emotional reactions to adverse events is perceived to protect professional functionality [27]. The feelings of overwhelm, sadness, anxiety and uniqueness identified in this study align with the symptoms of shock, guilt and anxiety described in the early stages of *second victim* responses of human healthcare practitioners [1,13,84–87]. The findings poignantly echo those of a seminal study by Scott et al. (2009) in which ‘chaos and accident’ and ‘intrusive reflections’ stages of response were defined [10], and a study by Luu (2012) where the emotional blow and subsequent cognitive load were characterised as the ‘kick’ and ‘fall’ of human surgeons’ emotional reactions to adverse events [88]. The findings in this study suggest that veterinary practitioners may benefit from signposting to immediate ‘at the time of the adverse event’ support, to prevent them experiencing a prolonged maladaptive emotional state.

The role self-judgement plays in practitioners’ emotional responses to adverse events is highlighted in this study. Factors that predispose veterinary practitioners to making negative self-judgements in relation to adverse events include those associated with imposter syndrome (IS). Described as an internal experience of ‘intellectual phoniness’ [89] IS impels personal feelings of doubt regarding knowledge, skills and accomplishments [90–92]. Established connections between IS and burnout, depression, anxiety and low self-esteem in human healthcare [93–96] alongside suggestion that IS may be most prevalent amongst high achieving females [97] is noteworthy to an increasingly feminised veterinary profession currently suffering a recruitment and retention crisis [98]. As a component of IS, perfectionism is a multifaceted trait concerned with self-centric standard setting and evaluation [99]. It has long been suggested that an entrenched culture of infallibility and perfectionism is present within the medical profession [100]. While adaptive perfectionism may enhance both performance and wellbeing as it is based on intrinsic personal goals [101], maladaptive perfectionism is driven by socially prescribed standards and overly critical self-judgement. Individuals who display maladaptive perfectionism are known to attribute poor outcomes to lack of personal ability [102–104] and have difficulties in coping with real or perceived failure [99,105–109]. Tellingly, ‘concern over mistakes’ is a major dimension in Multidimensional Perfectionism Scales (MPS) that are used to measure the construct [99,107]. The pervasiveness and implications of imposter syndrome and associated maladaptive perfectionism within the veterinary profession has received only a small amount of literary attention [110,111]. It has previously been suggested that perfectionism may enhance practitioners’ vulnerability to morally challenging events [112], but findings here prompt specific exploration into links between IS, maladaptive perfectionism and veterinary practitioners’ responses to adverse events. Findings provoke contentious questions surrounding the identification of veterinary practitioners who are at increased risk of suffering detrimental emotional impacts in the wake of adverse events, whether it is possible to emotionally prepare such individuals and with whom responsibility for this lies.

The combined emotional reactions and moral evaluation surrounding adverse events demonstrated in this study could reasonably convert to rumination, a tendency to experience guilt and anxiety for prolonged periods and to cultivate an already recognised professional culture of ‘blame and shame’ [113]. It is postulated that to recover from the emotional disruption caused by adverse events and to prevent longer term stigmatisation and such cultural sequalae, affected individuals must restructure the narrative surrounding what happened [114]. Honesty and accountability are identified as factors in human healthcare practitioners’ coping ability following adverse events [115,116] and avoidance of repressive behaviour is consistently stressed as key to successful adverse event management [115,117–120].

Narrative-based medicine (NBM) is built on the premise that meaningful interpretations of situations are derived through storytelling [121–123]. Be it through verbal or written medium, NBM encourages honest sharing of experiences and perspectives and has been postulated as an important means of maintaining emotional equilibrium amongst veterinary practitioner who experience guilt and shame as a result of their work [124]. NBM promotes reflective practices such as those facilitated in Schwartz rounds [125] which have recently been explored within a veterinary context [126]. As the findings here underline the potential benefits of collective reflection, they provide impetus for developing an evidence-base to support the implementation of such face-to-face narrative-based reflective methods.

Discordance in findings related to veterinary practitioners’ intentions and expressions of honesty and accountability are worthy of further examination. Such discordance may be down to an awareness amongst practitioners that in posting online about their experiences they are publicly publishing and are therefore less likely to be entirely candid, despite wanting to be. Another hypothesis is that veterinary practitioners’ intentions to be honest and accountable in relation to adverse events in order to overcome emotional detrimental impacts are paradoxically stifled by those emotional impacts. Veterinary professional identity is steeped in beliefs regarding a duty to safeguard the welfare of patients [127]. The psychological discomfort, known as ‘identity dissonance’ [128], experienced as a result of perceived breeches of this when adverse events occur, could naturally invoke a denial response which manifests as a lack of complete honesty. The notion of anticipated regret [129] also provides an explanatory lens here. Anticipated regret is an emotion experienced by individuals when considering interpersonal risk and may cause them to behave in a risk averse or defensive way. Veterinary practitioners’ perceptions of anticipated risk surrounding peer judgement, individual culpability and associated personal and experiences of professional repercussions in the wake of adverse events likely impedes honesty about factors that may have contributed. Bosk’s seminal ethnography, aptly named *‘Forgive and Remember’* [130], tackles morality and accountability in relation to adverse event occurrence in the human medical profession. Deftly suggesting that forgivable and culpable failings are ordinarily distinguished only by socially constructed norms, the work unsettles absolutist assumptions regarding ‘good and bad’ practitioners. Such philosophical musings not only underpin discourse regarding the appropriateness of sanction for practitioners who are involved in adverse events. They acknowledge that keeping patients safe is a ‘problem of many hands’ [131,132] and highlight the essentiality of creating professional cultures that discourage scapegoating to encourage balanced systems focused prevention of adverse events.

To the authors knowledge, this is the first study to provide evidence that by identifying as part of a community of shared negative experience in relation to adverse events, veterinary practitioners contextualise what happened and are able to redress emotional detriment and learn as a result of the experience. Human healthcare practitioners are shown to preferentially seek emotional support from peers than to engage with employee assistance programs in relation to their experiences of adverse events [133]. The need to explore operationalisation of peer-to-peer support (PTPS) for veterinary practitioners who experience stressful adverse veterinary events is supported by findings in this study that they seek to connect with those who understand. Peer-to-peer support (PTPS) refers to support given between individuals with common experiences and is founded on respect, shared responsibility and mutual agreement of what is helpful [134]. It is not based on psychological treatment but on the creation of social networks, where individuals may only be known to each other specifically for the purposes of emotional appraisal and knowledge exchange [135]. Facebook is a platform that facilitates creation of social networks, manifesting as online PTPS (oPTPS). Providing opportunity for individuals to exchange knowledge, thoughts and feelings with those sharing similar experiences, oPTPS has been shown to reduce feelings of social isolation and stigma in other spheres [38,136–142] and may counter enforcement of self-victimisation in relation to traumatising events. Reassuringly convenient access, the ability to readily exchange knowledge [143–152] and feelings [38,138–142] with those sharing similar experiences, coupled with perceived levels of trustworthiness and anonymity make such groups an attractive source of support. The authors suggest that the Facebook group examined in this study already provides a platform for oPTPS in a veterinary context. One observation made in the course of this study was that although some veterinary practitioners voiced a reluctance to verbalise their experiences of adverse events in a face-to-face manner, discussions regarding adverse events on the VV Facebook site were plentiful. Even anonymous posts within closed Facebook groups may expose individuals and organisations at scale, lead to associated reputational damage and risk breeches of professionalism. Despite this,

it would be interesting to explore veterinary practitioners’ perceptions of the usefulness of such groups and appetite for an online platform specifically aimed at providing oPTPS in relation to adverse events, including any concerns they may have regarding potential personal and professional ramifications.

Applications based on artificial intelligence that aim to support individuals through conversation are in use. So called ‘chatbots’ learn to recognise patterns in feelings written by users and to respond with positive support tailored to individuals’ emotional needs. Confirmatory and expansive findings in this study suggest that NLP may be useful in understanding practitioners’ responses to adverse events. Although discordant findings challenge this interpretation, further research exploring practitioner-chatbot interactions in relation to adverse events could expose whether generative NLP has a role to play in supporting those suffering emotional detriment.

### Strengths and limitations

Use of focus groups and interviews enabled in depth understanding of veterinary practitioners’ experiences but are subject to social desirability bias. The convenience and purposeful recruitment methods employed and relatively small numbers of practitioners participating additionally limits generalisability of the findings. The netnographic approach used was unobstructive, subject to less social desirability and recruitment bias and allowed examination of a greater sample of veterinary practitioners. Despite this, it was limited to a veterinary demographic who have access to and engage with a specific veterinary member-only Facebook group. While joint displays facilitate robust integration of findings, such analyses are interpretative and subject to bias imposed by the researchers.

The search terms used in the data collection phase of netnographic NLP part two of the study, were determined *a priori* by the primary researcher (JG). The choice of search terms was influenced by analysis of interview and focus group data in part one of the study, the researcher’s identity and positionality as an experienced veterinary surgeon and collaborations with the lead moderator of the VV UK FB group under study. These factors represent sources of bias; different search terms would likely yield collection of different data sets and may produce very different findings. The netnographic NLP data collected from the three groups in part two of this study were appropriately compared using nonparametric statistical tests in view of the non-gaussian distribution and large size of the data sets.

## Conclusions

This novel mixed method study not only explicates veterinary practitioners’ experiences of stress in relation to adverse events but suggests that by morally contextualising and catalysing learning and improvements as a result, practitioners may avoid pathologisation of their emotional responses in the longer term. By studying an established online veterinary members only social networking Facebook community, the role that SNS facilitated peer-to-peer mediated support may play in practitioners’ responses is specifically highlighted. In shedding light on practitioners’ experiences and naturalistic support behaviours, it is hoped that these findings will be used to stimulate the design and implementation of organisational led peer support for practitioners in relation to their experiences of adverse veterinary events.

The authors acknowledge that the content of this manuscript has the potential to trigger emotional reactions amongst the readership. Support and signposting for members of the veterinary community may be found at https://www.vetlife.org.uk/.

## Supporting information

S1 File Excel spreadsheet containing the minimal data set required to replicate the results that are summarised in Table 5A–5D. (PDF)
