# Supplementary material for: Understanding veterinary practitioners’ responses to adverse events using a combined grounded theory and netnographic natural language processing approach
Source: PLoS One. 2024 Dec 5;19(12):e0314081. doi: 10.1371/journal.pone.0314081 (PMC11620440; doi:10.1371/journal.pone.0314081)
Supplement: S1 File — (PDF) [file pone.0314081.s001.pdf]

Searched term	Author	Analytic	Clout	Authentic	Tone	total functi	i	we
1	x	45.91	57.85	69.28	20.23	62.69	0	0
1	x	12.74	40.06	1	99	57.89	5.26	0
1	x	46.7	24.36	93.5	99	50	2.94	2.94
1	x	25.29	47.97	26.54	20.23	62.67	5.33	4
1	x	51.04	64.04	45.99	1	50	2.7	0
1	x	6.22	90.88	1	92.27	57.89	2.63	0
1	x	20.07	7.5	84.38	8.11	63.16	6.58	0
1	x	81.21	40.06	87.24	58.42	58.54	2.44	0
1	x	6.68	1.58	74.91	20.23	63.64	7.27	0
1	x	4.85	93.13	51.08	33.68	69.23	1.92	0.96
1	x	3.43	40.06	99	20.23	53.13	6.25	3.13
1	x	67.62	99	1	1	63.16	0	5.26
1	x	44.88	60.74	57.28	32.27	60.87	6.96	0
1	x	89.52	40.06	1	1	0	0	0
1	x	1	40.06	15.38	99	50	25	0
1	x	3.37	97.57	98.13	99	66.67	7.41	0
1	x	1	1	89.39	1	66.67	0	0
1	x	1	94.84	1	20.23	50	0	0
1	x	10.19	98.75	39.59	99	66.67	0	0
1	x	52.92	93.87	45.04	20.23	56.88	4.59	0.92
1	x	13.27	11.66	15.38	96.74	56.25	0	6.25
1	x	26.1	99	1	20.23	62.5	0	0
1	x	2.35	97.11	2.36	1	50	0	0
1	x	23.18	49.82	3.62	1	54.1	1.64	1.64
1	x	74.58	99	21.25	1	56.86	0	7.84
1	x	7.78	97.48	4.6	1	64.71	5.88	5.88
1	x	45.65	96.81	1	1	58	0	6
1	x	78.62	47.29	14.11	3.01	54.88	0	0
1	x	4.09	53.95	2.18	87.73	65.12	2.33	0
1	x	84.92	99	12.44	95.42	52.94	0	0
1	x	70.16	71.69	2.05	7.77	53.42	4.11	5.48
1	x	29.72	19.4	31.39	6.3	55.28	6.5	0
1	x	39.7	3.95	63.35	1	65	0	0
1	x	29.85	12.77	90.94	1	64.71	7.84	5.88
1	x	39.19	14.81	90.78	5.65	61.4	4.39	0
1	x	57.76	23.05	43.39	35.47	64.52	5.38	0
1	x	1	40.06	1	20.23	71.43	0	0
1	x	73.36	1	89.39	20.23	63.89	8.33	0
1	x	58.76	17.46	20.79	20.23	59.09	4.55	0
1	x	82.9	14.45	35.73	2.4	62.42	5.37	0
1	x	11.45	96.25	7.72	62.77	65.95	1.62	4.32
1	x	29.57	97.34	5.07	76.62	65.45	0	1.82
1	x	13.53	94.84	25.1	24.31	62.82	0.64	3.21
1	x	24.47	99	1	2.35	62.16	1.35	4.05
1	x	31.07	67.13	41.41	1.63	64.62	1.54	0
1	x	2.35	93.83	12.96	1	67.86	1.19	5.95
1	x	12.09	94.01	3.34	50.98	62	0	2
1	x	47.73	98.04	19.9	49.66	51.92	0	1.92
1	x	37.99	48.8	67.28	66.58	63.24	1.47	0

1 x	5.75	89.2	38.31	1.29	61.54	3.3	4.4
1 x	5.8	96.16	8.2	1	67.16	0	1.49
1 x	49.31	95.78	40.45	20.23	60.58	2.19	2.19
1 x	69.05	77.41	1	72.45	50	0	0
1 x	29.85	95.91	12.44	98.98	63.24	2.94	0
1 x	1.3	99	1.13	54.36	69.23	2.2	6.59
1 x	9.6	98.5	73.75	81.75	64.86	2.7	0
1 x	47.15	20.71	92.99	1	61.25	3.75	0
1 x	23.08	73.96	2.87	11.57	61.86	0	0
1 x	67.99	3.5	76.37	61.07	55.84	1.3	0
1 x	1	1.92	86.68	1	69.7	15.15	3.03
1 x	33.89	50.15	7.47	1.14	54.24	0	0
1 x	27.91	3.95	11.24	1.98	64.29	4.29	0
1 x	1	99	1	1	79.17	0	4.17
1 x	81.89	92.77	1	3.56	50	0	0
1 x	1	71.32	65.19	2.35	63.51	5.41	6.76
1 x	1.97	75.49	3.08	1.56	67.19	1.56	0
1 x	14.87	55.87	55.91	2.48	64.39	6.06	1.14
1 x	6.83	70.12	7.91	1.05	58.62	0	0
1 x	44.1	92.24	27.44	77.5	70.37	0	3.7
1 x	18.87	95.32	1	20.23	53.85	0	0
1 x	43.84	40.06	1	1	65.12	0	0
1 x	24.95	96.99	8	10.8	59.43	4.72	6.6
1 x	24.16	98.45	3.43	6.37	62.9	0	3.23
1 x	40.05	90.15	32.79	8.85	63.86	1.2	2.41
1 x	16.2	92.65	17.84	5.73	64.35	1.74	0
1 x	56.86	99	56.69	20.23	54.76	0	0
1 x	34.86	95.94	32.79	28.26	62.05	2.41	2.41
1 x	15.29	88.96	6.58	20.23	66.01	3.92	1.96
1 x	28.93	93.9	16.2	7.03	66.42	2.99	1.49
1 x	2.08	89.9	34.62	41.62	65.22	0	0
1 x	78.76	95.48	43.66	2.64	62.93	0	6.03
1 x	23.88	98.75	1	93.93	44.44	0	1.85
1 x	6.22	99	1	92.27	63.16	0	5.26
1 x	12.51	62.84	25.1	79.29	58.65	5.77	0
1 x	9.48	1.93	79.1	20.23	62.9	3.23	0
1 x	13.27	97.37	5.64	69.4	61.46	0	1.04
1 x	82.95	94.01	35.01	20.23	68	4	0
1 x	21.94	95.01	19.52	20.23	66.14	1.57	0
1 x	49.68	72.07	86.93	52.41	61.11	2.78	0
1 x	48.44	47.29	75.67	20.23	56.1	1.22	0
1 x	44.79	98.98	6.47	8.75	59.76	0	3.66
1 x	51.48	30.13	4.78	20.23	66.07	1.79	1.79
1 x	34.72	1.4	82.12	1.92	62.32	5.8	0
1 x	6.94	49	33.61	42.52	62.41	7.52	3.01
1 x	65.5	9.71	69.11	5.73	66.09	4.35	0
1 x	8.79	76.91	11.41	1	57.38	0	0
1 x	1	40.06	1	1	63.64	9.09	0
1 x	96.35	97.11	1	98.65	57.14	0	0
1 x	70.71	87.7	1	69.4	53.13	3.13	0

1 x	8.25	87.48	5.5	3.38	69.77	0	4.65
1 x	8.85	1	99	6.65	64.06	12.5	0
1 x	11.57	82.38	12.44	50.31	67.65	0.98	6.86
1 x	52.42	42.95	7.91	1.05	55.17	0.99	0
1 x	49.68	6.61	94.8	4.22	62.5	8.33	0
1 x	77.13	14.33	45.99	20.23	66.22	6.76	0
1 x	79.95	25.32	82.59	1	57.53	8.22	0
1 x	8.79	4.17	92.94	6.23	67.21	8.2	3.28
1 x	57.83	45.56	60.15	3.38	60.47	2.79	1.4
1 x	71.74	95.32	16.81	8.34	64.1	1.28	2.56
1 x	28.19	19.85	69.17	9.61	60	6.37	0.22
1 x	8.37	65.68	17.84	1	60.87	4.35	0
1 x	10.19	72.07	10.18	1	55.56	5.56	0
1 x	19.76	1	89.39	1	69.44	2.78	0
1 x	31.07	69.89	66.8	1	64.1	0	5.13
1 x	1	1	89.39	20.23	50	0	0
1 x	2.75	1.48	1.77	1	54.84	6.45	0
1 x	6.11	18.24	17.84	3.88	65.22	4.35	0
1 x	5.64	99	1	1	56.25	0	0
1 x	1	62.05	27.44	1	62.96	3.7	0
1 x	19.76	10.48	91.13	1	62.22	5.56	0
1 x	43.4	91.85	15.38	1	65.63	0	0
1 x	76.81	78.45	21.61	20.23	55.17	6.9	0
1 x	39.7	7.28	96.32	81.12	64	8	0
1 x	2.21	86.82	2.02	1	61.36	2.27	0
1 x	56.86	91.33	72.58	1	57.14	3.57	0
1 x	62.72	94.48	14.46	20.23	54.39	1.75	0
1 x	1	3.34	70.28	1	63.16	10.53	0
1 x	75.78	56.48	3.7	11	53.21	2.75	3.67
1 x	16.49	73.71	67.28	66.58	55.88	2.94	0
1 x	16.96	99	39.59	1	58.33	0	0
1 x	68.59	6.34	65.66	45.75	50.85	0	0
1 x	26.1	98.04	11.63	1	55.77	0	0
1 x	26.73	34.42	17.04	1	57.43	1.98	0
1 x	19.66	40.06	74.45	32.27	64.35	6.96	0
1 x	1	1	99	20.23	66.67	11.11	0
1 x	10.19	5.61	77.17	97.84	55.56	8.89	0
1 x	10.19	5.61	77.17	97.84	55.56	8.89	0
1 x	3.37	92.24	10.18	5.19	61.11	9.26	0
1 x	32.63	99	2.73	99	55	0	0
1 x	94.15	40.06	14.83	52.04	57.73	1.03	0
1 x	49.68	40.06	1	52.41	56.25	2.08	0
1 x	12.37	5.27	66.42	3.56	68.18	2.27	0
1 x	48.6	65.16	4.07	99	63.83	2.13	0
1 x	18.12	64.19	1	99	61.22	2.04	0
1 x	42.52	1	84.92	1	42.86	4.76	0
1 x	26.1	11.66	54.55	96.74	59.38	6.25	0
1 x	80.4	40.06	1	1	47.37	0	0
1 x	35.59	84.23	18.08	1	55.95	2.38	1.19
1 x	5.35	71.32	1	1	70.27	5.41	0

1 x	6.68	40.06	13.81	99	63.64	6.06	0
1 x	1	82.58	1	99	52.63	5.26	0
1 x	11.88	40.06	60.9	5.5	64.29	5.36	0
1 x	9.61	8.88	82.08	1.26	62.91	5.3	0
1 x	1.16	96.04	1	1	60	0	0
1 x	26.1	84.23	1	1	62.5	0.83	0
1 x	1.78	82.12	1	9.52	57.78	0	2.22
1 x	66.04	92.24	1.17	2.17	44.44	0	0
1 x	2.8	71.57	2.49	47.86	65.45	5.45	0
1 x	22.17	10.48	1	20.23	53.33	0	0
1 x	95.53	57.59	1.39	99	50	0	2.94
1 x	1.97	94.84	15.38	20.23	43.75	12.5	0
1 x	10.19	28.59	1	4.22	58.33	4.17	0
1 x	1	40.06	63.35	20.23	53.33	6.67	0
1 x	18.12	79.51	2.36	20.23	42.86	0	0
1 x	89.52	99	1	99	68.42	0	0
1 x	8.96	85.08	63.35	1.98	57.14	5.71	0
1 x	19.3	82.98	13.33	20.23	64	4	0
1 x	33.38	1	98.13	1	62.96	3.7	0
1 x	25.81	62.95	79.73	10.62	63.29	5.8	0
1 x	26.1	99	1	99	25	0	0
1 x	99	77.41	19.26	1	40	0	0
1 x	35.59	79.51	24.32	98.65	57.14	7.14	0
1 x	16.08	1	98.13	1	66.67	0	0
1 x	43.72	84.73	1	5.94	47.46	0	0
1 x	30.37	40.06	63.35	72.45	60	10	0
1 x	89.52	1	99	20.23	40	0	0
1 x	14.64	97.29	1.33	7.29	68.12	0	1.45
1 x	1.5	99	50.45	76.62	67.27	5.45	3.64
1 x	41.25	40.06	76.37	28.93	62.34	5.19	3.9
1 x	36.67	99	33.61	1	60.53	0	0
1 x	62.1	40.06	1	1	37.5	0	0
1 x	16.49	93.56	12.44	95.42	64.71	0	0
1 x	10.82	94.56	37.99	1	58.9	0	0
1 x	69.05	1	98.38	1	50	0	0
1 x	1	92.24	77.17	20.23	55.56	11.11	0
1 x	5.64	99	1.63	20.23	56.25	0	0
1 x	32.63	97.73	15.38	20.23	60	7.5	0
1 x	1	1	10.18	20.23	55.56	0	0
1 x	16.24	92.77	2.02	1	47.73	2.27	0
1 x	95.04	26.01	76.37	20.23	44.16	2.6	0
1 x	40.88	63.27	60.65	4.72	56.86	0	0
1 x	3.37	51.1	52.89	48.44	62.96	9.26	1.85
1 x	84.92	24.36	1	66.58	47.06	0	0
1 x	89.52	40.06	1	20.23	33.33	0	0
1 x	1	99	35.01	1	60	8	4
1 x	82.85	48.08	27.74	7.88	56.76	0	0
1 x	1.97	40.06	15.38	1	68.75	6.25	0
1 x	1.78	99	10.18	1	55.56	0	0
1 x	1	77.41	1.91	97.84	63.33	0	0

1	x	11.51	99	1	7.54	63.38	4.23	0
1	x	10.19	2.75	77.17	20.23	44.44	11.11	0
1	x	99	98.04	98.89	99	46.15	0	0
1	x	51.7	40.06	75.94	4.56	72	6	2
1	x	3.8	73.71	5.72	1	64.71	2.35	1.18
1	x	49.68	18.97	15.38	1	50	4.17	0
1	x	20.65	90.5	18.63	1	64.15	3.77	3.77
1	x	8.85	87.7	5.64	20.23	60.94	6.25	1.56
1	x	69.8	18.97	39.59	40.61	65.28	5.56	0
1	x	69.8	18.97	39.59	40.61	65.28	5.56	0
1	x	3.37	98.75	1	5.19	70.37	0	0
1	x	50.85	96.76	29.27	1	59.3	1.16	0
1	x	8.64	78.51	8.54	4.43	67.48	2.03	0.41
1	x	21.6	62.84	1	49.66	61.54	0	0
1	x	3.23	49.82	18.18	99	67.21	1.64	0
1	x	44.9	74.06	50.97	8.95	60.32	1.98	1.19
1	x	39.7	3.95	7.03	20.23	40	0	0
1	x	39.7	77.41	19.26	20.23	46.67	0	0
1	x	30.18	11.27	27.91	20.23	63.83	6.38	0
1	x	37.99	99	46.57	95.42	44.12	2.94	0
1	x	61.53	67.92	1.7	1	52.38	3.17	0
1	x	1	1	89.39	20.23	58.33	8.33	0
1	x	2.59	62.84	11.63	1	65.38	3.85	0
1	x	24.64	18.24	31.47	14.89	59.42	4.83	0
1	x	34.33	58.66	15.38	20.23	54.69	0	0
1	x	3.8	98.04	9.12	43.1	55.38	1.54	0
1	x	11.71	76.43	1.77	20.23	54.84	6.45	0
1	x	1	74.89	3.57	51.68	67.35	2.04	0
1	x	30.79	82.78	34.3	20.23	60.26	1.32	0
1	x	41.01	76.75	85.35	53.96	60.87	4.35	0
1	x	17.22	40.06	35.88	20.23	64.52	3.23	0
1	x	43.4	49.35	19	6.65	60.94	3.13	0
1	x	69.66	74.37	41.37	15.98	57.14	1.13	0.75
1	x	57.59	50.51	33.61	46.76	60.53	1.75	1.75
1	x	59.91	68.22	66.6	47.67	57.83	3.01	0.6
1	x	6.92	99	1	7.66	59.72	1.39	6.94
1	x	22.17	99	63.35	97.84	53.33	0	6.67
1	x	30.37	99	1	97.84	60	0	3.33
1	x	27.51	5.61	77.17	99	60	8.89	0
1	x	63.92	84.73	1.98	1.14	55.93	1.69	0
1	x	96.85	63.73	1	81.12	56	0	0
1	x	20.83	99	13.81	67.94	66.67	3.03	9.09
1	x	81.45	79.51	5.94	57.46	50	2.38	0
1	x	11.51	64.99	16.15	1	61.97	1.41	1.41
1	x	19.94	58.38	63.35	43.1	64.62	6.15	4.62
1	x	48.23	24.76	24.32	1	60	2.86	0
1	x	81.29	95.44	6.29	6.37	54.84	0	6.45
1	x	36.2	97.86	13.81	20.23	66.67	0	0
1	x	67.62	90.88	3.19	20.23	57.89	0	0
1	x	5.28	99	21.61	1.05	65.52	0	10.34

1 x	37.45	47.76	24.32	91.84	63.64	5.19	2.6
1 x	11.2	1	85.35	99	65.22	8.7	0
1 x	87.4	65.68	4.38	99	59.42	2.9	1.45
1 x	3.8	1.24	99	97.84	53.33	6.67	0
1 x	45.8	81.78	2.93	60.52	69.23	0	0
1 x	73.45	29.82	55.58	15.11	57.14	4.61	0
1 x	54.03	4.03	94.94	20.23	59.6	5.96	0.66
1 x	39.23	63.91	83.05	6.37	62.1	2.82	3.63
1 x	63.53	7.28	99	1	48	8	0
1 x	7.34	40.06	56.69	1	57.14	4.76	2.38
1 x	55.7	7.28	63.35	4.56	60.67	5.33	0.67
1 x	47.86	28.22	24.9	20.23	53.96	1.44	0
1 x	9.13	16.2	59.99	98.92	56.1	14.63	0
1 x	44.1	33.09	6.75	2.93	60.49	4.94	0
1 x	3.91	88.69	6.36	20.23	67.96	3.88	4.85
1 x	68.82	60.07	52.67	6.01	58.82	3.36	2.52
1 x	74.19	74.29	97.54	34.28	61	3	0
1 x	9.26	6.28	98.23	83.95	63.83	2.13	0
1 x	5.64	1.71	1	99	37.5	0	0
1 x	8.37	65.68	92.58	1	56.52	4.35	0
1 x	37.52	20.62	62.07	2.04	57.55	6.6	0
1 x	1.97	1	15.38	1.56	62.5	15.63	0
1 x	89.52	81.78	1	99	38.46	7.69	0
1 x	1	61.28	24.32	1	67.86	0	0
1 x	33.38	1	99	93.93	66.67	5.56	0
1 x	37.02	40.06	5.5	98.39	60.47	4.65	2.33
1 x	79.1	97.48	1	1	52.94	2.94	0
1 x	94.5	96.39	1	20.23	50	0	0
1 x	43.04	62.44	41.82	1	57.55	6.6	0
1 x	93.64	21.49	24.32	20.23	50	3.57	0
1 x	95.3	92.24	10.18	99	50	0	0
1 x	22.78	88.23	8.84	20.23	57.87	4.49	3.37
1 x	1	22.56	3.81	20.23	65	3.33	0
1 x	89.52	40.06	68.87	1	62.5	4.17	0
1 x	23.31	63.88	31.32	7.94	59.06	2.01	0
1 x	14.21	63.6	56.6	11.54	63.35	3.69	0.85
1 x	53.56	17.19	92.75	68.66	63.08	3.08	0
1 x	44.51	80.39	76.27	41.97	58.09	1.47	0
1 x	89.52	20.31	30.98	20.23	38.46	0	0
1 x	53.29	67.92	31.58	8.95	61.9	0	2.38
1 x	73.93	32.46	98.19	39.97	55.41	5.41	0
1 x	13.62	60.57	56.1	1.05	62.07	5.17	0
1 x	49.68	98.75	30.29	93.93	55.56	2.78	0
1 x	10.19	99	1.51	1.71	54.55	0	15.15
1 x	22.98	2.75	77.17	37.01	67.25	7.6	0
1 x	39.7	78.45	76.32	63.69	59.31	4.14	5.52
1 x	23.27	4.61	84.92	3.2	66.67	9.52	0
1 x	37.61	1.14	49.26	5.43	62.16	8.11	0
1 x	1	99	1	83	75	4.17	12.5
1 x	7.14	90.19	16.81	8.34	65.38	3.85	0

1	x	1	98.75	10.18	1	72.22	5.56	0
1	x	1.5	74.59	13.81	96.12	57.58	6.06	0
1	x	21.81	26.81	99	20.23	56.1	4.88	0
1	x	2.08	99	17.84	95.08	73.91	0	7.25
1	x	89.52	40.06	1	99	0	0	0
1	x	8.72	33.55	67.94	20.23	63.22	6.9	1.15
1	x	7.54	98.99	18.39	80.5	71.05	1.32	1.32
1	x	96.35	40.06	1	20.23	7.14	0	0
1	x	27.08	96.64	9.12	99	58.46	1.54	3.08
1	x	73.93	98.5	3.49	1	59.46	0	0
1	x	84.4	98.45	1	1.4	61.29	0	3.23
1	x	66.92	34.48	26.78	10.51	58.82	2.94	0
1	x	14.06	81.78	4.55	35.85	75.82	1.1	0
1	x	13.87	8.72	46.57	41.97	63.24	7.35	0
1	x	17.64	55.22	59.84	5.94	64.41	4.24	0
1	x	5.64	40.06	45.52	59.44	60	2.5	0
1	x	7.5	99	1.91	54.76	71.11	0	8.89
1	x	1	99	1	99	87.5	0	0
1	x	20.83	40.06	66.42	86.79	61.36	2.27	0
1	x	47.48	65.68	55.19	7.29	62.32	2.9	0
1	x	7.54	77.01	1.85	20.23	61.84	0	1.32
1	x	17.96	98.86	3.94	55.18	64.61	1.69	0.56
1	x	10.94	99	6.65	89.9	70.49	3.28	1.64
1	x	48.72	72.6	1	49.04	58.49	1.89	0
1	x	93.52	94.12	7.91	1	67.24	0	0
1	x	10.19	99	1	99	66.67	0	0
1	x	1	63.73	13.33	20.23	72	4	0
1	x	31.5	97.37	1	20.23	64.58	0	0
1	x	93.76	40.06	27.44	20.23	59.26	0	0
1	x	23.79	62.84	30.98	99	60.58	4.81	0
1	x	52.84	75.49	15.38	96.74	59.38	0	0
1	x	49.68	98.75	99	99	75	0	0
1	x	60.85	98.59	1	11.45	58.62	0	0.86
1	x	5.64	99	15.38	1	68.75	0	0
1	x	12.03	91.25	1	33.82	65.05	0	0
1	x	1	99	1	1	68.75	3.13	0
1	x	10.19	40.06	70.28	46.76	59.65	5.26	0
1	x	71.44	1	5.07	99	45.45	9.09	0
1	x	23.79	81.78	15.38	79.29	58.65	1.92	0
1	x	1	1	99	20.23	62.5	0	0
1	x	54.7	81	15.38	90.5	57.5	2.5	0
1	x	14.18	82.58	50.06	28.01	62.28	0	0.29
1	x	37.52	62.44	10.87	78.39	58.49	3.77	0
1	x	99	40.06	5.07	99	36.36	0	0
1	x	76	73.29	4.38	41.62	60.87	0	0
1	x	28.93	98.61	1.44	86.16	71.64	1.49	0
1	x	13.27	75.49	1	20.23	68.75	6.25	0
1	x	16.49	99	1	1	70.59	0	0
1	x	67.11	91.33	15.38	47.3	64.29	1.79	0
1	x	5.88	99	2.18	3.38	67.44	2.33	0

1 x	53.56	99	2.93	99	46.15	0	0
1 x	6.68	99	1	1	63.64	0	0
1 x	48.96	99	1	1	59.15	0	4.23
1 x	1	98.75	1	48.44	72.22	3.7	0
1 x	13.5	40.06	1	72.45	67.78	4.44	0
1 x	31.07	1	2.93	1	69.23	0	0
1 x	96.08	99	1	97.84	46.67	0	6.67
1 x	54.15	69.54	29.73	20.23	50.63	0	2.53
1 x	4.85	81.78	2.93	20.23	53.85	7.69	0
1 x	9.23	81.78	9.79	97.66	57.14	0	0
1 x	50.71	28.8	78.4	82.06	51.02	2.04	2.04
1 x	37.3	64.67	3.81	83	54.17	4.17	0
1 x	83.25	62.84	11.63	20.23	61.54	3.85	0
1 x	14.64	65.68	17.84	99	60.87	0	0
1 x	15.41	1	98.38	20.23	60	20	0
1 x	1.5	96.39	50.45	20.23	63.64	4.55	0
1 x	21.79	75.49	43.94	16.07	62.5	2.57	6.99
1 x	71.44	40.06	5.07	20.23	51.52	3.03	3.03
1 x	1	15.75	28.56	20.23	65	10	5
1 x	2.35	40.06	1	20.23	64.29	0	0
1 x	3.8	69.2	95.54	90.5	67.5	2.5	0
1 x	21.6	99	1	99	53.85	3.85	0
1 x	3.27	3.25	61.28	42.71	71.21	6.06	0
1 x	1	1.71	89.39	20.23	62.5	12.5	0
1 x	58.76	17.46	95.15	3.56	59.09	0	2.27
1 x	14.43	90.01	46.17	48.44	66.67	0.93	1.85
1 x	71.81	14.81	48.36	35.11	51.58	0.53	0
1 x	15.41	3.95	98.38	20.23	60	10	0
1 x	80.96	3.95	98.38	20.23	50	5	0
1 x	1	1	98.38	20.23	60	10	0
1 x	14.06	7.93	81.58	99	69.23	7.69	0
1 x	60.52	65.68	4.38	84.9	52.17	0	0
1 x	19.76	40.06	77.17	5.19	64.81	3.7	0
1 x	45.12	5.27	50.45	99	36.36	9.09	0
1 x	10.19	1	99	1	58.33	8.33	0
1 x	89.52	40.06	1	20.23	0	0	0
1 x	7.34	1	99	99	64.29	7.14	0
1 x	6.68	40.06	5.07	99	72.73	0	0
1 x	1	40.06	1	20.23	66.67	0	0
1 x	1	6.61	39.59	1	58.33	0	0
1 x	39.7	6.61	89.39	1.23	63.33	1.67	0
1 x	89.52	71.57	1	1	58.18	0	0
1 x	30.77	79.94	34.62	1.92	60.87	2.9	4.35
1 x	77.34	96.04	63.35	97.84	60	0	0
1 x	1.78	40.06	10.18	20.23	77.78	11.11	0
1 x	1	99	26.35	58.42	53.66	0	2.44
1 x	89.52	2.75	77.17	20.23	55.56	0	0
1 x	29.85	79.15	17.36	9.05	57.65	2.35	0
1 x	33.38	95.32	1.45	2.7	62.39	0.85	0
1 x	1	78.45	2.1	20.23	62.07	0	0

1 x	34.33	1	32.54	1	65.63	3.13	0
1 x	1.57	9.98	28.97	63.38	65.75	2.74	2.74
1 x	14.06	31.46	30.98	20.23	56.92	3.08	0
1 x	74.95	81.78	2.93	2.7	46.15	0	0
1 x	20.83	99	5.07	1	63.64	0	0
1 x	63.53	55.96	22.74	39.67	64.67	0.67	0
1 x	67.11	30.13	72.58	92.84	53.57	1.79	0
1 x	26.1	57.27	2.93	10.65	62.5	0	0
1 x	45.8	81.78	1	99	56.41	0	0
1 x	5.14	67.92	99	20.23	66.67	0	4.76
1 x	1	96.39	1	1	63.64	0	0
1 x	14.64	8.97	44.75	1	57.97	2.9	1.45
1 x	68.66	65.68	17.84	1.92	65.22	0	0
1 x	6.92	13.82	77.17	2.17	66.67	2.78	0
1 x	3.8	7.28	7.03	20.23	62	0	4
1 x	20.63	88	55.97	4.13	58.95	0	0
1 x	88.4	54.16	15.81	6.58	61.42	1.57	0
1 x	49.68	29.78	27.44	5.19	57.41	1.85	0
1 x	11.83	55.61	6.03	5.73	60.87	2.61	0
1 x	98.09	18.24	9.43	1	45.65	2.17	0
1 x	1.9	82.98	1	20.23	68	4	0
1 x	14.91	11.16	21.44	5.27	66.97	3.67	0
1 x	11.97	62.44	68.36	1	62.26	0	0
1 x	35.59	40.06	24.32	98.65	50	7.14	0
1 x	11.36	3.95	1	1	65	7.5	0
1 x	24.55	40.06	7.69	2.7	71.79	5.13	0
1 x	79.1	2.18	12.44	20.23	64.71	0	0
1 x	99	40.06	97.09	1	50	0	0
1 x	98.55	1.71	89.39	20.23	50	0	0
1 x	45.12	23.94	95.15	20.23	51.52	3.03	0
1 x	12.97	1	11.24	1.98	71.43	0	0
1 x	10.19	63.27	32.94	1	60.78	0	0
1 x	2.87	40.06	39.59	20.23	66.67	0	0
1 x	13.76	2.06	84.92	57.46	61.9	2.38	0
1 x	62.1	1.71	89.39	1	50	0	0
1 x	44.27	31.46	41.41	6.78	66.15	0	0
1 x	68.25	1	67.28	95.42	55.88	1.47	0
1 x	65.54	99	10.77	44.38	54.84	0	0
1 x	81.45	40.06	84.92	20.23	38.1	0	0
1 x	26.1	6.61	96.01	2.17	52.78	0	0
1 x	50.71	90.06	87.61	51.68	51.02	0	0
1 x	45.12	99	1	99	54.55	0	0
1 x	48.6	98.95	27.91	20.23	46.81	0	0
1 x	12.37	99	5.07	20.23	59.09	0	0
1 x	1	99	1	20.23	65.22	0	0
1 x	52.34	99	1	92.27	52.63	0	0
1 x	15.41	40.06	7.03	99	60	0	0
1 x	28.63	63.73	1	99	64	0	0
1 x	18.87	95.32	7.69	91.4	69.23	0	0
1 x	97.77	40.06	63.35	99	50	0	0

1 x	35.2	82.38	21.25	50.31	62.75	3.92	0
1 x	37.7	99	1.49	32.15	63.79	0.86	0
1 x	20.07	55.75	12.73	92.27	64.47	1.32	0
1 x	1.05	73.71	1	99	70.59	0	5.88
1 x	1	99	15.38	96.74	71.88	3.13	0
1 x	83.65	80.05	1	1	56.36	0	0
1 x	40.49	93.71	25.39	92.27	61.84	2.63	0
1 x	23.88	62.05	10.18	20.23	59.26	7.41	0
1 x	23.22	34.53	31.95	1.89	60.19	6.8	1.94
1 x	26.1	82.26	98.17	88.05	62.5	6.25	0
1 x	13.27	97.11	54.55	5.5	67.86	0	1.79
1 x	6.77	94.16	13.68	71.92	76.92	0	0
1 x	9.02	75.76	83.21	5.43	63.06	4.5	0
1 x	33.63	68.76	37.69	20.23	64.75	2.46	2.46
1 x	1	99	5.94	99	76.19	4.76	0
1 x	20.83	86.82	7.03	11.07	65.45	1.82	0
1 x	20.1	85.08	19.26	20.23	70.48	2.86	0.95
1 x	33.38	89.16	43.97	2.93	70.37	0	0
1 x	2.87	99	1	99	66.67	0	0
1 x	1	10.48	19.26	97.84	73.33	6.67	0
1 x	23.88	97.57	39.59	93.93	51.85	1.85	0
1 x	2.7	99	13.92	10.86	70.09	0.93	1.87
1 x	34.62	92.62	10.77	70.91	62.9	3.23	4.03
1 x	2.35	91.33	1	1	75	3.57	0
1 x	59.18	13.82	62.27	12.02	64.29	7.14	0
1 x	9.48	95.44	58.85	70.91	61.29	6.45	3.23
1 x	18.12	92.91	91.58	1.98	71.43	0	2.86
1 x	23.88	40.06	27.44	77.5	70.37	7.41	0
1 x	35.59	79.51	1	75.77	71.43	0	0
1 x	24.02	99	13.6	46.24	60.34	0	0
1 x	9.45	99	7.47	20.23	74.58	0	0
1 x	15.41	15.75	63.35	90.5	75	5	0
1 x	23.35	94.52	52.48	43.1	58.46	3.08	0
1 x	9.6	94.28	13.97	93.12	66.22	0	0
1 x	7.19	99	1	1	60.38	1.89	0
1 x	13.44	99	1.84	71.67	63.93	3.28	4.92
1 x	16.76	40.06	6.47	89.58	63.41	0	0
1 x	84.4	59.25	58.85	97.31	48.39	0	0
1 x	1	99	1	20.23	70	0	10
1 x	49.68	93.37	51.55	72.45	60	3.33	1.67
1 x	1.5	99	5.07	67.94	63.64	0	0
1 x	1	55.75	3.19	2.53	68.42	2.63	0
1 x	24.55	81.78	48.76	99	64.1	0	0
1 x	23.77	99	12.16	97.41	68.83	0	2.6
1 x	35.59	99	1	1.66	55.1	0	0
1 x	58.88	63.05	15.91	79.74	64.08	2.91	0
1 x	15.41	99	7.03	97.84	66.67	0	0
1 x	17.28	97.73	22.74	1.76	68	2	3
1 x	6.56	91.56	13.97	1	66.22	1.35	2.7
1 x	6.89	95.94	32.79	89.13	65.06	1.2	0

1 x	20.83	99	5.07	99	59.09	0	0
1 x	22.41	99	89.39	69.4	64.06	1.56	1.56
1 x	21.24	99	89.39	1	60.42	0	8.33
1 x	1	99	2.93	99	69.23	0	0
1 x	24.37	40.06	42.95	99	68.57	1.43	0
1 x	10.19	40.06	1	20.23	66.67	0	0
1 x	54.26	97.86	13.81	67.94	59.09	0	1.52
1 x	1.86	70.24	3.05	91.84	67.53	3.9	2.6
1 x	10.19	88.15	1	99	61.9	0	0
1 x	7.95	99	1.07	62.77	67.57	2.7	8.11
1 x	34.72	99	92.58	1	63.04	0	0
1 x	21.6	99	1	99	69.23	0	0
1 x	35.2	99	60.65	80.19	68.63	3.92	0
1 x	2.72	19.67	22.74	95.9	64	2	0
1 x	19.15	99	70.28	46.76	61.4	3.51	3.51
1 x	1	60.57	43.66	20.23	68.97	3.45	0
1 x	41.98	20.31	30.98	49.66	67.31	3.85	0
1 x	26.1	91.85	43.27	99	54.69	4.69	0
1 x	17.22	59.25	6.29	20.23	61.29	0	0
1 x	77.83	83.23	17.19	70.91	62.9	0	0
1 x	2.72	94.01	1.46	20.23	68	4	0
1 x	3.04	46.49	60.36	1	69.57	1.09	1.09
1 x	19.03	87.7	75.23	69.4	62.5	3.13	0
1 x	11.2	99	1.81	97.48	67.39	2.17	0
1 x	13.92	99	1.72	92.61	72.34	2.13	0
1 x	5	60.74	96.08	5.73	65.22	4.35	0
1 x	4.45	99	26.35	20.23	69.11	1.63	1.63
1 x	10.19	99	1	99	70.83	0	0
1 x	19.3	94.01	1	99	64	4	0
1 x	43.4	75.49	39.59	52.41	60.42	4.17	0
1 x	2.12	47.2	25.3	57.93	77.11	1.2	3.61
1 x	2.65	99	9.35	96	69.88	1.2	0
1 x	1	98.98	26.35	89.58	63.41	0	0
1 x	10.19	99	1	99	60	0	0
1 x	22.45	94.66	34.53	92.55	58.41	1.77	0
1 x	11.71	99	1	70.91	77.42	0	3.23
1 x	19.3	91.22	13.33	2.44	62.67	0	4
1 x	47.93	90.41	67.94	20.23	62.07	0	0
1 x	57.83	67.32	3.54	56.53	63.95	1.16	0
1 x	57.83	67.32	3.54	56.53	63.95	1.16	0
1 x	1	99	66.94	20.23	77.68	0	3.57
1 x	20.17	99	43.66	74.09	72.41	0	0
1 x	1	55.96	85.71	7.99	72	2.67	2.67
1 x	1.39	99	68.87	83	79.17	4.17	8.33
1 x	35.92	99	3.62	89.9	59.02	0	0
1 x	6.03	98.75	5.41	5.19	70.37	3.7	3.7
1 x	1	84.23	15.38	4.22	70.83	0	0
1 x	42.52	16.63	1	20.23	66.67	0	0
1 x	2.87	99	89.39	20.23	61.11	0	0
1 x	31.07	1	30.98	99	69.23	0	0

1	x	12.09	91.22	44.33	7.99	66.67	2.67	0
1	x	16.96	40.06	15.38	99	62.5	4.17	0
1	x	8.11	99	1	90.5	60	0	0
1	x	10.19	89.5	7.03	1	63.33	5	0
1	x	11.2	97.92	4.38	84.9	67.39	0	0
1	x	45.12	99	1	99	54.55	0	0
1	x	16.76	91.94	4.11	58.42	63.41	0	0
1	x	1.05	99	4.6	99	67.65	2.94	5.88
1	x	14.51	89.96	12.81	20.23	66.95	0.85	0
1	x	1.05	99	1	99	58.82	0	0
1	x	23.51	99	29.9	20.23	68.48	0	1.09
1	x	1	73.71	12.44	95.42	76.47	0	0
1	x	28.02	97.86	50.45	20.23	66.67	1.52	0
1	x	53.56	99	2.93	99	71.79	0	0
1	x	28.44	72.07	86.01	77.5	66.67	7.41	0
1	x	62.1	99	1.63	99	43.75	0	0
1	x	5.64	87.7	15.38	20.23	62.5	3.13	3.13
1	x	75.4	47.47	36.72	8.55	57.5	0	0
1	x	96.67	40.06	30.98	20.23	76.92	0	0
1	x	41.36	72.07	10.18	64	58.33	0	2.78
1	x	28.44	2.75	77.17	77.5	55.56	3.7	0
2	x	63.29	14.45	27.14	39.82	57.72	1.34	0
2	x	69.05	69.2	1	20.23	60	5	0
2	x	69.05	69.2	1	20.23	60	5	0
2	x	34.12	26.81	42.46	98.92	56.1	7.32	0
2	x	89.52	98.75	1	20.23	66.67	0	0
2	x	46.7	54.11	3.67	37.12	57.65	4.71	1.18
2	x	89.52	96.04	1.91	72.45	56.67	0	0
2	x	41.62	1.48	79.1	97.31	58.06	6.45	0
2	x	1	40.06	12.44	95.42	58.82	11.76	0
2	x	91.88	80.9	34.26	5.11	54.21	1.87	0
2	x	98.59	88	26.97	35.11	51.58	0	0
2	x	91.13	89.16	1	20.23	50.62	1.23	0
2	x	95.09	70.59	1	20.23	42.11	0	0
2	x	35.05	98.5	1.07	93.12	56.76	2.7	2.7
2	x	85.37	66.32	54.55	32.62	49.11	1.79	0
2	x	26.1	64.67	39.59	20.23	58.33	0	0
2	x	94.68	40.06	84.92	1	47.62	4.76	0
2	x	20.83	96.39	99	20.23	68.18	2.27	0
2	x	55.8	40.06	87.24	8.75	56.1	1.22	0
2	x	65.65	5.84	89.39	20.23	48.75	1.25	0
2	x	47.78	66.57	2.75	20.23	57.89	1.5	4.51
2	x	24.55	95.32	16.81	20.23	64.1	2.56	0
2	x	37.52	29.6	10.87	5.02	60.38	1.89	0
2	x	52.84	49.35	32.54	20.23	59.38	1.56	0
2	x	46.7	73.71	67.28	20.23	44.12	0	5.88
2	x	5.64	98.75	39.59	20.23	50	0	8.33
2	x	13.44	6.87	96.14	1	65.57	6.56	1.64
2	x	26.1	40.06	89.39	1	75	0	0
2	x	21.43	63.73	49.13	20.23	56	2	3

2 x	76.24	6.61	48.09	8.95	53.57	4.76	0
2 x	18.12	40.06	2.36	1	60.71	3.57	0
2 x	2.63	27.89	1	54.76	66.67	2.22	0
2 x	40.88	40.06	1.39	20.23	62.75	1.96	0
2 x	99	1.08	99	20.23	54.55	0	0
2 x	22.98	1.34	84.38	61.63	55.26	5.26	0
2 x	69.05	1	98.38	20.23	50	20	0
2 x	20.17	3.55	86.26	20.23	62.07	3.45	0
2 x	46.7	12.77	67.28	20.23	47.06	5.88	0
2 x	10.19	2.75	1	99	55.56	0	0
2 x	28.39	26.95	66.6	20.23	67.47	2.41	1.2
2 x	22.17	22.56	39.59	20.23	66.67	3.33	0
2 x	95.3	40.06	10.18	20.23	38.89	0	0
2 x	41.62	59.25	58.85	20.23	54.84	3.23	3.23
2 x	42.22	18.61	16.56	20.23	59.57	4.26	0
2 x	89.52	50.33	31.84	46.24	53.45	1.72	0
2 x	41.52	12.03	14.31	20.23	58.16	1.02	0
2 x	99	82.98	35.01	20.23	44	0	0
2 x	99	24.76	42.95	65.27	42.86	2.86	0
2 x	14.06	99	11.63	20.23	65.38	0	3.85
2 x	94.88	97.73	7.03	90.5	45	0	0
2 x	31.07	7.93	1	1	46.15	0	0
2 x	32.77	52.23	14.31	4.4	57.14	2.04	0
2 x	43.4	11.66	15.38	96.74	56.25	0	0
2 x	74.95	1	2.93	99	76.92	0	0
2 x	48.6	65.16	4.07	4.04	48.94	0	0
2 x	13.62	1	43.66	20.23	65.52	6.9	0
2 x	2.54	1	86.68	20.23	66.67	3.03	0
2 x	49.68	3.95	99	72.45	56.67	10	0
2 x	10.19	40.06	56.69	88.66	57.14	0	0
2 x	39.7	10.48	19.26	20.23	46.67	0	0
2 x	69.55	7.4	67.11	10.86	54.21	5.61	0
2 x	66.04	20.91	52.89	33.13	53.7	0.93	0
2 x	9.45	30.62	99	5.94	64.41	6.78	1.69
2 x	69.05	74.29	96.32	95.9	50	2	2
2 x	37.7	9.86	95.69	98.26	51.72	3.45	0
2 x	14.87	1	98.58	20.23	57.58	9.09	0
2 x	94.88	69.2	1	20.23	60	0	0
2 x	93.64	40.06	89.39	20.23	60.71	7.14	0
2 x	3.43	98.18	96.5	99	59.38	3.13	0
2 x	67.62	99	33.61	20.23	52.63	0	0
2 x	51.97	96.39	50.45	86.79	47.73	2.27	0
2 x	29.57	91.79	5.07	20.23	60	0	0
2 x	89.52	11.66	54.55	96.74	62.5	0	0
2 x	94.33	65.68	17.84	20.23	69.57	0	0
2 x	54.26	1.92	50.45	1.71	57.58	12.12	0
2 x	97.37	1	5.07	20.23	72.73	9.09	0
2 x	42.14	6.83	67.02	40.28	52.05	2.74	0
2 x	41.16	68.54	75.67	3.01	56.1	0	7.32
2 x	1.97	52.49	89.39	52.41	60.42	4.17	6.25

2 x	33.38	62.05	27.44	77.5	66.67	3.7	0
2 x	59.67	77.41	39.59	20.23	60	0	0
2 x	1.97	40.06	96.5	69.4	71.88	3.13	0
2 x	98.27	24.66	2.79	29.97	42.45	0.36	0
2 x	28.63	1.98	63.35	20.23	60	0	2
2 x	6.03	20.91	27.44	5.19	68.52	5.56	3.7
2 x	66.04	40.06	1	20.23	11.11	0	0
2 x	1.62	1	99	90.5	75	5	0
2 x	1	1	99	20.23	80	20	0
2 x	82.85	32.46	91.47	39.97	52.7	2.7	0
2 x	89.52	3.95	63.35	1	50	10	0
2 x	1	1	7.03	20.23	55	0	0
2 x	92.9	18.73	61.43	40.95	54.93	2.82	1.41
2 x	80.1	56.18	55.76	62.77	62.16	0	0
2 x	27.64	26.81	75.67	20.23	58.54	4.88	2.44
2 x	67.62	90.88	33.61	20.23	57.89	0	0
2 x	56.86	1.51	97.09	41.28	52.86	5.71	1.43
2 x	26.1	94.84	1	20.23	62.5	0	0
2 x	49.68	23.94	20.79	42.71	57.58	1.52	0
2 x	89.52	79.51	1	20.23	39.29	0	0
2 x	1.78	1	99	20.23	55.56	22.22	0
2 x	99	19.67	85.71	20.23	64	4	0
2 x	1.97	99	1.63	20.23	62.5	0	0
2 x	10.19	40.06	10.18	77.5	70.37	3.7	0
2 x	89.52	88.59	79.1	20.23	51.61	3.23	6.45
2 x	27.64	4.28	94.28	89.58	58.54	2.44	0
2 x	33.38	2.75	99	20.23	44.44	11.11	0
2 x	29.85	73.71	1.39	20.23	70.59	5.88	0
2 x	1	99	1	20.23	70	0	0
2 x	1.05	12.77	1	20.23	52.94	5.88	0
2 x	89.52	26.5	95.54	20.23	42.5	2.5	0
2 x	62.1	40.06	15.38	20.23	62.5	0	0
2 x	85.63	26.34	65.08	59.98	52.53	0	0.63
2 x	87.02	15.45	20.4	73.26	49.15	0	0
2 x	51.97	53.63	66.42	20.23	61.36	2.27	0
2 x	20.01	1	99	53.17	55.32	8.51	0
2 x	48.93	3.61	95.04	41.97	55.15	6.62	0
2 x	95.09	14.81	97.49	46.76	45.61	0.88	0
2 x	3.8	1	63.35	20.23	70	10	0
2 x	61.03	12.59	17.04	50.64	58.42	6.93	0
2 x	43.4	1.71	15.38	20.23	56.25	6.25	0
2 x	2.35	4.61	84.92	20.23	66.67	0	0
2 x	19.3	1.98	13.33	81.12	52	8	0
2 x	39.04	53.48	14.79	36.25	52.81	0	1.12
2 x	37.02	46.94	16.67	3.38	52.33	3.49	1.16
2 x	50.32	1.62	90.41	59.98	56.96	7.59	1.27
2 x	1	12.77	46.57	95.42	64.71	5.88	0
2 x	18.12	1	97.09	20.23	57.14	0	0
2 x	59.67	77.41	87.45	85.84	51.11	2.22	0
2 x	94.68	67.92	97.09	88.66	52.38	0	0

2 x	89.52	12.77	67.28	66.58	44.12	2.94	0
2 x	67.24	1.98	63.35	7.99	53.33	5.33	0
2 x	1	99	39.59	99	75	8.33	0
2 x	59.67	27.89	77.17	54.76	60	0	2.22
2 x	27.81	25.5	99	2.35	51.35	8.11	0
2 x	80.1	25.5	98.19	2.35	59.46	5.41	0
2 x	85.89	40.06	84.92	20.23	61.9	2.38	0
2 x	55.25	40.06	77.17	77.5	66.67	0	0
2 x	1.9	1.98	63.35	81.12	72	8	4
2 x	1	40.06	24.32	20.23	66.67	0	0
2 x	14.06	1.53	16.81	20.23	51.28	5.13	0
2 x	68.25	40.06	96.24	20.23	61.76	4.41	0
2 x	64.21	24.36	46.57	20.23	55.88	2.94	0
2 x	81.89	99	79.84	20.23	50	0	9.09
2 x	2.87	84.23	99	20.23	58.33	0	0
2 x	54.26	23.94	29.49	1.71	54.55	0	3.03
2 x	89.52	2.75	99	20.23	44.44	0	0
2 x	40.77	86.29	72.58	75.77	48.21	1.79	7.14
2 x	1	99	1	20.23	88.89	0	11.11
2 x	89.52	40.06	1	20.23	33.33	0	0
2 x	56.86	1	97.09	20.23	28.57	14.29	0
2 x	45.12	66.75	79.84	20.23	54.55	0	4.55
2 x	93.76	92.24	99	1	55.56	0	0
2 x	62.1	40.06	32.54	20.23	37.5	0	0
2 x	1.5	17.46	50.45	99	59.09	9.09	0
2 x	42.52	1	84.92	20.23	47.62	4.76	0
2 x	70.33	40.06	56.69	88.66	57.14	4.76	0
2 x	76.24	9.23	72.58	20.23	42.86	7.14	0
2 x	51.7	40.06	35.01	81.12	56	0	4
2 x	10.19	99	77.17	20.23	55.56	0	11.11
2 x	63.85	72.27	81.58	20.23	61.54	0	3.5
2 x	5.64	94.84	1	96.74	62.5	6.25	0
2 x	22.98	14.81	93.14	20.23	57.89	10.53	0
2 x	30.37	89.5	19.26	20.23	66.67	6.67	6.67
2 x	6.68	17.46	95.15	20.23	63.64	0	4.55
2 x	1	69.2	98.38	20.23	67.5	0	12.5
2 x	78.84	89.5	49.13	20.23	64	0	2
2 x	49.68	27.1	56.69	88.66	52.38	4.76	0
2 x	97.63	95.44	1.77	20.23	58.06	0	6.45
2 x	57.51	9.52	27.61	30.91	58.59	5.47	0
2 x	1	84.23	39.59	20.23	66.67	0	16.67
2 x	4.38	99	4.38	20.23	60.87	4.35	8.7
2 x	78.28	75.49	54.55	96.74	50	6.25	0
2 x	97.77	40.06	98.38	20.23	70	10	0
2 x	22.04	45.17	21.61	60.88	56.9	6.03	0
2 x	22.17	53.33	87.45	20.23	55.56	11.11	0
2 x	99	79.51	97.09	20.23	42.86	0	3.57
2 x	76.24	9.23	72.58	98.65	35.71	0	0
2 x	76.24	9.23	72.58	98.65	35.71	0	0
2 x	4.85	1	99	20.23	53.85	0	0

2	x	6.68	1	5.07	20.23	63.64	0	0
2	x	89.52	92.24	10.18	20.23	66.67	0	0
2	x	13.76	88.15	12.96	20.23	66.67	4.76	0
2	x	2.08	1.4	98.67	20.23	65.22	8.7	0
2	x	58.3	70.12	67.94	20.23	56.9	1.72	3.45
2	x	63.81	9.23	92.84	88.66	47.62	7.14	0
2	x	81.45	40.06	1	57.46	54.76	0	0
2	x	39.7	5.04	85.07	43.1	59.23	3.08	0.77
2	x	77.34	40.06	93.95	97.84	46.67	0	0
2	x	98.7	40.06	74.45	20.23	34.78	0	0
2	x	1.16	2.75	63.35	20.23	73.33	4.44	0
2	x	5.64	75.49	89.39	99	62.5	6.25	6.25
2	x	57.45	24.95	85.48	40.95	53.52	4.23	0
2	x	60.04	51.31	18.63	49.04	60.38	1.89	0
2	x	89.52	1.71	15.38	99	25	0	0
2	x	39.7	89.5	7.03	99	30	10	0
2	x	26.1	11.66	99	20.23	56.25	12.5	0
2	x	85.2	13.82	39.59	20.23	50	0	0
2	x	94.5	17.46	95.15	20.23	59.09	9.09	0
2	x	95.19	3.01	99	20.23	56.76	5.41	0
2	x	14.51	22.3	53.8	73.26	62.71	3.39	1.69
2	x	37.98	2.18	97.97	66.58	61.76	11.76	0
2	x	44.56	64.19	83.42	4.4	53.06	4.08	0
2	x	1	4.61	24.32	1	80.95	4.76	0
2	x	99	40.06	72.58	20.23	64.29	0	0
2	x	31.07	40.06	2.93	20.23	53.85	0	0
2	x	68.35	3.65	2.93	99	51.28	2.56	0
2	x	99	16.63	84.92	20.23	47.62	4.76	0
2	x	96.21	78.45	43.66	98.26	48.28	0	0
2	x	57.74	32.21	69.82	7.6	54.55	2.8	0.35
2	x	54.7	2.26	39.59	6.08	65	6.67	0
2	x	99	40.06	1	99	14.29	0	0
2	x	1	1	89.39	20.23	75	10	0
2	x	74.04	49.66	79.1	89.28	53.23	3.23	0
2	x	95.3	84.23	3.81	93.93	50	0	2.78
2	x	81.45	40.06	5.94	1	33.33	0	0
2	x	1	40.06	97.09	99	71.43	0	0
2	x	62.1	7.83	84.92	28.16	56.55	1.19	0
2	x	49.35	20.21	49.6	28.87	61.94	1.94	1.29
2	x	6.11	53.04	29.9	20.23	67.39	2.17	0
2	x	39.7	96.64	41.41	20.23	41.54	1.54	0
2	x	30.37	40.06	7.03	20.23	56.67	3.33	0
2	x	28.63	1.98	99	81.12	64	12	0
2	x	4.85	7.93	99	20.23	69.23	0	0
2	x	18.12	40.06	97.09	20.23	57.14	0	0
2	x	94.88	15.75	98.38	20.23	60	5	0
2	x	76.81	53.79	67.94	94.86	50.57	3.45	0
2	x	98.73	25.84	33.61	20.23	47.37	2.63	0
2	x	34.21	19.21	75.48	20.23	65.75	0	0
2	x	77.88	18.49	24.32	12.71	55	1.43	1.43

2 x	52.34	9.55	93.14	20.23	57.89	8.77	0
2 x	28.93	57.85	37.86	20.23	58.21	1.49	0
2 x	38.85	2.33	55.19	20.23	53.62	2.9	0
2 x	91.9	24.88	23.57	20.23	47.17	0	0
2 x	7.34	1	72.58	20.23	53.57	3.57	0
2 x	71.14	19.89	70.28	80.5	56.58	6.58	0
2 x	74.19	99	13.33	20.23	56	0	0
2 x	98.13	18.04	93.28	3.8	56.04	4.4	0
2 x	50.85	67.32	82.44	20.23	67.44	0	0
2 x	35.05	71.32	55.76	62.77	54.05	0	2.7
2 x	47.15	1	89.39	20.23	55	10	0
2 x	49.68	6.61	97.72	83	70.83	4.17	0
2 x	72.12	73.71	67.28	20.23	61.76	2.94	2.94
2 x	84.4	95.44	99	97.31	58.06	0	3.23
2 x	96.67	62.84	11.63	20.23	46.15	3.85	0
2 x	97.49	94.84	99	20.23	59.38	3.13	3.13
2 x	45.12	40.06	1	20.23	54.55	0	0
2 x	70.03	91.63	13.52	8.85	55.42	2.41	6.02
2 x	52.65	7.61	73.43	20.23	56.86	3.92	3.92
2 x	68.66	93.24	44.75	20.23	57.97	0	4.35
2 x	50.71	74.89	51.8	51.68	59.18	1.02	2.04
2 x	45.12	40.06	99	20.23	45.45	0	0
2 x	76.76	26.42	46.3	69.7	62.26	5.03	0
2 x	55.8	12.11	59.99	20.23	53.66	3.66	1.22
2 x	15.41	1	7.03	20.23	60	5	0
2 x	63.17	12.22	86.68	20.23	48.48	0	0
2 x	80.4	28.48	5.84	69.9	46.32	2.11	0
2 x	76.53	50.51	14.46	74.93	56.14	0	0
2 x	1	99	2.63	77.5	55.56	0	0
2 x	87.46	79.09	6.71	40.95	56.34	1.41	0
2 x	72.33	5.86	72.16	10.58	50.49	2.91	0.97
2 x	69.05	89.5	7.03	99	50	0	0
2 x	1	1	44.75	20.23	69.57	8.7	0
2 x	52.34	14.81	45.82	20.23	54.39	5.26	0
2 x	49.68	26.18	39.59	79.29	57.69	3.85	0
2 x	70.33	4.61	84.92	20.23	66.67	9.52	0
2 x	95.8	11.66	54.55	20.23	56.25	6.25	0
2 x	8.96	97.11	4.17	94.7	54.29	2.86	2.86
2 x	37.08	56.33	7.03	11.07	52.73	4.55	0
2 x	47.3	36.21	50.01	20.23	59.73	4.7	0
2 x	55.58	1	67.28	20.23	64.71	2.94	0
2 x	3.63	80.84	59.19	86.16	67.16	1.49	0
2 x	33.38	96.69	10.18	20.23	66.67	2.78	0
2 x	1	1	99	20.23	54.55	9.09	0
2 x	92.78	7.05	73.75	20.23	51.35	2.7	0
2 x	42.72	96.66	26.13	91.27	55.47	1.46	3.65
2 x	11.97	40.06	79.41	49.04	64.15	3.77	1.89
2 x	69.05	77.41	39.59	20.23	66.67	0	0
2 x	1	1	79.84	20.23	90.91	9.09	0
2 x	26.1	99	1	20.23	66.67	0	0

2 x	1	99	1	99	80	0	10
2 x	15.41	12.41	35.01	81.12	66	6	0
2 x	98.16	92.24	77.17	20.23	61.11	11.11	0
2 x	1	1	97.97	95.42	58.82	5.88	0
2 x	4.85	7.93	98.89	20.23	84.62	7.69	0
2 x	89.52	40.06	15.38	20.23	75	0	0
2 x	14.06	99	2.93	99	46.15	7.69	0
2 x	93.76	4.99	52.89	77.5	44.44	1.85	0
2 x	74.19	40.06	35.01	20.23	44	4	0
2 x	97.77	89.5	7.03	72.45	63.33	0	0
2 x	45.8	26.18	7.69	91.4	56.41	5.13	2.56
2 x	35.05	99	1.07	62.77	62.16	0	0
2 x	29.85	1	46.57	20.23	58.82	17.65	0
2 x	1	3.95	7.03	20.23	70	10	0
2 x	10.19	40.06	99	20.23	66.67	0	0
2 x	97.77	89.5	98.38	99	70	0	10
2 x	10.82	4.52	88.22	20.23	63.01	6.85	2.74
2 x	68.11	9.86	2.1	20.23	62.07	0	0
2 x	80.75	32.88	80.86	38.69	59.24	3.82	0
2 x	37.3	4.33	22.12	83	65.28	2.78	0
2 x	93.3	11.07	99	70.91	51.61	6.45	0
2 x	66.04	13.82	1.17	20.23	61.11	0	0
2 x	99	86.82	50.45	99	54.55	0	0
2 x	75.4	15.75	99	20.23	52.5	5	0
2 x	1	81.78	1	99	69.23	0	0
2 x	47.48	5.93	44.75	20.23	69.57	8.7	0
2 x	89.52	40.06	99	20.23	0	0	0
2 x	34.21	9.98	75.48	2.26	54.79	5.48	0
2 x	62.82	52.23	24.32	4.4	53.06	4.08	4.08
2 x	3.8	1	63.35	1	53.33	0	0
2 x	48.44	4.28	59.99	20.23	58.54	4.88	0
2 x	89.52	97.11	2.36	98.65	57.14	0	0
2 x	89.52	1	89.39	20.23	50	0	0
2 x	15.41	99	63.35	20.23	70	0	10
2 x	56.47	45.39	49.26	32.75	60.36	2.7	0.9
2 x	93.9	7.93	94.47	20.23	57.69	3.85	0
2 x	22.75	40.06	41.82	78.39	69.81	3.77	0
2 x	54.26	1.92	71.27	20.23	51.52	6.06	0
2 x	1	5.27	95.15	20.23	63.64	9.09	0
2 x	80.69	26.18	30.98	60.52	53.85	5.13	0
2 x	7.34	1	72.58	20.23	64.29	14.29	0
2 x	82.95	63.73	85.71	20.23	72	4	4
2 x	98.16	2.75	77.17	20.23	44.44	0	0
2 x	1.97	99	15.38	96.74	68.75	0	0
2 x	1	40.06	99	20.23	100	0	0
2 x	63.17	8.2	71.27	20.23	51.52	4.55	1.52
2 x	4.38	99	17.84	20.23	56.52	0	0
2 x	97.04	40.06	54.55	83	41.67	2.08	0
2 x	69.05	1	63.35	20.23	50	10	0
2 x	43.72	6.34	97.35	98.08	55.93	5.08	0

2	x	99	40.06	77.17	20.23	44.44	0	0
2	x	78.46	6.03	6.75	38.04	55.56	4.94	0
2	x	10.19	1	52.89	1	62.96	3.7	0
2	x	34.72	1	98.67	20.23	56.52	8.7	0
2	x	15.41	12.41	35.01	20.23	60	0	0
2	x	1	98.75	1	20.23	50	0	0
2	x	12.74	1	33.61	20.23	42.11	5.26	0
2	x	99	1.71	89.39	20.23	75	0	0
2	x	89.52	3.95	63.35	1	60	0	0
2	x	1	2.75	1	20.23	88.89	11.11	0
2	x	15.41	40.06	63.35	20.23	60	0	0
2	x	10.19	1	99	20.23	66.67	16.67	0
2	x	70.71	11.66	89.39	20.23	59.38	9.38	0
2	x	43.4	11.66	54.55	20.23	43.75	0	0
2	x	67.62	14.81	8.42	20.23	57.89	0	0
2	x	3.8	1	99	20.23	65	0	0
2	x	96.35	40.06	2.36	20.23	35.71	0	0
2	x	26.1	99	1	20.23	25	0	25
2	x	77.34	77.41	63.35	1	66.67	0	0
2	x	22.63	5.72	12.44	1.85	55.88	2.94	0
2	x	50.85	9.66	91.21	20.23	51.16	4.65	0
2	x	16.49	2.18	83.69	20.23	29.41	5.88	0
2	x	89.52	25.84	89.39	39.38	48.68	2.63	0
2	x	56.34	26.95	49.65	8.85	44.58	4.82	0
2	x	16.49	40.06	12.44	20.23	47.06	11.76	0
2	x	36.67	98.27	8.42	20.23	57.89	0	0
2	x	36.2	1	98.58	1.71	54.55	3.03	0
2	x	3	99	7.91	74.09	68.97	0	3.45
2	x	52.72	33.25	32.79	37.57	56.63	2.41	0
2	x	62.1	99	54.55	20.23	62.5	0	3.13
2	x	57.89	8.66	87.98	58.75	50.82	6.56	0.82
2	x	1.05	99	12.44	20.23	70.59	0	0
2	x	2.35	1	24.32	20.23	57.14	4.76	0
2	x	78.46	20.91	77.17	20.23	55.56	3.7	1.23
2	x	99	40.06	99	20.23	52.63	0	0
2	x	72.44	18.24	99	20.23	56.52	0	0
2	x	49.68	40.06	89.39	20.23	50	0	0
2	x	1.16	1	63.35	20.23	80	6.67	0
2	x	79.34	29.41	44.14	4.87	61.54	1.92	0
2	x	3.8	13.3	80.49	1	60	2.86	0
2	x	89.52	67.92	56.69	88.66	42.86	0	0
2	x	1	1	99	20.23	66.67	33.33	0
2	x	89.52	99	1	20.23	44.44	0	11.11
2	x	1	1	39.59	20.23	72.22	11.11	5.56
2	x	51.7	1.98	85.71	20.23	60	4	0
2	x	38.96	20.52	97.66	59.98	60.76	5.06	2.53
2	x	89.52	99	1	20.23	60	0	0
2	x	1	1	99	20.23	75	12.5	0
2	x	7.9	29.78	52.89	20.23	61.11	1.85	5.56
2	x	1	1	96.01	20.23	66.67	11.11	5.56

2 x	49.68	13.82	96.01	20.23	44.44	11.11	5.56
2 x	63.17	12.22	99	20.23	66.67	6.06	0
2 x	69.05	89.5	1	99	45	0	0
2 x	39.7	1	99	20.23	60	6.67	0
2 x	80.78	15.45	76.13	20.23	54.24	3.39	0
2 x	4.85	40.06	16.81	20.23	64.1	7.69	2.56
2 x	6.68	1	95.15	20.23	54.55	18.18	0
2 x	1	7.28	99	20.23	72	12	4
2 x	1	1	97.09	20.23	100	14.29	0
2 x	12.37	40.06	50.45	20.23	50	0	0
2 x	31.5	6.61	99	4.22	58.33	8.33	0
2 x	79.79	92.24	10.18	20.23	50	0	0
2 x	57.01	5.83	48.28	20.23	60.58	5.11	0
2 x	24.55	26.18	16.81	60.52	64.1	7.69	0
2 x	44.1	2.75	77.17	20.23	48.15	0	0
2 x	18.75	18.24	96.69	20.23	60.87	6.52	0
2 x	19.3	1.98	63.35	20.23	60	4	0
2 x	20.35	1	99	59.44	60	10	0
2 x	89.52	1	33.61	20.23	57.89	15.79	0
2 x	1	1	24.32	20.23	100	14.29	0
2 x	3.8	1	63.35	20.23	100	20	0
2 x	52.34	1.83	70.28	20.23	50.88	3.51	0
2 x	35.59	97.11	2.36	20.23	57.14	0	0
2 x	81.13	22.81	92.94	20.23	44.26	4.92	0
2 x	97.77	59.9	1	20.23	46.67	0	0
2 x	89.52	67.67	35.26	20.23	45.28	3.77	1.89
2 x	96.88	2.41	67.94	36.68	55.17	6.9	0
2 x	32.02	1.08	5.07	99	59.09	9.09	0
2 x	1	89.5	1	20.23	40	0	0
2 x	34.49	8.91	37.49	47.86	61.82	3.64	1.82
2 x	76.24	1	99	1	57.14	7.14	0
2 x	3.8	99	63.35	20.23	80	0	0
2 x	82.95	63.73	20.6	31.2	52	3.2	0
2 x	84.4	68.33	58.85	20.23	59.68	3.23	0
2 x	44.1	2.75	52.89	20.23	66.67	11.11	0
2 x	7.34	1	24.32	98.65	71.43	0	0
2 x	23.88	1	52.89	20.23	66.67	0	0
3 x	42.75	63.86	31.84	27.87	60.92	4.02	1.15
3 x	31.5	31.33	83.28	14.52	60.94	7.81	2.6
3 x	24.29	2.51	85.51	13.77	62.28	8.68	0.6
3 x	1	60.57	21.61	20.23	62.07	0	3.45
3 x	74.95	99	30.98	1	61.54	0	0
3 x	26.1	31.33	43.27	99	64.06	3.13	0
3 x	75.84	43.65	59.99	89.58	60.98	3.05	0
3 x	8.01	40.06	97.49	74.93	67.54	10.53	3.51
3 x	1	1	39.59	99	75	8.33	0
3 x	1.88	99	9.07	92.96	58.06	5.38	2.15
3 x	3.8	1.24	99	20.23	73.33	16.67	0
3 x	1.16	1	99	97.84	60	20	0
3 x	27.11	79.51	67.59	1.48	61.9	5.56	2.38

3 x	30.53	1.47	95.69	1.05	68.97	11.21	0
3 x	65.66	50.93	26.08	20.23	55.93	2.13	1.22
3 x	77.67	46.49	37.09	84.9	59.78	6.52	0
3 x	57.83	99	22.48	87.73	65.12	2.33	6.98
3 x	69.62	18.85	74.09	29.67	47.55	7.69	0
3 x	28.02	58.11	61.28	6.9	57.58	6.06	1.52
3 x	6.22	66.57	41.37	73.17	60.53	4.51	2.63
3 x	17.35	78.45	1.6	52.16	57.24	2.76	1.38
3 x	33.38	51.1	52.89	93.93	59.26	1.85	0
3 x	3.8	99	63.35	20.23	80	20	0
3 x	8.79	19.26	37.69	2.96	58.2	4.1	0
3 x	79.1	12.77	26.78	66.58	58.82	5.88	0
3 x	41.98	57.27	25.1	49.66	66.35	1.92	1.92
3 x	95.53	1	83.69	1	41.18	0	0
3 x	22.75	1	98.5	94.44	56.6	0	1.89
3 x	89.52	40.06	1	20.23	0	0	0
3 x	7.19	4.74	29.13	20.23	64.15	7.55	0
3 x	99	40.06	1	99	40	0	0
3 x	66.04	2.75	59.49	1	47.22	2.78	0
3 x	26.1	40.06	1	1	61.36	0	0
3 x	94.11	19.33	7.55	4.4	57.14	0	0
3 x	55.58	73.71	2.58	7.16	58.82	0	0
3 x	1	1	2.36	20.23	50	14.29	0
3 x	58.02	1	98.75	2.17	58.33	5.56	0
3 x	10.19	3.47	68.87	20.23	60.42	8.33	0
3 x	40.35	65.94	4.55	9.61	60.44	2.2	0
3 x	69.05	47.47	45.52	8.55	58.75	2.5	0
3 x	17.13	6.54	9.87	3.79	59.03	3.08	0.44
3 x	30.01	69.75	24.32	51.68	55.1	2.04	0
3 x	89.52	40.06	1	20.23	44.44	0	0
3 x	89.52	7.93	98.89	99	46.15	7.69	0
3 x	1	1	99	1	62.5	12.5	0
3 x	81.89	5.27	50.45	86.79	45.45	4.55	0
3 x	89.52	99	1	99	50	0	0
3 x	89.52	40.06	63.35	20.23	40	0	0
3 x	14.64	40.06	1	1	65.22	0	0
3 x	82.63	34.13	68.87	20.23	58.33	2.08	2.08
3 x	77.08	30.62	84.57	5.94	61.02	0	0
3 x	81.21	4.28	42.46	1	53.66	4.88	0
3 x	77.67	5.93	9.43	84.9	63.04	8.7	0
3 x	80.2	14.49	48.09	1	53.57	3.57	0
3 x	5.94	24.76	24.32	65.27	54.29	2.86	0
3 x	42.52	1	5.94	20.23	52.38	0	0
3 x	39.06	9.33	22.37	23.69	54.64	2.46	0.27
3 x	28.28	15.13	7.91	5.8	60.34	0	0
3 x	32.21	31.33	27.61	3.32	63.28	0.78	0
3 x	1.3	1	81.58	20.23	61.54	0	0
3 x	39.7	10.48	19.26	1	50	3.33	0
3 x	66.04	2.75	10.18	20.23	33.33	11.11	0
3 x	21.47	31.99	43.84	16.15	60.43	4.32	0

3	x	26.1	1.71	1	99	25	0	0
3	x	36.67	40.06	93.14	20.23	57.89	0	5.26
3	x	1.78	92.24	1	1	44.44	0	0
3	x	98	3.34	99	92.27	57.89	0	0
3	x	37.76	14.57	53.97	13.14	56	1.33	0.67
3	x	1.58	40.06	17.19	20.23	64.52	0	0
3	x	89.52	40.06	63.35	20.23	40	0	0
3	x	1	1.63	15.95	52.78	70.53	6.32	0
3	x	12.51	51.52	1.95	89.04	62.5	4.81	0.96
3	x	1	1.71	15.38	20.23	50	12.5	0
3	x	2.08	18.24	17.84	20.23	60.87	4.35	0
3	x	94.18	6.61	3.81	99	54.17	4.17	0
3	x	4.19	23.94	39.59	20.23	69.7	4.55	3.03
3	x	16.96	3.47	26.04	1	64.58	0	0
3	x	56.07	29.95	9.55	93.39	56.36	3.64	0
3	x	34.92	10.48	19.26	72.45	60	0	0
3	x	82.42	22.17	91.37	11.52	44.44	5.13	0
3	x	89.52	59.9	7.03	6.08	55	0	0
3	x	30.81	88.82	64.2	37.8	68.9	1.83	3.05
3	x	73.7	68.76	61.1	20.23	65.57	0	3.28
3	x	91.94	40.06	30.98	1	59.62	1.92	0
3	x	13.4	55.55	76.37	2.61	63.64	1.3	3.9
3	x	88.45	92.59	44.04	3.61	51.88	0	5.26
3	x	5.47	98.6	37.69	6.23	63.93	0	8.2
3	x	1.62	15.75	1	20.23	70	0	0
3	x	35.59	88.15	64.98	37.34	60.71	3.57	2.38
3	x	1	1.71	89.39	20.23	62.5	12.5	0
3	x	89.52	84.23	98.75	1	63.89	0	2.78
3	x	62.1	11.66	89.39	20.23	50	0	6.25
3	x	21.46	49.5	24.32	97.05	60.32	3.17	0
3	x	31.5	97.37	39.59	83	62.5	0	8.33
3	x	86.52	7.28	96.32	20.23	62	4	0
3	x	99	93.56	83.69	1	52.94	0	0
3	x	77.59	49.82	61.1	99	55.74	3.28	1.64
3	x	63.7	99	13.1	67.25	50.75	0	5.97
3	x	21.46	4.61	67.59	70.15	63.49	6.35	0
3	x	66.45	71.69	28.97	98.19	65.75	4.11	2.74
3	x	1.39	18.97	1	1	70.83	8.33	4.17
3	x	89.52	40.06	93.95	20.23	53.33	6.67	0
3	x	12.74	70.59	70.28	20.23	36.84	5.26	5.26
3	x	35.59	21.49	89.39	20.23	64.29	0	0
3	x	66.59	86.82	50.45	47.86	54.55	0	3.64
3	x	23.18	99	71.87	1	60.66	0	8.2
3	x	5.28	99	86.26	74.09	58.62	0	13.79
3	x	27.08	87.26	21.89	68.66	66.15	0	4.62
3	x	18.36	54.61	59.99	6.3	59.35	6.5	0.81
3	x	74.95	98.04	2.93	20.23	69.23	0	7.69
3	x	45.4	40.06	83.13	83.95	57.45	4.26	3.19
3	x	5.64	1	1	20.23	62.5	12.5	0
3	x	10.19	99	1	20.23	66.67	0	0

3 x	8.25	99	53.52	56.53	66.28	1.16	5.81
3 x	24.55	55.35	48.76	91.4	61.54	2.56	2.56
3 x	51.7	19.67	13.33	99	56	4	0
3 x	10.68	80.78	27.91	92.61	61.7	7.45	4.26
3 x	94.56	40.06	21.89	6.78	63.08	0	1.54
3 x	65.33	17.46	20.79	20.23	47.73	0	0
3 x	95.53	40.06	46.57	95.42	52.94	0	0
3 x	41.62	83.23	91.84	1.4	56.45	4.84	0
3 x	1	40.06	1.91	1	60	0	6.67
3 x	95.19	91.56	27.74	2.35	62.16	1.35	5.41
3 x	15.82	1.86	99	68.3	62.6	7.63	0
3 x	27.81	99	20.17	20.23	58.11	0	10.81
3 x	75.62	2.75	98.13	20.23	51.85	7.41	0
3 x	13.87	5.72	26.78	20.23	57.35	2.94	0
3 x	23.88	92.24	99	48.44	62.96	1.85	9.26
3 x	53.72	16.99	72.01	29.19	56	2.67	4
3 x	22.81	9.74	59.49	40.61	52.78	4.17	1.39
3 x	19.3	40.06	85.71	20.23	52	0	0
3 x	28.02	74.59	79.84	30.55	53.79	4.55	3.03
3 x	40.46	59	19.47	13.42	64.33	0	0.64
3 x	75.62	40.06	52.89	1	66.67	0	0
3 x	40.88	20	90.94	20.23	62.75	0	1.96
3 x	71.44	53.63	95.15	20.23	68.18	0	6.82
3 x	39.38	99	60.36	14.3	59.78	0.54	5.98
3 x	46.7	7.61	46.57	95.42	52.94	1.96	0
3 x	50.03	22.04	63.35	2.21	61.38	3.45	0
3 x	26.1	40.06	89.39	1	50	0	0
3 x	43.4	11.66	75.23	1	59.38	6.25	0
3 x	4.78	32.05	24.32	20.23	67.14	1.43	1.43
3 x	76.24	40.06	39.59	20.23	54.76	2.38	2.38
3 x	29.57	8.91	74.91	20.23	69.09	3.64	1.82
3 x	8.11	1.71	63.35	59.44	70	2.5	0
3 x	34.44	40.06	98.68	14	57.47	1.72	4.6
3 x	1	23.05	91.84	1	64.52	9.68	0
3 x	82.8	1	99	20.23	55.1	8.16	0
3 x	29.85	97.48	67.28	20.23	58.82	2.94	8.82
3 x	48.72	62.44	29.13	20.23	69.81	1.89	5.66
3 x	89.52	92.24	10.18	99	66.67	0	0
3 x	6.11	5.93	17.84	53.96	65.22	4.35	0
3 x	1	99	63.35	1	60	5	5
3 x	7.19	97.81	29.13	20.23	62.26	1.89	5.66
3 x	35.59	40.06	99	20.23	71.43	0	14.29
3 x	9.48	72.52	86.48	31.3	63.71	1.61	7.26
3 x	9.45	73.96	5.54	31.93	62.71	1.69	0
3 x	63.76	85.21	43.5	20.23	58.28	0.66	1.32
3 x	47.93	9.86	82.53	20.23	56.03	6.03	1.72
3 x	5.25	62.05	81.93	20.23	61.11	2.78	0.93
3 x	67.24	77.41	53.97	39.67	66	2	4.67
3 x	1.78	92.24	94.6	1.48	69.84	1.59	12.7
3 x	71.44	99	50.45	1	45.45	0	0

3 x	8.11	40.06	89.39	8.55	60	2.5	5
3 x	43.94	32.05	27.12	41.28	57.62	5.24	0.48
3 x	6.22	98.27	99	61.63	63.16	0	2.63
3 x	26.1	3.47	68.87	20.23	58.33	6.25	0
3 x	1	77.41	63.35	20.23	66.67	6.67	0
3 x	10.19	40.06	39.59	20.23	66.67	0	0
3 x	51.23	40.06	36.04	20.23	56.92	4.62	0
3 x	5.14	16.63	56.69	1	57.14	0	0
3 x	28.02	94.21	71.27	20.23	54.55	0	0
3 x	49.68	72.07	10.18	99	52.78	8.33	0
3 x	50.6	2.92	90.83	20.23	58.18	3.64	0
3 x	66.04	1	77.17	93.93	55.56	16.67	0
3 x	13.27	40.06	89.39	20.23	43.75	6.25	0
3 x	13.27	47.47	71.5	38.3	61.25	5	0
3 x	5.35	3.01	73.75	62.77	59.46	5.41	0
3 x	62.1	97.73	45.52	1	52.5	0	0
3 x	74.45	55.75	52.19	2.53	44.74	5.26	0
3 x	3.59	25.84	84.38	5.65	62.28	5.26	0.88
3 x	20.35	15.75	63.35	1	65	2.5	0
3 x	1	99	1	96.74	68.75	0	12.5
3 x	1.3	98.04	1	2.7	66.67	0	10.26
3 x	1	98.04	81.58	20.23	65.38	0	11.54
3 x	4.19	99	8.65	1	63.64	0	4.55
3 x	6.68	53.63	11.02	55.62	63.64	4.55	0
3 x	10.19	40.06	1	1	50	0	0
3 x	89.52	99	1	1	40	0	0
3 x	96.46	16.2	99	20.23	63.41	2.44	0
3 x	95.53	12.77	12.44	20.23	52.94	0	0
3 x	89.52	3.95	63.35	99	60	0	0
3 x	18.87	26.18	57.98	1	60.26	2.56	0
3 x	18.12	3.12	76.73	7.42	64.29	6.43	0
3 x	7.69	23.73	30.98	6.78	63.08	7.69	0
3 x	1.97	54.96	63.35	38.3	67.5	5	1.25
3 x	55.58	24.36	46.57	1.85	60.29	2.94	0
3 x	7.78	2.18	97.97	1.85	55.88	8.82	0
3 x	58.5	4.22	90.94	20.23	50.98	7.84	0
3 x	1	2.75	59.49	93.93	58.33	8.33	0
3 x	84.79	4.45	86.1	13.74	56.02	6.63	0
3 x	63.92	6.34	35.69	1	55.93	4.24	1.69
3 x	1	1	98.38	1	80	0	0
3 x	1	1	99	20.23	60	10	0
3 x	13.27	99	15.38	20.23	43.75	0	0
3 x	32.54	56.87	31.69	2.07	63.38	0	4.23
3 x	10.94	80.49	55.28	58.75	64.75	1.64	2.46
3 x	6.68	2.92	37.49	47.86	67.27	3.64	1.82
3 x	31.41	18.14	78.16	3.84	61.75	2.19	1.64
3 x	4.05	18.97	94.8	20.23	64.58	6.25	0
3 x	1.5	1	93.44	1	68.6	7.44	0
3 x	1	99	17.84	1	60.87	0	0
3 x	10.19	98.75	1	20.23	50	0	0

3 x	71.09	11.94	73.25	43.1	53.85	1.54	0
3 x	51.14	43.46	32.43	46.42	47.4	1.73	0
3 x	56.86	6.73	88.76	42.52	60.9	6.02	0
3 x	84.53	64.91	5.84	92.27	57.89	3.16	0
3 x	55.17	7.73	80.56	41.8	62.29	7.54	0
3 x	97.77	3.95	63.35	99	50	10	0
3 x	61.21	7.53	76.87	4.68	57.14	6.4	0
3 x	49.42	40.06	80.42	26.91	52.79	3.05	2.54
3 x	10.19	99	3.81	20.23	50	0	0
3 x	82.95	98.38	13.33	20.23	58	0	0
3 x	22.98	40.06	1	20.23	68.42	5.26	0
3 x	3.27	96.39	50.45	20.23	59.09	0	0
3 x	64.84	93.13	57.98	20.23	53.85	0	0
3 x	39.7	99	1	20.23	60	0	0
3 x	47.93	1.94	99	20.23	56.9	5.17	0
3 x	39.7	89.5	1	20.23	50	0	0
3 x	10.19	40.06	99	20.23	55.56	11.11	0
3 x	1	1	99	20.23	57.14	14.29	0
3 x	84.31	40.06	61.1	99	63.93	3.28	3.28
3 x	16.54	29.55	62.7	14.98	55.92	2.84	0
3 x	9.2	92.77	5.07	86.79	63.64	4.55	0
3 x	33.98	19.67	7.03	20.23	58	2	0
3 x	63.17	5.27	95.15	67.94	69.7	3.03	0
3 x	58.3	46.86	75.03	20.23	47.13	2.3	0
3 x	66.04	40.06	63.35	54.76	53.33	0	0
3 x	81.31	56.52	43.66	40.45	55.17	0.69	3.45
3 x	89.52	96.04	19.26	20.23	60	0	0
3 x	39.7	10.48	98.38	20.23	53.33	0	0
3 x	80.69	26.18	81.58	99	53.85	2.56	0
3 x	55.25	1	99	20.23	55.56	7.41	0
3 x	15.41	3.95	99	99	60	10	0
3 x	41.98	62.84	81.58	79.29	38.46	3.85	0
3 x	3.27	1	79.84	3.56	65.91	9.09	0
3 x	39.7	3.95	99	20.23	70	0	0
3 x	1.61	66.94	40.49	20.23	68.7	5.34	0
3 x	1	99	1.55	20.23	75.51	2.04	10.2
3 x	1.78	54.78	99	58.91	60.49	2.47	1.23
3 x	62.1	23.51	96.5	96.74	62.5	9.38	0
3 x	66.04	87.35	10.18	77.5	61.11	1.85	0
3 x	7.5	96.04	19.26	85.84	53.33	2.22	0
3 x	3.8	22.56	39.59	72.45	66.67	3.33	0
3 x	1	1	99	1	61.54	7.69	0
3 x	46.31	6.97	93.95	85.84	60.74	3.7	0
3 x	20.83	99	1	20.23	72.73	0	0
3 x	1	24.76	80.49	1.98	57.14	8.57	0
3 x	1	1	63.35	20.23	60	20	0
3 x	1.62	99	19.26	1	71.67	1.67	5
3 x	46.07	79.51	24.32	20.23	71.43	7.14	0
3 x	11.53	62.64	63.35	33.54	61.9	1.9	1.9
3 x	39.7	57.11	91.58	20.23	65.71	0	2.86

3 x	53.56	26.18	99	60.52	58.97	2.56	0
3 x	3.55	28.37	12.21	92.61	70.21	6.38	1.06
3 x	18.12	40.06	97.09	88.66	64.29	4.76	0
3 x	2.82	80.84	78.08	86.16	68.66	4.48	5.97
3 x	10.19	98.04	91.37	91.4	69.23	2.56	5.13
3 x	11.36	54.96	89.39	2.86	60	7.5	0
3 x	28.77	79.09	70.75	1	59.15	1.41	0
3 x	4.26	61.28	89.39	1	46.43	0	0
3 x	17.22	59.25	91.84	97.31	64.52	6.45	0
3 x	5.88	45.56	84.54	71.34	67.44	6.98	1.4
3 x	6.28	1	75.03	56.07	62.07	8.05	0
3 x	45.12	5.27	99	99	63.64	0	0
3 x	62.75	23.77	87.8	8.7	58.28	1.84	1.84
3 x	89.52	3.95	99	99	50	0	0
3 x	70.52	49.43	80.38	57.15	59.06	1.57	2.36
3 x	1.43	99	1.27	20.23	77.14	0	5.71
3 x	1	97.11	24.32	98.65	71.43	7.14	7.14
3 x	96.67	7.93	30.98	1	53.85	7.69	0
3 x	99	97.11	72.58	20.23	50	0	0
3 x	2.87	94.84	1	1	54.17	0	0
3 x	20.83	74.59	95.15	67.94	63.64	0	3.03
3 x	1	5.93	99	20.23	60.87	10.87	0
3 x	1	1	99	20.23	80	20	0
3 x	46.07	97.11	8.95	99	64.29	3.57	0
3 x	15.89	91.56	36.48	99	59.46	2.7	2.7
3 x	4.19	1.92	13.81	20.23	69.7	9.09	0
3 x	1	99	10.18	99	66.67	0	22.22
3 x	16.66	57.98	93.14	42.52	58.65	2.26	0
3 x	20.56	63.23	57.89	41.2	63.35	3.2	1.78
3 x	3.07	54.96	78.67	1	62.5	5	0
3 x	7.65	75.49	89.39	83	60.42	4.17	0
3 x	4.49	69.99	86.77	30.21	66.91	2.94	0
3 x	19.76	92.24	1	1	61.11	0	0
3 x	10.19	20.91	52.89	99	62.96	3.7	0
3 x	2.75	11.07	91.84	1.4	64.52	3.23	0
3 x	29.08	34.52	81.28	26.6	59.97	4.7	1.13
3 x	6.75	40.06	93.83	81.49	59.68	4.84	4.03
3 x	70.67	15.85	90.71	3.76	55.25	2.76	2.76
3 x	3.03	24.11	73.36	76.05	67.66	5.39	0
3 x	68.5	19.33	66.12	99	57.14	2.04	0
3 x	43.18	73.71	96.65	88.2	57.65	2.35	0
3 x	93.12	23.94	95.15	1.71	48.48	6.06	0
3 x	4.65	99	36.72	8.55	67.5	0	5
3 x	42.75	60.57	13.6	98.26	55.17	3.45	0
3 x	86.82	61.66	50.45	76.62	54.55	3.64	1.82
3 x	18.87	47.66	81.58	99	62.82	8.97	3.85
3 x	37.02	49.95	8.72	24.47	54.15	1.66	1.99
3 x	97.04	84.23	1	20.23	50	0	0
3 x	7.34	79.51	24.32	20.23	60.71	0	3.57
3 x	74.95	20.31	1	20.23	42.31	3.85	0

3	x	67.62	1.34	8.42	20.23	60.53	0	0
3	x	30.65	89.78	7.29	20.23	62.63	2.02	1.01
3	x	42.89	10.23	45.99	20.23	60.81	2.7	0
3	x	1.62	69.2	89.39	20.23	65	5	5
3	x	5.64	1	89.39	1	50	12.5	0
3	x	1.16	99	1.02	1	66.67	2.67	1.33
3	x	16.96	99	1.63	96.74	68.75	0	0
3	x	1	99	67.94	5.8	72.41	0	0
3	x	78.06	31.19	24.32	43.94	50.79	1.59	0
3	x	52.18	7.45	12.87	50.64	63.37	5.94	0
3	x	8.22	94.45	35.26	5.02	66.04	0	0.94
3	x	15.41	99	22.74	50.98	58	2	0
3	x	1	18.24	44.75	1	73.91	8.7	0
3	x	29.3	44.31	49.02	7.35	54.68	0.72	2.16
3	x	19.76	98.75	1.17	2.17	72.22	0	0
3	x	60.19	19.89	66.03	20.23	56.58	3.29	0
3	x	46.31	17.85	4.71	99	44.44	2.22	0
3	x	62.1	81	28.56	20.23	52.5	2.5	2.5
3	x	76.53	78.98	6.22	11.33	64.91	0	0
3	x	8.85	1	54.55	20.23	59.38	0	0
3	x	36.09	85.69	63.35	1	42.5	1.25	2.5
3	x	39.7	29.01	49.13	34.28	59	5	3
3	x	35.59	50.7	24.32	20.23	53.57	1.79	0
3	x	29.54	97.03	3.49	7.88	59.46	0.68	0
3	x	8.89	58.11	50.45	34.44	66.67	3.03	0
3	x	77.46	68.97	33.04	6.16	44.63	1.65	1.65
3	x	1	86.82	50.45	1	72.73	9.09	0
3	x	36.11	52.42	53.87	6.69	58.03	3.11	1.04
3	x	89.52	2.75	77.17	1	50	5.56	0
3	x	23.27	99	1	20.23	69.05	2.38	0
3	x	94.68	40.06	1	20.23	42.86	0	0
3	x	66.14	25.59	31.32	24.51	57.38	3.69	0.34
3	x	14.87	8.2	86.68	1.71	69.7	10.61	0
3	x	46.52	15	15.38	52.41	56.25	5.21	0
3	x	1	1	99	99	72.73	9.09	0
3	x	61.33	1	83.13	97.14	59.57	6.38	0
3	x	1	40.06	1	1	80	0	0
3	x	1	6.61	39.59	1	66.67	8.33	0
3	x	73.36	1	99	1	66.67	8.33	0
3	x	83.25	20.31	2.93	1	50	0	0
3	x	89.52	12.77	12.44	20.23	41.18	0	0
3	x	1	3.95	7.03	20.23	65	0	0
3	x	45.12	66.75	1	1	68.18	4.55	0
3	x	99	1.71	99	20.23	50	12.5	0
3	x	73.36	1	3.81	99	41.67	0	0
3	x	1	40.06	1	1	50	0	0
3	x	5.22	63.73	75.94	20.23	56	0	0
3	x	59.12	14.33	3.49	2.35	67.57	2.7	0
3	x	73.93	25.5	73.75	20.23	40.54	2.7	0
3	x	26.1	40.06	1	20.23	37.5	0	0

3	x	75.62	70.19	10.18	20.23	61.48	0	1.48
3	x	24.81	18.61	57.41	83.95	53.19	4.26	4.26
3	x	10.19	40.06	3.81	1	61.11	0	0
3	x	40.52	14.77	35.38	17.57	45.41	1.83	0.69
3	x	87.71	81	71.5	78.09	61.25	0	3.75
3	x	18.12	97.11	1	1	57.14	0	0
3	x	39.7	40.06	63.35	1	40	10	0
3	x	41.16	80.24	59.99	20.23	48.78	7.32	0
3	x	10.19	15.29	2.93	20.23	66.67	5.13	0
3	x	54.26	86.82	71.27	67.94	51.52	0	3.03
3	x	1	40.06	1	1	50	0	0
3	x	58.82	33.94	37.44	45.26	60.53	2.35	2.45
3	x	44.83	1	99	35.47	61.29	11.83	0
3	x	10.19	23.28	62.27	31.1	65.87	7.14	0
3	x	85.27	56.4	1.12	20.23	53.42	0	1.37
3	x	60.04	8.26	29.13	20.23	47.17	1.89	0
3	x	62.96	68.54	65.56	58.42	50.41	1.63	3.25
3	x	99	40.06	1	20.23	33.33	0	0
3	x	10.19	98.75	39.59	20.23	66.67	16.67	0
3	x	30.97	18.65	45.19	20.23	58.96	3.3	2.36
3	x	10.19	40.06	3.81	1	50	0	0
3	x	15.77	40.06	51.8	11.83	62.04	1.63	4.49
3	x	99	40.06	39.59	99	33.33	0	0
3	x	48.67	34.37	22.74	50.98	54	2	0
3	x	29.85	40.06	1	66.58	58.82	0	5.88
3	x	44.67	87.41	59.82	23.15	55.45	0.93	0.93
3	x	35.92	89.05	92.94	71.67	59.02	3.28	6.56
3	x	56.38	77.41	47.52	97.84	54.44	3.33	2.22
3	x	8.37	5.93	4.38	99	47.83	4.35	0
3	x	64.29	10.57	66.12	34.6	57.65	4.59	1.02
3	x	1	70.59	70.28	20.23	68.42	5.26	5.26
3	x	89.52	86.82	79.84	99	40.91	0	0
3	x	62.96	98.98	42.46	58.42	53.66	4.88	9.76
3	x	45.99	32.61	25.96	13.18	54.3	1.99	1.99
3	x	1	1	99	20.23	66.67	0	0
3	x	63.02	25.96	63.35	46.5	59.13	5.65	0.87
3	x	55.1	50.79	41.2	36.29	53.83	1.35	1.8
3	x	75.94	21.2	99	47.86	54.55	5.45	0
3	x	1.3	99	98.89	79.29	61.54	3.85	7.69
3	x	64.92	35.93	20.95	20.23	56.83	1.44	0.72
3	x	74.19	19.67	63.35	50.98	48	2	0
3	x	26.1	54.96	2.73	59.44	57.5	7.5	2.5
3	x	51.66	28.33	69.93	20.23	59.43	3.56	2.85
3	x	29.44	90.88	18.39	20.23	63.16	2.63	2.63
3	x	56.86	66.32	24.32	11.2	56.25	0.89	1.79
3	x	7.58	44.84	83.05	44.38	64.52	5.65	0
3	x	8.96	1	95.74	49.34	69.52	6.67	0
3	x	59.5	22.5	34.33	20.23	55.65	2.09	1.26
3	x	58.5	78.3	26.78	99	53.92	1.96	0.98
3	x	57.64	40.06	82.94	99	53.27	6.54	0

3	x	15.57	67.44	40.7	77.95	59.81	2.8	5.61
3	x	36.67	1.34	52.19	2.53	55.26	7.89	0
3	x	42.52	67.92	56.69	20.23	42.86	14.29	4.76
3	x	19.15	95.39	29.82	56.76	61.4	0.58	7.6
3	x	95.49	12.94	8.85	20.23	49.51	5.83	1.94
3	x	26.1	94.84	15.38	99	25	12.5	0
3	x	90.81	29.21	32.94	33.97	50.98	2.94	0.98
3	x	68.5	19.33	66.12	99	51.02	8.16	0
3	x	1.78	92.24	1	99	55.56	11.11	0
3	x	64.17	53.85	55.9	6.35	53.35	3.46	0.23
3	x	14.57	29.5	80.49	4.95	60.95	7.62	2.86
3	x	11.52	97.04	23.86	80.85	65.91	1.14	0.57
3	x	4.85	99	1.57	99	72.31	0	0
3	x	50.6	93.66	63.35	63.17	60	0	0
3	x	32.23	96.44	45.67	60.52	60.68	5.13	0
3	x	11.39	84.99	48.76	20.23	62.39	5.13	1.71
3	x	6	31.86	95.23	73.61	66.34	7.8	0
3	x	9.76	62.84	57.98	49.66	55.77	8.65	0
3	x	16.2	30.38	74.45	32.27	62.61	4.35	0
3	x	60.37	88.15	31.58	57.46	52.38	1.19	0
3	x	63.17	99	13.81	1	60.61	0	0
3	x	33.03	40.06	55.07	75.21	62.94	7.65	1.76
3	x	24.16	49.66	58.85	1	56.45	4.84	1.61
3	x	29.33	99	1	91.11	62.71	1.69	0
3	x	52.14	80.24	59.99	37.8	64.63	3.66	1.22
3	x	22.63	95.91	36.2	95.42	61.76	1.47	0
3	x	24.48	56.06	87.73	56.07	63.6	6.51	1.53
3	x	20.68	40.06	37.78	56.84	60.16	4.69	3.13
3	x	37.7	99	2.1	74.09	58.62	0	0
3	x	36.26	92.55	34.3	1	54.3	0.66	1.32
3	x	38.37	5.27	34.4	99	59.09	6.82	0
3	x	54.7	99	1	99	57.5	2.5	0
3	x	42.27	55.61	69.11	84.9	55.65	3.48	0.87
3	x	5.58	1	96.78	27.91	63.58	10.4	0
3	x	2.14	1.24	96.79	45.29	63.33	13.33	0
3	x	64.35	93.46	1	98.75	63.06	0	2.7
3	x	3.73	57.4	85.04	81.93	65.12	6.4	0
3	x	27.21	87.2	50.59	32.38	60.75	2.08	2.52
3	x	20.83	96.98	18.26	51.32	61.62	2.02	4.04
3	x	9.26	96.21	37.14	41.11	65.25	3.55	3.55
3	x	33.38	84.23	49.53	64	61.11	4.17	0
3	x	1	3.95	89.39	90.5	70	15	0
3	x	10.94	98.6	49.37	97.57	62.3	3.28	0
3	x	8.5	99	53.97	99	62.67	2.67	0
3	x	44.66	84.23	1	11.69	56.67	1.67	0
3	x	69.92	11.07	79.1	99	45.16	6.45	0
3	x	9.13	1	99	20.23	68.29	9.76	0
3	x	21.69	52.89	98.45	84.42	59.14	7.53	1.08
3	x	15.07	99	95.3	10.8	64.15	2.83	0
3	x	36.92	98.45	35.88	89.28	59.68	1.61	0

3	x	15.72	99	22.93	46.76	57.89	3.51	0
3	x	15.64	99	17.57	91.84	61.04	0	1.3
3	x	13.06	93.56	9.16	99	55.88	1.96	0
3	x	43.49	16.02	96.43	85.74	57.14	9.36	0.49
3	x	10.19	99	1.51	96.12	63.64	0	0
3	x	73.36	99	15.38	99	50	0	0

you	conj	negate	cognitive p	insight	cause	discrepanc	tentative	certitude
2.99	4.48	4.48	17.91	2.99	1.49	0	5.97	1.49
10.53	10.53	5.26	15.79	0	0	10.53	5.26	0
0	11.76	2.94	8.82	2.94	0	0	2.94	0
0	5.33	2.67	26.67	6.67	1.33	4	6.67	2.67
2.7	6.76	1.35	10.81	5.41	0	1.35	2.7	1.35
2.63	2.63	0	13.16	0	0	2.63	5.26	2.63
0	13.16	1.32	9.21	2.63	1.32	0	0	1.32
2.44	4.88	0	14.63	2.44	0	2.44	2.44	0
0	7.27	3.64	10.91	0	1.82	1.82	0	0
5.77	7.69	0.96	16.35	4.81	0.96	1.92	5.77	1.92
0	12.5	3.13	6.25	0	0	0	0	6.25
0	5.26	0	0	0	0	0	0	0
0	7.83	0	13.91	0.87	2.61	2.61	0.87	1.74
0	0	0	0	0	0	0	0	0
12.5	0	0	12.5	0	0	12.5	12.5	0
11.11	11.11	0	3.7	0	0	3.7	3.7	0
0	8.33	8.33	25	8.33	8.33	0	0	0
6.25	6.25	0	25	0	6.25	6.25	6.25	0
8.33	0	0	16.67	0	0	8.33	8.33	8.33
1.83	2.75	0	12.84	4.59	1.83	2.75	1.83	0.92
0	6.25	6.25	31.25	0	0	0	6.25	12.5
12.5	12.5	0	0	0	0	0	0	0
7.14	7.14	0	0	0	0	0	0	0
0	4.92	4.92	11.48	0	0	3.28	0	1.64
0	3.92	0	7.84	1.96	1.96	0	0	1.96
0	5.88	2.94	2.94	0	2.94	0	0	0
0	6	4	14	0	4	8	2	0
0	7.32	0	17.07	3.66	3.66	1.22	4.88	2.44
2.33	4.65	4.65	13.95	2.33	2.33	9.3	2.33	0
11.76	2.94	0	14.71	0	0	0	0	2.94
0	4.11	4.11	16.44	1.37	4.11	6.85	1.37	0
0	11.38	1.63	9.76	1.63	0.81	2.44	0	0
5	10	5	25	5	0	0	5	0
0	9.8	5.88	15.69	5.88	0	1.96	1.96	0
0.88	8.77	3.51	16.67	7.02	1.75	1.75	0.88	0.88
1.08	6.45	1.08	7.53	1.08	1.08	1.08	0	1.08
0	14.29	0	28.57	0	0	0	0	14.29
0	5.56	2.78	8.33	0	0	2.78	0	0
0	4.55	0	9.09	4.55	0	4.55	4.55	0
0	4.7	1.34	13.42	2.01	4.03	3.36	3.36	0.67
4.32	7.03	3.78	14.05	2.16	3.24	3.24	2.16	1.08
5.45	14.55	0	18.18	1.82	1.82	1.82	7.27	0
4.17	8.33	1.28	16.03	1.92	2.88	3.53	3.85	0.96
2.7	5.41	0	10.81	1.35	1.35	5.41	1.35	0
6.15	6.15	1.54	26.15	4.62	6.15	6.15	1.54	0
5.95	8.33	5.95	21.43	3.57	5.95	3.57	4.76	0
6	6	4	14	4	2	0	0	4
0	1.92	0	11.54	1.92	5.77	3.85	1.92	0
4.41	5.88	1.47	13.24	5.88	0	1.47	0	0

3.3	10.99	0	18.68	4.4	2.2	5.49	1.1	2.2
4.48	8.96	2.99	25.37	2.99	5.97	4.48	5.97	4.48
5.84	11.68	0.73	14.6	3.65	2.19	1.46	5.84	0
0	3.33	0	16.67	0	0	6.67	6.67	0
7.35	5.88	0	14.71	4.41	2.94	4.41	0	0
9.89	9.89	1.1	24.18	4.4	3.3	8.79	2.2	0
10.81	1.35	1.35	13.51	5.41	0	4.05	1.35	0
2.5	6.25	2.5	23.75	6.25	0	3.75	3.75	2.5
7.63	10.17	3.39	24.58	2.54	5.08	6.78	3.39	0
1.3	11.69	3.9	18.18	3.9	2.6	1.3	5.19	0
0	15.15	3.03	21.21	6.06	0	3.03	3.03	0
1.69	5.08	5.08	13.56	3.39	1.69	1.69	0	3.39
0	11.43	2.86	25.71	4.29	5.71	7.14	5.71	0
8.33	8.33	0	12.5	0	0	8.33	4.17	0
2.27	2.27	2.27	9.09	2.27	0	4.55	0	0
0	6.76	2.7	14.86	2.7	1.35	4.05	2.7	2.7
7.81	4.69	4.69	17.19	1.56	0	9.38	1.56	3.13
5.3	9.85	2.27	18.94	3.03	2.27	5.3	3.03	0.76
5.17	8.62	3.45	24.14	3.45	3.45	3.45	1.72	1.72
3.7	11.11	0	3.7	0	0	0	0	0
7.69	7.69	2.56	15.38	2.56	2.56	5.13	2.56	0
2.33	6.98	4.65	16.28	0	2.33	6.98	2.33	0
4.72	4.72	0.94	20.75	1.89	1.89	6.6	3.77	2.83
8.06	6.45	3.23	14.52	0	6.45	3.23	3.23	0
4.22	4.22	3.01	19.88	4.82	5.42	3.61	4.22	1.2
7.83	9.57	1.74	16.52	1.74	2.61	3.48	4.35	0.87
14.29	4.76	2.38	16.67	7.14	0	9.52	0	0
6.02	7.23	3.01	18.07	1.81	4.22	4.22	4.22	0.6
6.54	7.19	2.61	16.99	3.92	1.96	4.58	3.27	0
5.22	5.97	0.75	14.18	3.73	2.24	2.99	0.75	0.75
11.59	4.35	10.14	11.59	0	1.45	2.9	0	2.9
1.72	4.31	1.72	18.1	3.45	2.59	1.72	0.86	2.59
5.56	1.85	0	11.11	3.7	1.85	3.7	0	1.85
5.26	5.26	0	10.53	5.26	5.26	5.26	0	0
5.77	6.73	0.96	17.31	0.96	0.96	6.73	4.81	0.96
2.42	10.48	3.23	16.94	0.81	0	0.81	5.65	0.81
10.42	7.29	4.17	17.71	3.13	2.08	4.17	1.04	1.04
8	0	0	12	8	4	0	0	4
10.24	8.66	1.57	17.32	2.36	2.36	5.51	7.09	0
5.56	4.86	1.39	11.11	2.78	2.78	1.39	0	1.39
2.44	9.76	2.44	12.2	1.22	3.66	2.44	0	0
8.54	6.1	3.66	24.39	3.66	2.44	3.66	3.66	3.66
3.57	5.36	7.14	16.07	0	3.57	3.57	0	1.79
1.45	2.9	4.35	14.49	1.45	1.45	5.8	1.45	0
3.01	9.02	2.26	19.55	3.01	0.75	6.02	2.26	3.01
0.87	6.09	2.61	13.91	2.61	2.61	2.61	3.48	5.22
0	11.48	3.28	6.56	1.64	0	0	0	1.64
0	9.09	0	9.09	0	0	0	0	9.09
7.14	0	0	7.14	0	0	7.14	0	0
3.13	6.25	0	12.5	3.13	3.13	3.13	0	0

0	16.28	0	11.63	0	0	2.33	6.98	2.33
3.13	6.25	3.13	15.63	6.25	3.13	1.56	3.13	0
0	6.86	3.92	12.75	3.92	3.92	1.96	1.96	0
2.96	7.88	3.94	11.33	2.46	3.45	1.48	0.99	0
0	6.25	4.17	14.58	4.17	0	2.08	0	0
0	9.46	2.7	13.51	4.05	0	2.7	4.05	1.35
4.11	6.85	1.37	17.81	8.22	2.74	2.74	2.74	0
0	8.2	4.92	18.03	3.28	1.64	3.28	1.64	1.64
1.86	6.05	1.4	15.81	3.72	1.86	1.86	3.26	0.47
3.85	2.56	2.56	16.67	3.85	2.56	6.41	1.28	0
0.88	8.79	1.54	11.21	2.86	1.1	1.1	2.86	0.22
4.35	4.35	0	4.35	0	0	0	0	4.35
5.56	0	0	0	0	0	0	0	0
2.78	5.56	5.56	25	2.78	2.78	0	8.33	2.78
0	10.26	2.56	17.95	5.13	0	2.56	5.13	0
0	0	25	25	0	0	0	0	0
0	3.23	6.45	19.35	0	0	3.23	9.68	3.23
4.35	13.04	0	21.74	2.17	0	8.7	6.52	0
12.5	3.13	0	18.75	3.13	0	12.5	0	6.25
3.7	11.11	3.7	33.33	11.11	3.7	7.41	14.81	0
4.44	12.22	3.33	18.89	3.33	2.22	3.33	5.56	0
4.69	3.13	1.56	9.38	0	1.56	0	4.69	1.56
3.45	0	0	10.34	3.45	0	6.9	0	0
0	4	0	8	0	0	4	0	0
4.55	9.09	0	11.36	2.27	2.27	2.27	0	4.55
3.57	3.57	0	7.14	0	0	0	3.57	3.57
7.02	8.77	0	12.28	3.51	1.75	3.51	1.75	1.75
5.26	0	5.26	21.05	0	0	0	5.26	10.53
0	6.42	0.92	8.26	1.83	2.75	3.67	0	0
2.94	8.82	0	8.82	0	0	0	0	2.94
8.33	0	0	16.67	4.17	0	0	0	4.17
1.69	3.39	3.39	15.25	3.39	1.69	0	1.69	1.69
7.69	11.54	0	5.77	1.92	0	0	0	0
2.97	9.9	2.97	17.82	2.97	0.99	3.96	4.95	0.99
2.61	7.83	2.61	8.7	1.74	0	0.87	2.61	0
0	11.11	11.11	33.33	11.11	0	0	0	0
0	8.89	0	8.89	0	0	4.44	0	0
0	8.89	0	8.89	0	0	4.44	0	0
9.26	5.56	1.85	18.52	5.56	3.7	3.7	0	3.7
12.5	10	2.5	17.5	7.5	0	5	2.5	0
1.03	4.12	0	15.46	4.12	3.09	3.09	2.06	2.06
2.08	10.42	2.08	10.42	0	0	6.25	2.08	4.17
2.27	6.82	4.55	25	4.55	2.27	2.27	2.27	2.27
4.26	4.26	2.13	8.51	2.13	0	0	0	4.26
4.08	4.08	4.08	20.41	6.12	0	6.12	4.08	2.04
0	9.52	4.76	9.52	0	0	0	4.76	0
6.25	6.25	3.13	18.75	3.13	0	3.13	3.13	0
0	5.26	0	10.53	0	0	5.26	0	5.26
0	7.14	2.38	4.76	1.19	1.19	0	0	1.19
2.7	5.41	0	10.81	2.7	0	0	8.11	0

6.06	12.12	3.03	18.18	0	0	3.03	6.06	3.03
7.89	7.89	2.63	15.79	2.63	0	10.53	7.89	2.63
3.57	8.93	0	19.64	3.57	0	3.57	3.57	5.36
1.32	9.27	3.31	17.22	3.31	1.32	1.99	1.99	2.65
6.67	13.33	0	20	6.67	0	6.67	0	6.67
4.17	11.67	0	15	1.67	0.83	5	5	0
1.11	10	1.11	14.44	1.11	2.22	5.56	2.22	1.11
5.56	11.11	0	5.56	0	0	0	0	2.78
5.45	7.27	1.82	16.36	3.64	1.82	10.91	1.82	3.64
0	6.67	0	20	0	0	13.33	0	20
0	5.88	0	17.65	2.94	2.94	5.88	5.88	0
12.5	0	0	18.75	6.25	6.25	6.25	12.5	0
0	8.33	0	6.25	0	0	4.17	2.08	0
6.67	6.67	6.67	13.33	6.67	0	6.67	0	0
7.14	14.29	0	7.14	0	0	0	7.14	0
10.53	0	0	15.79	5.26	0	10.53	0	10.53
5.71	11.43	0	11.43	2.86	0	2.86	2.86	2.86
8	8	0	8	0	0	0	0	4
3.7	14.81	3.7	25.93	7.41	0	3.7	14.81	0
2.42	10.63	0.97	15.46	3.38	2.42	2.9	5.8	0.48
12.5	0	0	12.5	0	0	12.5	12.5	0
0	6.67	0	6.67	0	6.67	0	0	0
7.14	14.29	0	7.14	0	0	0	0	0
0	11.11	3.7	22.22	0	0	0	14.81	3.7
5.08	5.08	1.69	6.78	0	0	5.08	1.69	3.39
0	6.67	0	10	3.33	0	3.33	3.33	0
0	0	0	20	0	0	0	0	0
5.8	5.8	1.45	13.04	4.35	2.9	2.9	0	2.9
9.09	7.27	0	12.73	3.64	1.82	5.45	3.64	3.64
0.65	5.19	2.6	16.23	2.6	3.9	3.9	2.6	0
10.53	5.26	2.63	7.89	2.63	2.63	0	2.63	0
0	12.5	0	12.5	0	0	0	0	0
5.88	0	0	11.76	0	0	5.88	5.88	5.88
5.48	8.22	1.37	15.07	4.11	0	2.74	2.74	1.37
0	15	5	25	0	0	0	20	0
11.11	0	0	22.22	11.11	0	11.11	11.11	0
12.5	6.25	0	12.5	6.25	0	6.25	6.25	0
10	5	0	10	5	0	2.5	0	2.5
0	11.11	11.11	22.22	0	0	0	0	11.11
4.55	9.09	2.27	13.64	2.27	2.27	0	6.82	2.27
0	2.6	0	7.79	1.3	1.3	0	0	2.6
1.96	9.8	0	13.73	3.92	0	0	7.84	0
3.7	9.26	0	16.67	3.7	0	3.7	3.7	0
0	8.82	0	8.82	0	0	0	5.88	0
0	3.7	0	3.7	3.7	0	0	0	0
8	12	0	16	8	0	4	0	4
0	6.76	0	16.22	6.76	0	2.7	4.05	1.35
0	12.5	0	12.5	0	6.25	0	0	6.25
11.11	11.11	0	0	0	0	0	0	0
6.67	10	3.33	10	3.33	0	3.33	0	3.33

8.45	5.63	0	15.49	2.82	0	5.63	2.82	2.82
0	0	0	22.22	0	0	11.11	0	11.11
7.69	0	0	7.69	7.69	7.69	0	0	0
4	6	2	8	2	0	0	0	0
7.06	14.12	3.53	16.47	1.18	1.18	5.88	2.35	0
0	4.17	0	12.5	0	0	4.17	4.17	0
3.77	6.6	2.83	17.92	2.83	3.77	2.83	4.72	0.94
9.38	4.69	4.69	17.19	0	0	4.69	4.69	4.69
2.78	5.56	1.39	9.72	1.39	0	0	1.39	2.78
2.78	5.56	1.39	9.72	1.39	0	0	1.39	2.78
9.26	11.11	1.85	18.52	1.85	1.85	5.56	3.7	1.85
5.81	6.98	0	15.12	4.65	1.16	2.33	8.14	0
4.47	8.13	2.85	21.54	4.07	0.81	3.66	6.1	2.44
0	9.62	1.92	23.08	1.92	1.92	1.92	11.54	1.92
1.64	6.56	3.28	9.84	1.64	0	3.28	1.64	0
4.37	10.32	1.59	19.05	2.78	2.38	3.57	5.95	1.98
0	10	0	30	0	0	0	30	0
0	6.67	0	6.67	0	6.67	0	0	0
2.13	6.38	0	14.89	2.13	0	4.26	0	4.26
5.88	5.88	0	8.82	2.94	0	0	5.88	0
1.59	4.76	3.17	6.35	0	0	1.59	1.59	0
0	0	16.67	25	8.33	0	8.33	0	0
7.69	7.69	3.85	19.23	3.85	0	7.69	3.85	11.54
1.45	9.18	1.45	14.01	1.93	0.48	2.42	2.42	0
0	6.25	1.56	9.38	0	0	1.56	3.13	0
7.69	13.85	0	16.92	4.62	3.08	4.62	4.62	0
6.45	6.45	3.23	12.9	3.23	0	9.68	3.23	0
6.12	6.12	4.08	12.24	2.04	2.04	4.08	0	4.08
6.62	7.28	1.99	16.56	1.99	0.66	5.3	4.64	0
4.35	10.87	2.17	15.22	8.7	0	4.35	0	4.35
3.23	6.45	3.23	9.68	3.23	0	0	3.23	3.23
3.13	8.59	0.78	16.41	1.56	0.78	3.91	5.47	0
2.26	7.52	0.75	11.65	3.01	1.13	1.13	2.63	0.75
1.75	5.26	2.63	14.91	1.75	4.39	5.26	0.88	0
4.82	7.83	0.6	17.47	4.82	2.41	3.01	3.01	1.81
2.78	11.11	0	12.5	0	0	8.33	2.78	0
13.33	0	0	6.67	6.67	0	0	0	0
10	3.33	0	10	3.33	6.67	3.33	3.33	0
2.22	4.44	0	20	4.44	0	4.44	4.44	4.44
3.39	10.17	0	8.47	0	1.69	3.39	1.69	1.69
4	4	0	12	0	0	4	0	4
3.03	6.06	3.03	6.06	0	0	3.03	3.03	0
2.38	9.52	0	0	0	0	0	0	0
1.41	8.45	0	14.08	1.41	2.82	2.82	2.82	2.82
0	12.31	3.08	12.31	1.54	0	1.54	1.54	3.08
0	5.71	2.86	5.71	0	0	0	0	2.86
0	4.84	3.23	11.29	1.61	3.23	4.84	0	0
6.06	12.12	0	21.21	3.03	3.03	3.03	9.09	0
2.63	7.89	0	18.42	2.63	2.63	7.89	5.26	0
0	17.24	0	6.9	0	0	0	0	6.9

0	6.49	1.3	14.29	2.6	2.6	2.6	3.9	0
0	6.52	2.17	13.04	2.17	0	0	2.17	4.35
0	5.8	0	4.35	0	0	0	0	1.45
0	6.67	0	13.33	6.67	0	0	0	0
0	5.13	0	12.82	2.56	0	2.56	2.56	0
1.38	4.61	2.3	6.45	0.46	0	1.84	1.38	0.46
0.66	5.3	3.31	16.56	4.64	1.99	1.99	2.65	0.66
1.21	4.84	1.61	22.58	8.06	2.82	2.82	2.82	3.23
0	12	0	12	4	4	0	0	0
0	4.76	2.38	14.29	2.38	0	0	4.76	0
0.67	9.33	2.67	18.67	4	1.33	3.33	4.67	1.33
0.72	5.76	2.16	6.47	0	0.72	1.44	1.44	0.72
4.88	7.32	0	7.32	0	0	2.44	2.44	2.44
0	3.7	1.23	13.58	3.7	1.23	3.7	2.47	0
0	5.83	0.97	14.56	0.97	0.97	3.88	1.94	2.91
0	5.04	0	10.92	3.36	2.52	0.84	3.36	0
6	7	1	16	4	1	3	2	1
2.13	8.51	6.38	15.96	2.13	1.06	0	3.19	0
0	12.5	12.5	0	0	0	0	0	0
0	8.7	0	4.35	0	0	0	0	0
0.94	6.6	2.83	12.26	5.66	0.94	0	3.77	0.94
0	12.5	0	34.38	0	0	15.63	6.25	3.13
0	0	0	7.69	0	0	7.69	0	0
0	10.71	0	17.86	0	3.57	0	3.57	3.57
0	5.56	5.56	27.78	5.56	5.56	5.56	5.56	0
0	9.3	2.33	6.98	0	0	0	0	4.65
0	8.82	0	2.94	0	2.94	0	0	0
4.55	0	0	9.09	0	0	9.09	0	0
1.89	6.6	0.94	15.09	6.6	2.83	0	3.77	0.94
0	0	0	10.71	0	0	0	7.14	0
5.56	5.56	0	11.11	0	0	5.56	5.56	0
0	5.06	2.25	11.24	1.12	1.69	3.37	2.25	0
5	13.33	1.67	23.33	0	1.67	6.67	10	3.33
4.17	4.17	0	25	8.33	4.17	0	0	4.17
6.04	7.38	2.68	17.45	2.68	1.34	2.68	1.34	1.34
3.13	10.23	0.85	15.34	3.98	1.99	2.56	1.7	1.42
1.54	4.62	3.08	13.85	3.08	0	1.54	4.62	0
4.41	11.76	2.21	10.29	2.94	0.74	0	1.47	0
0	0	3.85	3.85	0	0	0	0	0
2.38	7.14	1.19	14.29	4.76	4.76	2.38	2.38	0
2.7	5.41	1.35	12.16	4.05	0	2.7	6.76	0
5.17	10.34	3.45	22.41	3.45	3.45	5.17	1.72	0
4.17	8.33	0	9.72	2.78	2.78	2.78	0	0
0	6.06	0	12.12	3.03	3.03	3.03	6.06	0
0	12.87	1.17	22.22	2.34	1.17	5.26	7.6	2.92
0.69	6.9	1.38	16.55	5.52	1.38	4.14	2.07	0.69
0	7.14	0	23.81	7.14	0	0	7.14	4.76
0	3.6	3.6	9.91	1.8	0.9	2.7	0.9	0
0	8.33	0	8.33	0	0	4.17	0	4.17
6.41	10.26	2.56	19.23	1.28	2.56	2.56	5.13	0

11.11	5.56	5.56	11.11	0	5.56	0	0	0
6.06	6.06	3.03	21.21	9.09	3.03	0	0	6.06
2.44	4.88	0	12.2	2.44	2.44	2.44	2.44	0
2.9	11.59	2.9	2.9	0	1.45	1.45	0	0
0	0	0	0	0	0	0	0	0
3.45	10.34	2.3	10.34	3.45	1.15	1.15	1.15	1.15
6.58	14.47	1.32	10.53	2.63	0	2.63	0	1.32
0	0	0	7.14	0	7.14	0	0	0
4.62	6.15	1.54	13.85	1.54	1.54	1.54	4.62	0
5.41	0	2.7	2.7	0	0	0	2.7	0
3.23	0	0	16.13	3.23	3.23	0	6.45	6.45
0	3.92	0	16.67	3.92	2.94	2.94	4.9	0
7.69	4.4	4.4	13.19	1.1	1.1	2.2	1.1	0
2.94	10.29	1.47	14.71	2.94	0	1.47	2.94	1.47
4.24	8.47	2.54	14.41	4.24	0	1.69	2.54	0
5	15	2.5	17.5	0	0	0	7.5	2.5
4.44	4.44	0	8.89	2.22	2.22	0	0	6.67
12.5	0	0	12.5	0	0	0	0	12.5
2.27	6.82	0	22.73	4.55	0	2.27	6.82	4.55
7.25	8.7	2.9	17.39	4.35	2.9	0	2.9	0
3.95	9.21	3.95	10.53	1.32	0	0	2.63	1.32
7.87	8.99	1.12	14.04	1.12	0	3.93	5.06	1.69
11.48	6.56	3.28	9.84	0	0	0	1.64	1.64
3.77	7.55	0	13.21	0	1.89	3.77	1.89	1.89
5.17	1.72	1.72	12.07	1.72	1.72	3.45	0	1.72
16.67	0	0	0	0	0	0	0	0
8	8	8	16	0	0	8	4	0
6.25	4.17	3.13	9.38	1.04	1.04	4.17	2.08	0
3.7	3.7	3.7	14.81	3.7	3.7	0	3.7	0
3.85	7.69	1.92	14.42	0.96	1.92	1.92	2.88	4.81
3.13	6.25	0	12.5	3.13	3.13	3.13	3.13	0
8.33	0	0	8.33	8.33	0	0	0	0
6.03	6.03	0.86	11.21	0	0	1.72	3.45	1.72
10.42	8.33	4.17	18.75	4.17	2.08	4.17	2.08	0
6.8	7.77	5.83	10.68	0	0	2.91	2.91	0
6.25	6.25	3.13	18.75	12.5	0	3.13	3.13	0
3.51	10.53	3.51	12.28	1.75	0	3.51	0	1.75
0	0	9.09	0	0	0	0	0	0
5.77	8.65	2.88	13.46	3.85	1.92	1.92	4.81	0.96
0	25	0	25	12.5	0	0	0	0
5	7.5	2.5	22.5	5	2.5	5	7.5	0
5.85	7.6	2.92	19.01	4.09	1.75	2.92	5.85	0.29
5.66	3.77	0	5.66	1.89	0	0	1.89	0
0	0	0	9.09	0	0	0	9.09	0
0	4.35	1.45	8.7	1.45	1.45	1.45	2.9	0
7.46	2.99	2.99	8.96	0	0	5.97	0	1.49
6.25	6.25	0	12.5	0	0	6.25	0	0
5.88	5.88	0	11.76	0	0	5.88	5.88	0
5.36	5.36	1.79	8.93	1.79	0	3.57	1.79	0
9.3	2.33	2.33	13.95	0	0	4.65	6.98	2.33

7.69	0	0	7.69	0	0	0	0	7.69
9.09	0	0	9.09	0	0	0	0	9.09
7.04	2.82	0	15.49	1.41	1.41	4.23	4.23	2.82
9.26	11.11	3.7	16.67	0	3.7	7.41	0	1.85
2.22	5.56	3.33	14.44	1.11	1.11	3.33	4.44	2.22
0	7.69	7.69	23.08	0	0	7.69	7.69	0
0	0	0	0	0	0	0	0	0
1.27	8.86	0	17.72	3.8	0	2.53	8.86	0
7.69	0	0	15.38	7.69	0	7.69	0	0
5.49	9.89	3.3	23.08	3.3	2.2	6.59	6.59	0
4.08	4.08	6.12	14.29	4.08	0	0	4.08	0
0	8.33	0	12.5	0	0	8.33	4.17	0
0	0	0	7.69	0	0	3.85	3.85	0
0	0	8.7	4.35	4.35	0	0	0	0
0	10	0	20	10	0	0	0	10
9.09	4.55	0	13.64	4.55	0	0	9.09	0
0	7.35	2.57	15.44	2.57	1.84	2.21	2.57	0.37
3.03	3.03	3.03	15.15	0	6.06	9.09	0	0
0	10	0	20	0	10	0	0	5
0	14.29	0	14.29	0	0	7.14	0	0
10	7.5	7.5	20	7.5	2.5	2.5	5	0
7.69	7.69	3.85	7.69	0	0	3.85	3.85	0
1.52	6.06	1.52	18.18	0	1.52	1.52	3.03	3.03
0	12.5	0	12.5	0	0	0	0	12.5
0	15.91	4.55	20.45	4.55	0	2.27	4.55	0
4.63	7.41	0.93	14.81	3.7	2.78	1.85	4.63	0
0	8.95	2.11	18.42	3.68	3.68	2.11	4.21	0
10	0	10	30	10	0	10	0	0
0	5	0	10	5	0	0	0	0
0	10	10	15	0	0	0	10	5
0	15.38	0	0	0	0	0	0	0
0	0	0	13.04	0	0	4.35	4.35	4.35
9.26	9.26	7.41	14.81	3.7	0	3.7	3.7	0
0	0	0	18.18	9.09	0	9.09	9.09	0
0	8.33	0	16.67	8.33	0	0	0	0
0	0	0	0	0	0	0	0	0
0	7.14	7.14	21.43	0	0	7.14	0	7.14
0	9.09	9.09	9.09	0	0	0	9.09	0
0	0	0	0	0	0	0	0	0
0	16.67	8.33	41.67	8.33	8.33	8.33	16.67	0
1.67	6.67	5	16.67	3.33	0	0	3.33	0
1.82	1.82	1.82	20	3.64	3.64	3.64	9.09	1.82
0	5.8	1.45	11.59	5.8	0	1.45	1.45	0
6.67	0	0	13.33	0	0	6.67	6.67	0
11.11	11.11	0	33.33	0	0	11.11	0	11.11
12.2	0	2.44	12.2	4.88	0	2.44	0	0
0	5.56	5.56	16.67	5.56	5.56	0	0	5.56
7.06	5.88	2.35	14.12	2.35	1.18	3.53	2.35	0
7.69	4.27	3.42	13.68	3.42	0	1.71	3.42	1.71
6.9	3.45	3.45	17.24	0	3.45	0	0	3.45

0	6.25	6.25	28.13	3.13	0	6.25	0	3.13
1.37	9.59	5.48	17.81	2.74	1.37	2.74	2.74	0
3.08	4.62	7.69	23.08	3.08	1.54	1.54	4.62	4.62
2.56	5.13	2.56	7.69	5.13	0	0	0	0
9.09	4.55	4.55	13.64	0	0	0	0	0
2.67	6	0.67	18	2.67	3.33	3.33	5.33	0
5.36	3.57	1.79	21.43	5.36	1.79	1.79	3.57	1.79
3.85	7.69	5.77	14.42	3.85	4.81	1.92	2.88	0
2.56	7.69	2.56	7.69	0	0	2.56	2.56	2.56
0	9.52	0	14.29	0	0	4.76	0	4.76
9.09	9.09	9.09	27.27	0	4.55	9.09	9.09	4.55
1.45	7.25	5.8	14.49	2.9	1.45	4.35	4.35	0
2.9	0	4.35	7.25	0	1.45	0	0	4.35
5.56	5.56	2.78	13.89	0	0	0	2.78	0
0	14	6	18	0	2	2	4	4
7.37	9.47	3.16	20	6.32	4.21	3.16	3.16	1.05
3.94	8.66	0.79	11.02	0	0.79	2.36	0.79	0.79
3.7	3.7	3.7	16.67	0	1.85	1.85	1.85	0
4.35	4.35	4.35	16.52	2.61	4.35	1.74	0.87	0.87
0	0	2.17	8.7	0	0	2.17	4.35	0
4	8	4	20	4	0	8	0	8
3.67	7.34	5.5	20.18	1.83	0	2.75	7.34	0
5.66	3.77	7.55	11.32	0	0	0	3.77	0
0	7.14	0	0	0	0	0	0	0
0	5	7.5	20	0	5	2.5	0	2.5
0	2.56	2.56	7.69	2.56	5.13	0	0	0
0	11.76	5.88	17.65	0	0	0	5.88	0
0	14.29	0	7.14	0	0	0	7.14	0
0	12.5	0	12.5	0	0	0	12.5	0
0	6.06	0	18.18	6.06	0	0	9.09	0
0	2.86	14.29	14.29	0	0	0	5.71	2.86
1.96	1.96	5.88	21.57	9.8	5.88	3.92	3.92	0
0	12.5	8.33	12.5	0	0	0	4.17	0
4.76	9.52	7.14	30.95	9.52	2.38	4.76	4.76	0
0	12.5	0	12.5	0	0	0	12.5	0
1.54	12.31	3.08	15.38	1.54	0	1.54	6.15	0
0	8.82	5.88	23.53	4.41	2.94	2.94	7.35	0
11.29	8.06	1.61	12.9	1.61	1.61	1.61	4.84	0
0	4.76	4.76	28.57	14.29	4.76	4.76	0	4.76
0	11.11	2.78	19.44	5.56	0	0	8.33	0
2.04	6.12	0	10.2	2.04	2.04	2.04	4.08	0
18.18	0	0	0	0	0	0	0	0
8.51	6.38	2.13	6.38	4.26	0	0	0	2.13
13.64	0	0	13.64	9.09	0	0	0	4.55
13.04	4.35	8.7	13.04	0	0	4.35	0	0
10.53	0	0	21.05	0	0	5.26	5.26	5.26
0	0	0	0	0	0	0	0	0
4	8	4	8	0	0	4	0	0
12.82	5.13	5.13	17.95	0	0	0	2.56	7.69
0	0	0	30	20	20	0	0	0

7.84	7.84	0	13.73	1.96	1.96	1.96	1.96	0
9.48	7.76	1.72	6.9	2.59	1.72	2.59	0.86	0
2.63	9.21	1.32	17.11	1.32	0	5.26	5.26	0
0	11.76	5.88	5.88	0	0	0	5.88	0
15.63	6.25	0	12.5	6.25	0	3.13	0	3.13
1.82	5.45	1.82	0	0	0	0	0	0
7.89	5.26	1.32	9.21	1.32	3.95	0	1.32	0
7.41	7.41	0	7.41	3.7	0	3.7	0	0
0.97	7.77	1.94	12.62	0	1.94	3.88	1.94	1.94
9.38	4.69	3.13	14.06	6.25	0	1.56	1.56	1.56
8.93	8.93	2.68	16.07	5.36	0.89	0.89	1.79	0.89
7.69	9.89	2.2	17.58	2.2	0	5.49	5.49	0
8.11	10.81	1.8	18.02	3.6	2.7	3.6	3.6	0
4.1	7.38	1.64	13.11	1.64	1.64	0.82	4.1	0
14.29	0	4.76	0	0	0	0	0	0
7.27	8.18	1.82	16.36	0.91	0.91	5.45	2.73	1.82
4.76	8.57	1.9	9.52	0.95	0	2.86	0.95	2.86
2.47	4.94	0	14.81	1.23	3.7	0	3.7	1.23
16.67	0	0	8.33	0	0	0	0	8.33
6.67	0	6.67	20	6.67	0	6.67	0	6.67
7.41	7.41	1.85	3.7	1.85	1.85	0	0	0
9.35	6.54	2.8	14.02	1.87	0.93	1.87	1.87	0.93
3.23	7.26	2.42	12.9	0.81	1.61	2.42	3.23	0.81
7.14	10.71	0	17.86	0	0	7.14	7.14	0
0.79	9.52	0.79	8.73	1.59	0	2.38	3.17	0
11.29	3.23	4.84	24.19	1.61	0	6.45	3.23	0
5.71	5.71	2.86	14.29	2.86	0	0	11.43	0
3.7	3.7	0	3.7	3.7	0	0	0	0
7.14	3.57	3.57	14.29	0	0	3.57	3.57	0
10.34	6.9	3.45	12.07	0	1.72	1.72	1.72	5.17
15.25	1.69	1.69	5.08	1.69	0	0	0	0
10	10	5	15	0	0	0	0	0
10.77	9.23	0	10.77	1.54	0	1.54	3.08	1.54
8.11	9.46	2.7	13.51	1.35	1.35	4.05	1.35	1.35
11.32	11.32	1.89	18.87	3.77	7.55	3.77	5.66	0
3.28	8.2	0	6.56	0	0	3.28	6.56	0
7.32	7.32	4.88	14.63	0	0	2.44	7.32	0
3.23	3.23	0	9.68	3.23	0	0	3.23	3.23
10	10	0	0	0	0	0	0	0
6.67	1.67	1.67	11.67	3.33	1.67	1.67	1.67	0
15.15	6.06	3.03	15.15	0	0	6.06	3.03	0
5.26	10.53	5.26	7.89	0	0	0	2.63	0
7.69	2.56	2.56	12.82	5.13	0	0	7.69	0
9.09	14.29	1.3	5.19	0	0	0	0	1.3
7.14	7.14	1.02	15.31	3.06	2.04	5.1	3.06	1.02
2.91	6.8	0	7.77	1.94	2.91	0	2.91	0
13.33	3.33	0	13.33	3.33	0	3.33	0	6.67
3	9	1	14	3	0	4	1	2
2.7	9.46	2.7	20.27	6.76	0	5.41	4.05	2.7
10.84	10.84	2.41	14.46	1.2	1.2	1.2	6.02	1.2

9.09	13.64	0	13.64	0	4.55	0	9.09	0
7.81	6.25	0	7.81	3.13	0	1.56	0	0
4.17	10.42	0	16.67	8.33	2.08	2.08	4.17	2.08
15.38	0	7.69	15.38	0	0	0	0	7.69
7.14	7.14	4.29	17.14	2.86	1.43	2.86	1.43	0
0	0	0	16.67	0	0	0	0	16.67
6.06	6.06	0	10.61	1.52	1.52	1.52	0	1.52
2.6	3.9	3.9	15.58	1.3	1.3	6.49	6.49	0
7.14	4.76	2.38	16.67	2.38	2.38	2.38	0	2.38
10.81	2.7	0	16.22	0	0	10.81	0	0
13.04	8.7	2.17	19.57	4.35	2.17	2.17	2.17	2.17
11.54	3.85	0	7.69	0	0	3.85	0	3.85
9.8	1.96	0	7.84	3.92	1.96	0	0	0
8	6	6	28	2	0	12	8	0
8.77	7.02	1.75	12.28	3.51	0	1.75	1.75	0
10.34	10.34	6.9	13.79	0	0	3.45	0	0
3.85	5.77	1.92	11.54	0	0	1.92	5.77	0
7.81	6.25	0	6.25	1.56	0	1.56	1.56	3.13
3.23	6.45	0	9.68	3.23	0	0	0	3.23
6.45	4.84	1.61	14.52	1.61	0	1.61	3.23	0
8	14	0	22	6	2	6	2	4
4.35	9.78	4.35	22.83	3.26	1.09	2.17	8.7	0
9.38	6.25	3.13	12.5	3.13	3.13	0	0	0
15.22	4.35	0	19.57	2.17	0	4.35	6.52	2.17
17.02	1.06	0	7.45	1.06	0	4.26	1.06	1.06
7.83	8.7	2.61	23.48	8.7	1.74	0	2.61	1.74
10.57	13.01	2.44	13.01	3.25	2.44	1.63	0.81	0
12.5	8.33	0	0	0	0	0	0	0
8	8	0	0	0	0	0	0	0
6.25	6.25	0	8.33	2.08	2.08	0	2.08	0
3.61	6.02	6.02	16.87	3.61	1.2	3.61	2.41	1.2
13.25	7.23	3.61	13.25	1.2	2.41	2.41	1.2	0
12.2	14.63	2.44	17.07	2.44	0	2.44	0	4.88
13.33	8.89	0	13.33	6.67	4.44	4.44	2.22	0
8.85	6.19	1.77	11.5	0	0.88	1.77	1.77	0.88
6.45	6.45	3.23	9.68	0	0	3.23	3.23	0
4	8	2.67	18.67	2.67	0	2.67	6.67	1.33
6.9	6.9	3.45	0	0	0	0	0	0
3.49	8.14	2.33	11.63	3.49	1.16	1.16	1.16	2.33
3.49	8.14	2.33	11.63	3.49	1.16	1.16	1.16	2.33
12.5	11.61	2.68	15.18	3.57	0.89	1.79	5.36	0
10.34	6.9	0	3.45	0	0	0	0	0
6.67	18.67	1.33	22.67	2.67	1.33	2.67	5.33	2.67
12.5	8.33	8.33	33.33	0	0	12.5	20.83	0
11.48	1.64	3.28	13.11	1.64	4.92	0	3.28	1.64
5.56	3.7	1.85	7.41	0	1.85	1.85	1.85	1.85
4.17	10.42	2.08	20.83	4.17	0	4.17	2.08	2.08
0	19.05	0	14.29	0	0	0	9.52	4.76
13.89	5.56	2.78	8.33	5.56	0	0	0	0
0	7.69	7.69	15.38	0	0	0	7.69	0

8	6.67	2.67	12	5.33	1.33	0	1.33	0
4.17	8.33	4.17	16.67	0	0	0	8.33	0
15	0	0	15	5	0	0	5	0
6.67	5	1.67	10	0	0	5	0	0
13.04	4.35	4.35	19.57	0	0	2.17	4.35	2.17
13.64	4.55	0	13.64	0	0	0	9.09	0
8.54	7.32	2.44	19.51	3.66	2.44	3.66	1.22	3.66
11.76	5.88	0	0	0	0	0	0	0
11.02	9.32	3.39	19.49	0.85	1.69	5.08	3.39	0.85
23.53	0	0	17.65	5.88	0	11.76	5.88	0
9.78	9.78	1.09	10.87	3.26	2.17	1.09	0	1.09
11.76	17.65	5.88	17.65	0	5.88	0	0	0
10.61	9.09	0	12.12	3.03	0	0	1.52	1.52
12.82	7.69	0	7.69	0	0	0	2.56	2.56
7.41	7.41	0	9.26	1.85	0	0	1.85	0
6.25	12.5	0	0	0	0	0	0	0
6.25	6.25	3.13	9.38	0	0	0	3.13	0
3.75	6.25	2.5	12.5	1.25	1.25	3.75	5	2.5
0	7.69	0	0	0	0	0	0	0
2.78	8.33	2.78	25	8.33	11.11	5.56	2.78	0
0	7.41	1.85	22.22	7.41	1.85	1.85	7.41	0
0	7.38	1.34	15.44	2.68	2.01	2.01	2.01	0
0	0	0	15	5	0	10	0	0
0	0	0	15	5	0	10	0	0
2.44	7.32	0	19.51	4.88	2.44	2.44	4.88	0
0	0	0	0	0	0	0	0	0
0	7.06	0	9.41	0	1.18	4.71	2.35	0
3.33	6.67	0	13.33	3.33	0	3.33	3.33	0
0	16.13	0	16.13	0	0	0	6.45	0
0	5.88	0	11.76	5.88	0	5.88	0	0
0	8.41	0	4.67	0.93	0	0	2.8	0
1.05	7.37	0	9.47	2.11	1.05	2.11	3.16	0
0	2.47	0	8.64	0	1.23	2.47	2.47	1.23
0	7.89	2.63	13.16	0	0	5.26	0	0
5.41	8.11	0	5.41	0	0	5.41	0	0
4.46	8.93	1.79	8.04	1.79	0.89	0.89	3.57	0
8.33	20.83	4.17	12.5	0	0	4.17	8.33	0
0	0	0	4.76	0	0	0	0	0
11.36	9.09	0	20.45	2.27	0	4.55	9.09	0
0	7.32	2.44	7.32	2.44	0	0	3.66	0
1.25	7.5	3.75	15	1.25	0	3.75	5	1.25
0	6.02	3.01	12.03	0	0.75	3.01	3.76	0
0	10.26	0	5.13	0	0	0	0	2.56
0	7.55	0	11.32	1.89	0	0	1.89	5.66
0	1.56	1.56	6.25	0	1.56	0	3.13	1.56
0	5.88	2.94	8.82	0	0	0	5.88	0
0	4.17	4.17	12.5	0	0	0	0	4.17
0	8.2	0	11.48	4.92	4.92	0	0	0
0	0	0	25	25	12.5	0	0	0
2	10	3	17	5	4	1	6	3

0	7.14	1.19	11.9	1.19	2.38	2.38	1.19	2.38
0	10.71	0	10.71	3.57	0	3.57	3.57	0
0	2.22	2.22	22.22	2.22	2.22	2.22	6.67	6.67
1.96	5.88	1.96	15.69	1.96	0	3.92	5.88	1.96
0	9.09	4.55	13.64	4.55	0	0	4.55	0
0	5.26	0	15.79	2.63	0	0	2.63	2.63
0	0	0	10	0	0	0	0	0
0	3.45	6.9	17.24	3.45	3.45	3.45	6.9	0
0	8.82	0	8.82	2.94	0	0	0	0
0	0	11.11	11.11	0	0	11.11	0	0
0	6.02	0	19.28	4.82	1.2	2.41	6.02	0
0	13.33	0	13.33	3.33	3.33	6.67	3.33	0
0	5.56	0	11.11	0	0	5.56	5.56	0
0	8.06	0	14.52	6.45	1.61	0	4.84	0
0	6.38	0	17.02	2.13	4.26	4.26	6.38	0
0	1.72	0	5.17	0	1.72	0	1.72	0
2.04	8.16	4.08	12.24	1.02	0	2.04	1.02	3.06
0	0	0	12	0	0	4	4	0
0	2.86	0	14.29	5.71	0	2.86	2.86	0
3.85	11.54	0	11.54	0	0	3.85	3.85	0
5	0	0	10	5	0	0	0	0
0	0	0	30.77	0	0	7.69	0	7.69
0	10.2	0	12.24	2.04	0	4.08	0	2.04
0	12.5	0	18.75	0	0	0	12.5	0
0	7.69	7.69	15.38	0	0	0	7.69	0
2.13	6.38	2.13	12.77	2.13	2.13	2.13	2.13	2.13
0	10.34	3.45	24.14	3.45	0	3.45	10.34	6.9
0	6.06	9.09	12.12	3.03	0	3.03	0	0
0	6.67	3.33	10	0	0	0	6.67	0
0	14.29	0	28.57	4.76	0	0	23.81	0
0	13.33	0	6.67	0	0	0	6.67	0
0.47	6.07	1.87	9.35	1.4	0.93	0.93	3.74	0
1.85	8.33	4.63	10.19	0	0.93	1.85	3.7	0
0	10.17	0	6.78	0	0	1.69	1.69	0
2	8	0	10	4	0	0	2	0
3.45	13.79	3.45	17.24	3.45	0	3.45	3.45	0
0	12.12	9.09	12.12	0	0	0	0	0
5	5	0	5	0	0	0	0	0
3.57	7.14	0	10.71	3.57	3.57	0	0	0
9.38	9.38	0	15.63	6.25	0	0	9.38	0
10.53	5.26	0	10.53	5.26	5.26	0	0	0
6.82	6.82	2.27	4.55	2.27	0	2.27	0	0
5.45	16.36	1.82	9.09	1.82	0	3.64	0	0
0	6.25	0	12.5	0	6.25	0	6.25	0
4.35	8.7	0	13.04	0	0	4.35	0	0
0	0	0	3.03	0	0	0	0	0
0	0	9.09	18.18	0	9.09	0	0	0
2.74	4.11	2.74	21.92	2.74	1.37	4.11	2.74	0
0	4.88	2.44	12.2	0	2.44	0	0	0
0	10.42	4.17	16.67	4.17	0	4.17	0	0

7.41	7.41	3.7	11.11	3.7	0	3.7	0	0
3.33	10	0	13.33	6.67	0	3.33	0	0
6.25	6.25	3.13	18.75	3.13	0	3.13	9.38	0
0	6.47	0.36	8.63	2.52	0.72	2.16	1.08	1.44
0	12	4	14	0	0	0	2	0
0	3.7	3.7	11.11	3.7	3.7	1.85	0	0
0	11.11	0	0	0	0	0	0	0
0	15	5	30	0	0	0	0	0
0	0	0	20	20	0	0	0	0
2.7	9.46	0	9.46	1.35	0	1.35	5.41	0
0	0	0	10	10	0	0	0	0
0	15	10	10	0	0	0	0	0
0	5.63	1.41	8.45	1.41	0	0	2.82	0
5.41	10.81	2.7	10.81	2.7	0	2.7	5.41	0
0	9.76	2.44	7.32	2.44	0	0	2.44	0
5.26	5.26	0	10.53	5.26	0	5.26	0	0
0	7.14	4.29	28.57	12.86	2.86	2.86	7.14	0
12.5	0	12.5	12.5	0	0	0	0	0
0	7.58	1.52	7.58	1.52	1.52	1.52	1.52	0
3.57	14.29	0	7.14	0	3.57	3.57	0	0
0	0	11.11	11.11	11.11	0	0	0	0
0	4	0	4	4	0	0	0	0
12.5	18.75	0	12.5	0	0	6.25	6.25	0
0	14.81	0	0	0	0	0	0	0
0	9.68	3.23	9.68	3.23	0	3.23	0	0
0	9.76	0	9.76	0	0	0	4.88	0
0	11.11	0	11.11	11.11	0	0	0	0
5.88	0	5.88	0	0	0	0	0	0
20	10	0	10	0	10	0	0	0
5.88	5.88	5.88	23.53	0	0	11.76	0	0
0	5	0	12.5	2.5	0	2.5	2.5	0
0	12.5	0	12.5	0	0	0	0	0
0	6.96	2.53	17.09	1.9	1.9	1.9	3.16	3.8
0	8.47	1.69	18.64	1.69	3.39	6.78	3.39	0
4.55	4.55	2.27	13.64	0	2.27	0	2.27	0
0	10.64	2.13	23.4	10.64	2.13	0	8.51	0
0	2.94	0.74	14.71	2.21	2.21	0.74	5.15	0
0.88	7.89	1.75	18.42	2.63	4.39	2.63	5.26	1.75
0	10	10	10	0	0	0	0	0
0	3.96	1.98	11.88	3.96	0	3.96	3.96	0
0	0	12.5	18.75	6.25	0	6.25	0	0
4.76	4.76	14.29	23.81	9.52	4.76	4.76	0	0
0	8	0	32	4	0	12	0	4
3.37	8.99	2.25	22.47	3.37	2.25	5.62	4.49	0
0	10.47	0	6.98	1.16	0	1.16	5.81	0
0	6.33	2.53	15.19	0	1.27	5.06	6.33	0
5.88	11.76	11.76	17.65	5.88	0	5.88	5.88	0
0	14.29	28.57	28.57	0	0	0	14.29	0
6.67	6.67	2.22	6.67	4.44	0	0	4.44	0
4.76	4.76	0	9.52	0	0	0	0	0

0	14.71	0	14.71	0	0	2.94	2.94	0
0	5.33	2.67	13.33	4	2.67	2.67	2.67	0
16.67	8.33	0	8.33	0	0	8.33	8.33	0
0	8.89	2.22	4.44	0	0	0	0	0
2.7	5.41	2.7	2.7	2.7	0	0	0	0
2.7	8.11	0	10.81	5.41	0	0	0	0
0	11.9	0	0	0	0	0	0	0
0	14.81	3.7	3.7	0	0	0	0	3.7
0	12	4	16	0	0	4	12	0
4.76	4.76	0	23.81	0	0	9.52	9.52	0
0	10.26	5.13	10.26	2.56	0	5.13	2.56	0
0	4.41	1.47	8.82	1.47	0	0	1.47	2.94
2.94	14.71	0	17.65	5.88	0	2.94	5.88	2.94
0	0	0	4.55	4.55	0	0	0	0
0	8.33	8.33	8.33	0	0	8.33	0	0
0	6.06	6.06	18.18	0	0	3.03	0	9.09
0	0	0	11.11	0	0	0	0	0
0	7.14	3.57	14.29	5.36	1.79	3.57	0	0
0	0	11.11	22.22	0	0	11.11	0	11.11
0	0	0	16.67	0	0	0	16.67	0
0	0	0	0	0	0	0	0	0
0	13.64	4.55	18.18	0	4.55	4.55	0	0
7.41	7.41	0	3.7	0	0	0	0	0
0	3.13	0	18.75	6.25	3.13	0	9.38	0
0	9.09	0	13.64	4.55	4.55	4.55	9.09	0
0	0	4.76	14.29	4.76	0	0	4.76	0
4.76	4.76	0	9.52	4.76	0	0	0	0
0	0	0	14.29	7.14	14.29	0	0	0
0	16	4	8	0	0	0	4	0
0	11.11	0	11.11	0	0	0	11.11	0
0	6.99	2.1	9.79	2.8	0	1.4	2.1	0.7
12.5	12.5	0	25	0	0	12.5	6.25	0
10.53	10.53	0	26.32	5.26	0	5.26	15.79	0
0	13.33	0	6.67	0	0	0	0	0
0	9.09	13.64	22.73	4.55	0	0	9.09	4.55
0	10	10	17.5	2.5	0	2.5	2.5	0
0	10	0	6	0	0	2	2	0
2.38	11.9	2.38	4.76	0	2.38	0	0	0
0	12.9	0	9.68	3.23	0	3.23	3.23	0
0	8.59	1.56	12.5	1.56	0	6.25	0.78	0
0	8.33	16.67	33.33	0	0	8.33	8.33	8.33
4.35	8.7	0	17.39	4.35	0	8.7	8.7	0
0	6.25	0	12.5	6.25	0	6.25	0	0
0	0	0	20	0	0	10	10	0
3.45	5.17	3.45	15.52	4.31	0.86	2.59	3.45	0.86
4.44	6.67	0	6.67	0	2.22	0	0	4.44
0	3.57	3.57	0	0	0	0	0	0
0	14.29	0	14.29	7.14	0	0	7.14	0
0	14.29	0	14.29	7.14	0	0	7.14	0
0	15.38	15.38	15.38	0	0	7.69	0	0

0	0	18.18	0	0	0	0	0	0
0	0	0	0	0	0	0	0	0
4.76	4.76	2.38	11.9	2.38	0	4.76	0	0
0	8.7	4.35	8.7	0	0	0	0	0
0	1.72	5.17	6.9	1.72	0	3.45	0	0
0	2.38	0	16.67	0	2.38	4.76	4.76	0
0	4.76	0	16.67	0	2.38	9.52	0	2.38
0	9.23	3.08	17.69	5.38	0.77	4.62	3.08	1.54
0	0	0	0	0	0	0	0	0
0	0	0	4.35	4.35	4.35	0	0	0
0	4.44	4.44	26.67	0	0	4.44	6.67	6.67
0	6.25	0	18.75	6.25	0	12.5	0	0
1.41	11.27	2.82	19.72	8.45	0	2.82	7.04	0
0	7.55	1.89	11.32	0	3.77	1.89	3.77	0
0	12.5	0	12.5	0	0	0	0	0
10	0	0	0	0	0	0	0	0
6.25	0	6.25	6.25	6.25	0	0	0	0
0	2.78	5.56	13.89	5.56	2.78	0	5.56	0
0	0	0	4.55	4.55	0	0	0	0
0	0	2.7	18.92	2.7	0	0	5.41	0
1.69	13.56	1.69	16.95	1.69	1.69	3.39	5.08	0
0	8.82	2.94	8.82	5.88	0	0	0	0
0	8.16	0	11.22	3.06	1.02	2.04	3.06	1.02
0	9.52	4.76	19.05	4.76	4.76	4.76	0	0
0	0	0	0	0	0	0	0	0
7.69	0	0	23.08	0	0	7.69	0	0
0	7.69	0	12.82	0	0	5.13	5.13	0
0	0	0	0	0	0	0	0	0
3.45	6.9	0	6.9	0	0	0	6.9	0
0	8.39	2.45	11.54	2.1	0.35	0.7	3.15	1.75
0	5	1.67	16.67	1.67	0	3.33	5	1.67
0	0	0	0	0	0	0	0	0
5	5	5	30	10	0	10	5	0
3.23	8.06	0	8.06	3.23	3.23	0	1.61	0
0	8.33	0	5.56	2.78	2.78	0	0	0
0	9.52	0	0	0	0	0	0	0
14.29	0	14.29	42.86	14.29	0	0	0	14.29
0.6	10.71	1.19	17.86	2.98	2.38	1.19	6.55	1.79
0	7.1	1.94	10.97	1.94	1.29	1.29	6.45	0
4.35	13.04	0	13.04	2.17	2.17	0	4.35	0
6.15	10.77	0	3.08	1.54	0	0	0	0
3.33	13.33	3.33	10	0	0	6.67	0	3.33
0	4	0	20	4	4	0	8	4
7.69	7.69	7.69	30.77	7.69	0	0	23.08	0
0	0	0	28.57	14.29	0	0	14.29	0
0	10	0	10	5	5	0	0	0
3.45	8.05	0	11.49	3.45	0	3.45	3.45	0
0	2.63	0	23.68	5.26	7.89	2.63	10.53	0
1.37	9.59	1.37	19.18	2.74	2.74	4.11	2.74	0
0	7.14	2.14	15	1.43	0	4.29	6.43	0

1.75	1.75	5.26	12.28	8.77	0	0	0	0
1.49	8.96	0	8.96	0	0	1.49	7.46	0
0	13.04	0	14.49	1.45	0	4.35	7.25	0
0	9.43	0	9.43	0	0.94	0.94	3.77	0
3.57	10.71	7.14	21.43	0	3.57	3.57	7.14	0
0	6.58	0	11.84	3.95	0	3.95	3.95	0
16	4	4	16	4	0	4	4	0
0	5.49	1.1	7.69	4.4	0	1.1	3.3	0
2.33	9.3	2.33	11.63	0	0	2.33	2.33	4.65
0	8.11	2.7	8.11	0	5.41	0	2.7	0
0	10	0	20	5	0	5	10	0
0	4.17	4.17	8.33	0	0	0	0	0
2.94	5.88	0	14.71	2.94	2.94	2.94	2.94	0
0	6.45	0	9.68	3.23	0	3.23	0	0
0	11.54	0	0	0	0	0	0	0
3.13	0	0	3.13	3.13	0	0	0	0
0	9.09	9.09	0	0	0	0	0	0
0	2.41	2.41	3.61	0	0	2.41	0	0
0	11.76	3.92	9.8	0	0	1.96	1.96	0
1.45	10.14	0	5.8	1.45	0	0	2.9	0
0	7.14	2.04	6.12	1.02	1.02	0	1.02	0
0	9.09	0	0	0	0	0	0	0
0	6.92	1.89	8.81	2.52	0.63	1.26	2.52	0
0	9.76	1.22	15.85	2.44	2.44	4.88	3.66	0
0	15	5	25	5	5	10	0	0
0	9.09	0	21.21	6.06	6.06	3.03	3.03	3.03
0	7.37	3.16	14.74	2.11	4.21	1.05	4.21	1.05
1.75	10.53	1.75	7.02	3.51	0	0	1.75	1.75
7.41	11.11	0	3.7	0	0	3.7	0	0
4.23	5.63	0	14.08	4.23	2.82	4.23	4.23	2.82
0	7.77	3.88	19.42	2.91	4.85	1.94	5.83	0
0	20	0	10	0	10	0	0	0
0	4.35	13.04	13.04	0	8.7	0	0	0
0	5.26	1.75	8.77	1.75	0	1.75	1.75	0
0	5.13	1.28	8.97	5.13	1.28	0	3.85	1.28
0	4.76	0	4.76	4.76	0	0	4.76	0
0	6.25	0	12.5	6.25	6.25	0	0	0
5.71	8.57	0	8.57	0	0	5.71	2.86	0
0	5.45	1.82	12.73	0.91	0.91	0.91	5.45	0.91
0	9.4	0.67	12.08	2.01	1.34	0	2.68	1.34
0	11.76	5.88	17.65	2.94	2.94	0	5.88	0
10.45	11.94	4.48	17.91	2.99	0	2.99	1.49	1.49
8.33	2.78	2.78	11.11	0	0	2.78	2.78	2.78
0	18.18	18.18	36.36	9.09	0	0	9.09	0
0	5.41	1.35	14.86	2.7	2.7	2.7	2.7	2.7
0	9.49	0.73	7.3	2.19	0.73	2.92	2.19	0
0	3.77	5.66	15.09	5.66	5.66	1.89	0	0
0	6.67	3.33	6.67	0	3.33	0	3.33	0
0	4.55	9.09	27.27	4.55	4.55	4.55	4.55	0
0	0	0	8.33	0	0	8.33	0	0

10	0	0	20	0	0	10	10	0
0	10	2	16	6	4	6	0	0
11.11	5.56	0	11.11	5.56	0	5.56	0	0
0	11.76	11.76	29.41	5.88	0	5.88	5.88	5.88
7.69	0	7.69	15.38	7.69	0	7.69	0	0
0	0	0	0	0	0	0	0	0
7.69	0	0	15.38	0	0	7.69	7.69	0
0	9.26	1.85	12.96	1.85	0	1.85	1.85	0
0	4	0	4	0	0	0	0	0
3.33	13.33	0	6.67	0	3.33	0	0	0
0	10.26	0	7.69	0	2.56	2.56	0	0
5.41	8.11	0	10.81	2.7	0	2.7	5.41	0
0	5.88	0	17.65	5.88	0	11.76	5.88	0
0	0	0	10	0	0	0	0	10
0	0	0	0	0	0	0	0	0
0	10	0	20	10	0	0	0	0
1.37	10.96	4.11	21.92	4.11	1.37	2.74	6.85	1.37
0	6.9	0	17.24	0	6.9	3.45	3.45	0
0	7.64	1.27	11.46	1.91	1.91	1.27	3.82	0
0	15.28	1.39	25	2.78	0	9.72	6.94	0
0	6.45	3.23	3.23	0	0	0	0	3.23
0	11.11	5.56	16.67	5.56	0	5.56	0	0
0	0	0	0	0	0	0	0	0
0	7.5	0	10	5	0	0	0	0
7.69	15.38	7.69	7.69	0	0	0	0	0
0	4.35	0	17.39	4.35	0	13.04	0	0
0	0	0	25	25	0	0	0	0
0	8.22	4.11	16.44	1.37	0	4.11	4.11	1.37
0	6.12	4.08	20.41	6.12	4.08	4.08	2.04	0
0	13.33	6.67	33.33	6.67	0	6.67	0	0
0	4.88	4.88	9.76	0	0	2.44	2.44	0
7.14	0	0	14.29	0	0	7.14	7.14	0
0	12.5	12.5	12.5	0	0	0	6.25	0
0	20	0	10	10	0	0	0	0
0	8.11	1.8	9.91	2.7	0	1.8	4.5	0
0	7.69	0	23.08	11.54	7.69	3.85	3.85	0
0	11.32	0	7.55	0	1.89	1.89	1.89	0
0	9.09	3.03	24.24	3.03	6.06	0	6.06	3.03
9.09	0	9.09	18.18	9.09	0	0	0	0
2.56	5.13	0	7.69	0	0	2.56	2.56	0
0	0	0	0	0	0	0	0	0
0	4	0	4	0	4	0	0	0
0	0	0	11.11	0	0	0	0	0
12.5	6.25	0	0	0	0	0	0	0
0	50	0	0	0	0	0	0	0
0	6.06	3.03	19.7	6.06	0	4.55	4.55	0
8.7	17.39	0	13.04	4.35	0	4.35	4.35	0
0	2.08	2.08	4.17	2.08	0	0	0	0
0	0	0	30	0	0	10	0	0
1.69	8.47	3.39	22.03	5.08	3.39	6.78	5.08	0

0	0	0	0	0	0	0	0	0
0	4.94	1.23	9.88	1.23	0	2.47	2.47	0
0	11.11	7.41	22.22	0	3.7	7.41	3.7	0
0	4.35	0	17.39	4.35	0	0	0	4.35
2	10	6	12	2	6	0	2	0
0	0	0	0	0	0	0	0	0
0	5.26	5.26	15.79	5.26	0	5.26	5.26	0
0	0	0	12.5	0	0	0	0	0
0	0	0	10	0	0	0	0	0
0	0	0	11.11	0	0	11.11	0	0
0	10	0	10	0	10	0	0	0
0	0	0	16.67	16.67	0	0	0	0
6.25	6.25	3.13	21.88	9.38	0	3.13	0	0
0	6.25	0	18.75	6.25	6.25	0	6.25	0
0	15.79	5.26	10.53	0	0	5.26	5.26	0
0	15	5	20	0	0	5	5	0
0	7.14	0	7.14	0	0	7.14	0	0
0	0	0	0	0	0	0	0	0
6.67	0	0	6.67	0	0	0	0	0
0	8.82	2.94	17.65	5.88	0	2.94	2.94	5.88
2.33	11.63	2.33	11.63	0	0	2.33	6.98	0
0	11.76	0	11.76	0	0	5.88	5.88	0
0	6.58	0	15.79	6.58	0	2.63	6.58	0
0	8.43	0	14.46	2.41	1.2	2.41	7.23	1.2
5.88	0	0	11.76	0	0	11.76	0	0
10.53	15.79	5.26	5.26	0	0	5.26	0	0
0	6.06	6.06	6.06	0	0	0	0	0
13.79	13.79	3.45	10.34	0	0	6.9	0	0
0	7.23	4.82	12.05	2.41	0	3.61	4.82	0
0	6.25	0	6.25	0	0	6.25	0	0
0	4.1	0.82	17.21	4.1	1.64	2.46	3.28	0
17.65	0	5.88	23.53	5.88	0	5.88	0	0
4.76	14.29	9.52	33.33	4.76	0	9.52	4.76	4.76
0	4.94	2.47	12.35	3.7	0	1.23	7.41	0
0	10.53	0	0	0	0	0	0	0
0	8.7	0	8.7	0	0	4.35	4.35	0
0	0	0	8.33	0	0	8.33	0	0
0	13.33	13.33	6.67	0	0	0	0	0
1.92	9.62	0	13.46	3.85	3.85	0	3.85	0
0	14.29	0	28.57	8.57	2.86	5.71	8.57	2.86
4.76	4.76	0	14.29	4.76	0	0	9.52	0
0	33.33	0	33.33	0	0	0	0	0
0	0	0	11.11	0	0	11.11	0	0
0	0	16.67	22.22	0	0	11.11	0	0
0	12	4	12	0	0	4	8	0
0	11.39	1.27	12.66	2.53	2.53	1.27	2.53	0
0	0	0	20	20	0	0	0	0
0	12.5	12.5	25	0	0	0	12.5	0
1.85	11.11	7.41	20.37	3.7	0	3.7	3.7	1.85
0	8.33	8.33	27.78	8.33	0	2.78	5.56	2.78

0	5.56	0	11.11	0	0	0	0	5.56
0	12.12	3.03	6.06	3.03	0	0	0	0
5	5	0	5	5	0	0	0	0
0	0	6.67	33.33	20	6.67	6.67	0	0
3.39	3.39	1.69	23.73	8.47	0	1.69	6.78	6.78
2.56	7.69	2.56	17.95	7.69	2.56	5.13	2.56	5.13
0	0	9.09	0	0	0	0	0	0
0	8	0	28	4	4	4	12	0
0	0	14.29	28.57	0	0	0	0	14.29
0	18.18	0	13.64	4.55	0	0	9.09	0
2.08	8.33	0	14.58	4.17	0	4.17	2.08	0
0	5.56	0	5.56	0	0	0	0	0
0	8.03	2.55	12.04	2.19	1.09	1.46	1.46	1.46
2.56	5.13	0	7.69	2.56	0	5.13	0	0
3.7	14.81	0	29.63	0	0	7.41	7.41	0
0	10.87	2.17	10.87	2.17	0	0	6.52	0
0	16	0	28	4	4	8	8	0
2.5	10	2.5	20	10	0	0	2.5	2.5
0	0	0	10.53	5.26	0	5.26	0	0
0	0	14.29	0	0	0	0	0	0
0	0	20	0	0	0	0	0	0
0	8.77	1.75	14.04	0	1.75	3.51	1.75	1.75
7.14	7.14	0	7.14	0	0	0	0	0
1.64	4.92	3.28	6.56	1.64	0	0	0	0
0	10	0	3.33	0	0	3.33	0	0
0	5.66	0	4.72	1.89	1.89	0	0.94	0
0	9.2	1.15	14.94	1.15	0	4.6	1.15	1.15
4.55	9.09	4.55	31.82	4.55	0	13.64	4.55	0
0	10	10	10	0	0	10	0	0
0	3.64	1.82	18.18	1.82	3.64	5.45	3.64	0
0	3.57	7.14	14.29	0	3.57	3.57	0	0
20	0	0	20	20	0	0	0	0
0	5.6	1.6	8.8	3.2	0.8	2.4	2.4	0
3.23	9.68	3.23	9.68	3.23	0	3.23	3.23	0
0	7.41	0	3.7	3.7	0	0	0	0
0	14.29	7.14	35.71	0	0	7.14	7.14	0
0	18.52	7.41	18.52	0	0	3.7	7.41	0
2.3	6.32	0.57	12.64	4.6	2.87	1.15	1.72	0.57
0	6.25	1.04	17.71	5.21	3.13	2.6	2.6	1.56
0	8.68	1.5	14.07	2.69	1.5	2.4	1.8	0.9
3.45	10.34	3.45	20.69	3.45	0	0	6.9	3.45
15.38	7.69	0	15.38	15.38	7.69	0	0	0
0	15.63	0	10.94	3.13	3.13	0	3.13	0
4.88	5.49	3.05	17.07	3.66	1.22	2.44	3.05	0.61
0	7.89	2.63	13.16	5.26	2.63	1.75	0.88	0
0	8.33	8.33	25	0	0	16.67	8.33	0
1.08	7.53	0	0	0	0	0	0	0
0	6.67	0	13.33	0	0	3.33	6.67	0
0	0	0	13.33	13.33	0	0	0	0
3.17	8.73	0.79	11.9	2.38	1.59	2.38	1.59	0.79

0	5.17	0.86	16.38	4.31	4.31	1.72	0.86	0.86
1.22	9.12	0.61	16.11	3.95	2.43	3.34	2.43	2.13
0	6.52	0	4.35	2.17	0	0	0	1.09
2.33	2.33	0	4.65	4.65	0	0	0	0
0	2.1	1.4	11.89	5.59	1.4	1.4	2.1	0.7
0	4.55	1.52	12.12	4.55	1.52	4.55	1.52	0
0	11.28	1.13	13.91	3.76	2.26	1.13	2.63	1.5
0.69	5.52	1.38	17.24	4.14	2.07	4.14	4.14	0.69
1.85	7.41	1.85	14.81	3.7	1.85	3.7	0	1.85
20	0	0	20	0	0	0	0	20
0.82	13.11	1.64	13.93	3.28	0.82	1.64	4.92	2.46
0	2.94	0	11.76	2.94	2.94	0	2.94	0
0.96	5.77	3.85	11.54	0	0	4.81	1.92	0.96
0	11.76	0	23.53	0	0	0	17.65	0
0	7.55	3.77	37.74	13.21	5.66	1.89	9.43	0
0	0	0	0	0	0	0	0	0
0	3.77	1.89	15.09	3.77	1.89	3.77	3.77	1.89
0	0	0	20	0	0	0	0	0
0	2.78	8.33	13.89	2.78	5.56	0	5.56	5.56
6.82	6.82	4.55	27.27	0	2.27	6.82	4.55	6.82
0	8.16	4.08	6.12	2.04	2.04	0	4.08	0
1.47	8.82	1.47	13.24	1.47	2.94	5.88	1.47	0
0	14.29	0	28.57	7.14	0	14.29	0	0
0	5.56	0	16.67	5.56	5.56	0	0	2.78
0	6.25	2.08	10.42	2.08	0	2.08	2.08	0
2.2	12.09	0	18.68	1.1	4.4	3.3	6.59	0
0	5	2.5	18.75	5	2.5	0	8.75	1.25
0	11.01	3.96	16.74	3.08	1.76	3.52	4.41	0.44
3.06	8.16	1.02	14.29	4.08	2.04	4.08	2.04	1.02
0	0	0	11.11	0	0	0	0	11.11
0	0	0	0	0	0	0	0	0
0	12.5	0	25	12.5	0	0	0	0
0	9.09	0	13.64	0	0	4.55	4.55	0
12.5	0	0	0	0	0	0	0	0
0	0	0	40	20	20	0	0	0
0	4.35	4.35	8.7	0	0	4.35	0	0
1.04	5.21	3.13	20.83	4.17	3.13	3.13	2.08	4.17
0	11.86	1.69	18.64	1.69	3.39	0	6.78	0
0	4.88	4.88	9.76	0	2.44	4.88	0	0
0	4.35	0	21.74	4.35	2.17	8.7	2.17	0
0	5.36	1.79	7.14	0	0	3.57	0	0
2.86	0	5.71	31.43	5.71	0	8.57	2.86	2.86
0	9.52	4.76	14.29	0	0	0	0	0
0.82	7.38	1.64	16.12	2.19	0.55	3.28	4.37	0.27
0	12.07	1.72	17.24	1.72	1.72	3.45	8.62	0
0.78	9.38	3.13	13.28	3.13	3.13	1.56	1.56	0.78
0	7.69	15.38	23.08	7.69	7.69	0	0	0
0	6.67	0	20	0	3.33	3.33	3.33	3.33
0	0	0	22.22	11.11	0	11.11	0	0
1.8	11.87	1.8	13.67	2.88	0.72	3.96	3.6	0.36

0	25	0	25	0	0	12.5	12.5	0
5.26	5.26	5.26	26.32	0	5.26	0	10.53	0
0	0	0	11.11	0	0	0	11.11	0
0	5.26	5.26	15.79	5.26	0	0	0	0
0.67	8.67	2.67	17.33	3.33	0.67	3.33	1.33	0.67
3.23	6.45	6.45	25.81	3.23	0	6.45	12.9	0
0	0	0	40	20	20	0	0	0
0	11.58	5.26	20	2.11	1.05	6.32	3.16	0
0.96	6.73	0.96	13.46	2.88	0	2.88	2.88	0
0	0	0	25	12.5	0	12.5	0	0
8.7	4.35	8.7	13.04	0	0	0	4.35	0
0	4.17	0	8.33	0	0	0	0	4.17
1.52	9.09	7.58	18.18	1.52	1.52	0	7.58	0
0	12.5	6.25	8.33	0	0	0	6.25	0
0	7.27	0	18.18	3.64	0	3.64	0	7.27
1.67	6.67	3.33	16.67	3.33	1.67	3.33	5	0
0	5.13	0.85	9.4	2.56	0.85	1.71	1.71	0
0	8.33	0	5	0	0	1.67	3.33	0
1.83	6.71	3.66	12.2	2.44	2.44	3.05	4.27	0
0	8.2	0	8.2	0	1.64	1.64	0	0
0	3.85	0	15.38	3.85	1.92	1.92	0	3.85
0	10.39	1.3	18.18	2.6	0	0	3.9	3.9
0	3.76	2.26	10.53	0.75	1.5	2.26	2.26	0.75
0	8.2	0	14.75	1.64	1.64	1.64	8.2	0
0	5	10	25	5	0	10	0	0
7.14	4.76	4.76	13.1	3.57	0	1.19	4.76	1.19
0	0	0	37.5	12.5	12.5	0	12.5	0
0	2.78	2.78	0	0	0	0	0	0
0	0	12.5	18.75	0	0	6.25	0	0
0	7.94	1.59	12.7	3.17	1.59	0	3.17	3.17
0	4.17	6.25	14.58	2.08	0	4.17	4.17	2.08
0	2	2	12	2	0	2	0	4
5.88	0	0	11.76	11.76	5.88	0	0	0
0	8.2	0	4.92	0	0	0	1.64	0
0	7.46	0	7.46	0	0	2.99	1.49	0
0	4.76	3.17	19.05	9.52	1.59	4.76	0	0
1.37	2.74	1.37	9.59	2.74	2.74	2.74	0	1.37
0	4.17	4.17	12.5	0	4.17	8.33	0	0
0	6.67	0	3.33	3.33	0	0	0	0
0	10.53	0	5.26	5.26	0	0	0	0
0	3.57	7.14	14.29	1.79	0	3.57	0	1.79
0	7.27	1.82	12.73	1.82	1.82	1.82	3.64	1.82
0	9.84	0	16.39	1.64	0	3.28	3.28	1.64
0	6.9	3.45	13.79	0	0	0	3.45	0
0	7.69	1.54	12.31	1.54	0	3.08	4.62	0
1.63	10.57	0.81	8.94	2.44	0.81	1.63	2.44	1.63
0	0	7.69	0	0	0	0	0	0
1.06	9.57	2.13	10.64	1.06	2.13	0	1.06	2.13
0	12.5	6.25	25	0	0	18.75	0	0
16.67	0	0	16.67	0	0	16.67	0	0

2.33	13.95	2.33	15.12	3.49	0	3.49	5.81	0
2.56	7.69	5.13	10.26	0	2.56	2.56	0	0
0	4	0	8	0	4	0	0	0
0	6.38	1.06	21.28	4.26	4.26	7.45	2.13	3.19
0	9.23	1.54	10.77	0	0	3.08	4.62	0
0	6.82	2.27	13.64	0	2.27	0	6.82	0
0	0	0	0	0	0	0	0	0
8.06	3.23	1.61	19.35	9.68	4.84	0	1.61	0
0	6.67	6.67	26.67	0	0	6.67	0	13.33
0	5.41	0	4.05	2.7	0	0	0	0
0	8.4	3.05	14.5	3.82	0.76	1.53	3.05	0.76
0	2.7	1.35	9.46	0	1.35	6.76	1.35	0
0	7.41	3.7	3.7	0	0	3.7	0	0
0	8.82	5.88	20.59	1.47	0	4.41	8.82	0
0	12.96	3.7	18.52	5.56	0	3.7	7.41	0
0	8.67	3.33	16	2.67	2	3.33	0.67	0.67
0	5.56	4.17	13.89	1.39	0	1.39	6.94	0
0	16	0	12	0	0	0	0	0
0	6.82	3.79	8.33	3.03	0	2.27	0	0
1.91	7.01	1.27	19.11	3.18	1.27	4.46	4.46	0.64
0	7.41	3.7	18.52	3.7	3.7	3.7	0	0
0	7.84	3.92	13.73	1.96	1.96	1.96	3.92	0
0	2.27	6.82	11.36	0	0	0	2.27	0
1.09	3.8	1.09	9.78	2.17	1.09	2.17	1.09	0.54
0	9.8	3.92	9.8	1.96	0	1.96	1.96	0
2.76	5.52	4.14	14.48	0.69	0.69	3.45	3.45	0.69
5	5	5	30	15	5	0	5	0
0	0	3.13	18.75	3.13	3.13	0	6.25	0
1.43	5.71	4.29	24.29	0	1.43	8.57	5.71	0
0	9.52	4.76	11.9	0	0	2.38	4.76	0
0	7.27	5.45	12.73	0	0	0	0	5.45
0	5	7.5	15	2.5	0	0	5	2.5
0	9.77	2.3	15.52	2.87	1.15	1.72	5.17	1.15
6.45	0	6.45	35.48	12.9	3.23	0	16.13	0
0	4.08	6.12	18.37	4.08	2.04	4.08	2.04	6.12
0	14.71	0	14.71	5.88	0	2.94	0	0
1.89	5.66	3.77	15.09	1.89	1.89	0	5.66	0
0	0	0	11.11	0	0	0	11.11	0
0	10.87	2.17	28.26	4.35	0	4.35	6.52	6.52
15	10	0	40	10	0	10	15	0
1.89	1.89	1.89	13.21	1.89	1.89	1.89	3.77	0
0	14.29	7.14	21.43	0	0	0	7.14	0
0	9.68	4.03	12.1	1.61	0.81	0.81	3.23	0.81
3.39	5.93	2.54	15.25	3.39	0.85	2.54	3.39	2.54
1.32	5.3	2.65	9.93	1.32	1.32	1.32	1.32	1.32
0	3.45	3.45	10.34	2.59	0	0.86	2.59	0.86
6.48	7.41	6.48	14.81	5.56	3.7	0.93	0	0
2	6.67	3.33	16	0	0.67	2	4.67	2
0	12.7	4.76	11.11	0	0	0	3.17	0
18.18	0	0	9.09	9.09	0	0	0	0

0	10	6.25	17.5	2.5	1.25	2.5	3.75	1.25
0	9.05	0	11.43	1.9	0.48	2.86	2.38	0.95
7.89	7.89	0	23.68	7.89	0	0	7.89	2.63
0	8.33	2.08	18.75	6.25	2.08	2.08	4.17	2.08
6.67	6.67	6.67	6.67	6.67	0	0	0	0
0	0	0	0	0	0	0	0	0
0.77	6.92	0	12.31	3.08	0	2.31	1.54	1.54
0	9.52	9.52	19.05	4.76	0	4.76	0	4.76
6.06	15.15	0	6.06	0	0	3.03	0	0
2.78	5.56	2.78	2.78	0	0	2.78	0	0
0	10.91	1.82	27.27	12.73	1.82	1.82	1.82	1.82
0	0	0	11.11	5.56	5.56	0	0	0
0	0	0	0	0	0	0	0	0
5	6.25	1.25	11.25	0	1.25	1.25	3.75	0
0	10.81	2.7	10.81	0	0	0	0	2.7
7.5	15	0	7.5	0	0	0	0	2.5
2.63	5.26	0	7.89	5.26	0	0	0	0
5.26	5.26	4.39	23.68	5.26	0.88	3.51	5.26	2.63
0	2.5	2.5	17.5	5	2.5	0	7.5	2.5
6.25	6.25	6.25	18.75	0	6.25	6.25	6.25	0
0	5.13	2.56	20.51	2.56	5.13	7.69	2.56	0
0	0	7.69	19.23	3.85	0	0	3.85	0
0	7.58	0	9.09	4.55	1.52	0	1.52	0
2.27	9.09	0	13.64	0	0	6.82	4.55	2.27
0	0	0	0	0	0	0	0	0
10	0	0	0	0	0	0	0	0
0	14.63	2.44	7.32	0	0	2.44	0	0
0	0	5.88	11.76	0	5.88	0	0	0
0	0	0	20	0	0	10	10	0
3.85	10.26	2.56	10.26	0	1.28	2.56	1.28	0
0	7.14	3.57	17.86	2.86	2.86	2.86	4.29	0
0	10.77	0	9.23	3.08	0	1.54	1.54	0
2.5	6.25	0	18.75	7.5	1.25	2.5	1.25	1.25
0	5.88	0	7.35	0	1.47	1.47	1.47	1.47
0	2.94	0	17.65	5.88	0	0	11.76	0
0	7.84	1.96	3.92	0	0	1.96	1.96	0
0	11.11	2.78	16.67	2.78	0	2.78	2.78	2.78
0.6	6.63	1.2	10.84	1.81	1.81	0.6	3.01	0.6
0	7.63	4.24	12.71	0.85	0.85	2.54	2.54	0
0	10	20	30	0	10	0	10	0
0	0	10	30	10	0	10	10	0
18.75	6.25	0	18.75	0	6.25	6.25	6.25	0
0	9.15	3.52	15.49	2.82	1.41	2.11	5.63	0
4.1	8.2	4.92	13.93	3.28	0.82	1.64	4.1	0
0	10.91	3.64	16.36	0	3.64	1.82	3.64	0
0	5.46	3.28	18.03	4.37	1.09	0.55	3.28	1.64
0	14.58	2.08	12.5	4.17	0	0	4.17	2.08
2.48	7.44	7.44	19.01	4.13	2.48	1.65	4.96	0.83
8.7	8.7	8.7	8.7	8.7	0	0	0	0
0	0	0	16.67	0	0	16.67	0	0

1.54	7.69	1.54	15.38	1.54	0	0	4.62	3.08
1.16	5.78	2.31	12.72	1.73	2.31	2.31	2.89	0
0	5.26	2.26	12.03	0.75	0.75	1.5	3.01	2.26
0	6.32	0	9.47	2.11	1.05	1.05	0	1.05
0.49	6.81	1.95	10.46	2.43	0.49	1.22	1.95	0.73
0	0	0	10	0	10	0	0	0
0.49	5.91	2.46	13.79	3.94	0.99	2.46	0.99	1.97
0	7.11	1.52	11.68	3.05	0	2.03	4.06	0.51
8.33	8.33	0	16.67	8.33	0	0	8.33	0
8	6	2	12	2	2	0	6	0
0	5.26	5.26	10.53	0	5.26	0	0	0
9.09	18.18	0	9.09	4.55	0	0	4.55	0
3.85	7.69	0	19.23	0	0	3.85	15.38	0
20	0	0	40	0	0	40	0	0
0	12.07	3.45	13.79	5.17	0	0	1.72	1.72
10	10	0	20	0	0	10	10	0
0	0	0	22.22	11.11	0	0	0	0
0	0	0	28.57	14.29	14.29	0	0	0
0	9.84	1.64	13.11	1.64	1.64	3.28	1.64	0
1.42	11.85	2.84	15.17	2.37	0.47	1.42	7.11	0.95
4.55	13.64	0	22.73	6.82	2.27	6.82	4.55	0
0	10	2	16	0	2	8	4	0
0	3.03	3.03	12.12	3.03	0	3.03	3.03	0
0	11.49	0	9.2	3.45	0	2.3	0	1.15
4.44	8.89	2.22	17.78	2.22	2.22	4.44	4.44	0
0.69	14.48	0.69	13.1	2.07	3.45	3.45	3.45	0
6.67	6.67	0	20	4.44	4.44	4.44	2.22	2.22
0	10	0	23.33	6.67	0	3.33	13.33	0
2.56	10.26	0	23.08	5.13	0	0	12.82	2.56
3.7	7.41	3.7	18.52	3.7	0	0	0	0
0	0	0	0	0	0	0	0	0
0	11.54	0	11.54	7.69	3.85	0	0	0
0	13.64	4.55	9.09	2.27	0	2.27	0	0
0	10	0	20	0	0	0	10	0
2.29	12.21	0.76	16.03	4.58	1.53	3.82	1.53	2.29
2.04	10.2	2.04	16.33	6.12	2.04	4.08	0	0
1.23	9.88	3.7	16.05	8.64	0	0	4.94	0
0	6.25	0	6.25	3.13	0	0	0	0
3.7	5.56	0	9.26	0	1.85	1.85	3.7	0
0	11.11	0	8.89	6.67	0	2.22	0	0
0	13.33	0	13.33	0	0	3.33	6.67	0
0	7.69	15.38	23.08	15.38	0	0	0	0
0	11.85	1.48	11.85	1.48	2.22	0.74	3.7	0
9.09	0	0	9.09	0	0	9.09	0	0
2.86	8.57	2.86	11.43	0	0	0	5.71	0
0	0	0	40	0	20	0	0	20
0	6.67	1.67	15	1.67	0	5	8.33	0
0	0	0	14.29	7.14	7.14	7.14	0	0
0	6.67	0.95	21.9	3.81	0.95	5.71	7.62	1.9
0	8.57	2.86	14.29	2.86	5.71	0	2.86	0

0	10.26	2.56	20.51	5.13	2.56	0	7.69	0
0	9.57	2.13	17.02	1.06	3.19	1.06	3.19	2.13
0	4.76	2.38	14.29	4.76	0	0	4.76	0
0	5.97	5.97	14.93	5.97	0	1.49	2.99	1.49
2.56	12.82	0	7.69	0	0	0	2.56	0
0	12.5	0	20	2.5	5	2.5	7.5	0
8.45	7.04	2.82	23.94	1.41	0	2.82	9.86	1.41
3.57	7.14	10.71	21.43	10.71	3.57	0	3.57	0
0	3.23	0	12.9	6.45	3.23	0	3.23	0
0.47	11.16	2.79	16.74	5.58	3.26	0.47	5.12	1.4
0	6.9	2.3	18.39	1.15	0	2.3	5.75	2.3
0	9.09	0	9.09	0	0	0	0	0
0	7.36	1.84	15.95	1.84	1.84	1.23	2.45	0.61
0	10	0	10	0	0	0	0	0
0	11.81	0	10.24	3.94	2.36	1.57	2.36	0
5.71	5.71	2.86	8.57	2.86	0	5.71	0	0
0	0	0	14.29	0	0	7.14	7.14	0
0	0	0	0	0	0	0	0	0
7.14	0	0	14.29	14.29	0	0	7.14	0
8.33	8.33	4.17	12.5	4.17	4.17	4.17	0	4.17
3.03	9.09	3.03	15.15	3.03	0	3.03	3.03	0
0	6.52	0	21.74	10.87	2.17	0	4.35	0
0	20	0	20	0	0	0	20	0
0	3.57	0	3.57	0	0	0	3.57	0
0	8.11	0	18.92	5.41	5.41	2.7	5.41	0
0	6.06	0	9.09	0	0	3.03	6.06	0
0	11.11	0	33.33	11.11	22.22	0	0	0
0.75	12.03	0.75	16.54	3.01	2.26	1.5	4.51	1.5
0.36	10.68	1.07	11.03	1.42	1.07	2.14	2.49	0
1.25	5	2.5	23.75	11.25	5	1.25	5	0
2.08	16.67	0	14.58	6.25	0	2.08	4.17	0
0.74	8.82	2.21	20.59	3.68	3.68	2.94	7.35	0.74
5.56	5.56	0	22.22	0	0	11.11	0	11.11
0	7.41	0	14.81	3.7	3.7	0	3.7	0
0	6.45	3.23	19.35	3.23	0	3.23	6.45	3.23
2.11	9.08	1.62	19.29	4.7	1.46	3.4	6	0
0	8.87	4.84	15.32	4.03	1.61	2.42	4.84	0
0	9.39	2.21	16.02	2.21	0.55	1.1	6.63	1.1
0	11.38	3.59	22.16	4.19	1.8	4.19	9.58	1.8
0	6.12	2.04	22.45	8.16	6.12	0	2.04	6.12
2.35	7.06	0	24.71	11.76	5.88	1.18	2.35	0
0	6.06	0	27.27	12.12	6.06	3.03	0	0
1.25	10	2.5	16.25	1.25	0	5	6.25	1.25
1.72	0	3.45	10.34	3.45	3.45	0	0	0
0	7.27	1.82	12.73	3.64	0	1.82	1.82	3.64
0	8.97	0	8.97	2.56	0	1.28	1.28	0
0	7.64	2.33	13.95	0.66	1.66	4.98	2.33	0.33
8.33	0	8.33	8.33	0	0	8.33	0	0
3.57	7.14	7.14	17.86	3.57	0	3.57	3.57	0
0	0	0	11.54	3.85	0	7.69	0	0

0	5.26	5.26	18.42	0	2.63	5.26	2.63	0
6.06	6.06	2.02	18.18	2.02	0	6.06	2.02	1.01
1.35	8.11	4.05	16.22	2.7	0	2.7	1.35	0
0	5	5	20	5	0	5	0	0
0	0	12.5	25	12.5	0	12.5	12.5	0
10.67	2.67	4	18.67	4	2.67	6.67	2.67	2.67
12.5	6.25	2.08	12.5	0	0	2.08	6.25	0
10.34	12.07	3.45	12.07	1.72	0	5.17	5.17	0
1.59	6.35	1.59	12.7	4.76	0	4.76	0	0
1.98	3.96	2.97	13.86	0.99	0	6.93	0.99	1.98
7.55	4.72	2.83	14.15	1.89	0.94	2.83	2.83	0
14	2	2	12	4	2	0	2	0
8.7	4.35	4.35	30.43	4.35	4.35	8.7	8.7	0
0.72	5.04	4.32	9.35	1.44	0	1.44	0.72	0.72
11.11	2.78	2.78	11.11	0	0	2.78	0	0
0	7.89	1.32	18.42	4.61	3.29	1.32	4.61	1.32
0	8.89	2.22	13.33	0	0	4.44	6.67	0
2.5	10	0	10	2.5	5	2.5	0	0
1.75	6.14	0	9.65	0.88	1.75	2.63	0	0
0	12.5	9.38	31.25	0	3.13	6.25	6.25	3.13
0	12.5	0	2.5	0	1.25	0	1.25	0
0	8	4	13	2	1	2	0	1
3.57	8.93	3.57	16.07	3.57	0	3.57	5.36	1.79
8.11	4.05	2.7	11.49	0.68	0.68	2.7	0	2.7
4.04	8.08	2.02	20.2	3.03	3.03	7.07	2.02	1.01
0	7.44	0	12.4	2.48	1.65	2.48	1.65	0
9.09	9.09	0	9.09	0	0	0	0	0
1.55	8.29	1.04	14.51	1.04	2.07	1.55	5.18	1.04
0	11.11	0	11.11	0	0	0	0	0
11.9	4.76	2.38	4.76	0	0	4.76	0	0
0	9.52	0	0	0	0	0	0	0
0.34	4.7	1.34	9.4	1.68	2.01	1.01	1.01	0.67
1.52	0	1.52	10.61	1.52	0	0	1.52	3.03
0	6.25	1.04	14.58	3.13	1.04	5.21	3.13	2.08
0	9.09	18.18	45.45	9.09	9.09	9.09	0	0
0	8.51	4.26	25.53	6.38	0	6.38	4.26	4.26
0	0	0	40	0	0	20	20	0
0	16.67	0	8.33	8.33	0	0	0	0
0	0	8.33	25	8.33	8.33	0	0	8.33
0	7.69	0	11.54	0	0	3.85	0	3.85
0	11.76	0	5.88	0	0	0	0	0
5	10	15	10	0	0	5	5	0
0	4.55	0	13.64	4.55	0	9.09	0	0
0	0	0	25	12.5	0	0	0	0
0	0	8.33	33.33	0	0	8.33	0	16.67
0	0	0	0	0	0	0	0	0
4	12	0	10	6	2	0	2	0
0	8.11	0	21.62	2.7	2.7	5.41	2.7	2.7
0	5.41	2.7	8.11	5.41	0	0	0	0
0	12.5	0	12.5	0	0	12.5	0	0

2.22	7.41	0	14.81	2.22	2.22	2.22	4.44	0
0	8.51	2.13	17.02	4.26	0	4.26	2.13	2.13
0	5.56	8.33	27.78	11.11	5.56	2.78	8.33	0
0	8.49	3.44	13.53	2.75	1.38	2.75	3.67	0
0	2.5	2.5	5	0	0	0	0	0
14.29	0	14.29	0	0	0	0	0	0
0	0	0	10	10	0	0	0	0
0	9.76	2.44	2.44	0	0	0	0	0
0	5.13	0	15.38	0	0	7.69	2.56	0
0	6.06	0	12.12	6.06	3.03	3.03	0	0
0	0	0	0	0	0	0	0	0
0.59	5.88	3.82	12.54	1.57	1.67	2.45	2.35	0.69
0	6.45	3.23	10.75	2.15	2.15	1.08	2.15	2.15
0	6.35	3.17	19.05	3.17	2.38	4.76	1.59	3.17
0	6.85	2.74	6.85	0	0	1.37	1.37	0
0	13.21	0	18.87	1.89	1.89	3.77	9.43	0
0	6.5	0.81	9.76	0	0.81	0.81	6.5	0
0	0	0	0	0	0	0	0	0
16.67	0	0	16.67	0	0	0	0	16.67
0	8.02	2.83	14.15	2.83	1.89	1.89	2.83	0.94
0	16.67	0	0	0	0	0	0	0
0.41	9.39	5.31	17.55	4.08	2.04	1.63	3.27	1.22
0	0	0	16.67	16.67	16.67	0	0	0
4	4	3	19	4	4	3	3	1
0	11.76	2.94	17.65	0	2.94	5.88	0	0
3.48	8.82	1.39	9.05	0.46	1.62	1.62	2.55	0.7
4.92	6.56	1.64	16.39	3.28	0	3.28	0	1.64
1.11	5.56	2.22	6.67	2.22	1.11	1.11	0	1.11
0	8.7	0	17.39	0	4.35	0	0	4.35
1.02	6.12	2.04	13.78	3.06	0	2.55	1.53	1.02
0	10.53	2.63	13.16	0	0	5.26	0	2.63
0	4.55	0	13.64	0	0	0	13.64	0
0	2.44	0	12.2	7.32	0	4.88	0	0
0	7.95	1.32	12.58	2.65	1.32	1.32	2.65	1.32
0	16.67	16.67	16.67	16.67	0	0	0	0
1.3	4.78	1.74	10.87	4.35	2.17	1.74	2.17	0.87
1.13	7.21	2.48	12.39	2.7	1.13	2.48	1.13	0
1.82	1.82	1.82	18.18	9.09	1.82	1.82	1.82	0
3.85	3.85	0	15.38	11.54	7.69	0	3.85	0
0.72	7.91	2.16	12.23	2.16	2.16	2.16	1.44	1.44
2	6	2	12	0	2	0	2	2
2.5	2.5	0	12.5	2.5	5	5	0	2.5
0.36	8.54	2.14	14.59	3.56	1.07	1.07	3.56	0.71
2.63	5.26	0	13.16	0	0	5.26	5.26	2.63
1.79	7.14	1.79	15.18	6.25	0.89	4.46	4.46	0.89
3.23	8.87	2.42	12.9	4.84	1.61	1.61	1.61	2.42
0	8.57	5.71	14.29	0.95	1.9	0.95	1.9	0
0.42	6.69	4.18	12.97	3.77	0.84	1.67	1.26	0
3.92	6.86	0.98	13.73	4.9	0.98	1.96	1.96	0.98
1.87	6.54	0.93	13.08	3.74	2.8	0.93	1.87	0.93

2.8	8.41	4.67	18.69	1.87	3.74	2.8	3.74	0
0	2.63	2.63	15.79	5.26	0	5.26	0	0
0	0	0	9.52	0	0	4.76	0	4.76
0	9.36	1.17	15.2	1.75	2.34	1.75	2.92	0
0	4.85	0.97	12.62	0	0	2.91	0	0
12.5	0	0	0	0	0	0	0	0
0	5.88	0.98	11.76	2.94	0	2.94	1.96	0.98
0	8.16	0	14.29	4.08	0	4.08	6.12	0
11.11	0	0	11.11	0	0	11.11	11.11	0
0.69	7.62	1.15	7.85	1.85	0.92	1.15	1.15	0.69
0	13.33	0	14.29	3.81	1.9	2.86	0.95	0.95
7.39	8.52	1.14	11.93	3.41	0.57	2.27	2.84	0.57
18.46	6.15	1.54	6.15	0	0	4.62	0	0
7.27	9.09	0	13.64	1.82	2.73	2.73	2.73	0.91
7.26	8.55	0.43	13.25	3.85	1.28	1.28	1.71	0.85
6.84	8.55	2.56	11.11	0.85	0.85	1.71	1.71	1.71
5.37	11.22	1.95	17.56	4.39	1.46	2.44	2.44	0.98
6.73	8.65	0.96	8.65	1.92	0.96	1.92	0.96	0
4.35	8.7	1.74	16.52	0.87	0.87	0.87	4.35	3.48
3.57	5.95	1.19	16.67	5.95	0	3.57	2.38	0
9.09	0	3.03	3.03	3.03	0	0	3.03	0
2.35	7.06	0.59	15.29	3.53	1.18	3.53	3.53	0.59
1.61	1.61	3.23	11.29	4.84	1.61	3.23	0	0
8.47	3.39	0	11.86	5.08	0	3.39	1.69	1.69
6.1	9.76	0	17.07	2.44	0	3.66	6.1	1.22
8.82	8.82	0	14.71	1.47	0	4.41	2.94	1.47
3.45	6.9	1.15	8.81	2.3	1.15	1.15	2.68	0.38
2.34	8.59	1.56	17.19	3.13	1.56	3.13	6.25	0
13.79	3.45	6.9	17.24	6.9	3.45	3.45	3.45	0
5.96	5.96	1.32	9.27	4.64	0.66	0	2.65	0.66
4.55	6.82	4.55	22.73	2.27	0	6.82	2.27	4.55
7.5	5	0	2.5	0	0	0	2.5	0
2.61	10.43	0.87	12.17	3.48	0.87	2.61	2.61	0
1.73	6.94	3.47	20.23	4.62	0.58	2.89	4.05	2.89
5	10	3.33	10	1.67	0	1.67	1.67	0
1.8	5.41	2.7	15.32	0.9	1.8	4.5	1.8	2.7
5.81	12.79	1.16	14.53	2.91	1.74	1.74	4.07	1.16
3.62	6.36	1.32	16.12	3.62	1.97	2.96	2.74	1.21
5.05	7.07	2.02	17.17	6.06	3.03	4.04	4.04	0
7.8	9.93	2.13	14.18	4.96	0	3.55	1.42	0
4.17	8.33	1.39	11.11	2.78	0	1.39	4.17	0
10	10	5	25	5	0	5	5	5
8.2	4.92	1.64	9.84	8.2	0	0	0	1.64
12	8	2.67	16	8	1.33	1.33	1.33	1.33
5	5	1.67	14.17	0.83	2.5	4.17	3.33	0
0	6.45	0	3.23	0	0	0	0	3.23
2.44	12.2	4.88	21.95	9.76	0	0	4.88	0
4.3	9.68	1.08	7.53	4.3	1.08	0	2.15	0
13.21	7.55	2.83	15.09	2.83	1.89	0.94	2.83	0.94
8.06	8.06	0	8.06	3.23	0	0	3.23	0

10.53	10.53	0	8.77	3.51	1.75	1.75	1.75	0
10.39	7.79	3.9	11.69	1.3	0	2.6	2.6	0
5.88	6.86	0.98	11.76	0	0.98	1.96	2.94	4.9
3.45	9.85	0.49	12.32	3.45	0.49	0.99	4.93	0.49
6.06	12.12	0	0	0	0	0	0	0
12.5	16.67	0	0	0	0	0	0	0

differentia	emotion	emo_pos	emo_neg	emo_anx	emo_ange	emo_sad	social beha	prosocial b
7.46	0	0	0	0	0	0	10.45	0
5.26	5.26	5.26	0	0	0	0	5.26	5.26
2.94	2.94	0	2.94	2.94	0	0	8.82	5.88
5.33	2.67	1.33	1.33	0	0	0	2.67	0
2.7	0	0	0	0	0	0	12.16	4.05
2.63	7.89	5.26	2.63	2.63	0	0	0	0
3.95	0	0	0	0	0	0	2.63	0
7.32	4.88	4.88	0	0	0	0	4.88	0
3.64	3.64	1.82	1.82	1.82	0	0	10.91	1.82
2.88	3.85	2.88	0.96	0	0	0	4.81	1.92
0	3.13	0	3.13	3.13	0	0	6.25	0
0	5.26	0	0	0	0	0	5.26	0
2.61	0.87	0	0.87	0	0	0	4.35	0.87
0	100	0	100	0	0	0	0	0
0	12.5	12.5	0	0	0	0	0	0
0	11.11	7.41	3.7	0	0	0	3.7	3.7
16.67	8.33	0	8.33	0	0	8.33	8.33	0
6.25	6.25	6.25	0	0	0	0	12.5	6.25
0	16.67	8.33	8.33	0	0	0	0	0
2.75	1.83	0.92	0.92	0.92	0	0	6.42	2.75
12.5	0	0	0	0	0	0	0	0
0	0	0	0	0	0	0	12.5	12.5
0	7.14	0	0	0	0	0	14.29	7.14
6.56	1.64	0	1.64	0	0	0	8.2	1.64
1.96	1.96	0	1.96	0	0	0	11.76	0
0	8.82	0	8.82	0	8.82	0	8.82	0
0	6	0	2	0	0	0	8	0
3.66	2.44	0	2.44	1.22	0	2.44	7.32	1.22
0	2.33	2.33	0	0	0	0	9.3	2.33
5.88	2.94	2.94	0	0	0	0	14.71	11.76
4.11	0	0	0	0	0	0	8.22	0
2.44	0	0	0	0	0	0	15.45	0.81
15	5	0	5	0	5	0	10	5
5.88	0	0	0	0	0	0	19.61	5.88
3.51	0.88	0	0.88	0.88	0	0	5.26	0.88
3.23	1.08	0	1.08	0	1.08	0	2.15	0
0	0	0	0	0	0	0	0	0
5.56	0	0	0	0	0	0	5.56	0
0	4.55	4.55	0	0	0	0	9.09	0
2.01	1.34	0.67	0.67	0.67	0	0	4.03	1.34
3.78	4.32	2.16	1.08	0.54	0	0	6.49	2.7
3.64	1.82	0	1.82	0	0	0	5.45	0
4.81	0.96	0.32	0.32	0.32	0	0	4.49	1.6
0	1.35	0	1.35	0	0	0	8.11	1.35
9.23	1.54	0	1.54	0	0	1.54	3.08	1.54
7.14	0	0	0	0	0	0	3.57	2.38
4	0	0	0	0	0	0	6	2
0	1.92	1.92	0	0	0	0	0	0
4.41	0	0	0	0	0	0	10.29	2.94

3.3	4.4	2.2	2.2	0	0	1.1	5.49	0
4.48	1.49	0	1.49	0	0	1.49	4.48	0
3.65	1.46	0.73	0.73	0.73	0	0	8.03	3.65
3.33	3.33	3.33	0	0	0	0	3.33	3.33
0	1.47	1.47	0	0	0	0	8.82	4.41
7.69	2.2	1.1	1.1	1.1	0	0	6.59	1.1
4.05	0	0	0	0	0	0	5.41	1.35
6.25	0	0	0	0	0	0	7.5	0
6.78	2.54	1.69	0.85	0.85	0	0	4.24	0.85
9.09	0	0	0	0	0	0	7.79	3.9
6.06	0	0	0	0	0	0	6.06	0
1.69	1.69	0	1.69	1.69	0	0	6.78	1.69
5.71	0	0	0	0	0	0	2.86	0
0	0	0	0	0	0	0	0	0
2.27	2.27	0	2.27	0	0	2.27	2.27	0
2.7	0	0	0	0	0	0	6.76	0
3.13	1.56	1.56	0	0	0	0	6.25	0
5.3	0.76	0.38	0.38	0	0	0.38	4.92	1.52
8.62	3.45	0	3.45	3.45	0	0	13.79	5.17
3.7	0	0	0	0	0	0	7.41	0
2.56	2.56	0	2.56	0	0	0	2.56	0
6.98	0	0	0	0	0	0	6.98	0
5.66	0.94	0.94	0	0	0	0	5.66	0.94
3.23	3.23	1.61	1.61	0	0	0	4.84	0
3.61	3.01	0.6	2.41	0	1.2	0.6	5.42	0
4.35	1.74	0	1.74	0.87	0	0	5.22	0.87
0	0	0	0	0	0	0	4.76	0
4.22	3.01	1.2	1.81	0	0	1.2	6.02	2.41
5.23	2.61	0.65	1.96	0	0.65	0	7.19	1.96
2.24	2.24	0.75	1.49	0	0	1.49	2.24	0.75
4.35	0	0	0	0	0	0	4.35	0
6.03	4.31	1.72	2.59	1.72	0	0	2.59	0
0	1.85	1.85	0	0	0	0	9.26	5.56
0	5.26	5.26	0	0	0	0	0	0
4.81	2.88	2.88	0	0	0	0	6.73	0.96
10.48	0.81	0.81	0	0	0	0	5.65	1.61
6.25	1.04	1.04	0	0	0	0	3.13	1.04
0	0	0	0	0	0	0	0	0
4.72	1.57	1.57	0	0	0	0	4.72	0
2.78	0.69	0.69	0	0	0	0	6.94	2.78
2.44	0	0	0	0	0	0	7.32	2.44
7.32	1.22	0	1.22	0	0	0	6.1	0
7.14	0	0	0	0	0	0	7.14	0
7.25	1.45	0	1.45	1.45	0	0	4.35	0
4.51	1.5	0.75	0.75	0.75	0	0	6.02	0.75
3.48	1.74	0	1.74	0.87	0	0	3.48	0
3.28	4.92	0	4.92	0	0	3.28	3.28	0
0	9.09	0	9.09	9.09	0	0	0	0
0	7.14	7.14	0	0	0	0	0	0
3.13	6.25	3.13	3.13	3.13	0	0	6.25	0

4.65	4.65	0	4.65	4.65	0	0	4.65	2.33
4.69	1.56	0	1.56	1.56	0	0	6.25	0
2.94	0	0	0	0	0	0	0.98	0.98
3.94	1.48	0.49	0.99	0.49	0	0.49	9.85	0
4.17	0	0	0	0	0	0	6.25	0
2.7	1.35	0	1.35	0	1.35	0	6.76	0
1.37	5.48	0	5.48	4.11	0	0	8.22	0
8.2	3.28	0	3.28	3.28	0	0	4.92	0
6.05	1.86	0	1.86	1.4	0	0	6.05	1.86
3.85	2.56	0	2.56	0	0	1.28	5.13	0
3.3	0.88	0	0.88	0.66	0	0	7.03	0.88
0	4.35	0	4.35	0	0	0	13.04	4.35
0	5.56	0	5.56	0	0	0	5.56	0
13.89	8.33	0	5.56	2.78	0	0	8.33	2.78
7.69	7.69	0	7.69	0	0	5.13	7.69	2.56
25	0	0	0	0	0	0	0	0
3.23	3.23	0	3.23	0	0	0	16.13	3.23
10.87	2.17	0	2.17	0	0	0	4.35	0
0	0	0	0	0	0	0	3.13	0
7.41	3.7	0	3.7	0	0	0	3.7	0
8.89	0	0	0	0	0	0	10	1.11
3.13	4.69	0	4.69	3.13	0	1.56	3.13	0
0	3.45	0	3.45	0	0	0	6.9	3.45
4	0	0	0	0	0	0	4	4
0	4.55	0	2.27	0	0	0	11.36	0
0	3.57	0	3.57	3.57	0	0	14.29	0
1.75	0	0	0	0	0	0	0	0
5.26	10.53	0	10.53	5.26	5.26	0	15.79	5.26
0	5.5	1.83	3.67	0.92	1.83	0.92	7.34	1.83
0	5.88	5.88	0	0	0	0	8.82	5.88
0	0	0	0	0	0	0	0	0
8.47	0	0	0	0	0	0	6.78	1.69
1.92	5.77	0	5.77	5.77	0	0	5.77	1.92
6.93	1.98	0.99	0.99	0	0	0	10.89	3.96
5.22	1.74	1.74	0	0	0	0	7.83	1.74
22.22	0	0	0	0	0	0	0	0
4.44	6.67	4.44	2.22	0	0	0	13.33	8.89
4.44	6.67	4.44	2.22	0	0	0	13.33	8.89
0	1.85	0	1.85	1.85	0	0	5.56	1.85
2.5	7.5	2.5	2.5	0	0	0	12.5	7.5
5.15	4.12	2.06	2.06	2.06	0	0	6.19	2.06
4.17	2.08	0	2.08	2.08	0	0	14.58	6.25
11.36	2.27	0	2.27	0	0	0	2.27	0
2.13	4.26	2.13	2.13	2.13	0	0	6.38	4.26
6.12	4.08	4.08	0	0	0	0	0	0
9.52	4.76	4.76	0	0	0	0	23.81	4.76
9.38	3.13	3.13	0	0	0	0	12.5	9.38
0	0	0	0	0	0	0	5.26	0
1.19	0	0	0	0	0	0	8.33	0
0	0	0	0	0	0	0	8.11	2.7

3.03	9.09	6.06	3.03	0	0	0	3.03	3.03
0	15.79	7.89	5.26	2.63	0	0	5.26	5.26
5.36	3.57	1.79	1.79	0	0	0	5.36	3.57
5.96	1.32	0	1.32	0	0.66	0.66	5.96	0.66
0	6.67	0	6.67	0	0	0	0	0
5	0.83	0	0	0	0	0	6.67	0.83
3.33	4.44	2.22	2.22	0	0	0	5.56	2.22
2.78	11.11	2.78	8.33	2.78	2.78	0	5.56	2.78
3.64	3.64	1.82	1.82	0	0	0	7.27	5.45
0	0	0	0	0	0	0	0	0
2.94	5.88	5.88	0	0	0	0	5.88	2.94
0	12.5	6.25	6.25	0	0	0	6.25	0
0	4.17	2.08	2.08	2.08	0	0	12.5	4.17
6.67	0	0	0	0	0	0	0	0
7.14	0	0	0	0	0	0	0	0
0	5.26	5.26	0	0	0	0	0	0
2.86	8.57	0	5.71	2.86	2.86	0	5.71	2.86
4	0	0	0	0	0	0	4	4
14.81	0	0	0	0	0	0	7.41	0
3.38	2.9	0.48	1.93	0.48	0.97	0.48	5.31	0.48
0	12.5	12.5	0	0	0	0	0	0
0	0	0	0	0	0	0	13.33	0
7.14	0	0	0	0	0	0	7.14	0
11.11	0	0	0	0	0	0	11.11	0
1.69	3.39	1.69	1.69	0	0	0	8.47	1.69
0	0	0	0	0	0	0	3.33	0
20	0	0	0	0	0	0	0	0
0	1.45	1.45	0	0	0	0	5.8	2.9
0	9.09	5.45	3.64	0	0	0	7.27	7.27
4.55	1.95	0.65	1.3	0	0.65	0.65	9.09	2.6
5.26	5.26	2.63	2.63	0	0	0	13.16	2.63
0	12.5	0	12.5	12.5	0	12.5	12.5	0
0	5.88	5.88	0	0	0	0	5.88	5.88
6.85	0	0	0	0	0	0	8.22	0
20	0	0	0	0	0	0	15	0
0	11.11	11.11	0	0	0	0	0	0
0	12.5	6.25	6.25	0	0	0	6.25	6.25
0	2.5	0	2.5	0	0	0	7.5	5
11.11	11.11	11.11	0	0	0	0	22.22	11.11
2.27	2.27	0	2.27	0	0	0	9.09	2.27
1.3	0	0	0	0	0	0	7.79	3.9
5.88	5.88	0	3.92	0	0	1.96	5.88	1.96
3.7	0	0	0	0	0	0	9.26	5.56
2.94	0	0	0	0	0	0	8.82	2.94
0	0	0	0	0	0	0	0	0
0	4	0	4	0	0	0	0	0
2.7	0	0	0	0	0	0	5.41	1.35
0	6.25	0	6.25	0	6.25	0	0	0
0	11.11	0	11.11	0	0	0	11.11	11.11
3.33	0	0	0	0	0	0	6.67	3.33

0	2.82	1.41	1.41	0	0	0	5.63	0
0	0	0	0	0	0	0	0	0
0	7.69	0	7.69	0	0	0	7.69	0
6	2	0	2	0	0	0	10	4
7.06	2.35	1.18	1.18	1.18	0	0	3.53	1.18
4.17	0	0	0	0	0	0	8.33	0
3.77	4.72	0.94	3.77	1.89	1.89	0	4.72	0.94
4.69	6.25	1.56	3.13	1.56	1.56	0	15.63	7.81
5.56	0	0	0	0	0	0	6.94	2.78
5.56	0	0	0	0	0	0	6.94	2.78
3.7	1.85	0	1.85	0	0	0	5.56	5.56
3.49	3.49	0	3.49	0	1.16	1.16	5.81	1.16
6.1	0.81	0	0.81	0	0.41	0.41	8.94	3.66
5.77	0	0	0	0	0	0	9.62	0
4.92	0	0	0	0	0	0	13.11	6.56
6.75	1.59	0	1.59	1.19	0	0.4	5.95	1.59
10	0	0	0	0	0	0	20	10
0	0	0	0	0	0	0	13.33	6.67
4.26	2.13	0	2.13	2.13	0	0	6.38	0
0	0	0	0	0	0	0	17.65	14.71
1.59	6.35	1.59	4.76	1.59	0	0	6.35	0
16.67	0	0	0	0	0	0	0	0
3.85	3.85	0	3.85	3.85	0	0	3.85	3.85
6.76	1.93	0.48	0.97	0.97	0	0	5.31	1.45
4.69	4.69	3.13	1.56	0	0	0	6.25	0
4.62	1.54	1.54	0	0	0	0	3.08	0
3.23	3.23	3.23	0	0	0	0	3.23	0
2.04	0	0	0	0	0	0	8.16	2.04
6.62	2.65	1.32	0.66	0	0	0	8.61	1.99
2.17	0	0	0	0	0	0	6.52	0
0	0	0	0	0	0	0	3.23	3.23
6.25	2.34	0	2.34	1.56	0	1.56	8.59	2.34
2.63	0.75	0.75	0	0	0	0	5.64	0.75
3.51	7.02	3.51	2.63	0.88	0	1.75	4.39	2.63
6.02	1.2	0	0.6	0	0	0	4.22	1.2
1.39	4.17	4.17	0	0	0	0	8.33	1.39
0	6.67	0	6.67	0	0	0	0	0
0	6.67	3.33	3.33	3.33	0	0	16.67	6.67
4.44	2.22	2.22	0	0	0	0	8.89	4.44
0	6.78	1.69	3.39	0	1.69	0	6.78	1.69
4	4	4	0	0	0	0	12	0
3.03	3.03	0	3.03	3.03	0	0	12.12	9.09
0	2.38	2.38	0	0	0	0	7.14	4.76
2.82	4.23	0	2.82	0	2.82	0	5.63	0
6.15	3.08	0	3.08	1.54	0	0	7.69	3.08
2.86	0	0	0	0	0	0	8.57	0
3.23	1.61	1.61	0	0	0	0	8.06	1.61
9.09	0	0	0	0	0	0	9.09	0
5.26	0	0	0	0	0	0	5.26	0
0	3.45	0	3.45	0	0	0	6.9	0

2.6	0	0	0	0	0	0	10.39	2.6
4.35	2.17	2.17	0	0	0	0	6.52	0
2.9	2.9	1.45	0	0	0	0	13.04	5.8
6.67	0	0	0	0	0	0	13.33	0
5.13	0	0	0	0	0	0	2.56	2.56
2.3	1.84	0.46	1.38	0.46	0.46	0	6.91	0.46
4.64	1.32	0	1.32	0	0	0	3.97	0.66
4.03	1.21	0	1.21	0.81	0	0	3.63	0.4
4	4	0	4	0	4	0	4	0
4.76	4.76	2.38	2.38	2.38	0	0	0	0
6	2	0	2	0.67	0.67	0	8	0
3.6	2.88	0.72	2.16	0.72	0	0.72	6.47	2.16
2.44	7.32	4.88	2.44	2.44	0	0	0	0
2.47	4.94	1.23	3.7	0	2.47	0	7.41	0
3.88	0.97	0	0.97	0	0	0	8.74	0.97
3.36	2.52	0	2.52	0	1.68	0	10.08	1.68
6	0	0	0	0	0	0	9	4
10.64	2.13	1.06	1.06	1.06	0	0	10.64	4.26
0	12.5	0	0	0	0	0	50	37.5
4.35	0	0	0	0	0	0	4.35	0
2.83	0.94	0	0.94	0	0.94	0	4.72	0
3.13	3.13	0	3.13	0	0	0	6.25	0
0	0	0	0	0	0	0	7.69	7.69
7.14	0	0	0	0	0	0	10.71	0
11.11	11.11	11.11	0	0	0	0	5.56	5.56
2.33	0	0	0	0	0	0	4.65	0
0	2.94	0	2.94	0	0	0	8.82	0
0	0	0	0	0	0	0	13.64	0
1.89	2.83	0	2.83	0.94	0	0.94	7.55	0
3.57	0	0	0	0	0	0	0	0
5.56	0	0	0	0	0	0	5.56	5.56
2.81	1.69	1.12	0.56	0	0	0.56	8.99	0
8.33	5	3.33	1.67	0	0	1.67	10	1.67
4.17	4.17	0	0	0	0	0	4.17	0
8.72	0.67	0.67	0	0	0	0	8.05	1.34
3.69	2.84	0.57	1.7	0	0.28	0	5.68	0.85
4.62	1.54	1.54	0	0	0	0	7.69	0
3.68	2.21	0.74	0.74	0	0.74	0	4.41	0.74
3.85	0	0	0	0	0	0	3.85	0
4.76	1.19	0	1.19	0	0	0	10.71	1.19
4.05	8.11	4.05	4.05	0	2.7	0	5.41	1.35
6.9	5.17	1.72	3.45	0	3.45	0	8.62	0
1.39	6.94	4.17	1.39	0	1.39	0	11.11	4.17
0	9.09	0	9.09	3.03	3.03	0	12.12	3.03
5.85	2.34	0.58	0.58	0.58	0	0	2.34	1.75
4.14	5.52	2.07	2.07	0.69	1.38	0	3.45	2.07
4.76	2.38	0	0	0	0	0	4.76	0
3.6	0.9	0	0.9	0	0.9	0	6.31	1.8
0	0	0	0	0	0	0	0	0
3.85	1.28	0	0	0	0	0	6.41	2.56

5.56	0	0	0	0	0	0	16.67	5.56
3.03	6.06	3.03	0	0	0	0	6.06	6.06
4.88	0	0	0	0	0	0	2.44	0
0	2.9	2.9	0	0	0	0	1.45	0
0	50	50	0	0	0	0	0	0
3.45	0	0	0	0	0	0	4.6	0
1.32	2.63	0	0	0	0	0	2.63	1.32
0	0	0	0	0	0	0	0	0
4.62	3.08	0	1.54	0	1.54	0	6.15	1.54
0	0	0	0	0	0	0	5.41	0
0	0	0	0	0	0	0	3.23	0
1.96	0.98	0.98	0	0	0	0	1.96	0
7.69	5.49	1.1	4.4	0	3.3	1.1	5.49	0
7.35	2.94	1.47	1.47	0	0	0	1.47	0
5.93	1.69	0.85	0.85	0	0.85	0	4.24	0
10	0	0	0	0	0	0	10	2.5
0	0	0	0	0	0	0	2.22	2.22
0	0	0	0	0	0	0	0	0
6.82	0	0	0	0	0	0	2.27	2.27
8.7	0	0	0	0	0	0	4.35	0
5.26	0	0	0	0	0	0	3.95	1.32
4.49	1.12	0	1.12	0	0.56	0	7.3	1.69
4.92	3.28	3.28	0	0	0	0	8.2	3.28
1.89	0	0	0	0	0	0	5.66	0
3.45	5.17	0	5.17	0	3.45	0	5.17	0
0	0	0	0	0	0	0	0	0
12	0	0	0	0	0	0	12	0
3.13	1.04	1.04	0	0	0	0	4.17	1.04
3.7	0	0	0	0	0	0	11.11	3.7
2.88	0.96	0.96	0	0	0	0	14.42	5.77
3.13	3.13	3.13	0	0	0	0	3.13	3.13
0	0	0	0	0	0	0	8.33	0
4.31	0.86	0.86	0	0	0	0	1.72	0
8.33	2.08	0	2.08	0	0	2.08	6.25	0
6.8	0.97	0.97	0	0	0	0	6.8	1.94
0	9.38	0	9.38	0	0	6.25	0	0
3.51	5.26	0	1.75	1.75	0	1.75	8.77	5.26
0	0	0	0	0	0	0	9.09	0
4.81	1.92	0.96	0.96	0	0.96	0	13.46	2.88
12.5	0	0	0	0	0	0	12.5	0
5	0	0	0	0	0	0	7.5	2.5
6.14	1.46	0.29	1.17	0.29	0	0.29	3.51	0.88
3.77	1.89	1.89	0	0	0	0	5.66	1.89
0	9.09	9.09	0	0	0	0	0	0
2.9	1.45	0	1.45	0	0	0	7.25	0
1.49	0	0	0	0	0	0	5.97	2.99
6.25	0	0	0	0	0	0	0	0
0	5.88	0	5.88	0	0	0	0	0
1.79	0	0	0	0	0	0	12.5	1.79
4.65	4.65	2.33	2.33	0	2.33	0	6.98	0

0	0	0	0	0	0	0	7.69	7.69
0	0	0	0	0	0	0	9.09	9.09
0	2.82	0	2.82	0	2.82	0	4.23	1.41
3.7	0	0	0	0	0	0	3.7	1.85
5.56	4.44	2.22	2.22	0	1.11	0	7.78	4.44
15.38	0	0	0	0	0	0	7.69	0
0	0	0	0	0	0	0	0	0
6.33	5.06	1.27	3.8	1.27	2.53	0	13.92	7.59
0	0	0	0	0	0	0	7.69	0
5.49	1.1	1.1	0	0	0	0	5.49	2.2
8.16	4.08	4.08	0	0	0	0	6.12	0
4.17	4.17	4.17	0	0	0	0	4.17	0
0	0	0	0	0	0	0	0	0
0	4.35	4.35	0	0	0	0	4.35	4.35
0	0	0	0	0	0	0	0	0
4.55	0	0	0	0	0	0	9.09	0
5.51	1.84	0	1.1	0	0.74	0.37	7.35	1.47
3.03	0	0	0	0	0	0	3.03	0
5	0	0	0	0	0	0	15	0
0	0	0	0	0	0	0	7.14	0
7.5	0	0	0	0	0	0	10	5
3.85	0	0	0	0	0	0	7.69	3.85
7.58	0	0	0	0	0	0	1.52	0
0	0	0	0	0	0	0	12.5	0
4.55	2.27	0	2.27	2.27	0	0	2.27	0
3.7	1.85	0.93	0.93	0	0	0	3.7	0
9.47	2.11	0.53	0.53	0	0	0	3.68	2.11
10	0	0	0	0	0	0	0	0
5	0	0	0	0	0	0	0	0
5	0	0	0	0	0	0	0	0
0	0	0	0	0	0	0	0	0
0	0	0	0	0	0	0	8.7	0
7.41	0	0	0	0	0	0	1.85	0
0	0	0	0	0	0	0	0	0
8.33	8.33	0	8.33	0	8.33	0	8.33	0
0	0	0	0	0	0	0	100	0
14.29	0	0	0	0	0	0	0	0
9.09	0	0	0	0	0	0	9.09	0
0	0	0	0	0	0	0	0	0
8.33	8.33	0	8.33	0	0	8.33	8.33	0
10	0	0	0	0	0	0	3.33	0
1.82	1.82	0	1.82	0	0	0	5.45	0
4.35	1.45	0	1.45	0	0	0	5.8	0
0	6.67	6.67	0	0	0	0	6.67	0
11.11	0	0	0	0	0	0	0	0
4.88	7.32	0	7.32	0	0	2.44	14.63	9.76
5.56	0	0	0	0	0	0	16.67	0
4.71	1.18	0	1.18	0	0	0	5.88	2.35
2.56	0.85	0	0.85	0.85	0	0	10.26	0.85
6.9	3.45	3.45	0	0	0	0	0	0

12.5	3.13	0	3.13	3.13	0	0	3.13	0
6.85	0	0	0	0	0	0	8.22	4.11
4.62	1.54	0	1.54	0	0	0	3.08	0
2.56	12.82	5.13	7.69	0	2.56	2.56	10.26	5.13
4.55	13.64	0	13.64	0	9.09	4.55	0	0
4.67	2	1.33	0.67	0.67	0	0	7.33	2
8.93	1.79	1.79	0	0	0	0	7.14	3.57
5.77	0.96	0	0.96	0	0	0	4.81	0.96
0	7.69	5.13	2.56	2.56	0	0	2.56	0
4.76	0	0	0	0	0	0	0	0
0	0	0	0	0	0	0	9.09	4.55
5.8	0	0	0	0	0	0	7.25	1.45
1.45	4.35	1.45	2.9	0	1.45	1.45	5.8	1.45
11.11	0	0	0	0	0	0	8.33	2.78
10	8	0	8	2	0	0	12	2
7.37	3.16	0	3.16	1.05	2.11	0	7.37	2.11
5.51	3.94	0.79	3.15	1.57	0	0.79	3.94	0
9.26	1.85	0	1.85	1.85	0	0	5.56	1.85
6.09	3.48	0.87	2.61	0	0	0	6.09	1.74
2.17	0	0	0	0	0	0	6.52	0
0	0	0	0	0	0	0	4	0
8.26	3.67	0	1.83	0.92	0	0.92	6.42	0.92
5.66	3.77	0	3.77	3.77	0	0	3.77	0
0	0	0	0	0	0	0	0	0
5	2.5	0	2.5	0	2.5	0	15	0
2.56	2.56	0	2.56	2.56	0	0	12.82	0
5.88	0	0	0	0	0	0	0	0
7.14	7.14	0	7.14	0	7.14	0	14.29	0
12.5	0	0	0	0	0	0	25	0
6.06	0	0	0	0	0	0	3.03	0
8.57	2.86	0	2.86	2.86	0	0	8.57	0
0	1.96	0	1.96	0	0	1.96	3.92	0
4.17	0	0	0	0	0	0	8.33	0
14.29	2.38	2.38	0	0	0	0	9.52	7.14
12.5	12.5	0	12.5	0	0	0	12.5	0
9.23	1.54	0	1.54	0	0	0	4.62	1.54
11.76	2.94	2.94	0	0	0	0	10.29	5.88
3.23	3.23	1.61	1.61	1.61	0	0	9.68	1.61
4.76	0	0	0	0	0	0	14.29	4.76
5.56	0	0	0	0	0	0	5.56	2.78
0	6.12	4.08	2.04	0	0	2.04	6.12	2.04
0	0	0	0	0	0	0	9.09	0
0	8.51	2.13	6.38	0	0	2.13	6.38	0
0	4.55	0	4.55	0	0	4.55	4.55	0
13.04	0	0	0	0	0	0	17.39	17.39
5.26	0	0	0	0	0	0	5.26	5.26
0	0	0	0	0	0	0	10	0
4	0	0	0	0	0	0	8	0
7.69	0	0	0	0	0	0	2.56	2.56
0	0	0	0	0	0	0	10	0

7.84	1.96	0	1.96	0	1.96	0	7.84	1.96
0.86	0.86	0.86	0	0	0	0	6.9	1.72
5.26	2.63	1.32	0	0	0	0	5.26	3.95
0	0	0	0	0	0	0	11.76	0
0	3.13	3.13	0	0	0	0	3.13	3.13
0	9.09	1.82	7.27	0	3.64	0	10.91	1.82
2.63	2.63	1.32	1.32	0	0	0	7.89	6.58
0	0	0	0	0	0	0	3.7	3.7
2.91	0.97	0	0.97	0	0.97	0	3.88	0
4.69	7.81	1.56	4.69	1.56	0	0	3.13	1.56
7.14	1.79	0	1.79	1.79	0	0	4.46	0.89
5.49	3.3	3.3	0	0	0	0	5.49	1.1
5.41	1.8	0	1.8	0	0	1.8	2.7	0
5.74	0.82	0	0.82	0.82	0	0	4.1	0.82
0	4.76	4.76	0	0	0	0	4.76	4.76
4.55	3.64	0	3.64	1.82	0	0	3.64	0
1.9	1.9	1.9	0	0	0	0	1.9	0
4.94	2.47	0	2.47	0	2.47	0	4.94	1.23
0	8.33	8.33	0	0	0	0	0	0
6.67	0	0	0	0	0	0	0	0
0	3.7	3.7	0	0	0	0	3.7	1.85
4.67	3.74	1.87	1.87	0	0	0	1.87	0.93
4.03	1.61	0	1.61	0	0	0	5.65	1.61
0	0	0	0	0	0	0	3.57	0
3.17	1.59	0.79	0.79	0	0	0	5.56	0.79
11.29	6.45	3.23	3.23	1.61	0	0	3.23	1.61
5.71	2.86	0	2.86	0	0	0	0	0
0	3.7	3.7	0	0	0	0	7.41	0
7.14	3.57	0	3.57	0	0	0	3.57	3.57
1.72	3.45	1.72	1.72	1.72	0	0	1.72	1.72
1.69	0	0	0	0	0	0	0	0
15	5	5	0	0	0	0	5	0
4.62	1.54	0	1.54	0	0	0	7.69	6.15
4.05	4.05	4.05	0	0	0	0	0	0
1.89	5.66	1.89	3.77	0	0	0	9.43	3.77
0	6.56	3.28	3.28	1.64	0	0	11.48	0
9.76	2.44	2.44	0	0	0	0	2.44	2.44
3.23	6.45	3.23	0	0	0	0	3.23	0
0	0	0	0	0	0	0	0	0
1.67	6.67	3.33	1.67	0	1.67	0	5	1.67
6.06	3.03	3.03	0	0	0	0	6.06	0
5.26	5.26	0	5.26	2.63	0	0	7.89	2.63
5.13	2.56	2.56	0	0	0	0	0	0
3.9	1.3	1.3	0	0	0	0	5.19	5.19
0	5.1	1.02	4.08	1.02	2.04	0	5.1	0
1.94	2.91	1.94	0.97	0	0	0.97	2.91	1.94
0	0	0	0	0	0	0	3.33	0
1	3	0	2	0	0	2	3	0
4.05	2.7	0	2.7	0	0	2.7	4.05	1.35
4.82	3.61	1.2	2.41	0	0	0	4.82	0

0	9.09	9.09	0	0	0	0	13.64	9.09
1.56	1.56	0	1.56	0	0	0	4.69	4.69
2.08	2.08	0	2.08	0	0	0	2.08	0
7.69	0	0	0	0	0	0	23.08	23.08
10	1.43	1.43	0	0	0	0	5.71	4.29
0	0	0	0	0	0	0	0	0
3.03	4.55	1.52	3.03	0	0	0	3.03	3.03
3.9	3.9	3.9	0	0	0	0	3.9	0
4.76	4.76	4.76	0	0	0	0	2.38	2.38
5.41	0	0	0	0	0	0	2.7	2.7
6.52	2.17	0	2.17	2.17	0	0	4.35	2.17
0	11.54	11.54	0	0	0	0	7.69	3.85
1.96	7.84	3.92	3.92	1.96	0	0	9.8	7.84
12	2	2	0	0	0	0	12	4
5.26	0	0	0	0	0	0	7.02	3.51
6.9	0	0	0	0	0	0	3.45	3.45
5.77	5.77	1.92	1.92	0	0	0	1.92	1.92
0	4.69	3.13	0	0	0	0	6.25	3.13
3.23	0	0	0	0	0	0	12.9	0
4.84	1.61	0	0	0	0	0	3.23	1.61
2	6	2	4	0	0	2	6	4
9.78	3.26	0	3.26	0	1.09	0	1.09	0
9.38	3.13	3.13	0	0	0	0	3.13	3.13
4.35	6.52	4.35	2.17	0	0	0	4.35	0
1.06	1.06	1.06	0	0	0	0	6.38	2.13
6.96	2.61	0	1.74	0	1.74	0	4.35	0
4.07	3.25	0.81	2.44	0.81	0	0.81	2.44	0.81
0	4.17	4.17	0	0	0	0	0	0
0	8	8	0	0	0	0	4	0
2.08	2.08	0	0	0	0	0	2.08	2.08
9.64	2.41	1.2	1.2	1.2	0	0	3.61	1.2
4.82	0	0	0	0	0	0	8.43	7.23
4.88	2.44	2.44	0	0	0	0	7.32	2.44
0	2.22	0	2.22	0	0	0	13.33	8.89
4.42	3.54	2.65	0.88	0	0.88	0	5.31	0.88
3.23	0	0	0	0	0	0	6.45	3.23
5.33	0	0	0	0	0	0	4	0
0	3.45	3.45	0	0	0	0	0	0
3.49	3.49	1.16	1.16	0	0	0	3.49	0
3.49	3.49	1.16	1.16	0	0	0	3.49	0
6.25	3.57	0.89	2.68	0.89	0	0.89	6.25	1.79
3.45	3.45	3.45	0	0	0	0	0	0
12	1.33	0	1.33	1.33	0	0	1.33	1.33
12.5	4.17	4.17	0	0	0	0	0	0
3.28	6.56	4.92	1.64	0	0	1.64	6.56	0
0	7.41	3.7	3.7	0	0	0	1.85	1.85
8.33	6.25	0	6.25	0	0	2.08	8.33	6.25
4.76	4.76	0	4.76	0	0	0	4.76	0
2.78	2.78	0	2.78	0	0	0	8.33	0
15.38	0	0	0	0	0	0	0	0

2.67	2.67	0	2.67	1.33	0	0	4	1.33
8.33	0	0	0	0	0	0	4.17	0
5	15	10	5	0	0	0	0	0
5	0	0	0	0	0	0	1.67	0
8.7	0	0	0	0	0	0	2.17	2.17
4.55	0	0	0	0	0	0	13.64	13.64
6.1	1.22	1.22	0	0	0	0	3.66	2.44
0	8.82	8.82	0	0	0	0	2.94	2.94
11.02	3.39	0.85	1.69	0	1.69	0	4.24	0.85
0	11.76	11.76	0	0	0	0	11.76	0
3.26	2.17	0	2.17	0	0	1.09	2.17	1.09
11.76	0	0	0	0	0	0	0	0
4.55	1.52	1.52	0	0	0	0	3.03	0
2.56	5.13	5.13	0	0	0	0	5.13	2.56
3.7	1.85	1.85	0	0	0	0	0	0
0	6.25	6.25	0	0	0	0	6.25	0
3.13	3.13	3.13	0	0	0	0	3.13	0
6.25	1.25	0	1.25	1.25	0	0	3.75	0
0	0	0	0	0	0	0	0	0
2.78	0	0	0	0	0	0	8.33	2.78
12.96	1.85	1.85	0	0	0	0	3.7	0
6.04	0	0	0	0	0	0	4.03	1.34
0	0	0	0	0	0	0	5	0
0	0	0	0	0	0	0	5	0
4.88	2.44	2.44	0	0	0	0	2.44	0
0	0	0	0	0	0	0	0	0
2.35	1.18	1.18	0	0	0	0	7.06	3.53
3.33	0	0	0	0	0	0	3.33	3.33
12.9	3.23	3.23	0	0	0	0	0	0
0	0	0	0	0	0	0	0	0
2.8	0.93	0	0.93	0	0	0	2.8	0
4.21	1.05	1.05	0	0	0	0	4.21	0
0	0	0	0	0	0	0	8.64	2.47
2.63	0	0	0	0	0	0	0	0
0	0	0	0	0	0	0	5.41	5.41
4.46	0	0	0	0	0	0	2.68	0
8.33	0	0	0	0	0	0	0	0
4.76	0	0	0	0	0	0	4.76	0
9.09	0	0	0	0	0	0	2.27	0
2.44	0	0	0	0	0	0	0	0
7.5	0	0	0	0	0	0	2.5	0
5.26	0	0	0	0	0	0	8.27	0.75
2.56	0	0	0	0	0	0	5.13	0
1.89	0	0	0	0	0	0	3.77	0
0	0	0	0	0	0	0	6.25	1.56
2.94	0	0	0	0	0	0	5.88	0
8.33	0	0	0	0	0	0	0	0
4.92	4.92	0	4.92	0	3.28	0	3.28	0
0	12.5	0	12.5	0	0	0	0	0
6	2	1	1	1	0	0	2	0

3.57	1.19	1.19	0	0	0	0	7.14	0
3.57	0	0	0	0	0	0	0	0
2.22	0	0	0	0	0	0	2.22	0
3.92	1.96	0	0	0	0	0	9.8	0
9.09	0	0	0	0	0	0	0	0
7.89	2.63	2.63	0	0	0	0	2.63	0
10	0	0	0	0	0	0	10	0
3.45	0	0	0	0	0	0	0	0
2.94	0	0	0	0	0	0	0	0
0	11.11	11.11	0	0	0	0	11.11	0
7.23	0	0	0	0	0	0	3.61	0
0	0	0	0	0	0	0	0	0
5.56	0	0	0	0	0	0	5.56	0
3.23	0	0	0	0	0	0	3.23	0
4.26	0	0	0	0	0	0	2.13	0
1.72	0	0	0	0	0	0	0	0
6.12	0	0	0	0	0	0	4.08	1.02
0	0	0	0	0	0	0	4	0
2.86	0	0	0	0	0	0	11.43	0
7.69	0	0	0	0	0	0	0	0
0	0	0	0	0	0	0	0	0
7.69	7.69	0	7.69	0	0	0	7.69	0
4.08	0	0	0	0	0	0	4.08	0
12.5	6.25	6.25	0	0	0	0	0	0
7.69	0	0	0	0	0	0	0	0
4.26	0	0	0	0	0	0	6.38	0
6.9	0	0	0	0	0	0	3.45	0
6.06	0	0	0	0	0	0	3.03	0
6.67	0	0	0	0	0	0	10	0
9.52	0	0	0	0	0	0	0	0
6.67	0	0	0	0	0	0	0	0
4.21	2.34	0.47	1.87	0.47	0.47	0	4.67	0.93
5.56	0	0	0	0	0	0	2.78	0.93
3.39	1.69	0	1.69	0	0	1.69	3.39	1.69
4	2	2	0	0	0	0	0	0
10.34	0	0	0	0	0	0	3.45	3.45
9.09	0	0	0	0	0	0	0	0
5	0	0	0	0	0	0	0	0
3.57	0	0	0	0	0	0	0	0
6.25	0	0	0	0	0	0	3.13	0
0	0	0	0	0	0	0	5.26	5.26
0	0	0	0	0	0	0	4.55	2.27
1.82	0	0	0	0	0	0	3.64	0
6.25	0	0	0	0	0	0	0	0
4.35	0	0	0	0	0	0	0	0
0	3.03	0	3.03	3.03	0	0	0	0
0	0	0	0	0	0	0	0	0
8.22	1.37	1.37	0	0	0	0	0	0
7.32	0	0	0	0	0	0	0	0
8.33	0	0	0	0	0	0	0	0

3.7	0	0	0	0	0	0	0	0
0	0	0	0	0	0	0	3.33	0
12.5	0	0	0	0	0	0	3.13	0
3.24	0.36	0.36	0	0	0	0	1.08	0
12	0	0	0	0	0	0	6	0
1.85	1.85	0	1.85	0	0	0	1.85	0
0	0	0	0	0	0	0	11.11	0
30	0	0	0	0	0	0	10	5
0	0	0	0	0	0	0	0	0
4.05	0	0	0	0	0	0	1.35	0
0	0	0	0	0	0	0	0	0
5	0	0	0	0	0	0	10	0
4.23	1.41	0	1.41	0	0	0	2.82	2.82
5.41	2.7	2.7	0	0	0	0	0	0
2.44	2.44	0	2.44	2.44	0	0	0	0
0	0	0	0	0	0	0	0	0
8.57	0	0	0	0	0	0	1.43	0
0	0	0	0	0	0	0	0	0
3.03	0	0	0	0	0	0	1.52	0
0	0	0	0	0	0	0	3.57	0
0	0	0	0	0	0	0	11.11	11.11
0	0	0	0	0	0	0	0	0
6.25	0	0	0	0	0	0	0	0
0	0	0	0	0	0	0	3.7	0
3.23	0	0	0	0	0	0	0	0
9.76	0	0	0	0	0	0	2.44	0
0	0	0	0	0	0	0	0	0
0	0	0	0	0	0	0	0	0
0	0	0	0	0	0	0	0	0
5.88	0	0	0	0	0	0	0	0
5	0	0	0	0	0	0	2.5	0
12.5	0	0	0	0	0	0	0	0
5.7	0	0	0	0	0	0	3.16	1.27
5.08	1.69	1.69	0	0	0	0	5.08	1.69
9.09	0	0	0	0	0	0	6.82	0
8.51	0	0	0	0	0	0	6.38	0
6.62	0	0	0	0	0	0	5.15	2.21
7.02	0.88	0.88	0	0	0	0	1.75	0.88
10	0	0	0	0	0	0	0	0
2.97	0.99	0.99	0	0	0	0	8.91	1.98
6.25	0	0	0	0	0	0	12.5	6.25
9.52	0	0	0	0	0	0	4.76	0
8	4	4	0	0	0	0	0	0
7.87	0	0	0	0	0	0	8.99	0
2.33	3.49	0	3.49	1.16	1.16	0	0	0
5.06	1.27	1.27	0	0	0	0	1.27	0
5.88	0	0	0	0	0	0	5.88	5.88
28.57	0	0	0	0	0	0	0	0
2.22	0	0	0	0	0	0	11.11	6.67
4.76	0	0	0	0	0	0	4.76	0

8.82	0	0	0	0	0	0	2.94	2.94
4	1.33	0	1.33	0	0	0	2.67	1.33
0	8.33	8.33	0	0	0	0	0	0
4.44	2.22	2.22	0	0	0	0	0	0
0	5.41	0	5.41	2.7	0	0	0	0
2.7	2.7	0	2.7	0	0	0	5.41	0
0	0	0	0	0	0	0	2.38	0
0	0	0	0	0	0	0	0	0
8	0	0	0	0	0	0	0	0
9.52	0	0	0	0	0	0	0	0
2.56	0	0	0	0	0	0	0	0
2.94	0	0	0	0	0	0	1.47	0
5.88	0	0	0	0	0	0	2.94	0
0	0	0	0	0	0	0	4.55	0
0	0	0	0	0	0	0	0	0
3.03	3.03	0	0	0	0	0	6.06	3.03
11.11	0	0	0	0	0	0	0	0
3.57	1.79	1.79	0	0	0	0	3.57	0
0	0	0	0	0	0	0	11.11	0
0	0	0	0	0	0	0	0	0
0	0	0	0	0	0	0	0	0
9.09	0	0	0	0	0	0	9.09	0
3.7	0	0	0	0	0	0	0	0
3.13	0	0	0	0	0	0	6.25	3.13
0	4.55	4.55	0	0	0	0	4.55	4.55
4.76	0	0	0	0	0	0	0	0
4.76	0	0	0	0	0	0	4.76	0
0	0	0	0	0	0	0	28.57	0
8	0	0	0	0	0	0	4	0
0	0	0	0	0	0	0	0	0
3.5	0	0	0	0	0	0	2.8	0
6.25	0	0	0	0	0	0	12.5	0
15.79	0	0	0	0	0	0	15.79	5.26
0	0	0	0	0	0	0	6.67	0
9.09	0	0	0	0	0	0	9.09	0
12.5	0	0	0	0	0	0	2.5	0
0	0	0	0	0	0	0	0	0
2.38	0	0	0	0	0	0	7.14	2.38
0	0	0	0	0	0	0	6.45	0
4.69	0.78	0.78	0	0	0	0	6.25	1.56
25	0	0	0	0	0	0	0	0
4.35	0	0	0	0	0	0	8.7	0
0	0	0	0	0	0	0	0	0
0	0	0	0	0	0	0	0	0
4.31	0.86	0.86	0	0	0	0	4.31	0
0	0	0	0	0	0	0	6.67	0
0	0	0	0	0	0	0	3.57	0
7.14	0	0	0	0	0	0	7.14	0
7.14	0	0	0	0	0	0	7.14	0
7.69	0	0	0	0	0	0	0	0

0	0	0	0	0	0	0	0	0
0	0	0	0	0	0	0	0	0
4.76	2.38	0	0	0	0	0	0	0
8.7	0	0	0	0	0	0	8.7	0
1.72	1.72	0	1.72	0	0	1.72	3.45	0
4.76	0	0	0	0	0	0	2.38	0
2.38	0	0	0	0	0	0	2.38	0
6.92	0	0	0	0	0	0	5.38	2.31
0	0	0	0	0	0	0	6.67	0
0	0	0	0	0	0	0	0	0
6.67	0	0	0	0	0	0	2.22	2.22
0	6.25	6.25	0	0	0	0	0	0
4.23	2.82	2.82	0	0	0	0	5.63	0
3.77	0	0	0	0	0	0	1.89	1.89
12.5	12.5	12.5	0	0	0	0	12.5	0
0	10	10	0	0	0	0	10	0
0	0	0	0	0	0	0	6.25	0
2.78	0	0	0	0	0	0	0	0
0	0	0	0	0	0	0	0	0
10.81	0	0	0	0	0	0	0	0
8.47	1.69	1.69	0	0	0	0	6.78	3.39
2.94	0	0	0	0	0	0	2.94	0
3.06	2.04	0	2.04	1.02	0	0	1.02	0
4.76	0	0	0	0	0	0	0	0
0	0	0	0	0	0	0	0	0
15.38	0	0	0	0	0	0	0	0
7.69	2.56	2.56	0	0	0	0	15.38	7.69
0	0	0	0	0	0	0	0	0
6.9	0	0	0	0	0	0	3.45	3.45
3.85	0.7	0	0.7	0.35	0.35	0	3.15	0.35
6.67	0	0	0	0	0	0	0	0
0	0	0	0	0	0	0	0	0
10	0	0	0	0	0	0	0	0
3.23	0	0	0	0	0	0	0	0
0	0	0	0	0	0	0	13.89	2.78
0	0	0	0	0	0	0	0	0
14.29	14.29	14.29	0	0	0	0	14.29	14.29
7.74	0	0	0	0	0	0	1.79	0
5.16	0	0	0	0	0	0	3.23	1.29
6.52	0	0	0	0	0	0	0	0
1.54	0	0	0	0	0	0	1.54	0
0	0	0	0	0	0	0	0	0
4	0	0	0	0	0	0	4	0
15.38	0	0	0	0	0	0	0	0
14.29	0	0	0	0	0	0	0	0
0	0	0	0	0	0	0	0	0
3.45	0	0	0	0	0	0	6.9	4.6
2.63	0	0	0	0	0	0	0	0
9.59	0	0	0	0	0	0	2.74	0
7.86	0	0	0	0	0	0	3.57	0

1.75	3.51	1.75	1.75	0	0	0	3.51	0
7.46	0	0	0	0	0	0	7.46	0
8.7	0	0	0	0	0	0	1.45	1.45
6.6	0	0	0	0	0	0	0	0
10.71	0	0	0	0	0	0	0	0
1.32	1.32	1.32	0	0	0	0	10.53	5.26
12	0	0	0	0	0	0	0	0
2.2	0	0	0	0	0	0	4.4	1.1
2.33	2.33	0	2.33	2.33	0	0	0	0
0	2.7	0	2.7	0	0	0	5.41	0
7.5	0	0	0	0	0	0	5	2.5
8.33	0	0	0	0	0	0	4.17	0
2.94	0	0	0	0	0	0	0	0
3.23	0	0	0	0	0	0	3.23	0
0	0	0	0	0	0	0	0	0
0	0	0	0	0	0	0	0	0
0	0	0	0	0	0	0	0	0
0	0	0	0	0	0	0	2.41	0
7.84	0	0	0	0	0	0	0	0
1.45	0	0	0	0	0	0	0	0
4.08	1.02	1.02	0	0	0	0	3.06	0
0	0	0	0	0	0	0	0	0
3.77	0.63	0.63	0	0	0	0	8.81	4.4
6.1	1.22	0	1.22	1.22	0	0	1.22	0
5	0	0	0	0	0	0	0	0
6.06	0	0	0	0	0	0	3.03	0
3.16	1.05	1.05	0	0	0	0	6.32	3.16
1.75	0	0	0	0	0	0	3.51	0
0	3.7	3.7	0	0	0	0	0	0
1.41	1.41	1.41	0	0	0	0	0	0
6.8	0.97	0	0.97	0	0	0	4.85	0
0	0	0	0	0	0	0	0	0
4.35	0	0	0	0	0	0	0	0
5.26	0	0	0	0	0	0	5.26	0
1.28	2.56	2.56	0	0	0	0	2.56	0
0	0	0	0	0	0	0	14.29	4.76
0	0	0	0	0	0	0	0	0
2.86	5.71	2.86	2.86	2.86	0	0	2.86	2.86
4.55	1.82	0.91	0.91	0.91	0	0	6.36	0
4.7	0.67	0	0	0	0	0	5.37	0
11.76	0	0	0	0	0	0	2.94	2.94
10.45	1.49	1.49	0	0	0	0	5.97	2.99
0	0	0	0	0	0	0	0	0
27.27	0	0	0	0	0	0	9.09	0
4.05	0	0	0	0	0	0	5.41	0
0.73	0	0	0	0	0	0	4.38	1.46
3.77	0	0	0	0	0	0	5.66	1.89
3.33	0	0	0	0	0	0	6.67	0
9.09	0	0	0	0	0	0	4.55	0
0	0	0	0	0	0	0	0	0

0	10	10	0	0	0	0	0	0
2	2	0	2	2	0	0	6	2
0	11.11	5.56	5.56	0	0	0	0	0
17.65	0	0	0	0	0	0	11.76	11.76
7.69	0	0	0	0	0	0	0	0
0	0	0	0	0	0	0	0	0
7.69	7.69	7.69	0	0	0	0	15.38	0
7.41	0	0	0	0	0	0	3.7	0
0	0	0	0	0	0	0	4	0
0	0	0	0	0	0	0	0	0
2.56	5.13	2.56	2.56	2.56	0	0	2.56	0
2.7	0	0	0	0	0	0	0	0
5.88	0	0	0	0	0	0	5.88	0
0	0	0	0	0	0	0	10	0
0	0	0	0	0	0	0	0	0
10	10	10	0	0	0	0	0	0
12.33	0	0	0	0	0	0	2.74	1.37
6.9	0	0	0	0	0	0	0	0
4.46	1.91	1.27	0.64	0	0	0	8.28	1.91
11.11	0	0	0	0	0	0	4.17	0
0	0	0	0	0	0	0	0	0
5.56	0	0	0	0	0	0	0	0
0	0	0	0	0	0	0	0	0
5	0	0	0	0	0	0	0	0
7.69	0	0	0	0	0	0	7.69	7.69
0	0	0	0	0	0	0	0	0
0	0	0	0	0	0	0	0	0
5.48	1.37	0	1.37	0	0	0	2.74	0
2.04	0	0	0	0	0	0	4.08	0
20	6.67	0	6.67	0	0	0	0	0
4.88	0	0	0	0	0	0	0	0
0	0	0	0	0	0	0	0	0
6.25	0	0	0	0	0	0	0	0
0	0	0	0	0	0	0	0	0
4.5	0	0	0	0	0	0	3.6	0.9
3.85	0	0	0	0	0	0	0	0
3.77	0	0	0	0	0	0	13.21	1.89
6.06	0	0	0	0	0	0	0	0
9.09	0	0	0	0	0	0	0	0
2.56	0	0	0	0	0	0	5.13	0
0	0	0	0	0	0	0	0	0
0	0	0	0	0	0	0	4	0
11.11	0	0	0	0	0	0	0	0
0	0	0	0	0	0	0	6.25	0
0	0	0	0	0	0	0	0	0
7.58	0	0	0	0	0	0	7.58	3.03
4.35	0	0	0	0	0	0	4.35	0
2.08	0	0	0	0	0	0	12.5	10.42
10	0	0	0	0	0	0	0	0
6.78	3.39	3.39	0	0	0	0	8.47	1.69

0	0	0	0	0	0	0	0	0
3.7	1.23	1.23	0	0	0	0	7.41	3.7
14.81	0	0	0	0	0	0	3.7	0
8.7	0	0	0	0	0	0	4.35	0
4	0	0	0	0	0	0	0	0
0	0	0	0	0	0	0	0	0
5.26	0	0	0	0	0	0	0	0
12.5	0	0	0	0	0	0	0	0
10	0	0	0	0	0	0	0	0
0	0	0	0	0	0	0	0	0
0	0	0	0	0	0	0	0	0
0	0	0	0	0	0	0	0	0
6.25	0	0	0	0	0	0	0	0
6.25	0	0	0	0	0	0	6.25	0
0	0	0	0	0	0	0	5.26	0
10	0	0	0	0	0	0	0	0
0	0	0	0	0	0	0	7.14	0
0	0	0	0	0	0	0	0	0
6.67	0	0	0	0	0	0	6.67	0
2.94	0	0	0	0	0	0	2.94	0
4.65	0	0	0	0	0	0	4.65	0
5.88	0	0	0	0	0	0	5.88	0
3.95	0	0	0	0	0	0	6.58	2.63
2.41	1.2	0	1.2	0	0	0	3.61	0
0	0	0	0	0	0	0	5.88	0
0	0	0	0	0	0	0	0	0
6.06	3.03	0	3.03	0	3.03	0	3.03	0
3.45	0	0	0	0	0	0	0	0
3.61	1.2	0	1.2	0	0	0	6.02	3.61
0	0	0	0	0	0	0	3.13	0
5.74	0	0	0	0	0	0	3.28	1.64
5.88	0	0	0	0	0	0	0	0
14.29	0	0	0	0	0	0	0	0
6.17	1.23	0	1.23	1.23	0	0	8.64	0
0	0	0	0	0	0	0	0	0
4.35	0	0	0	0	0	0	0	0
0	0	0	0	0	0	0	0	0
6.67	0	0	0	0	0	0	0	0
3.85	0	0	0	0	0	0	1.92	0
8.57	0	0	0	0	0	0	14.29	2.86
4.76	0	0	0	0	0	0	4.76	4.76
33.33	0	0	0	0	0	0	0	0
0	0	0	0	0	0	0	0	0
11.11	0	0	0	0	0	0	5.56	0
8	0	0	0	0	0	0	0	0
6.33	1.27	1.27	0	0	0	0	0	0
0	0	0	0	0	0	0	0	0
25	0	0	0	0	0	0	0	0
9.26	0	0	0	0	0	0	0	0
8.33	0	0	0	0	0	0	2.78	0

5.56	0	0	0	0	0	0	0	0
3.03	0	0	0	0	0	0	0	0
0	5	5	0	0	0	0	20	10
6.67	0	0	0	0	0	0	0	0
6.78	0	0	0	0	0	0	8.47	1.69
2.56	0	0	0	0	0	0	5.13	0
0	0	0	0	0	0	0	0	0
8	0	0	0	0	0	0	0	0
14.29	0	0	0	0	0	0	0	0
4.55	0	0	0	0	0	0	0	0
4.17	2.08	0	2.08	0	0	0	6.25	0
5.56	0	0	0	0	0	0	5.56	0
4.01	0.36	0	0.36	0	0	0	3.65	0.73
0	0	0	0	0	0	0	2.56	0
22.22	0	0	0	0	0	0	0	0
4.35	0	0	0	0	0	0	17.39	0
12	0	0	0	0	0	0	0	0
7.5	0	0	0	0	0	0	0	0
0	0	0	0	0	0	0	5.26	0
0	0	0	0	0	0	0	0	0
0	0	0	0	0	0	0	0	0
5.26	0	0	0	0	0	0	1.75	0
0	0	0	0	0	0	0	0	0
4.92	0	0	0	0	0	0	4.92	1.64
0	0	0	0	0	0	0	3.33	0
0.94	0	0	0	0	0	0	5.66	0
5.75	0	0	0	0	0	0	1.15	1.15
9.09	9.09	9.09	0	0	0	0	0	0
0	0	0	0	0	0	0	0	0
5.45	1.82	1.82	0	0	0	0	3.64	0
7.14	3.57	0	3.57	3.57	0	0	0	0
0	0	0	0	0	0	0	0	0
1.6	0.8	0.8	0	0	0	0	5.6	2.4
1.61	0	0	0	0	0	0	3.23	0
0	0	0	0	0	0	0	3.7	0
21.43	0	0	0	0	0	0	0	0
14.81	0	0	0	0	0	0	11.11	0
1.72	4.6	0.57	2.3	0.57	0	1.72	3.45	2.3
2.6	2.6	0.52	2.08	1.04	0.52	0.52	1.56	0
5.69	2.69	0.3	2.1	0.6	0	0.3	2.99	0
6.9	3.45	0	3.45	0	0	3.45	6.9	3.45
0	15.38	0	7.69	0	0	7.69	7.69	7.69
1.56	3.13	1.56	1.56	0	0	1.56	3.13	3.13
6.71	6.71	0.61	0	0	0	0	9.15	7.32
3.51	0.88	0	0.88	0	0	0	1.75	1.75
16.67	0	0	0	0	0	0	0	0
0	2.15	2.15	0	0	0	0	5.38	1.08
3.33	0	0	0	0	0	0	3.33	0
0	0	0	0	0	0	0	6.67	0
1.59	5.56	0	4.76	0	0	3.17	3.97	2.38

3.45	3.45	0	3.45	0	0.86	0.86	2.59	0.86
3.04	3.65	1.52	2.13	0.3	0	1.22	3.95	1.22
1.09	2.17	1.09	1.09	0	0	1.09	3.26	1.09
0	0	0	0	0	0	0	0	0
2.1	6.29	2.1	4.2	0	0.7	0.7	4.9	1.4
0	6.06	1.52	4.55	1.52	0	1.52	6.06	1.52
4.14	3.76	3.01	0.75	0	0	0.75	3.38	0
4.14	0.69	0	0.69	0	0	0.69	5.52	2.76
3.7	1.85	0	1.85	0	0	1.85	9.26	5.56
0	0	0	0	0	0	0	20	0
5.74	4.1	0	4.1	0	1.64	0	4.1	0
2.94	0	0	0	0	0	0	0	0
4.81	2.88	1.92	0.96	0	0.96	0	4.81	0
17.65	5.88	0	5.88	0	0	0	0	0
16.98	0	0	0	0	0	0	7.55	0
0	0	0	0	0	0	0	14.29	0
3.77	1.89	1.89	0	0	0	0	1.89	0
0	0	0	0	0	0	0	0	0
2.78	5.56	0	5.56	0	5.56	0	2.78	0
9.09	2.27	0	2.27	0	2.27	0	6.82	0
2.04	4.08	0	4.08	0	0	0	4.08	2.04
1.47	2.94	0	2.94	2.94	0	0	2.94	1.47
0	0	0	0	0	0	0	21.43	7.14
8.33	0	0	0	0	0	0	2.78	2.78
4.17	4.17	0	4.17	0	2.08	2.08	6.25	4.17
4.4	2.2	1.1	1.1	1.1	0	0	2.2	1.1
1.25	1.25	0	1.25	0	0	0	1.25	1.25
6.61	4.41	0	4.41	2.2	0	0.88	2.64	0.88
2.04	4.08	2.04	2.04	0	2.04	0	8.16	2.04
0	0	0	0	0	0	0	0	0
0	0	0	0	0	0	0	0	0
12.5	12.5	0	12.5	0	0	12.5	0	0
4.55	0	0	0	0	0	0	4.55	4.55
0	0	0	0	0	0	0	25	12.5
0	0	0	0	0	0	0	0	0
4.35	0	0	0	0	0	0	0	0
7.29	0	0	0	0	0	0	4.17	2.08
5.08	3.39	1.69	1.69	0	0	0	3.39	1.69
2.44	4.88	0	4.88	4.88	0	0	4.88	0
2.17	4.35	4.35	0	0	0	0	4.35	0
1.79	5.36	0	5.36	5.36	0	0	5.36	1.79
8.57	0	0	0	0	0	0	8.57	2.86
9.52	0	0	0	0	0	0	0	0
6.56	1.91	0.82	1.09	0.82	0	0.27	4.37	1.37
8.62	1.72	0	1.72	1.72	0	0	1.72	0
4.69	1.56	0	1.56	0.78	0	0	0.78	0
7.69	0	0	0	0	0	0	0	0
3.33	13.33	0	13.33	0	0	0	0	0
0	0	0	0	0	0	0	0	0
3.96	2.52	0.36	2.16	0.36	0	1.08	2.52	0.72

12.5	0	0	0	0	0	0	25	25
15.79	0	0	0	0	0	0	5.26	5.26
0	22.22	0	22.22	0	22.22	0	0	0
10.53	0	0	0	0	0	0	0	0
6	2	0	1.33	0.67	0	0.67	2.67	0.67
9.68	0	0	0	0	0	0	3.23	0
0	0	0	0	0	0	0	0	0
7.37	0	0	0	0	0	0	3.16	0
2.88	3.85	1.92	1.92	0	0.96	0	2.88	0.96
0	12.5	0	12.5	0	0	12.5	12.5	12.5
13.04	0	0	0	0	0	0	4.35	0
4.17	0	0	0	0	0	0	4.17	4.17
9.09	1.52	0	1.52	0	0	0	1.52	0
6.25	2.08	0	2.08	0	0	0	0	0
1.82	1.82	1.82	0	0	0	0	5.45	3.64
6.67	0	0	0	0	0	0	1.67	0
4.27	2.56	0.85	1.71	0	0	1.71	6.84	1.71
0	1.67	0	1.67	1.67	0	0	0	0
2.44	1.22	0.61	0.61	0.61	0	0	2.44	0
4.92	0	0	0	0	0	0	1.64	0
3.85	0	0	0	0	0	0	3.85	0
5.19	1.3	0	1.3	0	0	1.3	2.6	0
3.76	0.75	0	0.75	0	0	0	3.76	0
3.28	0	0	0	0	0	0	6.56	0
5	0	0	0	0	0	0	0	0
4.76	4.76	2.38	2.38	1.19	0	1.19	4.76	1.19
0	0	0	0	0	0	0	12.5	0
0	0	0	0	0	0	0	2.78	0
12.5	0	0	0	0	0	0	6.25	0
1.59	3.17	3.17	0	0	0	0	7.94	1.59
6.25	0	0	0	0	0	0	6.25	0
4	0	0	0	0	0	0	2	0
0	5.88	0	5.88	0	0	5.88	5.88	0
3.28	0	0	0	0	0	0	4.92	1.64
1.49	1.49	1.49	0	0	0	0	2.99	1.49
4.76	0	0	0	0	0	0	1.59	0
0	1.37	1.37	0	0	0	0	5.48	1.37
0	0	0	0	0	0	0	4.17	4.17
0	0	0	0	0	0	0	3.33	0
0	10.53	5.26	5.26	0	0	5.26	5.26	0
5.36	1.79	0	1.79	0	0	0	5.36	1.79
3.64	1.82	1.82	0	0	0	0	5.45	1.82
3.28	4.92	0	4.92	3.28	0	0	1.64	0
10.34	0	0	0	0	0	0	6.9	0
3.08	3.08	3.08	0	0	0	0	4.62	0
0.81	1.63	0	1.63	0	0	0.81	4.88	0
0	0	0	0	0	0	0	0	0
5.32	2.13	1.06	0	0	0	0	6.38	4.26
6.25	0	0	0	0	0	0	12.5	6.25
0	0	0	0	0	0	0	16.67	0

5.81	2.33	1.16	1.16	1.16	0	0	4.65	2.33
5.13	2.56	2.56	0	0	0	0	5.13	2.56
4	12	8	4	0	0	4	8	4
3.19	3.19	3.19	0	0	0	0	6.38	0
4.62	3.08	0	3.08	3.08	0	0	3.08	1.54
9.09	0	0	0	0	0	0	6.82	2.27
0	5.88	5.88	0	0	0	0	5.88	0
6.45	6.45	1.61	4.84	0	0	4.84	8.06	0
6.67	0	0	0	0	0	0	20	0
1.35	2.7	0	1.35	0	0	1.35	2.7	0
6.11	1.53	0.76	0	0	0	0	2.29	0
1.35	1.35	0	1.35	0	0	1.35	8.11	5.41
0	0	0	0	0	0	0	3.7	0
8.82	1.47	1.47	0	0	0	0	5.88	1.47
9.26	0	0	0	0	0	0	3.7	1.85
8	0.67	0.67	0	0	0	0	2.67	0
5.56	0	0	0	0	0	0	8.33	0
4	0	0	0	0	0	0	0	0
3.03	0.76	0.76	0	0	0	0	3.03	0
7.01	3.82	1.27	2.55	0	0.64	1.91	8.28	0.64
7.41	7.41	0	7.41	0	0	7.41	3.7	3.7
7.84	0	0	0	0	0	0	1.96	0
6.82	0	0	0	0	0	0	4.55	0
1.63	1.63	0.54	1.09	0.54	0	0	2.72	1.63
5.88	1.96	1.96	0	0	0	0	3.92	3.92
6.21	1.38	0.69	0.69	0	0	0	3.45	0
10	5	0	5	0	0	5	0	0
3.13	3.13	0	3.13	0	0	3.13	0	0
14.29	0	0	0	0	0	0	1.43	0
7.14	0	0	0	0	0	0	9.52	2.38
3.64	0	0	0	0	0	0	1.82	0
5	0	0	0	0	0	0	5	2.5
8.05	1.15	0	1.15	0	0	1.15	4.02	0.57
0	3.23	0	3.23	0	0	3.23	3.23	0
6.12	0	0	0	0	0	0	4.08	2.04
5.88	0	0	0	0	0	0	2.94	0
7.55	1.89	1.89	0	0	0	0	3.77	0
0	11.11	11.11	0	0	0	0	0	0
6.52	0	0	0	0	0	0	6.52	4.35
10	5	0	5	0	0	0	0	0
1.89	1.89	0	1.89	0	0	1.89	5.66	1.89
21.43	0	0	0	0	0	0	0	0
6.45	0.81	0.81	0	0	0	0	6.45	0
3.39	0	0	0	0	0	0	5.08	0.85
3.31	0	0	0	0	0	0	2.65	0.66
3.45	3.45	0.86	2.59	0.86	0	0	0	0
5.56	0.93	0	0.93	0	0	0.93	7.41	1.85
6.67	0.67	0	0	0	0	0	2	1.33
9.52	1.59	0	1.59	0	1.59	0	3.17	0
0	9.09	0	9.09	0	0	9.09	9.09	0

8.75	2.5	0	1.25	0	0	0	5	1.25
1.9	2.86	0.95	1.43	0.48	0.48	0	2.86	0
7.89	0	0	0	0	0	0	7.89	0
6.25	4.17	2.08	2.08	0	0	0	6.25	4.17
0	13.33	6.67	6.67	0	0	6.67	6.67	6.67
0	0	0	0	0	0	0	16.67	0
3.08	3.08	0	0.77	0	0	0.77	6.15	0
4.76	4.76	0	4.76	0	0	0	4.76	0
3.03	0	0	0	0	0	0	15.15	6.06
0	0	0	0	0	0	0	8.33	2.78
7.27	1.82	1.82	0	0	0	0	3.64	3.64
0	11.11	5.56	5.56	5.56	0	0	11.11	0
0	0	0	0	0	0	0	0	0
6.25	6.25	3.75	2.5	2.5	0	0	6.25	0
8.11	5.41	2.7	2.7	0	0	0	5.41	0
5	2.5	0	2.5	0	0	0	5	0
2.63	2.63	0	2.63	0	0	0	5.26	0
7.89	2.63	0	2.63	0	0.88	1.75	1.75	0
5	0	0	0	0	0	0	5	0
6.25	12.5	0	6.25	0	0	0	12.5	6.25
5.13	5.13	2.56	2.56	0	0	2.56	5.13	2.56
11.54	7.69	0	3.85	0	0	0	7.69	3.85
1.52	7.58	0	6.06	1.52	0	0	6.06	1.52
0	6.82	4.55	2.27	0	0	0	2.27	2.27
0	16.67	0	16.67	0	0	0	0	0
0	0	0	0	0	0	0	20	0
4.88	0	0	0	0	0	0	2.44	0
5.88	0	0	0	0	0	0	5.88	0
10	0	0	0	0	0	0	10	0
6.41	3.85	0	3.85	1.28	0	0	0	0
7.86	2.14	0.71	1.43	0.71	0	0.71	2.14	0
3.08	1.54	0	1.54	0	0	0	3.08	0
5	2.5	0	2.5	0	2.5	0	3.75	2.5
1.47	2.94	0	2.94	1.47	0	1.47	0	0
5.88	0	0	0	0	0	0	2.94	0
1.96	1.96	0	1.96	0	0	0	9.8	1.96
5.56	2.78	2.78	0	0	0	0	0	0
3.01	1.2	0.6	0	0	0	0	4.22	1.2
6.78	2.54	0	2.54	0	0	0	5.08	0.85
20	0	0	0	0	0	0	10	0
20	0	0	0	0	0	0	0	0
0	0	0	0	0	0	0	0	0
6.34	3.52	0	3.52	0	0	0.7	0.7	0
4.92	1.64	0.82	0.82	0.82	0	0	1.64	0
10.91	0	0	0	0	0	0	5.45	0
6.01	1.64	0	1.64	0.55	0	0	2.73	0
4.17	0	0	0	0	0	0	8.33	0
7.44	8.26	1.65	5.79	1.65	1.65	1.65	0.83	0
0	0	0	0	0	0	0	8.7	0
0	0	0	0	0	0	0	0	0

7.69	0	0	0	0	0	0	1.54	1.54
2.89	4.05	2.89	0.58	0	0	0.58	4.62	0.58
3.76	0.75	0	0.75	0	0	0	1.5	0
2.11	2.11	1.05	1.05	1.05	0	0	9.47	4.21
3.65	2.43	1.22	0.97	0	0.24	0	4.62	0.24
0	0	0	0	0	0	0	30	10
3.94	2.46	0	1.48	0.49	0.99	0	2.96	0.49
3.55	0.51	0	0.51	0.51	0	0	3.05	1.02
0	0	0	0	0	0	0	8.33	0
4	0	0	0	0	0	0	0	0
5.26	5.26	5.26	0	0	0	0	10.53	5.26
4.55	0	0	0	0	0	0	4.55	4.55
7.69	0	0	0	0	0	0	15.38	0
0	0	0	0	0	0	0	0	0
5.17	0	0	0	0	0	0	1.72	1.72
10	0	0	0	0	0	0	10	0
0	0	0	0	0	0	0	0	0
0	0	0	0	0	0	0	0	0
4.92	1.64	1.64	0	0	0	0	1.64	1.64
4.74	0.95	0	0.95	0	0.47	0	1.9	0.47
6.82	0	0	0	0	0	0	2.27	2.27
6	0	0	0	0	0	0	6	0
6.06	0	0	0	0	0	0	0	0
1.15	2.3	1.15	1.15	0	0	0	6.9	0
11.11	4.44	2.22	2.22	0	0	0	2.22	2.22
4.83	0.69	0.69	0	0	0	0	4.83	1.38
4.44	0	0	0	0	0	0	2.22	2.22
10	0	0	0	0	0	0	6.67	3.33
10.26	2.56	0	0	0	0	0	17.95	12.82
14.81	7.41	3.7	3.7	0	0	0	3.7	3.7
0	10	10	0	0	0	0	10	0
0	0	0	0	0	0	0	3.85	0
4.55	0	0	0	0	0	0	2.27	0
10	0	0	0	0	0	0	0	0
3.05	3.82	1.53	2.29	0	0	0	5.34	2.29
2.04	4.08	2.04	2.04	0	0	0	6.12	0
6.17	2.47	1.23	1.23	0	0	0	4.94	1.23
3.13	3.13	3.13	0	0	0	0	6.25	3.13
1.85	0	0	0	0	0	0	7.41	3.7
0	2.22	2.22	0	0	0	0	8.89	2.22
10	6.67	3.33	3.33	0	0	0	10	3.33
7.69	0	0	0	0	0	0	15.38	0
5.93	2.96	2.22	0.74	0	0	0	10.37	0.74
0	0	0	0	0	0	0	0	0
8.57	5.71	2.86	2.86	2.86	0	0	5.71	0
0	0	0	0	0	0	0	0	0
3.33	6.67	0	6.67	0	0	1.67	3.33	0
0	3.57	0	0	0	0	0	3.57	0
6.67	6.67	3.81	2.86	0	0	1.9	4.76	0
2.86	0	0	0	0	0	0	0	0

5.13	5.13	2.56	2.56	0	0	0	10.26	2.56
4.26	2.13	2.13	0	0	0	0	6.38	1.06
4.76	2.38	2.38	0	0	0	0	2.38	0
1.49	4.48	2.99	1.49	0	0	0	4.48	1.49
7.69	5.13	2.56	2.56	0	0	0	7.69	2.56
2.5	7.5	2.5	5	5	0	0	2.5	0
9.86	4.23	0	4.23	1.41	0	1.41	0	0
7.14	7.14	0	7.14	0	0	0	3.57	0
3.23	3.23	3.23	0	0	0	0	9.68	6.45
6.51	3.72	1.86	1.4	0.47	0	0.47	8.84	1.4
10.34	2.3	2.3	0	0	0	0	10.34	2.3
9.09	0	0	0	0	0	0	0	0
6.13	3.07	1.84	1.23	0	0	0	6.13	0.61
10	0	0	0	0	0	0	0	0
3.15	1.57	1.57	0	0	0	0	5.51	0.79
0	2.86	0	2.86	0	0	0	5.71	2.86
0	0	0	0	0	0	0	7.14	0
0	7.69	0	7.69	0	0	0	23.08	15.38
0	0	0	0	0	0	0	35.71	7.14
0	12.5	4.17	8.33	0	0	8.33	16.67	8.33
9.09	9.09	6.06	3.03	0	0	0	3.03	0
4.35	0	0	0	0	0	0	4.35	0
20	0	0	0	0	0	0	0	0
0	3.57	3.57	0	0	0	0	17.86	7.14
2.7	2.7	2.7	0	0	0	0	16.22	2.7
6.06	0	0	0	0	0	0	6.06	0
0	11.11	11.11	0	0	0	0	11.11	0
6.77	3.76	2.26	0.75	0	0	0	4.51	1.5
4.98	3.2	1.42	1.78	0	0	0.71	4.63	0.71
1.25	3.75	0	3.75	0	0	1.25	5	0
4.17	4.17	2.08	2.08	0	0	2.08	6.25	0
7.35	2.21	0.74	0.74	0	0	0.74	6.62	0.74
5.56	0	0	0	0	0	0	11.11	0
3.7	7.41	7.41	0	0	0	0	7.41	0
3.23	9.68	0	9.68	0	0	0	6.45	6.45
6.97	2.11	0.49	1.46	0.16	0.16	0.81	6.16	0.65
4.84	3.23	0.81	2.42	0.81	0	1.61	4.84	0
8.84	1.1	0	1.1	0.55	0	0	5.52	0.55
8.38	1.2	1.2	0	0	0	0	7.19	0.6
4.08	0	0	0	0	0	0	4.08	4.08
4.71	2.35	1.18	1.18	0	0	0	7.06	3.53
6.06	3.03	0	3.03	0	0	3.03	6.06	0
6.25	1.25	0	1.25	0	0	1.25	5	0
3.45	5.17	5.17	0	0	0	0	5.17	0
3.64	3.64	0	1.82	0	0	1.82	5.45	5.45
3.85	3.85	3.85	0	0	0	0	8.97	3.85
3.99	1	0	1	0.33	0.33	0	7.31	2.66
0	0	0	0	0	0	0	0	0
10.71	3.57	0	3.57	0	0	0	3.57	3.57
0	0	0	0	0	0	0	7.69	0

10.53	2.63	0	2.63	0	0	0	5.26	2.63
5.05	2.02	0	2.02	0	0	1.01	3.03	2.02
10.81	0	0	0	0	0	0	6.76	1.35
10	0	0	0	0	0	0	0	0
12.5	12.5	0	12.5	0	0	12.5	0	0
2.67	6.67	1.33	5.33	0	0	4	2.67	1.33
2.08	4.17	2.08	2.08	0	0	0	2.08	0
3.45	0	0	0	0	0	0	3.45	0
3.17	0	0	0	0	0	0	3.17	1.59
4.95	0	0	0	0	0	0	5.94	0.99
8.49	0	0	0	0	0	0	4.72	2.83
6	0	0	0	0	0	0	6	4
8.7	8.7	0	8.7	0	0	8.7	4.35	0
5.04	0.72	0.72	0	0	0	0	6.47	0.72
5.56	2.78	0	2.78	0	0	0	2.78	2.78
7.24	0.66	0	0.66	0	0	0	2.63	0.66
6.67	0	0	0	0	0	0	2.22	2.22
0	0	0	0	0	0	0	2.5	2.5
1.75	1.75	0.88	0.88	0	0	0.88	0.88	0.88
15.63	0	0	0	0	0	0	3.13	0
0	5	1.25	3.75	0	2.5	0	7.5	0
3	0	0	0	0	0	0	3	2
3.57	1.79	0	1.79	0	0	0	5.36	0
4.05	1.35	0.68	0.68	0	0	0	5.41	2.7
5.05	1.01	0	0	0	0	0	4.04	2.02
3.31	1.65	0.83	0.83	0	0	0	8.26	1.65
0	0	0	0	0	0	0	9.09	9.09
3.11	3.63	1.04	2.59	1.55	0.52	0.52	5.7	1.55
5.56	0	0	0	0	0	0	5.56	0
0	0	0	0	0	0	0	4.76	4.76
0	0	0	0	0	0	0	0	0
3.36	1.01	0.34	0.67	0.34	0	0.34	4.03	1.68
3.03	0	0	0	0	0	0	0	0
4.17	0	0	0	0	0	0	6.25	4.17
18.18	0	0	0	0	0	0	9.09	9.09
8.51	2.13	2.13	0	0	0	0	4.26	2.13
0	0	0	0	0	0	0	0	0
0	8.33	0	8.33	0	0	8.33	8.33	0
0	0	0	0	0	0	0	16.67	0
3.85	0	0	0	0	0	0	7.69	3.85
5.88	0	0	0	0	0	0	5.88	0
10	0	0	0	0	0	0	0	0
0	4.55	0	4.55	0	4.55	0	13.64	4.55
0	0	0	0	0	0	0	0	0
8.33	0	0	0	0	0	0	8.33	8.33
0	50	0	25	0	0	0	0	0
4	4	0	4	0	2	0	12	4
5.41	0	0	0	0	0	0	2.7	0
2.7	0	0	0	0	0	0	0	0
0	0	0	0	0	0	0	12.5	12.5

5.19	0.74	0.74	0	0	0	0	4.44	0
6.38	0	0	0	0	0	0	8.51	4.26
0	5.56	0	5.56	2.78	2.78	0	5.56	2.78
3.9	1.38	0.23	1.15	0.46	0.69	0.23	4.36	1.15
5	1.25	1.25	0	0	0	0	3.75	0
0	14.29	0	14.29	0	0	0	0	0
0	0	0	0	0	0	0	0	0
2.44	2.44	0	2.44	0	0	2.44	7.32	2.44
5.13	0	0	0	0	0	0	7.69	2.56
0	0	0	0	0	0	0	3.03	0
0	0	0	0	0	0	0	50	0
5.09	1.67	0.88	0.78	0.2	0	0	4.11	0.69
2.15	1.08	1.08	0	0	0	0	2.15	0
3.97	1.59	0.79	0.79	0	0.79	0	1.59	0
4.11	1.37	0	1.37	0	0	0	5.48	1.37
9.43	0	0	0	0	0	0	0	0
4.07	0	0	0	0	0	0	2.44	0
0	0	0	0	0	0	0	0	0
0	0	0	0	0	0	0	16.67	0
4.72	2.36	0	2.36	0.47	0.47	0.47	2.83	0.47
0	16.67	0	16.67	0	0	16.67	0	0
6.53	2.04	0.41	1.63	0.82	0.41	0	1.22	0
0	0	0	0	0	0	0	0	0
6	3	2	1	1	0	0	2	1
8.82	0	0	0	0	0	0	2.94	2.94
3.25	0.23	0	0.23	0	0.23	0	4.41	0.93
8.2	0	0	0	0	0	0	3.28	3.28
1.11	2.22	2.22	0	0	0	0	10	2.22
4.35	8.7	8.7	0	0	0	0	13.04	4.35
6.63	1.02	0.51	0.51	0.51	0	0	3.06	0.51
5.26	0	0	0	0	0	0	0	0
0	4.55	4.55	0	0	0	0	0	0
0	2.44	0	2.44	0	0	0	2.44	0
5.3	0.66	0	0.66	0	0	0	3.31	0
0	0	0	0	0	0	0	16.67	0
1.74	1.74	1.3	0.43	0.43	0	0	0.43	0
4.73	1.13	0	0.68	0.23	0	0	2.7	0.68
3.64	1.82	1.82	0	0	0	0	0	0
0	0	0	0	0	0	0	7.69	0
4.32	3.6	0.72	2.16	1.44	0	0.72	4.32	0.72
4	0	0	0	0	0	0	4	0
0	0	0	0	0	0	0	10	2.5
5.34	2.14	0.36	1.78	0.36	0.36	0.71	3.91	1.78
2.63	0	0	0	0	0	0	7.89	0
2.68	1.79	0	1.79	0	0	0	3.57	0
3.23	0	0	0	0	0	0	3.23	1.61
7.62	5.71	2.86	1.9	0	0	0	1.9	0.95
5.44	2.51	0.42	1.67	0.42	0.42	0	4.18	0.42
1.96	0.98	0.98	0	0	0	0	5.88	3.92
0.93	2.8	2.8	0	0	0	0	7.48	2.8

11.21	0.93	0.93	0	0	0	0	7.48	2.8
5.26	0	0	0	0	0	0	2.63	0
0	0	0	0	0	0	0	9.52	0
7.02	0	0	0	0	0	0	5.85	3.51
5.83	4.85	1.94	2.91	2.91	0	0	5.83	0.97
0	12.5	12.5	0	0	0	0	12.5	0
3.92	0	0	0	0	0	0	1.96	0
2.04	6.12	6.12	0	0	0	0	8.16	4.08
0	11.11	11.11	0	0	0	0	11.11	11.11
2.31	2.54	0	2.31	0.46	0	1.39	3.7	0.69
3.81	3.81	1.9	1.9	0	0	0.95	1.9	0.95
3.41	3.98	1.7	1.14	0	0	0.57	3.41	1.7
1.54	0	0	0	0	0	0	6.15	4.62
5.45	2.73	0.91	0.91	0	0	0.91	3.64	3.64
3.42	2.99	1.71	1.28	0	0	0.85	4.7	2.14
4.27	7.69	2.56	5.13	0.85	0	4.27	5.13	1.71
4.39	2.44	0.98	0.98	0.49	0	0.49	2.93	1.95
2.88	4.81	1.92	1.92	0.96	0	0	5.77	0
6.09	1.74	0	1.74	0.87	0	0.87	4.35	1.74
2.38	4.76	0	2.38	0	0	0	4.76	3.57
0	6.06	0	6.06	0	0	0	6.06	3.03
3.53	3.53	1.18	1.76	0	0	0.59	4.71	2.35
1.61	9.68	3.23	6.45	1.61	0	3.23	4.84	0
0	3.39	1.69	0	0	0	0	6.78	3.39
6.1	4.88	1.22	2.44	1.22	0	1.22	3.66	2.44
5.88	1.47	0	1.47	0	0	0	8.82	2.94
1.92	2.68	1.15	1.15	0	0	0.77	5.36	2.3
7.03	3.91	0.78	1.56	0	0	0.78	2.34	1.56
0	6.9	3.45	3.45	3.45	0	3.45	0	0
1.32	10.6	1.99	7.95	0.66	2.65	3.31	5.3	0
9.09	6.82	4.55	2.27	0	0	2.27	9.09	4.55
0	7.5	2.5	0	0	0	0	5	2.5
5.22	4.35	2.61	0.87	0	0	0	1.74	0.87
7.51	2.89	0.58	1.73	0.58	0	0.58	1.73	0
6.67	3.33	1.67	1.67	0	0	0	5	1.67
3.6	1.8	0.9	0.9	0	0	0	9.01	3.6
4.65	2.33	1.16	0	0	0	0	4.07	2.91
4.61	2.85	0.33	2.08	0.22	0	0.77	3.62	1.75
3.03	5.05	2.02	3.03	0	0	0	4.04	3.03
4.26	3.55	1.42	0.71	0	0	0	4.26	1.42
1.39	2.78	0	2.78	0	0	0	8.33	4.17
10	5	5	0	0	0	0	5	0
0	1.64	1.64	0	0	0	0	3.28	1.64
4	1.33	1.33	0	0	0	0	8	5.33
1.67	5	1.67	2.5	1.67	0	0.83	2.5	1.67
0	9.68	6.45	3.23	0	0	3.23	12.9	0
9.76	0	0	0	0	0	0	4.88	0
2.15	2.15	2.15	0	0	0	0	4.3	2.15
4.72	0.94	0	0.94	0.94	0	0	6.6	1.89
1.61	4.84	3.23	1.61	0	0	0	1.61	1.61

1.75	5.26	0	1.75	0	0	1.75	5.26	1.75
6.49	3.9	0	1.3	0	0	1.3	9.09	6.49
1.96	2.94	1.96	0.98	0	0	0.98	4.9	1.96
3.45	1.48	0	1.48	0	0	0.49	2.96	2.46
0	3.03	3.03	0	0	0	0	6.06	3.03
0	4.17	0	0	0	0	0	12.5	12.5

politeness	conflict	moralisatic	communica	focuspast	focusprese	focusfuture
1.49	2.99	1.49	7.46	5.97	1.49	2.99
0	0	0	0	0	5.26	5.26
0	0	0	2.94	2.94	8.82	2.94
0	0	1.33	1.33	6.67	6.67	0
0	5.41	1.35	8.11	0	9.46	1.35
0	0	0	0	10.53	2.63	2.63
0	1.32	1.32	1.32	14.47	0	0
0	0	0	4.88	12.2	2.44	2.44
0	0	1.82	7.27	14.55	3.64	1.82
0	1.92	0.96	1.92	8.65	9.62	4.81
0	0	0	6.25	12.5	3.13	3.13
5.26	0	0	0	10.53	0	0
0	0	0.87	1.74	11.3	0	0
0	0	0	0	0	0	0
0	0	0	0	0	12.5	12.5
0	0	0	0	11.11	14.81	3.7
0	0	8.33	0	0	16.67	0
0	0	6.25	0	12.5	6.25	0
0	0	0	0	0	25	8.33
0	1.83	0	2.75	12.84	0.92	2.75
0	0	0	0	0	18.75	0
0	0	0	0	12.5	0	0
0	0	7.14	0	14.29	7.14	0
1.64	1.64	3.28	3.28	14.75	0	0
0	1.96	0	5.88	7.84	3.92	0
0	0	5.88	0	2.94	11.76	0
0	8	0	4	8	0	0
0	2.44	0	7.32	8.54	1.22	2.44
0	2.33	0	4.65	9.3	4.65	2.33
2.94	2.94	0	2.94	5.88	0	2.94
0	4.11	0	5.48	6.85	0	0
0	4.07	0	10.57	11.38	0	0.81
0	5	0	0	10	0	0
0	1.96	1.96	7.84	11.76	1.96	1.96
0	0	0.88	1.75	12.28	0.88	4.39
0	1.08	0	0	11.83	1.08	4.3
0	0	0	0	14.29	0	0
0	2.78	0	5.56	11.11	0	0
0	4.55	4.55	4.55	9.09	9.09	0
0	1.34	0	4.03	8.72	3.36	1.34
0	1.08	1.08	1.08	4.86	7.03	4.86
0	1.82	0	5.45	14.55	1.82	5.45
0	0	0.64	1.6	2.56	7.37	3.21
0	0	2.7	2.7	4.05	5.41	0
0	0	0	0	7.69	1.54	3.08
1.19	0	0	1.19	1.19	11.9	1.19
0	0	2	0	6	8	2
0	0	0	0	3.85	9.62	0
0	1.47	1.47	7.35	0	2.94	2.94

0	0	2.2	0	4.4	5.49	2.2
0	1.49	0	2.99	10.45	5.97	0
0.73	0	2.19	0.73	2.92	2.19	2.19
0	0	3.33	0	0	16.67	3.33
0	0	2.94	1.47	7.35	2.94	0
4.4	0	0	2.2	1.1	10.99	3.3
0	1.35	0	1.35	1.35	2.7	6.76
2.5	0	1.25	1.25	8.75	1.25	0
0	0	0.85	0	5.93	3.39	2.54
0	0	0	2.6	3.9	6.49	1.3
6.06	0	0	0	3.03	12.12	0
1.69	0	1.69	1.69	6.78	8.47	1.69
0	0	0	2.86	5.71	5.71	4.29
0	0	0	0	16.67	0	4.17
0	0	0	2.27	2.27	6.82	2.27
0	0	2.7	1.35	2.7	8.11	0
0	0	3.13	0	12.5	3.13	1.56
0	0	1.14	1.52	4.17	6.82	0.76
3.45	0	1.72	3.45	1.72	13.79	3.45
0	0	3.7	3.7	7.41	3.7	3.7
0	0	0	2.56	5.13	10.26	0
0	0	2.33	4.65	6.98	2.33	2.33
0	0	0.94	2.83	0	4.72	0.94
0	1.61	0	3.23	4.84	1.61	6.45
0	1.81	0	2.41	4.82	8.43	3.61
0	0.87	0	2.61	0.87	6.09	5.22
0	0	2.38	0	11.9	4.76	0
0	0	1.2	2.41	1.81	7.83	0.6
0	0	1.31	2.61	2.61	8.5	1.96
0	0	0	0.75	2.99	7.46	2.99
0	0	1.45	1.45	14.49	5.8	5.8
0	0	0	0	5.17	4.31	0
3.7	0	0	3.7	5.56	7.41	1.85
0	0	0	0	0	10.53	0
0	0	0.96	1.92	3.85	3.85	0.96
0	1.61	0.81	1.61	6.45	8.06	1.61
0	0	0	1.04	4.17	8.33	0
0	0	0	0	4	4	0
0	0	2.36	2.36	4.72	3.94	1.57
0.69	0.69	1.39	1.39	1.39	6.94	4.86
0	0	2.44	3.66	7.32	2.44	1.22
0	1.22	2.44	2.44	1.22	8.54	2.44
0	0	0	7.14	3.57	7.14	0
0	0	0	2.9	2.9	7.25	1.45
0	1.5	0.75	3.01	6.02	3.76	3.76
0	0.87	0.87	2.61	7.83	2.61	0.87
1.64	1.64	0	1.64	11.48	4.92	0
0	0	0	0	9.09	0	0
0	0	0	0	0	0	0
0	0	0	6.25	0	3.13	0

0	0	0	2.33	2.33	0	0
0	3.13	0	3.13	12.5	0	3.13
0	0	0	0	0.98	9.8	0
0	4.43	0.99	6.4	3.94	6.9	0
0	2.08	2.08	2.08	10.42	4.17	0
0	0	0	5.41	6.76	0	1.35
0	0	1.37	6.85	6.85	2.74	0
0	1.64	1.64	3.28	9.84	1.64	0
0	0.47	0.47	2.79	1.4	5.12	2.79
0	1.28	0	3.85	0	7.69	0
0.22	1.1	0.22	4.18	8.35	3.08	1.1
0	4.35	0	0	0	17.39	0
0	0	0	0	5.56	5.56	0
0	0	0	2.78	0	11.11	0
0	2.56	2.56	0	0	10.26	0
0	0	0	0	0	0	0
0	3.23	3.23	3.23	0	9.68	0
0	0	0	0	2.17	10.87	0
0	0	0	0	6.25	9.38	0
0	3.7	0	0	0	14.81	0
0	5.56	0	3.33	11.11	1.11	0
0	1.56	0	3.13	0	4.69	6.25
3.45	0	0	3.45	0	6.9	0
0	0	0	4	8	0	4
0	4.55	2.27	2.27	0	13.64	0
0	3.57	0	7.14	7.14	7.14	0
0	0	0	0	3.51	5.26	1.75
0	5.26	0	0	0	21.05	0
0	1.83	0	3.67	0.92	5.5	0
0	0	2.94	2.94	8.82	8.82	0
0	0	0	0	0	8.33	0
0	0	0	5.08	5.08	3.39	3.39
0	0	0	5.77	1.92	5.77	1.92
0	0.99	0.99	3.96	1.98	5.94	2.97
0	2.61	0.87	2.61	11.3	0	4.35
0	0	0	0	11.11	0	0
0	0	0	6.67	15.56	2.22	0
0	0	0	6.67	15.56	2.22	0
3.7	0	0	1.85	11.11	3.7	0
0	0	0	5	2.5	5	10
0	2.06	0	1.03	1.03	4.12	0
2.08	0	0	10.42	0	2.08	2.08
0	0	0	2.27	4.55	6.82	0
0	0	2.13	2.13	2.13	2.13	4.26
0	0	0	0	2.04	8.16	2.04
0	4.76	0	4.76	0	4.76	0
3.13	0	0	6.25	3.13	9.38	3.13
0	5.26	0	0	5.26	10.53	0
0	1.19	0	5.95	7.14	2.38	1.19
0	5.41	0	0	0	8.11	5.41

0	0	0	3.03	0	3.03	3.03
0	0	0	0	5.26	13.16	10.53
0	1.79	0	1.79	8.93	1.79	0
0	1.32	0.66	1.99	5.3	5.3	0.66
0	0	0	0	0	6.67	0
0.83	1.67	1.67	2.5	2.5	5.83	1.67
0	0	1.11	1.11	3.33	8.89	0
0	0	0	0	0	5.56	0
1.82	0	1.82	0	1.82	7.27	3.64
0	0	0	0	0	0	0
0	0	0	5.88	2.94	0	8.82
0	0	0	0	0	0	6.25
0	0	2.08	8.33	4.17	6.25	0
0	0	0	0	0	20	6.67
0	0	0	0	0	0	0
0	0	0	0	10.53	0	0
0	0	0	2.86	2.86	8.57	0
0	0	0	0	0	4	4
0	0	3.7	0	0	0	3.7
0	0.97	0	2.42	2.9	4.83	1.45
0	0	0	0	0	0	25
0	6.67	0	6.67	0	0	0
0	0	7.14	0	0	0	0
0	7.41	0	0	0	7.41	7.41
0	3.39	0	0	1.69	6.78	1.69
0	3.33	0	3.33	10	0	3.33
0	0	0	0	0	0	0
1.45	0	0	1.45	8.7	7.25	0
1.82	0	0	1.82	5.45	9.09	5.45
0.65	2.6	0	3.25	3.9	5.19	2.6
0	5.26	5.26	2.63	2.63	0	0
0	0	12.5	0	0	0	0
0	0	0	0	0	17.65	17.65
0	4.11	2.74	0	1.37	10.96	0
0	5	5	0	0	5	0
0	0	0	0	11.11	11.11	22.22
0	0	0	0	6.25	6.25	6.25
0	0	0	7.5	0	7.5	5
0	0	11.11	11.11	0	11.11	0
2.27	0	0	2.27	0	2.27	2.27
0	0	0	2.6	10.39	1.3	0
0	1.96	0	0	0	5.88	5.88
0	0	3.7	1.85	5.56	7.41	1.85
0	2.94	2.94	2.94	0	2.94	2.94
0	0	0	0	3.7	3.7	0
0	0	0	0	0	8	0
0	0	0	2.7	4.05	4.05	0
0	0	0	0	0	18.75	0
0	0	0	0	0	11.11	11.11
0	0	0	3.33	3.33	6.67	6.67

0	0	0	2.82	0	11.27	1.41
0	0	0	0	11.11	11.11	0
0	0	0	0	7.69	0	0
2	2	0	4	4	8	2
0	1.18	0	1.18	2.35	7.06	0
0	4.17	0	0	0	12.5	4.17
0	0.94	0.94	0	3.77	5.66	2.83
0	0	3.13	4.69	3.13	9.38	0
0	0	0	4.17	9.72	2.78	2.78
0	0	0	4.17	9.72	2.78	2.78
0	0	0	0	3.7	7.41	0
0	0	0	3.49	3.49	5.81	0
0.41	0.81	0.81	3.25	2.85	7.32	2.03
0	1.92	0	3.85	0	1.92	5.77
0	0	0	6.56	1.64	14.75	3.28
0.4	1.98	0.4	2.78	2.38	5.56	1.98
0	0	10	0	0	0	10
0	0	0	13.33	6.67	13.33	0
0	2.13	0	2.13	4.26	6.38	0
0	0	0	8.82	0	0	2.94
0	0	0	4.76	6.35	1.59	0
0	0	0	0	0	25	16.67
0	0	0	3.85	11.54	7.69	0
0.48	1.45	0	2.42	2.9	9.18	4.35
0	4.69	0	4.69	6.25	3.13	3.13
0	1.54	1.54	0	1.54	4.62	1.54
0	0	0	3.23	3.23	12.9	3.23
0	2.04	0	2.04	6.12	10.2	0
0.66	2.65	0.66	3.31	1.32	7.95	1.99
2.17	2.17	2.17	0	0	6.52	4.35
0	0	0	0	0	12.9	3.23
0	1.56	0.78	3.13	2.34	1.56	3.91
0.38	1.5	0.38	1.88	6.02	3.76	0.38
0	0	0	2.63	4.39	4.39	1.75
0.6	0	0.6	1.81	1.81	1.81	1.2
0	0	4.17	2.78	2.78	8.33	1.39
0	0	0	0	6.67	0	6.67
3.33	0	0	3.33	6.67	6.67	6.67
0	0	0	4.44	2.22	4.44	6.67
0	1.69	0	1.69	0	3.39	0
0	0	0	4	0	0	0
3.03	0	0	3.03	0	6.06	3.03
0	0	0	2.38	4.76	2.38	0
0	1.41	1.41	1.41	1.41	4.23	0
0	0	0	3.08	10.77	4.62	0
0	2.86	2.86	5.71	14.29	0	0
0	3.23	1.61	0	6.45	0	0
0	3.03	0	3.03	0	3.03	0
2.63	2.63	0	5.26	0	10.53	0
0	3.45	0	6.9	20.69	0	0

0	0	0	5.19	6.49	2.6	6.49
0	0	2.17	0	13.04	0	0
0	2.9	0	2.9	5.8	5.8	0
0	0	0	13.33	13.33	0	0
0	0	0	0	0	7.69	0
0	0.46	0.46	5.53	11.06	1.38	0.92
0	0.66	0	3.31	9.93	3.97	0.66
0	0.81	0.4	1.21	2.02	8.06	1.61
0	4	0	0	12	0	0
0	0	0	0	7.14	9.52	2.38
0	2	0.67	4	1.33	6.67	0
0	2.16	0.72	2.88	5.76	3.6	2.88
0	0	0	0	4.88	7.32	2.44
0	2.47	2.47	3.7	8.64	2.47	0
0	0	0.97	3.88	10.68	1.94	2.91
0	3.36	0	7.56	7.56	8.4	0
1	0	0	4	5	10	0
1.06	2.13	1.06	4.26	4.26	11.7	2.13
0	0	12.5	12.5	0	0	0
0	0	4.35	0	21.74	0	0
0	0.94	0.94	2.83	4.72	7.55	0.94
0	3.13	0	3.13	0	3.13	3.13
0	0	0	0	7.69	0	0
0	3.57	7.14	3.57	7.14	7.14	10.71
0	0	0	0	5.56	0	5.56
2.33	2.33	0	2.33	2.33	4.65	4.65
0	2.94	0	5.88	20.59	0	0
0	0	0	13.64	0	4.55	0
0	0.94	1.89	5.66	4.72	7.55	0.94
0	0	0	0	3.57	3.57	0
0	0	0	0	0	5.56	0
0	0.56	0	6.18	10.67	3.37	0.56
0	0	1.67	6.67	10	8.33	1.67
0	0	4.17	0	0	8.33	8.33
0	2.68	1.34	6.04	2.68	8.72	1.34
0	2.27	1.99	1.7	3.13	7.67	1.14
0	0	0	4.62	0	6.15	1.54
0	2.21	0	2.21	3.68	3.68	2.94
0	0	3.85	0	11.54	3.85	0
0	0	2.38	4.76	3.57	5.95	1.19
0	1.35	0	2.7	1.35	6.76	0
0	3.45	3.45	0	1.72	8.62	6.9
2.78	0	1.39	2.78	0	4.17	4.17
0	0	3.03	3.03	3.03	6.06	0
0	0	0.58	0	3.51	1.75	2.34
0	0.69	0	0.69	3.45	6.21	0.69
0	0	0	0	4.76	4.76	0
0	1.8	0.9	3.6	9.01	3.6	1.8
0	0	0	0	12.5	8.33	8.33
0	0	2.56	2.56	3.85	5.13	0

0	0	11.11	0	5.56	16.67	0
0	0	0	0	9.09	3.03	0
0	0	0	2.44	9.76	7.32	2.44
0	0	0	1.45	0	2.9	7.25
0	0	0	0	0	0	0
0	0	2.3	2.3	13.79	2.3	0
0	0	1.32	0	5.26	0	2.63
0	0	0	0	0	0	7.14
0	1.54	0	1.54	3.08	4.62	1.54
0	0	5.41	0	8.11	5.41	0
0	3.23	0	0	6.45	3.23	3.23
0	0	0	1.96	5.88	0.98	1.96
0	2.2	0	3.3	3.3	6.59	0
0	0	0	1.47	14.71	1.47	1.47
0	0.85	2.54	0.85	7.63	1.69	0.85
2.5	0	2.5	5	7.5	5	0
0	0	0	0	4.44	6.67	0
0	0	0	0	37.5	0	0
0	0	0	0	4.55	9.09	0
0	0	0	4.35	4.35	2.9	0
0	1.32	0	1.32	1.32	7.89	2.63
0	1.12	0.56	4.49	2.25	6.74	2.25
0	1.64	1.64	1.64	3.28	8.2	0
0	0	0	0	5.66	3.77	0
0	3.45	1.72	0	0	6.9	0
0	0	0	0	16.67	0	0
0	0	0	8	4	4	0
1.04	0	0	1.04	5.21	5.21	0
0	0	0	11.11	3.7	0	3.7
0.96	0	0.96	5.77	5.77	2.88	0
0	0	0	0	3.13	3.13	6.25
0	0	8.33	0	16.67	0	0
0	0	1.72	0	5.17	4.31	0.86
0	2.08	0	4.17	2.08	8.33	6.25
0.97	0.97	0.97	2.91	2.91	4.85	3.88
0	0	0	0	12.5	18.75	0
0	1.75	0	1.75	3.51	1.75	7.02
0	0	0	0	0	0	0
1.92	0.96	0	9.62	4.81	4.81	1.92
0	0	0	0	12.5	0	12.5
0	0	0	5	2.5	0	0
0.29	0	0	1.46	4.09	7.89	4.09
0	0	1.89	1.89	5.66	3.77	0
0	0	0	0	0	0	0
0	2.9	1.45	4.35	1.45	4.35	1.45
0	0	0	1.49	2.99	5.97	2.99
0	0	0	0	6.25	6.25	0
0	0	0	0	5.88	5.88	5.88
0	1.79	0	10.71	5.36	3.57	0
0	2.33	2.33	0	2.33	6.98	4.65

0	0	0	0	15.38	0	0
0	0	0	0	9.09	9.09	0
0	0	0	1.41	2.82	2.82	1.41
0	0	0	0	7.41	9.26	0
0	0	1.11	4.44	3.33	8.89	3.33
0	7.69	0	0	0	7.69	0
0	0	0	0	0	6.67	0
1.27	3.8	0	3.8	1.27	8.86	0
0	0	0	0	7.69	7.69	0
0	0	0	2.2	1.1	5.49	3.3
0	0	0	6.12	10.2	2.04	0
0	0	0	0	0	12.5	0
0	0	0	0	3.85	0	15.38
0	0	0	0	8.7	8.7	0
0	0	0	0	10	0	0
0	0	0	9.09	4.55	9.09	4.55
0.37	0.74	0.37	3.31	5.88	6.25	1.47
0	0	0	0	6.06	3.03	0
0	0	5	5	5	10	0
0	0	0	7.14	0	7.14	0
0	0	0	5	12.5	2.5	5
0	0	0	3.85	3.85	7.69	3.85
0	0	0	1.52	0	10.61	4.55
0	0	0	0	0	0	0
0	0	0	0	0	4.55	4.55
0	0	0	3.7	5.56	5.56	0.93
0	0	0.53	0.53	3.16	5.79	2.63
0	0	0	0	10	0	0
0	0	0	0	5	10	0
0	0	0	0	15	5	0
0	0	0	0	7.69	0	0
0	0	0	8.7	4.35	4.35	0
0	0	0	1.85	5.56	5.56	0
0	0	0	0	0	0	0
0	0	0	8.33	0	16.67	0
0	0	0	0	0	0	0
0	0	0	0	0	7.14	0
0	0	0	9.09	0	0	9.09
0	0	0	0	0	0	0
0	0	0	0	0	8.33	0
0	0	1.67	1.67	1.67	6.67	3.33
0	3.64	1.82	0	0	7.27	0
0	2.9	0	5.8	7.25	2.9	2.9
0	6.67	0	6.67	0	6.67	6.67
0	0	0	0	11.11	0	0
0	2.44	0	2.44	0	12.2	4.88
0	0	0	11.11	11.11	0	0
1.18	0	1.18	3.53	7.06	5.88	1.18
1.71	1.71	1.71	4.27	4.27	5.13	1.71
0	0	0	0	6.9	10.34	0

0	0	0	3.13	0	0	0
1.37	0	1.37	1.37	6.85	13.7	2.74
0	0	0	1.54	1.54	3.08	6.15
0	2.56	2.56	2.56	0	5.13	0
0	0	0	0	0	9.09	0
0	0.67	0	4	1.33	4	2
0	0	0	0	1.79	3.57	3.57
1.92	0.96	0	0.96	0	5.77	0
0	0	2.56	0	5.13	10.26	5.13
0	0	0	0	0	4.76	0
4.55	0	4.55	0	9.09	18.18	0
0	1.45	1.45	4.35	1.45	7.25	1.45
0	1.45	1.45	4.35	1.45	7.25	10.14
0	2.78	2.78	2.78	0	13.89	2.78
0	2	0	10	6	8	0
0	1.05	3.16	1.05	0	4.21	4.21
0	2.36	0	3.15	1.57	3.15	0
0	1.85	0	5.56	1.85	7.41	0
0	2.61	0	3.48	5.22	6.96	2.61
0	2.17	0	2.17	2.17	4.35	0
0	0	0	0	4	12	0
0	0.92	1.83	2.75	5.5	9.17	0
0	0	1.89	1.89	1.89	9.43	7.55
0	0	0	0	0	0	7.14
5	2.5	0	10	17.5	0	0
0	5.13	0	12.82	23.08	0	0
0	0	0	0	0	5.88	0
0	14.29	0	0	0	0	0
0	0	0	25	0	0	0
0	0	0	0	0	3.03	3.03
0	0	2.86	5.71	2.86	5.71	2.86
0	0	3.92	0	0	7.84	5.88
0	4.17	0	8.33	0	8.33	0
0	2.38	0	2.38	0	11.9	0
0	12.5	0	12.5	0	12.5	0
0	3.08	0	4.62	1.54	7.69	3.08
0	1.47	0	1.47	1.47	10.29	2.94
0	0	0	1.61	6.45	4.84	1.61
0	0	0	4.76	0	9.52	0
0	2.78	0	5.56	11.11	11.11	2.78
0	0	0	2.04	4.08	2.04	4.08
0	0	0	0	9.09	9.09	0
4.26	0	0	0	2.13	8.51	0
0	0	0	0	13.64	0	9.09
0	0	0	0	4.35	13.04	0
0	0	0	0	0	10.53	0
0	0	0	0	0	10	0
0	0	4	0	4	4	0
0	0	0	0	7.69	2.56	2.56
0	0	10	0	20	0	0

0	0	0	1.96	11.76	3.92	0
0	0.86	1.72	0.86	5.17	5.17	1.72
0	0	0	0	1.32	5.26	5.26
0	0	5.88	5.88	5.88	11.76	0
0	0	0	0	6.25	15.63	0
0	3.64	3.64	1.82	3.64	7.27	1.82
1.32	0	1.32	0	5.26	3.95	2.63
0	0	0	0	11.11	0	0
0	0	0	3.88	8.74	1.94	0.97
0	0	1.56	0	7.81	4.69	0
0.89	0.89	0	3.57	6.25	8.04	2.68
0	0	2.2	2.2	8.79	1.1	7.69
0	0.9	0	2.7	9.01	4.5	0
0	0	0	2.46	7.38	4.1	4.1
0	0	0	0	9.52	4.76	4.76
0	0.91	0.91	2.73	10	5.45	1.82
0	0	0	0	2.86	9.52	0
0	2.47	0	1.23	6.17	3.7	4.94
0	0	0	0	8.33	16.67	0
0	0	0	0	20	6.67	0
0	0	0	1.85	0	3.7	3.7
0	0	0	0.93	1.87	7.48	0.93
1.61	0	0	3.23	3.23	2.42	3.23
0	0	3.57	0	0	7.14	0
0	0.79	0	5.56	7.94	3.17	1.59
0	1.61	0	1.61	6.45	6.45	3.23
0	0	0	0	5.71	8.57	8.57
0	0	0	3.7	3.7	11.11	0
0	0	0	0	7.14	0	0
0	0	0	0	6.9	6.9	5.17
0	0	0	0	16.95	5.08	0
0	0	0	0	5	0	0
0	0	1.54	0	10.77	0	1.54
0	0	0	0	4.05	6.76	1.35
0	0	1.89	3.77	3.77	11.32	0
1.64	3.28	4.92	3.28	3.28	1.64	4.92
2.44	0	0	0	9.76	4.88	0
0	0	0	0	0	6.45	0
0	0	0	0	10	0	0
1.67	0	1.67	3.33	1.67	3.33	0
0	0	3.03	0	9.09	0	3.03
0	2.63	0	2.63	7.89	10.53	2.63
0	0	0	0	7.69	5.13	0
0	0	0	0	1.3	3.9	1.3
0	4.08	0	1.02	2.04	7.14	2.04
0	0	0	0	4.85	3.88	1.94
0	0	3.33	0	10	6.67	0
0	0	0	0	6	7	0
0	1.35	0	2.7	2.7	6.76	2.7
0	1.2	2.41	2.41	3.61	6.02	4.82

0	0	0	4.55	4.55	4.55	4.55
0	0	0	0	4.69	4.69	0
0	0	2.08	0	4.17	10.42	2.08
0	0	0	0	15.38	0	0
0	0	1.43	0	2.86	2.86	0
0	0	0	0	16.67	0	0
0	0	0	0	3.03	1.52	0
0	2.6	0	1.3	5.19	6.49	3.9
0	0	0	0	7.14	2.38	0
0	0	0	0	8.11	2.7	2.7
0	0	0	0	4.35	8.7	2.17
0	0	0	0	3.85	3.85	0
1.96	0	1.96	0	9.8	0	0
2	0	0	10	6	0	6
1.75	1.75	0	1.75	5.26	3.51	1.75
0	0	0	0	6.9	6.9	0
0	0	0	0	1.92	5.77	3.85
3.13	0	3.13	0	3.13	3.13	3.13
0	3.23	0	9.68	3.23	6.45	0
0	0	1.61	0	1.61	3.23	4.84
2	0	0	0	4	10	0
1.09	0	0	0	2.17	7.61	3.26
3.13	0	0	0	3.13	3.13	0
4.35	0	0	0	8.7	2.17	2.17
0	0	2.13	1.06	11.7	0	3.19
0	2.61	0	0	5.22	6.96	2.61
0	0	0.81	0	4.07	5.69	1.63
0	0	0	0	8.33	4.17	0
0	0	0	0	4	4	12
0	0	0	0	14.58	2.08	0
0	1.2	1.2	0	6.02	4.82	0
4.82	0	0	1.2	7.23	4.82	1.2
0	0	4.88	0	2.44	12.2	2.44
4.44	0	2.22	0	0	13.33	2.22
0.88	1.77	2.65	1.77	11.5	2.65	2.65
3.23	0	0	3.23	6.45	0	0
0	1.33	1.33	0	4	5.33	0
0	0	0	0	3.45	3.45	0
0	1.16	1.16	1.16	8.14	3.49	2.33
0	1.16	1.16	1.16	8.14	3.49	2.33
0	0	1.79	1.79	7.14	3.57	2.68
0	0	0	0	10.34	0	0
1.33	0	0	0	2.67	8	1.33
0	0	0	0	0	4.17	4.17
0	1.64	3.28	0	4.92	4.92	1.64
0	0	0	0	7.41	3.7	5.56
0	0	0	0	2.08	10.42	0
0	0	4.76	0	0	0	0
0	0	2.78	0	8.33	5.56	0
0	0	0	0	7.69	0	0

0	0	0	2.67	4	8	2.67
0	0	0	4.17	12.5	0	0
0	0	0	0	5	5	5
0	0	0	0	3.33	3.33	1.67
0	0	0	0	2.17	0	6.52
4.55	0	0	0	4.55	4.55	4.55
0	0	0	1.22	6.1	2.44	3.66
2.94	0	0	2.94	5.88	5.88	0
0	1.69	0.85	0.85	5.93	6.78	0.85
0	0	5.88	0	0	5.88	11.76
0	0	0	1.09	4.35	4.35	3.26
0	0	0	0	5.88	11.76	0
0	0	1.52	0	9.09	3.03	1.52
0	0	0	2.56	5.13	0	0
0	0	0	0	11.11	1.85	1.85
0	0	6.25	0	6.25	0	0
0	0	0	3.13	3.13	9.38	0
1.25	0	0	2.5	1.25	6.25	1.25
0	0	0	0	0	15.38	0
0	0	0	0	2.78	2.78	0
0	0	0	1.85	1.85	7.41	0
1.34	0	0	4.03	5.37	3.36	0.67
0	0	0	5	0	0	5
0	0	0	5	0	0	5
0	0	0	0	9.76	0	4.88
0	0	0	0	0	16.67	0
1.18	0	0	2.35	3.53	5.88	1.18
0	0	0	0	3.33	0	0
0	0	0	0	6.45	3.23	0
0	0	0	0	11.76	11.76	0
0	0	0	2.8	1.87	5.61	1.87
0	0	0	4.21	3.16	5.26	1.05
1.23	0	0	6.17	2.47	6.17	1.23
0	0	0	0	0	2.63	0
5.41	0	0	2.7	0	8.11	0
0	0	0	1.79	3.57	2.68	3.57
0	0	0	0	4.17	0	0
0	4.76	0	0	4.76	0	0
0	0	0	2.27	11.36	2.27	0
0	0	0	0	3.66	4.88	0
0	0	0	2.5	2.5	5	0
0	0	3.01	3.76	5.26	4.51	0
0	0	0	5.13	23.08	0	0
0	0	3.77	0	11.32	1.89	1.89
1.56	0	0	3.13	6.25	7.81	1.56
0	0	0	2.94	11.76	0	0
0	0	0	0	20.83	0	0
0	0	3.28	0	3.28	16.39	0
0	0	0	0	0	12.5	0
0	0	1	1	5	2	0

1.19	0	2.38	2.38	9.52	4.76	1.19
0	0	0	0	0	3.57	0
0	0	2.22	0	0	6.67	4.44
0	0	1.96	1.96	3.92	11.76	0
0	0	0	0	4.55	4.55	0
0	0	2.63	0	0	7.89	2.63
0	0	0	10	10	0	0
0	0	0	0	10.34	0	3.45
0	0	0	0	11.76	0	0
0	0	11.11	0	0	11.11	0
0	0	0	2.41	12.05	1.2	2.41
0	0	0	0	10	3.33	0
0	0	0	5.56	0	5.56	0
0	0	1.61	0	8.06	3.23	0
0	0	0	0	0	8.51	0
0	0	0	0	6.9	0	0
0	0	1.02	2.04	9.18	3.06	0
0	0	4	0	0	0	0
0	0	0	2.86	0	2.86	0
0	0	0	0	0	0	3.85
0	0	0	0	0	5	0
0	0	7.69	0	0	0	0
0	0	2.04	2.04	4.08	6.12	2.04
0	0	0	0	0	6.25	12.5
0	0	0	0	0	7.69	0
0	0	0	4.26	0	2.13	0
0	0	0	3.45	3.45	6.9	0
0	0	0	3.03	6.06	12.12	0
0	0	0	3.33	0	3.33	0
0	0	0	0	0	9.52	4.76
0	0	0	0	0	6.67	0
1.87	0.47	0	1.87	3.74	3.27	0
0	0	0	0	0.93	6.48	1.85
0	0	0	3.39	1.69	3.39	3.39
0	0	0	0	0	10	2
0	0	0	0	3.45	0	3.45
0	0	0	0	6.06	0	0
0	0	0	0	0	5	10
0	0	0	0	0	7.14	14.29
3.13	0	0	3.13	9.38	0	3.13
0	0	0	0	0	0	0
0	0	0	2.27	2.27	6.82	0
0	0	0	3.64	5.45	14.55	0
0	0	0	0	6.25	0	6.25
0	0	0	0	0	0	4.35
0	0	0	0	12.12	0	3.03
0	0	0	0	9.09	0	0
0	0	0	0	2.74	4.11	2.74
0	0	0	0	9.76	0	0
0	0	0	0	6.25	8.33	2.08

0	0	0	0	0	7.41	0
0	0	0	3.33	0	13.33	0
0	0	3.13	0	6.25	9.38	9.38
0	0	0	0.36	0.72	3.6	0
0	0	0	6	2	6	8
0	0	0	1.85	0	12.96	3.7
0	0	0	11.11	0	0	0
0	0	5	5	0	10	0
0	0	0	0	0	20	0
0	0	0	1.35	2.7	5.41	0
0	0	0	0	0	0	0
0	0	0	10	5	10	0
1.41	0	0	0	2.82	1.41	0
0	0	0	0	5.41	0	8.11
0	0	0	0	12.2	4.88	0
0	0	0	0	10.53	5.26	0
0	0	0	1.43	0	7.14	0
0	0	0	0	0	25	25
0	0	0	1.52	3.03	3.03	4.55
0	0	0	3.57	0	7.14	0
0	0	0	11.11	0	11.11	0
0	0	0	0	8	0	0
0	0	0	0	12.5	6.25	0
0	0	0	3.7	7.41	3.7	7.41
0	0	0	0	6.45	0	0
0	0	0	2.44	12.2	2.44	0
0	0	0	0	11.11	0	0
0	0	0	0	5.88	11.76	0
0	0	0	0	0	10	0
0	0	0	0	0	11.76	11.76
0	0	0	2.5	0	2.5	0
0	0	0	0	0	0	0
1.27	0	0	1.9	1.27	4.43	5.7
0	0	0	1.69	0	13.56	5.08
0	0	0	4.55	0	6.82	2.27
0	0	0	6.38	10.64	4.26	2.13
1.47	0	0	2.94	7.35	4.41	3.68
0	0	0	0.88	3.51	3.51	0.88
0	0	0	0	30	0	0
1.98	0	0	6.93	2.97	6.93	0.99
0	0	0	12.5	0	12.5	0
0	0	0	4.76	4.76	9.52	0
0	0	0	0	0	4	0
0	0	0	6.74	6.74	7.87	0
0	0	0	0	5.81	3.49	2.33
0	0	0	1.27	5.06	3.8	1.27
5.88	0	0	5.88	5.88	5.88	0
0	0	0	0	0	0	0
2.22	0	0	6.67	6.67	4.44	0
0	0	0	0	0	0	0

0	0	0	0	0	5.88	0
0	0	0	1.33	1.33	6.67	4
0	0	0	0	16.67	0	8.33
0	0	0	0	8.89	6.67	2.22
0	0	0	0	13.51	2.7	0
0	0	0	5.41	2.7	0	0
0	0	0	2.38	2.38	2.38	0
0	0	0	0	0	3.7	0
0	0	0	0	0	4	12
0	0	0	0	0	4.76	0
0	0	0	0	5.13	7.69	5.13
1.47	0	0	0	1.47	1.47	4.41
0	0	0	2.94	2.94	5.88	0
0	0	0	4.55	9.09	0	0
0	0	0	0	0	8.33	0
0	0	3.03	0	0	12.12	0
0	0	0	0	0	11.11	0
0	0	0	1.79	3.57	8.93	1.79
0	0	0	11.11	22.22	0	0
0	0	0	0	0	0	0
0	0	0	0	0	14.29	0
0	0	0	4.55	0	4.55	0
0	0	0	0	0	3.7	7.41
0	0	0	3.13	0	9.38	0
0	0	0	0	4.55	4.55	4.55
0	0	0	0	0	4.76	4.76
4.76	0	0	4.76	0	9.52	0
0	0	0	21.43	0	7.14	0
0	0	0	4	12	4	0
0	0	0	0	0	11.11	0
0	0	0	2.8	4.2	4.9	3.5
0	0	0	6.25	0	6.25	0
5.26	0	0	10.53	0	5.26	0
0	0	0	6.67	0	0	0
0	0	0	4.55	4.55	4.55	0
0	0	2.5	0	7.5	10	0
0	0	0	0	2	4	0
0	0	0	2.38	4.76	2.38	0
0	0	0	6.45	0	3.23	0
0	0	0	5.47	7.81	4.69	0.78
0	0	0	0	0	8.33	0
0	0	0	8.7	0	13.04	0
0	0	0	0	0	6.25	0
0	0	0	0	0	0	0
2.59	0	0	1.72	3.45	7.76	5.17
0	0	0	6.67	13.33	4.44	0
0	0	0	3.57	7.14	0	3.57
0	0	0	7.14	0	7.14	0
0	0	0	7.14	0	7.14	0
0	0	0	0	23.08	0	7.69

0	0	0	0	9.09	18.18	0
0	0	0	0	11.11	0	0
0	0	0	0	4.76	2.38	0
0	0	0	4.35	8.7	0	4.35
0	0	0	1.72	12.07	6.9	0
2.38	0	0	2.38	0	4.76	0
0	0	0	0	2.38	0	0
0.77	0	0	2.31	6.15	3.85	0
0	0	0	6.67	13.33	0	0
0	0	0	0	0	4.35	0
0	0	0	0	0	11.11	4.44
0	0	0	0	0	6.25	0
0	0	0	4.23	1.41	7.04	0
0	0	0	0	1.89	1.89	1.89
0	0	0	0	0	12.5	0
0	0	0	0	0	0	0
0	0	0	6.25	0	12.5	6.25
0	0	0	0	0	0	5.56
0	0	0	0	0	4.55	0
0	0	0	0	0	5.41	0
0	0	0	1.69	5.08	6.78	0
0	0	0	2.94	2.94	8.82	0
0	0	0	0	1.02	7.14	4.08
0	0	0	0	4.76	19.05	0
0	0	0	0	0	7.14	0
0	0	0	0	0	15.38	0
0	2.56	0	2.56	12.82	5.13	2.56
0	0	0	0	4.76	0	0
3.45	0	0	3.45	0	3.45	0
0	0.7	0.35	1.4	2.45	4.2	0.7
0	0	0	0	3.33	6.67	0
0	0	0	0	0	0	0
0	0	0	0	5	15	5
0	0	0	0	0	6.45	0
5.56	0	0	5.56	2.78	2.78	0
0	0	0	0	9.52	4.76	0
0	0	0	0	0	0	0
0	0	0	0.6	2.98	3.57	1.19
0	0	0	1.29	7.74	6.45	2.58
0	0	0	0	4.35	6.52	0
0	0	0	0	3.08	7.69	3.08
0	0	0	0	6.67	0	0
4	0	0	4	12	4	0
0	0	0	0	0	23.08	0
0	0	0	0	0	0	0
0	0	0	0	5	10	0
3.45	0	0	3.45	0	6.9	1.15
0	0	0	0	0	7.89	0
0	0	0	1.37	0	6.85	5.48
1.43	0	0	2.86	5.71	5.71	0

0	0	0	3.51	5.26	7.02	0
0	0	0	7.46	2.99	10.45	0
0	0	0	0	5.8	5.8	0
0	0	0	0	0	6.6	0
0	0	0	0	7.14	0	0
1.32	0	0	3.95	5.26	2.63	0
0	0	0	0	4	8	0
0	0	0	3.3	0	4.4	0
0	0	0	0	0	4.65	4.65
0	0	2.7	0	10.81	2.7	0
2.5	0	0	2.5	0	5	0
0	0	0	4.17	4.17	0	0
0	0	0	0	2.94	2.94	0
0	0	0	0	0	0	6.45
0	0	0	0	0	0	0
0	0	0	0	3.13	3.13	0
0	0	0	0	0	9.09	0
0	1.2	0	0	1.2	4.82	0
0	0	0	0	7.84	1.96	0
0	0	0	0	1.45	4.35	1.45
0	0	0	3.06	4.08	3.06	1.02
0	0	0	0	0	0	18.18
1.26	0	0	4.4	2.52	8.81	0
0	0	0	1.22	9.76	2.44	0
0	0	0	0	10	0	0
0	0	0	0	3.03	0	0
1.05	0	0	3.16	6.32	1.05	2.11
0	0	0	3.51	8.77	7.02	3.51
0	0	0	0	11.11	3.7	0
0	0	0	0	5.63	2.82	0
0	0	0	3.88	5.83	2.91	1.94
0	0	0	0	0	10	0
0	0	0	0	30.43	0	0
0	0	0	5.26	1.75	3.51	3.51
0	0	0	2.56	6.41	6.41	0
0	0	0	14.29	14.29	0	0
0	0	0	0	6.25	0	0
2.86	0	0	2.86	2.86	2.86	0
0	0.91	0	3.64	2.73	6.36	1.82
0	0	0	4.7	10.74	2.68	1.34
2.94	0	0	0	5.88	8.82	0
0	0	0	2.99	1.49	7.46	4.48
0	0	0	0	2.78	11.11	0
0	0	0	9.09	0	18.18	0
1.35	0	0	1.35	8.11	1.35	0
0.73	0	0.73	1.46	3.65	6.57	0
0	0	0	3.77	15.09	1.89	0
0	0	0	0	0	3.33	0
0	0	0	4.55	4.55	4.55	0
0	0	0	0	8.33	8.33	0

0	0	0	0	0	0	0
0	0	0	4	4	0	4
0	0	0	0	11.11	0	0
0	0	0	0	0	11.76	0
0	0	0	0	7.69	7.69	0
0	0	0	0	12.5	0	0
0	0	0	15.38	7.69	0	7.69
1.85	0	0	3.7	7.41	1.85	1.85
0	0	0	4	12	0	0
0	0	0	0	6.67	6.67	0
0	0	0	0	2.56	10.26	0
0	0	0	0	0	8.11	0
0	0	0	5.88	0	5.88	0
0	0	0	0	10	0	0
0	0	0	0	0	33.33	0
0	0	0	0	0	0	0
0	0	0	1.37	1.37	6.85	5.48
0	0	0	0	6.9	13.79	0
1.91	0	0	3.82	3.82	6.37	1.91
0	0	0	2.78	2.78	5.56	0
0	0	0	0	12.9	0	0
0	0	0	0	0	11.11	0
0	0	0	0	9.09	0	9.09
0	0	0	0	5	7.5	0
0	0	0	0	0	15.38	0
0	0	0	0	0	0	0
0	0	0	0	25	0	0
0	0	1.37	1.37	1.37	8.22	1.37
0	0	0	0	2.04	6.12	0
0	0	0	0	13.33	6.67	6.67
0	0	0	0	7.32	0	0
0	0	0	0	7.14	0	0
0	0	0	0	0	0	0
0	0	0	0	0	10	0
0	0	0	2.7	0.9	4.5	1.8
0	0	0	0	3.85	11.54	0
0	0	0	11.32	15.09	1.89	1.89
0	0	0	0	18.18	0	0
0	0	0	0	36.36	0	0
0	0	2.56	2.56	0	2.56	0
0	0	0	0	0	7.14	0
0	0	0	4	12	4	8
0	0	0	0	0	0	0
0	0	0	6.25	12.5	0	0
0	0	0	0	0	0	0
1.52	0	0	3.03	6.06	3.03	1.52
0	0	0	4.35	13.04	0	0
6.25	0	0	6.25	2.08	4.17	4.17
0	0	0	0	10	10	0
3.39	0	0	8.47	1.69	8.47	0

0	0	0	0	0	0	0
2.47	1.23	1.23	2.47	4.94	3.7	0
0	0	0	3.7	3.7	11.11	0
0	0	0	4.35	17.39	0	0
0	0	0	0	12	8	0
0	0	0	0	16.67	0	0
0	0	0	0	0	10.53	0
0	0	0	0	0	12.5	0
0	0	0	0	0	10	0
0	0	0	0	11.11	0	0
0	0	0	0	30	0	0
0	0	0	0	0	16.67	0
0	0	0	0	3.13	3.13	0
0	0	0	6.25	0	6.25	0
0	0	0	5.26	5.26	0	0
0	0	0	0	10	0	0
0	0	0	7.14	7.14	7.14	0
0	0	0	0	0	0	0
0	0	0	6.67	6.67	0	0
0	0	0	2.94	0	8.82	0
0	0	0	4.65	0	2.33	0
0	0	0	5.88	5.88	0	0
2.63	0	0	5.26	2.63	3.95	1.32
0	0	0	3.61	1.2	4.82	1.2
0	0	0	0	17.65	0	0
0	0	0	0	0	10.53	5.26
0	3.03	0	0	9.09	0	0
0	0	0	0	0	10.34	0
1.2	0	0	3.61	1.2	7.23	4.82
0	0	0	3.13	12.5	3.13	0
1.64	0	0	2.46	7.38	9.02	0
0	0	0	0	0	11.76	0
0	0	0	0	0	4.76	0
1.23	0	0	7.41	0	6.17	2.47
0	0	0	0	0	5.26	5.26
0	0	0	0	0	8.7	4.35
0	0	0	0	0	8.33	0
0	0	0	0	6.67	0	0
0	0	0	1.92	9.62	3.85	3.85
2.86	5.71	0	5.71	0	8.57	0
4.76	0	0	4.76	0	4.76	0
0	0	0	0	0	0	0
0	0	0	0	0	22.22	0
0	0	0	5.56	5.56	5.56	5.56
0	0	0	0	4	0	0
0	0	0	0	5.06	5.06	2.53
0	0	0	0	0	20	0
0	0	0	0	0	12.5	0
0	0	0	0	3.7	5.56	0
0	2.78	0	0	5.56	5.56	0

0	0	0	0	0	0	5.56
0	0	0	0	3.03	3.03	9.09
10	0	0	20	5	0	0
0	0	0	0	6.67	6.67	0
0	0	0	6.78	1.69	1.69	5.08
0	0	0	5.13	5.13	2.56	2.56
0	0	0	0	18.18	9.09	0
0	0	0	0	16	8	0
0	0	0	0	0	28.57	0
0	0	0	0	0	9.09	0
0	0	0	6.25	2.08	2.08	0
0	0	0	5.56	0	11.11	0
0.73	0	0	2.92	8.39	5.11	0.36
2.56	0	0	2.56	5.13	2.56	0
0	0	0	0	0	7.41	7.41
0	0	0	17.39	6.52	0	0
0	0	0	0	0	8	0
0	0	0	0	5	7.5	0
0	0	0	5.26	0	0	0
0	0	0	0	14.29	0	0
0	0	0	0	0	20	0
0	0	0	1.75	17.54	1.75	0
0	0	0	0	0	0	7.14
1.64	0	0	4.92	3.28	1.64	0
0	0	0	3.33	0	3.33	0
0	0	0	3.77	8.49	0.94	0.94
1.15	0	0	0	0	5.75	2.3
0	0	0	0	0	4.55	0
0	0	0	0	0	20	0
0	0	0	3.64	10.91	9.09	5.45
0	0	0	0	3.57	7.14	0
0	0	0	0	0	20	0
0	0	0	3.2	0	8	0
0	0	0	3.23	6.45	0	0
0	0	0	0	3.7	7.41	0
0	0	0	0	0	7.14	0
0	0	0	11.11	0	3.7	0
0	0	0	1.15	4.02	7.47	2.87
0.52	0	0	1.04	11.98	3.13	2.6
0	0.6	0.6	0.9	11.38	1.5	0.6
0	0	0	0	3.45	10.34	0
0	0	0	0	7.69	0	0
0	0	0	0	7.81	6.25	1.56
0	0	0	0.61	2.44	4.88	1.83
0	0	0	0	5.26	4.39	0.88
0	0	0	0	0	16.67	0
2.15	0	0	2.15	8.6	6.45	0
0	0	0	3.33	3.33	3.33	0
0	0	0	6.67	6.67	20	0
0.79	0	0	2.38	8.73	3.97	0

0	0	0	1.72	11.21	0.86	7.76
0	0	0.3	1.22	0.91	6.08	1.52
0	0	0	0	6.52	1.09	1.09
0	0	0	0	2.33	9.3	0
0	0	0	0.7	6.99	2.1	1.4
0	0	0	1.52	1.52	4.55	1.52
0	0	0.75	0.38	9.4	3.76	0
0.69	0	0	1.38	6.21	7.59	0.69
0	0	0	0	1.85	5.56	0
0	0	0	0	0	0	0
0	1.64	0	1.64	4.1	10.66	0
0	0	0	0	0	2.94	0
0.96	0.96	0	1.92	3.85	2.88	0.96
0	0	0	0	0	5.88	0
0	0	1.89	3.77	1.89	1.89	0
0	0	0	0	0	0	0
0	0	0	0	3.77	5.66	0
0	0	0	0	0	0	0
0	0	0	2.78	0	8.33	0
0	6.82	0	0	0	11.36	0
0	0	0	2.04	0	6.12	0
0	0	1.47	0	1.47	1.47	0
7.14	0	0	7.14	0	21.43	0
0	0	0	0	5.56	2.78	0
0	2.08	0	0	6.25	8.33	0
0	1.1	0	0	0	5.49	3.3
0	0	0	0	6.25	6.25	1.25
0.44	0.88	0.44	0.44	1.32	4.85	1.76
0	4.08	0	2.04	4.08	4.08	0
0	0	0	0	0	11.11	0
0	0	0	0	15.38	0	0
0	0	0	0	0	12.5	0
0	0	0	0	4.55	0	4.55
12.5	0	0	25	0	0	0
0	0	0	0	0	20	0
0	0	0	0	0	8.7	0
0	0	1.04	0	2.08	4.17	0
0	0	0	1.69	0	6.78	0
0	0	0	4.88	4.88	4.88	0
0	0	0	0	6.52	6.52	0
0	1.79	1.79	0	7.14	3.57	0
0	0	2.86	2.86	0	11.43	0
0	0	0	0	0	14.29	0
0	0	0.55	1.91	2.46	10.11	1.09
0	1.72	0	0	5.17	1.72	1.72
0	0	0.78	0	5.47	6.25	0.78
0	0	0	0	0	23.08	0
0	0	0	0	0	10	0
0	0	0	0	0	0	0
0.36	0	0.36	0.36	5.4	3.6	0.72

0	0	0	0	0	0	0
0	0	0	0	0	10.53	0
0	0	0	0	0	22.22	0
0	0	0	0	0	0	0
0	0	0	2	2.67	8	0
0	0	0	3.23	9.68	6.45	0
0	0	0	0	0	20	0
0	0	0	0	4.21	11.58	1.05
0	0	0	0	8.65	0.96	0
0	0	0	0	0	12.5	0
0	0	0	0	8.7	0	0
0	0	0	0	12.5	0	0
0	0	1.52	0	3.03	6.06	3.03
0	0	0	0	0	8.33	2.08
0	0	1.82	0	1.82	3.64	10.91
0	0	0	0	3.33	13.33	0
0	0.85	0.85	0	6.84	3.42	0
0	0	0	0	1.67	5	0
0	0.61	0	0.61	2.44	4.88	0
0	0	0	0	3.28	4.92	0
0	0	0	1.92	3.85	3.85	1.92
0	0	0	0	1.3	12.99	1.3
0	0	0	2.26	1.5	4.51	1.5
0	0	0	4.92	6.56	4.92	4.92
0	0	0	0	10	5	15
1.19	0	0	1.19	1.19	9.52	0
0	0	0	12.5	0	25	0
0	2.78	0	0	5.56	0	0
0	0	0	0	0	6.25	0
0	1.59	1.59	0	0	7.94	0
0	0	0	0	4.17	8.33	0
0	0	0	0	6	4	4
0	0	5.88	0	5.88	11.76	0
0	0	0	0	0	9.84	0
0	0	0	0	0	7.46	5.97
0	1.59	0	0	0	6.35	3.17
0	0	0	1.37	0	6.85	1.37
0	0	0	0	0	8.33	0
0	0	0	0	3.33	3.33	0
0	0	0	0	0	10.53	0
1.79	0	0	1.79	3.57	5.36	0
0	0	0	0	0	5.45	0
0	0	0	0	6.56	3.28	0
0	0	0	0	0	10.34	0
1.54	0	0	0	7.69	0	0
0	0.81	0	2.44	3.25	8.13	1.63
0	0	0	0	0	7.69	0
1.06	0	0	0	7.45	3.19	0
0	0	0	6.25	0	0	0
0	0	0	0	0	33.33	0

1.16	0	0	1.16	2.33	4.65	4.65
0	0	0	0	12.82	0	2.56
0	0	0	0	4	8	0
1.06	0	0	1.06	7.45	6.38	0
0	0	0	1.54	3.08	1.54	1.54
0	0	0	2.27	0	2.27	0
0	0	0	0	5.88	11.76	0
0	0	1.61	4.84	3.23	12.9	0
0	0	13.33	0	6.67	6.67	0
0	0	1.35	0	2.7	5.41	0
0	0	0	0	1.53	8.4	0.76
0	0	0	1.35	5.41	4.05	5.41
0	0	0	3.7	11.11	3.7	0
0	0	0	2.94	7.35	5.88	1.47
0	0	0	0	0	9.26	5.56
0	0	0	0	2.67	4.67	0
0	0	0	2.78	2.78	8.33	1.39
0	0	0	0	0	4	12
0	0	0.76	1.52	10.61	0	0
0	0.64	0	3.18	0	8.28	1.27
0	0	0	0	3.7	7.41	0
0	0	0	0	1.96	11.76	5.88
0	0	0	0	0	6.82	0
0	0	0	0.54	4.35	5.43	3.26
0	0	0	0	0	5.88	3.92
0.69	0.69	0.69	0.69	3.45	6.21	1.38
0	0	0	0	0	15	0
0	0	0	0	6.25	9.38	3.13
0	0	1.43	0	1.43	10	0
0	0	0	2.38	11.9	2.38	0
0	0	0	0	0	10.91	3.64
0	0	0	0	2.5	12.5	0
0	0	0	1.72	0.57	7.47	0.57
0	3.23	0	0	0	9.68	0
0	0	0	2.04	0	4.08	0
0	0	0	0	0	8.82	0
0	0	1.89	0	0	11.32	0
0	0	0	0	0	11.11	0
0	0	0	0	4.35	8.7	0
0	0	0	0	5	10	0
0	0	0	0	1.89	15.09	0
0	0	0	0	0	0	0
0.81	0	0	3.23	9.68	4.84	1.61
0	0	0	2.54	6.78	9.32	0
0	0	0	0	0.66	7.95	1.99
0	0	0	0	0.86	8.62	1.72
1.85	0	0	4.63	4.63	10.19	1.85
0	0	0.67	0.67	1.33	4	2.67
0	0	0	1.59	3.17	6.35	0
0	0	9.09	0	0	18.18	0

0	0	1.25	0	2.5	8.75	0
0	0.95	0	1.43	8.1	2.38	0.95
0	2.63	2.63	5.26	5.26	5.26	2.63
0	2.08	0	2.08	2.08	6.25	0
0	0	0	0	0	13.33	0
0	16.67	0	16.67	16.67	0	0
1.54	2.31	0.77	3.08	4.62	3.85	0.77
0	4.76	0	4.76	19.05	0	0
3.03	0	9.09	3.03	0	9.09	0
2.78	0	0	5.56	8.33	0	0
0	0	0	0	16.36	1.82	0
0	0	0	0	0	16.67	0
0	0	0	0	18.75	6.25	0
0	1.25	1.25	0	2.5	10	3.75
0	2.7	0	5.41	21.62	0	0
0	0	2.5	0	2.5	2.5	2.5
0	0	0	5.26	7.89	5.26	0
0	0.88	0	0.88	6.14	6.14	0
0	0	0	5	15	5	0
0	6.25	0	6.25	18.75	6.25	0
2.56	0	0	2.56	12.82	0	0
0	3.85	0	3.85	19.23	0	0
0	3.03	0	3.03	16.67	0	0
0	0	0	0	2.27	11.36	4.55
0	0	0	0	0	16.67	0
10	0	10	0	10	0	0
0	0	0	2.44	4.88	0	0
0	0	5.88	0	5.88	0	0
0	0	0	0	0	10	0
0	0	0	0	14.1	2.56	0
0	0	0	2.14	11.43	0	3.57
0	0	0	1.54	13.85	0	1.54
0	1.25	0	0	6.25	12.5	0
0	0	0	0	5.88	2.94	0
0	0	0	2.94	2.94	8.82	2.94
1.96	1.96	0	3.92	17.65	1.96	1.96
0	0	0	0	16.67	0	0
1.2	0.6	1.2	1.81	6.63	1.81	1.2
0	1.69	0	3.39	13.56	0.85	1.69
0	0	10	0	10	10	0
0	0	0	0	0	20	0
0	0	0	0	0	0	6.25
0	0	0	0.7	2.11	6.34	2.11
0	0	0.82	0	13.11	3.28	0
0	1.82	0	5.45	18.18	5.45	0
0	1.09	1.09	1.09	0.55	7.65	3.83
0	0	0	8.33	2.08	10.42	4.17
0	0	0	0.83	9.09	4.96	1.65
0	4.35	4.35	4.35	8.7	4.35	4.35
0	0	0	0	16.67	0	0

0	0	0	0	4.62	4.62	1.54
0	0	0.58	0.58	10.4	1.73	1.16
0	0	0	1.5	14.29	1.5	1.5
1.05	1.05	1.05	5.26	5.26	1.05	4.21
0.24	0.97	0.24	2.68	8.76	3.41	1.46
0	10	10	0	0	0	0
0.49	1.48	0	1.48	7.39	1.97	0.99
0	0	0	2.03	8.12	5.08	1.52
0	0	0	0	0	8.33	8.33
0	0	0	0	0	4	0
0	0	5.26	0	0	10.53	0
0	0	0	0	0	18.18	0
3.85	0	0	7.69	0	11.54	0
0	0	0	0	0	0	0
0	0	0	0	10.34	1.72	0
0	0	0	0	0	10	0
0	0	0	0	11.11	0	0
0	0	0	0	28.57	14.29	0
0	0	0	0	3.28	1.64	1.64
0	0	0.47	0.95	6.64	4.27	0.95
0	0	0	0	2.27	2.27	0
0	0	0	6	2	4	4
0	0	0	0	3.03	6.06	0
0	0	0	2.3	21.84	0	0
0	0	0	0	0	8.89	0
0	0	0	2.76	2.07	3.45	0.69
0	0	0	0	0	6.67	0
0	0	0	3.33	13.33	3.33	0
5.13	0	0	10.26	0	5.13	0
0	0	0	3.7	0	7.41	0
0	0	0	10	0	0	0
0	0	0	3.85	0	0	0
0	0	0	2.27	4.55	6.82	4.55
0	0	0	0	0	0	10
0.76	0	0	3.82	3.05	7.63	0
2.04	0	0	2.04	2.04	12.24	0
0	0	0	3.7	1.23	11.11	0
0	0	0	0	0	9.38	0
0	0	1.85	3.7	0	5.56	0
0	0	2.22	4.44	0	4.44	0
0	0	3.33	6.67	3.33	10	0
0	0	7.69	7.69	0	15.38	0
0.74	0	0	8.15	3.7	5.93	1.48
0	0	0	0	0	18.18	0
0	0	0	5.71	5.71	5.71	0
0	0	0	0	0	20	0
0	0	0	3.33	3.33	8.33	1.67
0	0	0	3.57	0	7.14	3.57
0	0	0	1.9	4.76	8.57	1.9
0	0	0	0	5.71	8.57	0

0	0	0	5.13	7.69	2.56	0
1.06	0	0	3.19	12.77	0	0
0	0	0	2.38	7.14	2.38	0
0	0	1.49	1.49	0	11.94	2.99
0	0	0	2.56	5.13	10.26	0
0	0	0	2.5	2.5	2.5	2.5
0	0	0	0	0	7.04	4.23
0	0	0	3.57	0	17.86	0
0	0	0	3.23	12.9	3.23	0
0.47	0	0.93	4.19	7.91	3.26	1.86
0	1.15	0	2.3	3.45	9.2	2.3
0	0	0	0	0	0	9.09
0.61	0	0	3.68	1.23	6.75	1.23
0	0	0	0	0	0	0
0	0	0	3.15	2.36	5.51	0.79
2.86	0	0	5.71	5.71	11.43	0
0	0	0	7.14	0	14.29	0
0	0	0	7.69	0	0	0
0	0	7.14	28.57	0	0	0
8.33	0	0	4.17	4.17	8.33	0
0	0	0	0	0	6.06	6.06
0	0	0	4.35	2.17	4.35	4.35
0	0	0	0	20	0	0
0	0	3.57	3.57	3.57	10.71	0
0	0	5.41	5.41	0	2.7	0
0	0	0	6.06	3.03	6.06	0
0	0	0	0	0	11.11	0
0	0	0.75	3.01	4.51	3.76	1.5
0.71	0	0	2.14	2.85	8.19	0.71
0	0	1.25	2.5	5	10	1.25
0	0	0	4.17	4.17	8.33	0
0	0	0	5.15	0	5.88	2.21
0	0	0	11.11	0	5.56	0
0	0	0	3.7	14.81	7.41	0
0	0	0	0	0	9.68	0
0.16	0.16	0.65	2.59	1.62	6.48	1.94
0	0	0	1.61	0.81	8.06	1.61
0	0	0	4.42	1.66	5.52	3.87
0	0	0.6	3.59	5.39	5.99	1.8
0	0	0	0	2.04	4.08	0
0	0	0	2.35	3.53	9.41	2.35
0	0	3.03	0	3.03	0	0
0	0	0	5	2.5	11.25	3.75
0	0	0	3.45	6.9	5.17	0
0	0	0	0	1.82	5.45	0
1.28	0	0	2.56	1.28	5.13	2.56
0	0.33	0.66	3.65	6.64	4.98	1
0	0	0	0	8.33	0	0
0	0	0	0	0	7.14	0
0	0	0	3.85	0	11.54	0

0	0	0	0	10.53	0	5.26
1.01	0	0	0	6.06	5.05	1.01
1.35	0	0	5.41	2.7	5.41	2.7
0	0	0	0	0	5	0
0	0	0	0	0	12.5	0
0	0	0	0	5.33	5.33	1.33
0	0	0	2.08	8.33	0	8.33
0	0	0	3.45	3.45	12.07	3.45
1.59	0	0	0	3.17	4.76	0
0	0	0	4.95	6.93	1.98	0.99
0	0	0.94	0.94	2.83	9.43	0
0	0	0	4	8	4	4
0	0	0	4.35	8.7	8.7	0
0	2.16	0.72	3.6	14.39	2.16	0.72
0	0	0	0	8.33	8.33	0
0	0	0	2.63	0.66	6.58	0
0	0	0	0	0	6.67	0
0	0	0	0	15	2.5	0
0	0	0	0.88	3.51	3.51	3.51
0	0	0	3.13	3.13	6.25	3.13
0	3.75	0	2.5	17.5	0	0
0	0	0	0	2	6	2
0	0	3.57	1.79	1.79	3.57	0
0.68	0	1.35	2.7	9.46	5.41	2.03
0	0	0	1.01	8.08	2.02	1.01
0	1.65	0	4.96	8.26	4.96	0
0	0	0	0	9.09	18.18	0
0	2.07	0	1.55	8.29	4.66	2.07
0	5.56	0	0	16.67	0	0
0	0	0	0	9.52	2.38	0
0	0	0	0	0	4.76	0
1.01	0	0	1.68	4.36	5.37	0.67
0	0	0	0	16.67	1.52	3.03
0	1.04	3.13	0	3.13	5.21	0
0	0	9.09	0	0	18.18	0
0	0	0	2.13	2.13	2.13	0
0	0	0	0	0	0	0
0	0	8.33	0	0	25	0
0	0	16.67	0	0	0	0
0	0	3.85	0	0	0	3.85
0	0	0	0	0	5.88	5.88
0	0	0	0	0	15	10
0	9.09	9.09	0	0	4.55	0
0	0	0	0	0	0	0
0	0	8.33	0	0	8.33	0
0	0	0	0	0	25	0
0	4	6	2	0	12	0
0	0	0	0	2.7	5.41	0
0	0	0	0	16.22	0	0
0	0	0	0	0	12.5	0

0	0	0	2.96	0.74	5.19	2.22
0	0	4.26	2.13	2.13	8.51	0
0	2.78	2.78	0	0	2.78	5.56
0.46	0	0.23	3.21	11.24	3.67	1.15
1.25	0	0	1.25	6.25	3.75	0
0	0	0	0	0	0	0
0	0	0	0	10	0	0
2.44	0	0	2.44	4.88	4.88	0
2.56	0	0	0	7.69	7.69	0
0	0	0	3.03	3.03	3.03	0
0	0	50	0	0	50	0
0.29	0.1	0.39	2.25	4.11	4.6	2.15
0	0	0	0	6.45	1.08	1.08
0	0	0	0.79	3.97	5.56	0.79
0	0	0	1.37	2.74	2.74	1.37
0	0	0	0	0	0	0
0	0	0.81	0.81	5.69	2.44	0
0	0	0	0	0	0	0
0	0	0	0	0	0	0
0	0	0	0.94	5.66	5.19	1.89
0	0	0	0	8.33	0	0
0	0	0.41	0.82	4.49	5.31	1.22
0	0	0	0	0	0	0
0	0	0	1	1	8	1
0	0	0	0	0	2.94	0
0	1.16	0	2.09	2.78	5.57	2.32
0	0	0	0	4.92	6.56	0
2.22	1.11	0	5.56	1.11	4.44	3.33
0	0	0	8.7	13.04	0	0
0	0	0	1.53	6.63	2.55	0
0	0	0	0	0	13.16	0
0	0	0	0	0	0	9.09
0	0	2.44	0	0	7.32	0
0	0	0	1.99	1.99	5.3	1.99
0	0	0	16.67	0	16.67	0
0	0	0	0	1.3	5.22	2.61
0.23	0	0	1.58	2.7	6.08	1.58
0	0	0	0	1.82	0	1.82
0	0	0	7.69	11.54	3.85	7.69
0	0.72	0.72	1.44	2.16	7.19	1.44
0	0	0	4	8	4	0
2.5	0	0	5	10	2.5	2.5
0.36	0.36	0.36	0.71	4.98	4.98	0.71
0	0	0	5.26	5.26	2.63	5.26
0	0.89	0	3.57	0	3.57	0.89
0	0	0	0.81	6.45	4.84	1.61
0	0	0.95	0	6.67	5.71	0.95
0.42	0	2.51	0.84	1.67	5.44	2.09
2.94	0	0	3.92	1.96	2.94	0.98
0	0	0	0.93	7.48	3.74	0.93

0	0	0.93	2.8	0.93	6.54	0
0	0	0	2.63	2.63	5.26	0
0	0	0	4.76	0	4.76	4.76
0	0	0	2.34	0	2.92	1.17
0	0	0.97	1.94	0	3.88	0
0	0	0	0	0	0	0
0	0	0.98	0	0.98	1.96	0
4.08	0	0	6.12	0	2.04	2.04
0	0	0	0	0	22.22	11.11
0.69	0.23	0	2.77	8.78	2.77	0.46
0	0	0	0.95	7.62	2.86	1.9
0	0	0	1.14	5.11	8.52	0.57
0	0	0	0	0	15.38	1.54
0	0	0	0	6.36	5.45	0.91
0	0	0.43	1.71	3.85	5.13	0.85
0.85	0	0	2.56	0	5.98	0.85
0	0	0	0.98	1.95	8.78	4.39
0	0	0	2.88	1.92	5.77	2.88
0	0	0	1.74	3.48	9.57	0.87
0	0	0	0	8.33	1.19	1.19
0	0	0	0	0	6.06	0
0	0	0.59	1.76	1.76	7.06	2.35
0	1.61	0	3.23	3.23	6.45	3.23
0	0	0	3.39	5.08	3.39	0
0	0	0	1.22	0	7.32	0
0	0	0	5.88	2.94	5.88	5.88
0	0	0.38	1.15	4.21	5.75	2.3
0	0	0	0.78	0	6.25	0.78
0	0	0	0	3.45	10.34	0
0	0	1.99	2.65	3.31	5.96	3.31
0	0	2.27	2.27	2.27	4.55	4.55
0	0	0	0	5	7.5	0
0	0	0	0	1.74	3.48	2.61
0	0	0	1.16	1.73	7.51	1.16
0	0	0	1.67	3.33	10	0
0	0	1.8	1.8	4.5	4.5	0.9
0	0	0	1.16	8.14	6.98	0
0	0.11	0.11	0.88	5.26	5.59	2.3
0	0	0	1.01	7.07	4.04	3.03
0	0	0	0.71	3.55	5.67	0
0	0	0	2.78	1.39	6.94	2.78
0	0	0	0	5	0	10
0	0	0	1.64	1.64	8.2	1.64
0	0	0	4	0	5.33	2.67
0	0	0	0	5	3.33	0
6.45	0	0	3.23	3.23	3.23	3.23
0	0	0	2.44	0	12.2	0
0	0	0	1.08	3.23	8.6	2.15
0	0	2.83	1.89	6.6	9.43	1.89
0	0	0	0	3.23	6.45	1.61

1.75	0	0	0	5.26	7.02	0
0	0	0	1.3	0	9.09	0
0	0	0	1.96	1.96	6.86	0.98
0	0	0	0.99	0	4.93	0.49
0	0	0	0	6.06	6.06	0
0	0	0	0	0	4.17	0
